# Effects of whole-body vibration training on muscle performance in healthy women: A systematic review and meta-analysis of randomized controlled trials

**DOI:** 10.1371/journal.pone.0322010

**Published:** 2025-05-30

**Authors:** Bopeng Qiu, Ziyu Wang, Mingyue Yin, Jinghan Feng, Penglin Diao, Juan Del Coso, Redha Taiar

**Affiliations:** 1 School of Strength and Conditioning, Beijing Sport University, Beijing, China; 2 College of Swimming, Beijing Sport University, Beijing, China; 3 School of Athletic Performance, Shanghai University of Sport, Shanghai, China; 4 Sports Business School, Beijing Sport University, Beijing, China; 5 Sport Sciences Research Centre, Rey Juan Carlos University, Fuenlabrada, Spain; 6 MATériaux et Ingénierie Mécanique (MATIM), Université de Reims Champagne-Ardenne, Reims, France; Universita degli Studi di Verona, ITALY

## Abstract

**Objective:**

This study aimed to perform a comprehensive meta-analysis of randomized controlled trials examining the effectiveness of whole-body vibration training (WBVT) on muscle performance in healthy women.

**Methods:**

A systematic search of studies available up to 30 May 2024 was conducted using seven databases, including PubMed, EMBASE, Web of Science, Scopus, CINAHL, PEDro, and the Cochrane Library. Studies with a randomized and controlled protocol in which the effect of WBVT on muscle performance variables was compared to that of a) a non-exercise intervention or b) exercise intervention in healthy women were assessed for eligibility. The methodological quality of the included studies was assessed using the PEDro scale. Meta-analyses were performed using random effects models, and the results were expressed as standardized mean differences (SMDs) with corresponding 95% confidence intervals (95% CIs).

**Results:**

A total of 21 randomized controlled trials, encompassing 748 healthy women, was included in the meta-analysis. WBVT demonstrated significantly greater effects on muscle strength and power when compared with the non-exercise control groups with regard to knee extension (SMD = 0.534, 95% CI: 0.303 to 0.766, *p* < 0.001), leg press (SMD = 0.794, 95% CI = 0.424 to 1.163, *p* < 0.001), ankle plantar flexion (SMD = 0.462, 95% CI: 0.019 to 0.904, *p* = 0.041), and the countermovement jump performance (SMD = 0.470, 95% CI: 0.211 to 0.729, *p* < 0.001). However, WBVT significantly improved only the countermovement jump performance (SMD = 0.338, 95% CI: 0.037 to 0.640, *p* = 0.028) when compared with the exercise control groups. Subgroup analyses revealed that longer periods (≥ 12 weeks) of WBVT resulted in greater benefits for both muscle strength and power compared to the non-exercise control group. Additionally, higher frequencies (> 30 Hz, SMD = 0.736, *p* < 0.001; ≤ 30 Hz, SMD = 0.284, *p* = 0.109) provided greater benefits for improving muscle strength. Last, post-menopausal women (post-menopausal, SMD = 0.561, *p* = 0.001; pre-menopausal, SMD = 0.354, *p* = 0.076) obtained greater benefits in muscle power with WBVT than pre-menopausal women.

**Conclusions:**

WBVT is efficacious in improving lower-body muscle strength and power in healthy women. However, the potential benefits of WBVT compared to other exercise interventions were only associated with an enhancement in countermovement jump performance. Longer periods (≥ 12 weeks) of WBVT resulted in greater benefits for both muscle strength and power compared to the non-exercise control group. Additionally, higher vibration frequencies (> 30 Hz) provided greater improvements in muscle strength, while post-menopausal women reaped greater benefits in muscle power than pre-menopausal women.

## Introduction

Muscle strength and power are crucial not only for daily activities but also for maintaining good health and preventing chronic diseases [1–4]. Recent investigations have suggested that muscle strength can be considered a lifelong indicator of general health [5,6]. For instance, low levels of muscle strength, indicated by low age- and sex-corrected values of leg extension and handgrip strength, is linked to an increased risk of disability [7]. Deficits in upper or lower body muscle strength are linked to a higher risk of all-cause mortality in both healthy young adults and older adults [8–10]. Additionally, muscle power (power = force * velocity) correlates with muscle strength [11,12]. With aging, a reduction in motor units leads to a decline in muscle power, preceding the decrease in muscle strength [13]. Consequently, assessing muscle power might be more sensitive in detecting the onset of a decline in physical function [4]. These outcomes point toward the potential health benefits of maintaining muscle strength and power throughout life for both men and women [1,14].

In recent years, whole-body vibration training (WBVT) has received much scientific and clinical attention as a potentially effective form of strength training [15]. WBVT typically involves static or dynamic exercise on a vibrating platform [16–18]. In WBVT, the vibration stimulus is delivered to the body via a vibrating platform while the mechanical vibration signal activates the muscle spindle, thus activating α-motor neurons [19,20]. Additionally, the vibration reduces the inhibitory response of the Golgi tendon organ to motor neurons, thus triggering the same muscle contraction as the tonic vibration reflex [21–23]. When compared with other strength training methods, WBVT has several advantages; these include being safer and less physically demanding over the long term [24–26], and being beneficial for body composition, cardiorespiratory fitness, and quality of life [27–29]. This training modality can serve as an alternative or complementary strategy to traditional training methods for healthy or trained individuals [26]. Additionally, WBVT can be used as a therapy for clinical populations to treat some conditions as it may improve bone mineral density, cognition, and functional abilities, as well as the reduction of the level of pain and risk of falls [30].

Several systematic reviews and meta-analyses have reported that WBVT has benefits on various outcomes related to muscle strength and power [24,25,31–36]. However, some of these systematic reviews and meta-analyses [24,25,32–34] have compared WBVT protocols with non-exercise control groups, which has possibly influenced the effect of WBVT on muscle strength and power, at least in comparison to other forms of strength training. Moreover, when testing the efficacy of WBVT in women, existing research has produced conflicting results, some studies find improvements in strength and power [37,38], while others do not [39,40]. Collectively, these findings suggest the need to determine the specific effects of WBVT on muscle strength and power in women compared to other strength training [38–58]. This information is key to understanding the efficacy and practical application of WBVT in exercise programs for women.

As far as we know, no systematic reviews and meta-analyses have comprehensively evaluated the effects of WBVT on muscle strength and power in women, particularly in comparisons between WBVT and both non-exercise and exercise control groups. Therefore, this study aimed to perform a comprehensive meta-analysis of randomized controlled trials examining the effectiveness of WBVT on muscle performance in healthy women, contrasting the changes induced by WBVT with different control groups (non-exercise and exercise control groups). Additionally, we investigated the potential moderating effects of various training protocol parameters. We hypothesized that WBVT would significantly enhance muscle strength and power in healthy women compared to non-exercise controls and may offer additional benefits over traditional exercise, particularly in countermovement jump performance. We also hypothesized that certain training protocol parameters, such as duration and vibration frequency, would be crucial for maximizing the benefits of WBVT.

## Methods

This systematic review and meta-analysis was conducted following the Preferred Reporting Items for Systematic Reviews and Meta-Analyses (PRISMA) 2020 statement [59]. The systematic review was registered on the International Prospective Register of Systematic Reviews (CRD42023415971).

### Search strategy

The search strategy utilized both medical subject headings (MeSH) and free-text keywords to identify key concepts: Concept 1 (Women OR Female) AND Concept 2 (Whole-body vibration OR WBV) AND Concept 3 (Strength OR Power). A detailed search was conducted using seven databases, including PubMed, Embase, Web of Science, Scopus, CINAHL, PEDro, and the Cochrane Library. There was no year limit or language filter used in the search strategy. The specific details of the search strategy for each database are described in the S1 Table. Searches were conducted from the earliest records up to 30 May 2024. In addition, the reference lists of the identified studies were combed to find extra relevant research articles. The titles and abstracts of all the results retrieved in the searches were imported into Endnote 20 (Clarivate Analytics, London, UK) to remove duplicates. Articles that appeared potentially relevant based on their title were further reviewed by consulting their abstract. If an article was deemed eligible based on its abstract, the full text was immediately reviewed for further assessment. Two authors (BQ and ZW) conducted an independent search for published studies, and discrepancies were resolved through consultation with the third author (JDC).

### Eligibility criteria

We established the inclusion and exclusion criteria according to the PICOS strategy [59]. We selected studies with randomized and controlled experimental designs in which an experimental group of healthy women exposed to WBVT was compared to a) a non-exercise control group or b) an exercise control group. In either case, the outcomes of the intervention and control groups should have been tested via the pre-and post-intervention testing of at least one muscle performance variable. In evaluating muscle power, we opted for jumps that incorporated a countermovement (e.g., countermovement jump or vertical jump), and we excluded studies in which participants underwent acute WBVT (e.g., only one session of WBVT). Additionally, studies that included women with any disease or in a sub-healthy state (e.g., obesity, osteoporosis, etc.) were excluded, as these conditions may artificially enlarge the benefits of WBVT, particularly when compared to a non-exercise situation [60,61]. Lastly, study designs that involved the ingestion of substances or medicaments during the training interventions that could have affected physical function (e.g., creatine or caffeine) were excluded [62]. Systematic reviews, conference papers, dissertations, opinion articles, reviews, case reports, and editorials were also excluded. A detailed definition of the inclusion and exclusion criteria is shown in the S2 Table.

### Data extraction

Once the inclusion/exclusion criteria were applied, data extraction was performed based on the following items: (a) type of study design; (b) participants’ age and characteristics; (c) sample size for the experimental and control groups; (d) characteristics of the WBVT program; (e) vibrating platform parameter settings (amplitude, frequency, and acceleration); and (f) muscle performance outcomes, such as muscle strength and muscle power. The data extraction was done to assure the obtaining of the “Big Five” of WBVT: vibration amplitude, vibration frequency, method of application, session duration/frequency, and total intervention duration, following the guidelines of Oroszi et al [63]. Two authors were responsible for the data extraction procedure (BQ and ZW). When the data were presented as images, we used WebPlotDigitizer to enlarge the images for data extraction [64]. For studies that did not report the means and standard deviations of the muscle strength outcomes and met the inclusion criteria for this paper, we requested data from the corresponding author via email. When the two assessors disagreed with regard to data extraction, this was resolved via a joint discussion with a third author (JDC).

### Assessment of methodological quality

The methodological quality of the included studies was evaluated using the Physiotherapy Evidence Database (PEDro) scale, which has been shown to have good reliability and validity [65]. The PEDro scale has 11 possible points and examines the external validity (criterion 1) and internal validity (criteria 2–9) of controlled trials and whether there is sufficient statistical information to interpret the results (criteria 10–11). The studies were scored as excellent (score = 9–10), good (score = 6–8), fair (score = 4–5), and poor (score < 4). Two independent researchers (BQ and ZW) evaluated the quality of the included studies, and disagreements were resolved via discussion with the third author (JDC).

### Data analysis

We followed the Cochrane Handbook for Systematic Evaluation of Interventions for the processing and analysis of the data obtained from the included studies [66] and used Comprehensive Meta-Analysis software (version 3; Biostat, Englewood, NJ, USA) as the processing software for the data analysis in this review. The standardized mean differences (SMDs) and 95% confidence intervals (CIs) were calculated for each outcome that investigated the pooled effect of WBVT on muscle performance outcomes, using the mean and SD of the pre-and post-intervention changes observed in the experimental group (exposed to WBVT) and the control group (exposed to non-exercise or another type of exercise), as well as the sample sizes for each group. The SD of the pre-to-post-intervention differences was calculated using a formula described in a previous study [67]. All meta-analyses were conducted using a model with random effects, and each outcome required a minimum of three studies [68]. We utilized the following classification to categorize the magnitude of the SMD: very small (≤ 0.20), small (0.20–0.49), medium (0.50–0.79), and large (≥ 0.80), respectively [69]. The threshold for statistical significance was set at *p* < 0.050. We measured the degree of heterogeneity using the *I*^2^ statistic, with values < 50% indicating low heterogeneity, 50–75% indicating moderate heterogeneity, and > 75% indicating fairly high heterogeneity [70]. Finally, we used Egger’s linear regression tests to detect publication bias and used *p* < 0.10 as the threshold for significant publication bias [71].

In order to investigate the potential effects of the moderating variables, we performed subgroup analyses to explore the effects of WBVT on muscle strength and power depending on the participants’ menopausal status (pre-menopausal vs. post-menopausal), the type of muscle contraction tested (dynamic vs. isometric), the duration of the intervention period (< 12 weeks vs. ≥ 12 weeks), the amplitude of the vibration (> 3 mm vs. ≤ 3 mm) and the frequency of the vibration (> 30 Hz vs. ≤ 30 Hz); this was inspired by a previous publication [34]. Additionally, sensitivity analysis was conducted by removing studies of fair-to-poor quality for variables with at least three studies of good to excellent quality.

### Certainty of evidence

The certainty of evidence from  each meta-analysis was evaluated using the Grading of Recommendation, Assessment, Development, and Evaluation (GRADE) methodology [72]. The evidence was rated on a scale of very low (the true effect is likely to differ significantly from the estimated effect), low (the true effect may differ significantly from the estimated effect), moderate (the authors believe that the true effect is likely to be close to the estimated effect), or high (the authors have a high degree of confidence that the true effect is similar to the estimated effect). The GRADE assessment was conducted by two authors (BQ and ZW) and disputes were resolved through discussion.

## Results

### Study selection

**Fig 1:**
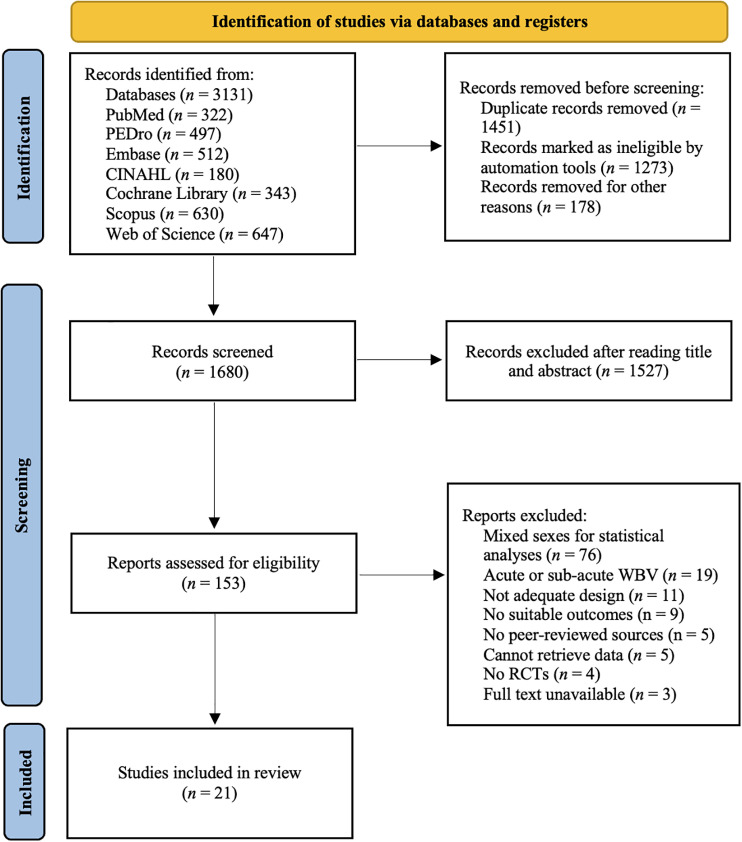
PRISMA flowchart of the study selection process.

A total of 3131 records was identified in the initial search, while 1680 records remained after removing the duplicates (Fig 1). Then, after screening for titles and abstracts, 1527 records were excluded, leaving 153 articles for the full-text review and the application of the inclusion and exclusion criteria. Finally, 21 studies were considered suitable for the systematic review and meta-analysis based on the characteristics of the study. All articles were considered to meet the methodological quality criteria and were retained in the systematic review.

### Methodological quality of the study

The outcomes of the methodological quality assessment are shown in the S3 Table. The included studies scored between 4 and 8 points, with a mean ± SD value of 5.10 ± 1.26 points and an overall article quality that ranged from fair to good. There were no studies that were judged to have either poor or excellent quality based on the PEDro criteria.

### The characteristics of included studies

The 21 studies [38–58] included in this study comprised a pooled number of 748 healthy women. The mean age of the participants ranged from 9.7 to 79.3 years. Of these studies, 11 trials included post-menopausal women, and 10 trials included young and pre-menopausal women. In terms of the study design with regard to the control group, 14 studies compared WBVT with blank/non-exercise control groups, 6 studies compared WBVT with exercise control groups that performed the same exercise training without WBVT, and only 1 study included both types of control groups. The mechanical vibration parameters reported in the included studies had a vibration amplitude that ranged from 1 to 6 mm, and the frequency of vibration ranged from 10 to 45 Hz. The duration of the interventions ranged between 3 and 48 weeks, with a frequency of 1–5 sessions per week. The basic characteristics of the included studies are shown in Table 1.

**Table 1:** Main characteristics of the participants and interventions of the studies included in the systematic review (*n* = 21).

Author(s) Year Country	Participants type N Age (years)	WBVT duration Sessions/Per Week	Exercises performed with WBVT	Amplitude (mm) Frequency (Hz) Acceleration (g)	Comparator	Outcomes
Dallas et al. 2019 Greece [41]	Young females WBVT: 12 CON: 10 9.7 ± 0.95	6 weeks 3/week	(a) Squat (b) One-legged squat	2.5 mm 30 Hz 2.28 g	CG B	CMJ
Delecluse et al. 2003 Belgium [38]	Young females WBVT: 18 Sham WBVT: 19 CON: 12 WBVT: 21.5 ± 2.1 Sham WBVT (CG B): 22.2 ± 1.4 CON (CG A): 20.6 ± 1.7	12 weeks 3/week	(a) Squat (b) Deep squat (c) Wide-stance squat (d) One-legged squat (e) Lunge	2.5 mm; 5 mm 35–40 Hz 2.28 g–5.09 g	CG A, B	(a) Maximal dynamic knee-extensor torque (b) Maximal isometric knee-extensor torque (c) CMJ
Eider et al. 2011 Poland [42]	Young women WBVT: 19 CON: 18 WBVT: 23.25 ± 0.67 CON: 20.87 ± 0.23	8 weeks 3/week	(a) Standing (b) One leg flexion-extension (c) Half-squat position and dynamic squat (d) Extend the foot at 165º and pull calibrated tubes back. (e) Push calibrated tubes forward. (f)Push-up on the platform (g) Abdominal isometric stress in horizontal stabilization.	2/3.5/5 mm 20 Hz NR	CG B	CMJ
Fagnani et al. 2006 Italy [43]	Young women WBVT: 13 CON: 11 WBVT: 24.0 ± 1.82 CON: 23.63 ± 1.91	8 weeks 3/week	(a) Squat (b) One-legged squat	4 mm 35 Hz 17 g	CG A	(a) Leg press (b) CMJ
Gerodimos et al. 2015 Italia [40]	Middle-aged women WBVT: 12 CON: 12 WBVT: 43.2 ± 3.0 CON: 44.8 ± 3.4	8 weeks 3/week	Maintained an upright position with their knees flexed at 10°	6 mm 20–25 Hz NR	CG A	(a) Knee extensors (b) Knee flexors (c) CMJ
Han et al. 2017 Korea [44]	Elderly women WBVT (VI): 12 WBVT (ET): 13 CON: 15 69.0 ± 4.0	8 weeks 3/week	(a) Two-leg calf raises (b) Half squat (c) Half squat and calf raise (d) One leg calf raise	VI:1.1–2.5 mm ET:1.1 mm VI:25–40 Hz ET:25–35 Hz NR	CG A	Ankle Muscle Force
Hartard et al. 2022 Germany [39]	Young women WBVT: 25 CON: 27 WBVT: 33.7 ± 6.8 CON: 35.0 ± 5.0	5 weeks 2/week	(a) Squatting (b) Calf raises (c) Squat jumps	2–3.5 mm 20 Hz NR	CG B	CMJ
Hawkey et al. 2016 United Kingdom [45]	Young and middle-aged women WBVT: 12 CON: 13 WBVT 1: 24.7 ± 2.6 CON 1: 21.0 ± 0.8 WBVT 2: 52.0 ± 4.4 CON 2: 49.5 ± 2.5	5 weeks 1/week	(a) Static squat (90°) (b) A lunge on each leg	4 mm 30–45 Hz NR	CG B	CMJ
Jaime et al. 2019 USA [46]	Elderly women WBVT:13 CON: 8 WBVT: 64 ± 1 CON: 67 ± 1	12weeks NR	a) Full squats b) High squats c) Wide squats d) Calf raises	NR 24-40 Hz NR	CG A	Leg press
Karatrantou et al. 2013 Greece [47]	Young females WBVT: 13 CON: 13 WBVT: 20.4 ± 0.4 CON: 20.5 ± 0.4	3 weeks NI	Upright position (knees flexed at 10°)	6 mm 25 Hz NR	CG A	(a) Knee extensors (b) Knee flexors (c) CMJ
Machado et al. 2010 Spain [48]	Elderly women WBVT: 13 CON: 13 WBVT: 79.3 ± 7.3 CON: 76.2 ± 8.4	10 weeks 3-5/week	(a) Half-squat (b) Deep squat (c) Wide stance (d) Squat calves	2-4 mm 20-40 Hz NR	CG A	Leg press
Marin-Cascales et al. 2015 Spain [49]	Elderly women WBVT: 14 CON: 10 WBVT: 60.1 ± 5.8 CON: 62.4 ± 5.1	12 weeks 3/week	Half-squat (knee and hip angle 120°) Ankle plantarflexion and dorsiflexion were performed	4 mm 35Hz NR	CG A	Knee extension
Marin-Cascales et al. 2017 Spain [50]	Elderly women WBVT: 15 CON: 10 60.0 ± 6.3	24 weeks 3/week	Half-squat (knee and hip angle 120°) Ankle plantarflexion and dorsiflexion were performed	4 mm 35-40 Hz NR	CG A	(a) Knee Extension (b)Ankle Plantar flexion
Mikami et al. 2019 Japan [51]	Young women WBVT: 14 CON: 12 WBVT: 24.1 ± 2.2 CON: 24.2 ± 2.1	12 weeks 3/week	(a) Single leg standing with eyes open (b) Squatting	4.5 mm 10 Hz NR	CG B	(a) Knee extension (b) Knee flexion
Oliveira et al. 2018 Brazil [52]	Elderly women WBVT: 17 CON: 15 WBVT: 56.3 ± 6.4 CON: 54.1 ± 5.2	24 weeks 3/week	Stand on the platform oscillation plate with knees semi flexed at 30°	4 mm 20 Hz 3.2g	CG A	(a) Knee extensors (b) Knee flexors
Roelants et al. 2004 Belgium [53]	Elderly women WBVT: 24 CON: 25 WBVT: 64.6 ± 3.43 CON: 64.2 ± 3.0	24 weeks 3/week	(a) High squat (b) Deep squat (c) Wide-stance squat (d) Lunge	2-5 mm 35-40 Hz 2.28g-5.09 g	CG A	(a) Maximal dynamic knee- extensor torque (b) Maximal isometric knee- extensor torque (c) CMJ
Shin et al. 2018 Korea [54]	Elderly women WBVT (LS): 13 WBVT: 13 CON: 11 WBVT (LS): 55.76 ± 3.98 WBVT: 57.23 ± 6.04 CON: 54.62 ± 6.41	12weeks 5/week	(a) Standing	1-3 mm 25-30 Hz NR	CG A	Knee extensor strength
Spiliopoulou et al. 2013 Greece [55]	Middle-aged women WBVT: 11 CON: 10 WBVT: 43.35 ± 4.12 CON: 42.31 ± 3.73	9 weeks 3/week	(a) Half squat (b) Wide-stance squat (c) One-legged half squat	2-12.8 cm 15-25 Hz 0.91-16.3 g	CG A	(a) Dynamic knee extensor/flexors (b) Isometric knee extensor/flexors (c) Ankle strength
Verschueren et al. 2004 Belgium [56]	Elderly women WBVT: 25 CON: 23 WBVT: 64.6 ± 3.3 CON: 64.2 ± 3.1	24 weeks 3/week	(a) Squat (b) Deep squat (c) Wide stance squat (d) One-legged squat (e) Lunge	1.7 mm; 2.5 mm 35-40 Hz 2.28g-5.09 g	CG A	(a) Isometric knee strength (b) Isotonic knee strength
Von Stengel et al. 2011 Germany [57]	Elderly women WBVT (VVT): 34 WBVT (RVT): 29 CG: 33 WBVT (VVT): 68.1 ± 4.0 WBVT (RVT): 67.9 ± 3.8 CG: 67.6 ± 4.1	48 weeks 3/week	(a) Two-legged squat (b) Two-legged dynamic squats (c) Leg abduction (d) One-legged squats (e) One-legged squat (f) Repetition of exercise	VVT: 1.7 mm 35 Hz RVT: 12 mm 12.5 Hz 8 g	CG A	(a) Leg press (b) CMJ
Xiong et al. 2023 China [58]	Elderly women WBVT: 21 CON: 23 WBVT: 64.7 ± 1.8 CON: 64.6 ± 2.0	16 weeks 3/week	(a) Half squats (b) Static weight-free squats (c) Left and right lunge squats	3 mm 45Hz NR	CG B	Dynamic knee extension

**Note:** EG, whole-body vibration training group; CG, control group; CG A, non-exercise control group; CG B, control group with the same exercise without WBVT; CMJ, countermovement jump; NR, not reported; VVT, Vertical Vibration Training; RVT, Rotational Vibration Training; LS, Load Stimulation; VI, Vibration Intensity; ET, Exposure Time.

### Meta-analysis of knee strength

#### Effects of WBVT compared with non-exercise control groups on knee extension.

**Fig 2:**
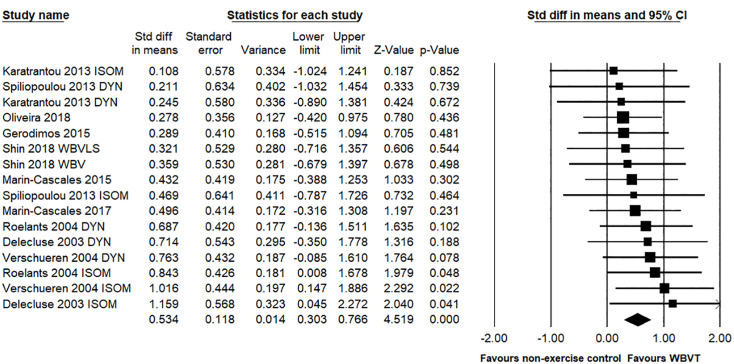
Effect of the WBVT group compared with non-exercise control groups on the knee extension strength. The black diamond at the bottom of the graph represents the pooled standardized mean difference following random effects meta-analyses.

In terms of knee extension strength, 10 studies with a total of 16 pairwise comparisons tested the efficacy of WBVT compared to a non-exercise control group, with a total of 309 participants. The results showed that the knee extension strength gains were higher with WBVT than with the non-exercise comparator (SMD = 0.534, 95% CI: 0.303 to 0.766, *p* < 0.001, magnitude = medium; Fig 2); this showed low heterogeneity (*I*^2^ = 0%, *p* = 0.984). Egger’s linear regression test did not reveal significant publication bias (*p* = 0.809).

#### Subgroup analysis of WBVT compared with non-exercise control groups on knee extension.

The subgroup analyses for WBVT compared with the non-exercise control groups with regard to knee extension showed that WBVT improved the strength of both isometric (SMD = 0.659, *p* = 0.001, magnitude = medium) and dynamic muscle contractions (SMD = 0.462, *p* = 0.002, magnitude = small); this was significant in both post-menopausal (SMD = 0.576, *p* < 0.001, magnitude = medium) and pre-menopausal women (SMD = 0.448, *p* = 0.030, magnitude = small), and in both whole-body vibration training with an amplitude > 3 mm (SMD = 0.331, *p* = 0.047, magnitude = small) and with ≤ 3 mm (SMD = 0.664, *p* = 0.005, magnitude = medium). However, the subgroup analysis of the mechanical vibration frequency of > 30 Hz was superior to the non-exercise control group (SMD = 0.736, *p* < 0.001, magnitude = medium), while the effect of WBVT with ≤ 30 Hz did not reach statistical significance (SMD = 0.284, *p* = 0.109, magnitude = small). Similarly, the studies with a training duration of ≥ 12 weeks were superior to the non-exercise control group (SMD = 0.618, *p* < 0.001, magnitude = medium); meanwhile, the effect of WBVT for < 12 weeks did not reach statistical significance (SMD = 0.264, *p* = 0.279, magnitude = small; Table 2).

**Table 2:** Subgroup analysis of whole-body vibration training compared to non-exercise controls on knee extension strength.

Subgroups	*n*	SMD	95% CI	*p*	*I* ^2^	Magnitude
**Strength outcome**						
Isometric	7	0.659	0.276-1.041	0.001	0%	Medium
Dynamic	9	0.462	0.171-0.754	0.002	0%	Small
**Menopausal status**						
Pre-menopausal	7	0.448	0.042-0.854	0.030	0%	Small
Post-menopausal	9	0.576	0.294-0.859	<0.001	0%	Medium
**Intervention Duration**						
< 12 weeks	5	0.264	−0.214-0.741	0.279	0%	Small
≥ 12 weeks	11	0.618	0.353-0.883	<0.001	0%	Medium
**Amplitude**						
> 3 mm	8	0.331	0.005-0.656	0.047	0%	Small
≤ 3 mm	4	0.664	0.197-1.132	0.005	0%	Medium
**Frequency**						
> 30 Hz	8	0.736	0.424-1.047	<0.001	0%	Medium
≤ 30 Hz	8	0.284	−0.063-0.631	0.109	0%	Small

SMD, standardized mean difference; CI, confidence intervals; *I*^2^, a measure of heterogeneity between studies expressed as a percentage; *p*, Significance level of pooled standardized mean difference; *n*, number of trials.

#### Effects of WBVT compared with exercise control groups on knee extension.

**Fig 3:**
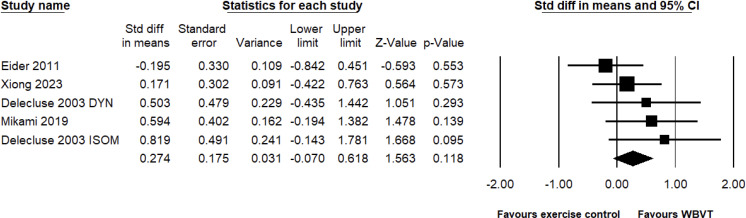
Effect of the WBVT group compared with the exercise control groups on the knee extension strength. The black diamond at the bottom of the graph represents the pooled standardized mean difference following random effects meta-analyses.

A total of five pairwise comparisons were conducted in four studies that tested the efficacy of WBVT compared to exercise control groups, with a total of 143 participants. The results showed that the improvements in the knee extension strength observed with WBVT were not significantly different from those observed in the exercise control groups (SMD = 0.274, 95% CI: −0.070 to 0.618, *p* = 0.118, magnitude = small; Fig 3); meanwhile, heterogeneity was low (*I*^2^ = 6%, *p* = 0.375). Egger’s linear regression test did not reveal significant publication bias (*p* = 0.112).

#### Effects of WBVT compared with non-exercise groups on knee flexion.

**Fig 4:**
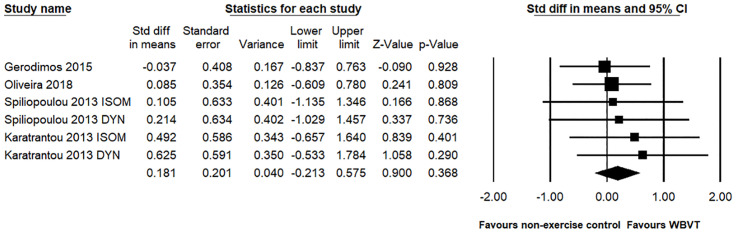
Effect of the WBVT group compared with the non-exercise control groups on the knee flexion strength. The black diamond at the bottom of the graph represents the pooled standardized mean difference following random effects meta-analyses.

In terms of knee flexion strength, a total of four studies and six pairwise comparisons were conducted to test WBVT compared to non-exercise control groups, with a total of 126 participants. The meta-analysis showed that the improvement in the knee flexion muscle strength observed with WBVT was not significantly different from that observed in the non-exercise control groups (SMD = 0.181, 95% CI = −0.213 to 0.575, *p* = 0.368, magnitude = small; Fig 4), with a low level of heterogeneity (*I*^2^ = 0%, *p* = 0.943). Egger’s linear regression test did not reveal significant publication bias (*p* = 0.175). Because two studies used an exercise control group to examine the effect of WBVT on knee flexion strength, we did not perform a meta-analysis of the WBVT group that compared it with the exercise control groups.

### Meta-analysis of leg strength

**Fig 5:**
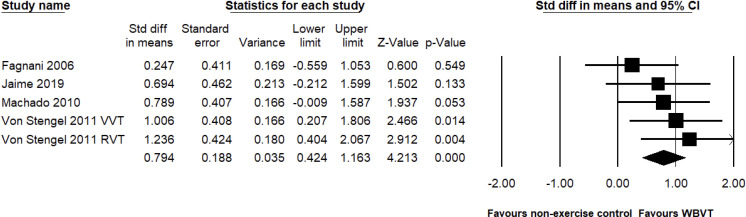
Effect of the WBVT group compared with the non-exercise control groups on leg press. The black diamond at the bottom of the graph represents the pooled standardized mean difference following random effects meta-analyses.

For the leg press outcomes, a total of four studies with six paired comparisons examined the leg press strength gains observed with WBVT compared to the non-exercise control groups, with a total of 150 participants. The meta-analysis showed that the improvements in the leg strength observed with WBVT were greater than those observed in the control group (SMD = 0.794, 95% CI = 0.424 to 1.163, *p* < 0.001, magnitude = medium; Fig 5), with a low level of heterogeneity (*I*^2^ = 0%, *p* = 0.529). Egger’s linear regression test did not detect significant publication bias (*p* = 0.967). No study used an exercise control group to test the effect of WBVT.

### Meta-analysis of ankle strength

**Fig 6:**
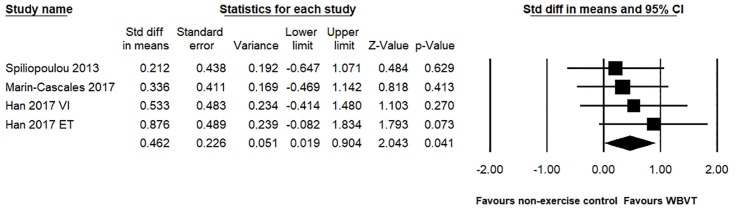
Effect of the WBVT group compared with the non-exercise control groups on the ankle plantar flexion strength. The black diamond at the bottom of the graph represents the pooled standardized mean difference following random effects meta-analyses.

For the ankle strength outcomes, a total of four studies investigated the effects of WBVT compared to a non-exercise control group. We found that WBVT greatly improved the ankle plantar flexion strength compared to the non-exercise control group (SMD = 0.462, 95% CI: 0.019 to 0.904, *p* = 0.041, magnitude = small; Fig 6), with low heterogeneity (*I*^2^ = 0%, *p* = 0.763). Due to insufficient data regarding ankle inversion, ankle eversion and ankle dorsiflexion, we did not perform this meta-analysis.

### Meta-analysis of CMJ

#### Effects of WBVT compared with non-exercise control groups on CMJ.

**Fig 7:**
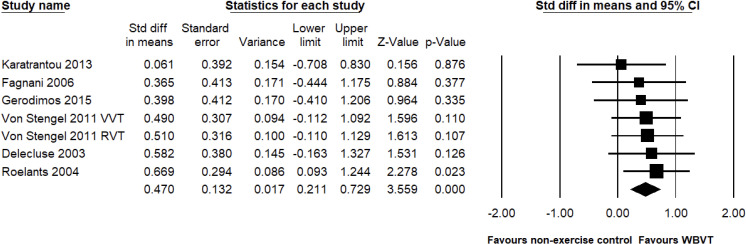
Effect of the WBVT group compared with the non-exercise control groups on countermovement jump. The black diamond at the bottom of the graph represents the pooled standardized mean difference following random effects meta-analyses.

In terms of the countermovement jump performance, six studies with a total of seven pairwise comparisons tested the efficacy of WBVT compared to the non-exercise control groups, with a total of 248 participants. The results showed significantly greater improvements in the countermovement jump performance with WBVT compared to the non-exercise control group (SMD = 0.470, 95% CI: 0.211 to 0.729, *p* < 0.001, magnitude = small; Fig 7), with low heterogeneity (*I*^2^ = 0%, *p* = 0.941). Egger’s linear regression test did not reveal significant publication bias (*p* = 0.120).

#### Subgroup analyses of WBVT compared with non-exercise control groups on CMJ.

In Table 3, subgroup analyses showed that WBVT was superior with regard to improvements in the countermovement jump performance compared to the non-exercise control group, with < 12 weeks (SMD = 0.267, *p* = 0.254, magnitude = small) or ≥ 12 weeks interventions (SMD = 0.564, *p* < 0.001, magnitude = medium); moreover, the effect of WBVT was greater than the non-exercise control group in post-menopausal women (SMD = 0.561, *p* = 0.001, magnitude = medium) but not in pre-menopausal women (SMD = 0.354, *p* = 0.076, magnitude = small).

**Table 3:** Subgroup analysis of whole-body vibration training compared to non-exercise controls in countermovement jump performance.

Subgroups	*n*	SMD	95% CI	*p*	*I* ^2^	Magnitude
**Menopausal status**						
Pre-menopausal	4	0.354	−0.037-0.745	0.076	0%	Small
Post-menopausal	3	0.561	0.215-0.906	0.001	0%	Medium
**Intervention Duration**						
< 12 weeks	3	0.267	−0.192-0.726	0.254	0%	Small
≥ 12 weeks	4	0.564	0.251-0.878	<0.001	0%	Medium

SMD, standardized mean difference; CI, confidence intervals; *I*^2^, a measure of heterogeneity between studies expressed as a percentage; *p*, Significance level of pooled standardized mean difference; *n*, number of trials.

#### Effects of WBVT compared with exercise control groups on CMJ.

**Fig 8:**
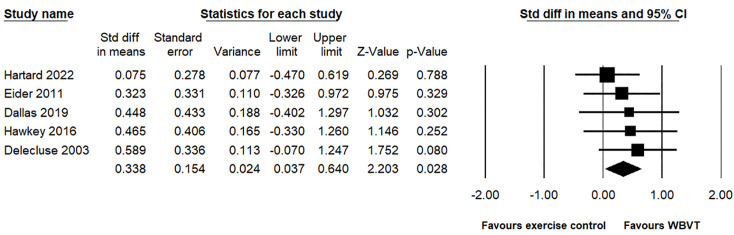
Effect of the WBVT group compared with exercise control groups on countermovement jump. The black diamond at the bottom of the graph represents the pooled standardized mean difference following random effects meta-analyses.

A total of six studies and seven pairwise comparisons were conducted to test the WBVT group against the exercise control group, with a total of 173 participants. The results showed that WBVT improved the countermovement jump performance to a greater extent than the exercise control group (SMD = 0.338, 95% CI: 0.037 to 0.640, *p* = 0.028, magnitude = small; Fig 8), with low heterogeneity (*I*^2^ = 0%, *p* = 0.805). Egger’s linear regression test did not reveal significant publication bias (*p* = 0.194).

### Sensitivity analysis

As shown in S4 Fig, the sensitivity analysis showed that WBVT compared to a non-exercise control group remained statistically significant (*p* = 0.022) for knee extension strength. Additionally, WBVT compared to an exercise control group achieved statistical significance for knee extension strength (*p* = 0.028).

### GRADE assessment

The GRADE assessment of the certainty of the evidence is presented in S5 Table. All analyses were rated as very low to low quality of evidence, except for the analyses comparing the WBVT to the non-exercise control group in knee extension strength and leg press strength and countermovement jump, which were rated as moderate.

## Discussion

To the best of our knowledge, this is the first systematic review to summarize investigations focused on the effect of whole-body vibration training on muscle strength outcomes in women by considering the characteristics of the comparator (non-exercise and exercise control groups). Our systematic review included 21 RCTs with 748 healthy female participants, and the main findings were as follows: (1) whole-body vibration training induced greater improvements in the knee extension, leg press, and ankle plantar flexion strength and countermovement jump performance in healthy women compared to non-exercise; (2) in comparison to exercise control groups, WBVT manifested superiority solely in augmenting countermovement jump performance; (3) subgroup analyses found that longer periods (≥ 12 weeks) of WBVT resulted in greater benefits for both muscle strength and power compared to shorter training periods. Additionally, higher vibration frequencies (> 30 Hz) provided greater benefits for improving muscle strength than lower frequencies, and post-menopausal women reaped greater benefits in muscle power than pre-menopausal women. These findings underscore that WBVT can be used as a means of improving muscle strength in healthy women and may be more effective than traditional strength exercises only on CMJ performance.

### Muscle strength

#### Knee strength.

Our meta-analysis showed that WBVT had a significantly greater effect on the knee extension strength of women compared to the non-exercise control group; however, this effect was not statistically significant compared to that in the exercise control group. In addition, we found no significant improvements in the knee flexion strength with whole-body vibration training with respect to the non-exercise control group, which may be related to the characteristics of the whole-body vibration training used for studies that test the knee flexion strength. The lack of significant improvement in knee flexion strength observed in this study aligns with findings from a previous study, which reported more pronounced effects of WBVT on knee extensor muscles compared to knee flexor muscles [60]. This discrepancy could be attributed to inherent differences in the biomechanical loading patterns, or the muscle activation strategies required during WBVT. Additionally, the specific vibration parameters used in the intervention may have had a limited impact on the flexors. Further research is needed to elucidate the underlying mechanisms and optimize WBVT protocols for different muscle groups. In the subgroup analyses of studies that have tested the knee extension strength, we found that higher amplitudes (> 3 mm) and lower frequencies (≤ 30 Hz) resulted in smaller effect sizes than lower amplitudes and higher frequencies. However, all of the literature included when testing the knee flexion strength used higher amplitudes (> 3 mm) and lower frequencies (≤ 30 Hz); this suggests that the minimal effect that WBVT had with regard to this variable may be associated with the amplitude and frequency settings of the whole-body vibration employed in these studies. As reported in previous studies, frequencies of ≤ 30 Hz are generally less beneficial to improving muscle strength [73,74], as these frequencies may not be sufficient to elicit adequate stimulation [75]. Finally, we found that women receiving longer periods (≥ 12 weeks) and higher vibration frequencies (> 30 Hz) of WBVT may obtain greater benefits in knee extension strength.

Regarding muscle contraction patterns, isometric exercises showed a larger effect size in response to WBVT compared to dynamic exercises, especially in knee extension tasks. This suggests that the static nature of isometric tasks, which require sustained muscle tension and joint stability, may allow for more efficient adaptation to the repetitive mechanical oscillation’s characteristic of WBVT. However, the extent to which WBVT differentially impacts neuromuscular activation in isometric versus dynamic exercises remains unclear. The potential factor contributing to these differences could be the biarticular nature of the rectus femoris may lead to different responses to WBVT between isometric tasks, which emphasize joint stability, and dynamic tasks, which require greater joint movement and coordination across multiple muscle groups.

#### Leg press strength.

For leg press strength, we found that WBVT significantly improved the leg press strength compared to the non-exercise control group. Due to the limited availability of data, we were unable to implement subgroup analyses of the effects of WBVT on the leg press strength of women compared to the non-exercising controls. For the same reason, we were unable to explore the effects of WBVT on the leg press strength of women compared with an exercising control group. Future studies should strengthen the investigation of WBVT with regard to the leg press strength of women.

#### Ankle strength.

For ankle muscle strength, we found that the whole-body vibration training resulted in significant increases in strength in the ankle plantar flexors. A recent meta-analysis [25] produced similar results to ours, finding significant increases in strength only in the ankle plantar flexors with regard to ankle-related outcomes. Until more investigations are performed to test the effect of WBVT in other forms of ankle strength testing, we can only suggest that WBVT is able to enhance ankle plantar flexion strength.

### Muscle power

#### CMJ performance.

For CMJ performance, we found that the countermovement jump performance was improved to a greater extent in the whole-body vibration training group than in the non-exercise control group and the exercise control group. Whole-body vibration training triggers a physiological response known as the “tonic vibration reflex” by stimulating sensory receptors and afferent pathways. This reflex is thought to increase the facilitation of the reflex action in the myoclonic reflex and the motor neuron pool [76]. These effects are particularly pronounced in muscle groups actively engaged during WBVT, suggesting that vibration training may optimize neural drive to muscles and improve force production efficiency. Therefore, considering the role of the stretch reflex and sensory fiber afferents in the stretch-shortening contraction of jumps (especially for countermovement jumps), vibration training may lead to more effective utilization of the stretch reflex in countermovement actions [40]. Besides, Piezo1 and Piezo2 are mechanosensitive ion channels in human tissues, triggering mechano-biomodulation. This may induce the production of hormonal and non-hormonal molecules, affecting physiological responses like proprioception, bone mineral density, metabolism, immune systems, and homeostasis [77]. Additionally, post-menopausal women obtained somewhat greater benefits than pre-menopausal women with regard to CMJ performance. As discussed previously [78], it can be observed that whole-body vibration interventions have a greater effect on participants with lower baseline values regarding power or other physiological variables [79]. Declines in muscle, bone, and joint function are related to age, so older women may have had less muscle power at baseline; this may account for the greater effect that WBVT had on post-menopausal women. Also, we found that women undergoing longer periods (≥ 12 weeks) of WBVT may induce greater benefits in countermovement jump performance with respect to shorter WBVT periods.

### Strengths and limitations

One of the study strengths is that we only included studies that investigated healthy women participants who have experienced long-term WBVT, which could avoid some artificial influence on the efficacy of WBVT when applied after a unique training session. In addition, we compared the effects of the WBVT with a) a non-exercise control group or b) an exercise control group on muscle strength and power, which allows us to understand the benefits of WBVT per se but also in comparison to other forms of training. However, this study also has some limitations. The analysis of the efficacy of the different vibration types and accelerations used for the WBVT could not be conducted due to the limited number of studies reporting specific mechanical vibration parameters. Additionally, there was some heterogeneity in the age of the participants included in the study. Although we made a subgroup analysis considering participants’ menopausal status, we were unable to perform subgroup analyses to compare, e.g., young and adult women. Consequently, the application of the outcomes of this study should be performed cautiously, specifically when applied to young women. In the current study, *n* = 14 (66%) of the studies were rated as fair-to-poor quality. A sensitivity analysis excluding these studies revealed that the effect of WBVT compared to the exercise control group on knee extension reached statistical significance. This finding suggests that the effect of WBVT may have been underestimated in some variables due to bias introduced by lower-quality studies. These results underscore the need for conducting more studies of good-to-excellent quality to better understand the true effects of WBVT. To enhance the quality of future research, the guidelines by van Heuvelen et al. [80] provide a robust framework for designing well-controlled randomized controlled trials (RCTs) in WBVT studies on humans. Finally, it was not possible to investigate the effects of this training method on upper body strength and power because there was a limited number of studies available. Future studies should expand the investigation of upper body strength to determine whether WBVT is equally able to improve upper body muscle strength and power in healthy women.

## Conclusions

In conclusion, WBVT has demonstrated its efficacy in improving muscle strength and power in healthy women compared to non-exercise control groups. Overall, WBVT was beneficial for enhancing knee extension, leg press, ankle plantar flexion, and countermovement jump. The potential benefits of WBVT were only associated with an enhancement in jump performance compared to other exercise interventions. Longer periods (≥ 12 weeks) of WBVT resulted in greater benefits for both muscle strength and power compared to the non-exercise control groups, higher vibration frequencies (> 30 Hz) provided greater improvements in muscle strength, and post-menopausal women reaped greater benefits in muscle power with WBVT than pre-menopausal women. To strengthen the current findings, further studies are necessary, particularly in young women and in studies that use upper body WBVT.

## Supporting information

S1 Table Complete search strategy. (DOCX)

S2 Table PICOS criteria. (DOCX)

S3 Table Methodological quality of included study. (DOCX)

S4 Fig Sensitivity analysis result. (DOCX)

S5 Table GRADE assessment of the certainty of the evidence. (DOCX)

S6 Table PRISMA 2020 checklist. (DOCX)

S7 Table List of screened studies. (DOCX)
