# Supplementary material for: Effects of whole-body vibration training on muscle performance in healthy women: A systematic review and meta-analysis of randomized controlled trials
Source: PLoS One. 2025 May 30;20(5):e0322010. doi: 10.1371/journal.pone.0322010 (PMC12124539; doi:10.1371/journal.pone.0322010)
Supplement: S1 Table — (DOCX) [file pone.0322010.s001.docx]

Strategies for Searching the Seven Databases Used in the Investigation

**S1 Table 1.** PubMed search strategy.

| 1 | “Female” [Mesh] | 9,776,378 |
| --- | --- | --- |
| 2 | Females [Title/Abstract] | 460,406 |
| 3 | “Women” [Mesh] | 45,911 |
| 4 | (Girls [Title/Abstract]) OR (Girl [Title/Abstract]) OR (Woman [Title/Abstract]) OR (Women’s Groups [Title/Abstract]) OR (Women Groups [Title/Abstract]) OR (Women’s Group [Title/Abstract]) | 449,705 |
| 5 | 1 OR 2 | 9,867,998 |
| 6 | 3 OR 4 | 490,991 |
| 7 | 5 OR 6 | 9,954,702 |
| 8 | (strength [Title/Abstract]) OR (power [Title/Abstract]) | 816,661 |
| 9 | (Whole body vibration training [Title/Abstract]) OR (Whole body vibration exercise [Title/Abstract]) OR (Whole-body vibration [Title/Abstract]) OR (WBV[Title/Abstract]) OR (whole body vibrations [Title/Abstract]) OR (wholebody vibration [Title/Abstract]) OR (whole body vibration [Title/Abstract]) | 2,977 |
| 10 | 7 AND 9 | 1,116 |
| 11 | 8 AND 10 | 322 |

**S1 Table 2.** Embase search strategy.

| S1 | ‘female’/exp | 12,534,891 |
| --- | --- | --- |
| S2 | females: ab,ti OR woman: ab,ti OR women: ab,ti | 2,653,063 |
| S3 | S1 OR S2 | 12,836,052 |
| S4 | ‘whole body vibration’/exp | 2,015 |
| S5 | 'whole body vibration training':ab,ti OR 'whole body vibration exercise':ab,ti OR 'whole-body vibration':ab,ti OR wbv:ab,ti OR 'whole body vibrations':ab,ti OR 'wholebody vibration':ab,ti | 3,745 |
| S6 | S4 OR S5 | 4,075 |
| S7 | S3 AND S6 | 1,830 |
| S8 | strength: ab,ti OR power: ab,ti | 933,432 |
| S9 | S7 AND S8 | 512 |

**S1 Table 3.** Web of Science search strategy.

| #1 | TS = (Female OR Females OR Women OR Girls OR Girl OR Woman OR Women’s Groups OR Women Groups OR Women’s Group) | 3,246,348 |
| --- | --- | --- |
| #2 | TS = (strength OR power) | 4,432,297 |
| #3 | TS = (Whole body vibration training OR Whole body vibration exercise OR Whole-body vibration OR WBV OR whole body vibrations OR wholebody vibration OR whole body vibration) | 6,284 |
| #4 | #1 AND #2 AND #3 | 647 |

**S1 Table 4.** Cochrane Library search strategy.

| #1 | MeSH descriptor: [Women] explode all trees | 1,489 |
| --- | --- | --- |
| #2 | (Women Groups OR Women’s Groups OR Women’s Group OR Girls OR Girl OR Woman):ti,ab,kw | 202,087 |
| #3 | #1 OR #2 | 202,087 |
| #4 | MeSH descriptor: [Female] in all MeSH products | 603,955 |
| #5 | (Females):ti.ab.kw | 38,526 |
| #6 | #4 OR #5 | 627,404 |
| #7 | (Whole body vibration training OR Whole body vibration exercise OR Whole-body vibration OR WBV OR whole body vibrations OR wholebody vibration OR whole body vibration):ti,ab,kw | 1,644 |
| #8 | (strength OR power):ti,ab,kw | 94,049 |
| #9 | #3 OR #6 | 727,805 |
| #10 | #7 AND #9 | 656 |
| #11 | #8 AND #10 | 343 |

| (TITLE-ABS-KEY (Whole body vibration training) OR TITLE-ABS-KEY (Whole body vibration exercise) OR TITLE-ABS-KEY (Whole-body vibration) OR TITLE-ABS-KEY (WBV) OR TITLE-ABS-KEY (whole body vibrations)) AND (TITLE-ABS-KEY (women) OR TITLE-ABS-KEY (female)) AND (TITLE-ABS-KEY (strength) OR TITLE-ABS-KEY (power)) | 630 |
| --- | --- |

**S1 Table 5.** Scopus search strategy.

**S1 Table 6.** CINAHL search strategy.

| S1 | AB female OR women OR women OR females | 2,448,501 |
| --- | --- | --- |
| S2 | AB strength OR power | 221,890 |
| S3 | AB Whole body vibration training OR Whole body vibration exercise OR Whole-body vibration OR WBV OR whole body vibrations OR wholebody vibration OR whole body vibration | 1,338 |
| S4 | S1 AND S2 AND S3 | 180 |

| Whole body vibration | 497 |
| --- | --- |

**S1 Table 7.** PEDro search strategy.
