# Supplementary material for: Effects of whole-body vibration training on muscle performance in healthy women: A systematic review and meta-analysis of randomized controlled trials
Source: PLoS One. 2025 May 30;20(5):e0322010. doi: 10.1371/journal.pone.0322010 (PMC12124539; doi:10.1371/journal.pone.0322010)
Supplement: S2 Table — (DOCX) [file pone.0322010.s002.docx]

**Table 1.** PICOS criteria for the inclusion/exclusion of randomized controlled trials.

| Parameters | Inclusion | Exclusion | Extraction |
| --- | --- | --- | --- |
| P (Participants) | Healthy women | Women with medical conditions | Number of participants, age, gender, health status, and anthropometric characteristics |
| I (Interventions) | An intervention with WBVT | Other strength training protocols | Duration and frequency of the training program, and characteristics of WBVT |
| C (Comparisons) | Non-exercise control groups or exercise control groups | Other types of control groups with characteristics non-comparable to the intervention group | Characteristics of the control intervention, if any |
| O (Outcomes) | Muscle Performance | Other outcomes not associated with muscle performance | Knee flexion strength, leg strength, ankle flexion/extension strength and jump height |
| S (Study design) | Randomized controlled trials | Systematic reviews, dissertations, conference papers, unpublished research | Experimental design, date of publication |
