# Supplementary material for: Effects of whole-body vibration training on muscle performance in healthy women: A systematic review and meta-analysis of randomized controlled trials
Source: PLoS One. 2025 May 30;20(5):e0322010. doi: 10.1371/journal.pone.0322010 (PMC12124539; doi:10.1371/journal.pone.0322010)
Supplement: S3 Table — (DOCX) [file pone.0322010.s003.docx]

**Table 1.** Assessment of the methodological quality of eligible studies (*n* = 21) using the Physiotherapy Evidence Database (PEDro) scale.

|  | Assessment Criteria | | | | | | | | | | | |  | |  | |
| --- | --- | --- | --- | --- | --- | --- | --- | --- | --- | --- | --- | --- | --- | --- | --- | --- |
| Study | 1 | 2 | 3 | 4 | 5 | 6 | 7 | 8 | 9 | 10 | 11 | Total Score | | Quality Assessment | |  |
| Dallas et al., 2019 | Y | 1 | 0 | 1 | 0 | 0 | 0 | 0 | 0 | 1 | 1 | 4 | | Fair | |  |
| Delecluse et al., 2003 | Y | 1 | 0 | 1 | 0 | 0 | 0 | 1 | 1 | 1 | 1 | 6 | | Good | |  |
| Eider et al., 2011 | Y | 1 | 0 | 1 | 0 | 0 | 0 | 0 | 0 | 1 | 1 | 4 | | Fair | |  |
| Fagnani et al., 2006 | Y | 1 | 0 | 1 | 0 | 0 | 0 | 1 | 0 | 1 | 1 | 5 | | Fair | |  |
| Gerodimos et al., 2015 | Y | 1 | 0 | 1 | 0 | 0 | 0 | 1 | 0 | 1 | 1 | 5 | | Fair | |  |
| Han et al., 2017 | Y | 1 | 0 | 1 | 0 | 0 | 0 | 0 | 0 | 1 | 1 | 4 | | Fair | |  |
| Hartard et al., 2022 | Y | 1 | 0 | 1 | 0 | 0 | 0 | 0 | 0 | 1 | 1 | 4 | | Fair | |  |
| Hawkey et al., 2016 | Y | 1 | 0 | 1 | 0 | 0 | 0 | 1 | 0 | 1 | 1 | 5 | | Fair | |  |
| Jaime et al., 2019 | Y | 1 | 0 | 1 | 0 | 1 | 0 | 1 | 0 | 1 | 1 | 6 | | Good | |  |
| Karatrantou et al.,2013 | Y | 1 | 0 | 1 | 0 | 0 | 0 | 0 | 0 | 1 | 1 | 4 | | Fair | |  |
| Machado et al., 2010 | Y | 1 | 0 | 1 | 0 | 0 | 1 | 1 | 0 | 1 | 1 | 6 | | Good | |  |
| Marin-Cascales et al., 2015 | Y | 1 | 0 | 1 | 0 | 0 | 0 | 0 | 0 | 1 | 1 | 4 | | Fair | |  |
| Marin-Cascales et al., 2017 | Y | 1 | 1 | 1 | 0 | 0 | 0 | 0 | 0 | 1 | 1 | 5 | | Fair | |  |
| Mikami et al., 2019 | Y | 1 | 1 | 1 | 1 | 1 | 0 | 1 | 0 | 1 | 1 | 8 | | Good | |  |
| Oliveira et al., 2018 | Y | 1 | 1 | 1 | 1 | 0 | 0 | 1 | 1 | 1 | 1 | 8 | | Good | |  |
| Roelants et al., 2004 | Y | 1 | 0 | 1 | 0 | 0 | 0 | 0 | 0 | 1 | 1 | 4 | | Fair | |  |
| Shin et al., 2018 | Y | 1 | 1 | 1 | 0 | 0 | 0 | 1 | 0 | 1 | 1 | 6 | | Good | |  |
| Spiliopoulou et al., 2013 | Y | 1 | 0 | 1 | 0 | 0 | 0 | 0 | 0 | 1 | 1 | 4 | | Fair | |  |
| Verschueren et al., 2004 | Y | 1 | 0 | 1 | 0 | 0 | 0 | 0 | 0 | 1 | 1 | 4 | | Fair | |  |
| Von Stengel et al., 2011 | Y | 1 | 0 | 1 | 0 | 0 | 0 | 1 | 0 | 1 | 1 | 5 | | Fair | |  |
| Xiong et al., 2023 | Y | 1 | 0 | 1 | 0 | 0 | 0 | 1 | 0 | 1 | 1 | 6 | | Fair | |  |

**Note:** 1 = eligibility criteria; 2 = random allocation; 3 = concealed allocation; 4 = baseline comparability; 5 = blind subjects; 6 = blind therapists; 7 = blind assessors; 8 = adequate follow-up; 9 = intention-to-treat analysis; 10 = between-group comparisons; 11 = point estimates and variability; Y = yes. The total score represents the score of the PEDro scale. Item 1 was not scored.
