# Supplementary material for: Effects of whole-body vibration training on muscle performance in healthy women: A systematic review and meta-analysis of randomized controlled trials
Source: PLoS One. 2025 May 30;20(5):e0322010. doi: 10.1371/journal.pone.0322010 (PMC12124539; doi:10.1371/journal.pone.0322010)
Supplement: S4 Fig — (DOCX) [file pone.0322010.s004.docx]

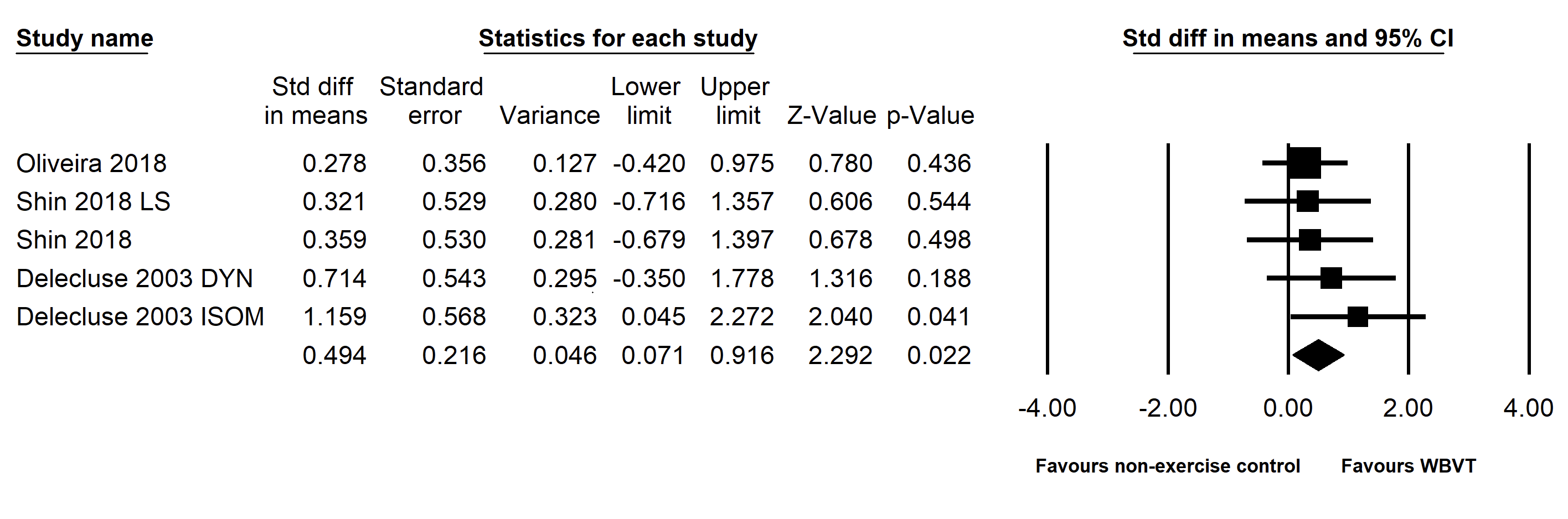


**Figure 1:** Sensitivity analysis of WBVT compared to non-exercise control groups for knee extensor strength.


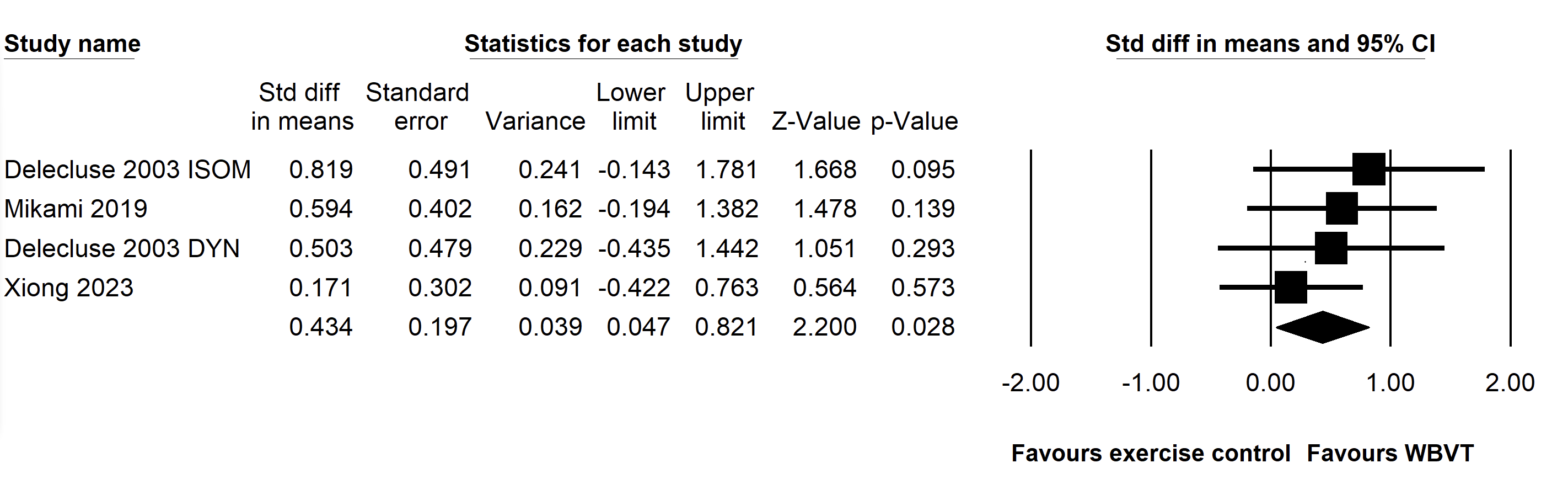


**Figure 2:** Sensitivity analysis of WBVT compared to exercise control groups for knee extensor strength.
