# Supplementary material for: Effects of whole-body vibration training on muscle performance in healthy women: A systematic review and meta-analysis of randomized controlled trials
Source: PLoS One. 2025 May 30;20(5):e0322010. doi: 10.1371/journal.pone.0322010 (PMC12124539; doi:10.1371/journal.pone.0322010)
Supplement: S5 Table — (DOCX) [file pone.0322010.s005.docx]

**Table S5.** Grades of Recommendation, Assessment, Development and Evaluation (GRADE) quality of evidence.

| Outcome | Design | Risk of bias | Indirectness | Inconsistency | Imprecision | Publication bias | Effect size | GRADE quality |
| --- | --- | --- | --- | --- | --- | --- | --- | --- |
| Knee extension strength ^a^  *I*^2^ = 0%, SMD = 0.534, 95% CI = 0.303 to 0.766 | 10 RCTs | – 1 | 0 | 0 | 0 | 0 | 0 | ⨁⨁⨁◯  Moderate |
| Knee extension strength ^b^  *I*^2^ = 6%, SMD = 0.274, 95% CI = -0.070 to 0.618 | 4 RCTs | – 1 | 0 | 0 | – 2 | 0 | 0 | ⨁◯◯◯  Very low |
| Knee flexion strength ^a^  *I*^2^ = 0%, SMD = 0.181, 95% CI = -0.213 to 0.575 | 4 RCTs | – 1 | 0 | 0 | – 2 | 0 | 0 | ⨁◯◯◯  Very low |
| Leg press strength ^a^  *I*^2^ = 0%, SMD = 0.794, 95% CI = 0.424 to 1.163 | 4 RCTs | – 1 | 0 | 0 | 0 | 0 | 0 | ⨁⨁⨁◯  Moderate |
| Ankle plantar flexion ^a^  *I*^2^ = 0%, SMD = 0.462, 95% CI = 0.019 to 0.904 | 3 RCTs | – 2 | 0 | 0 | – 1 | 0 | 0 | ⨁◯◯◯  Very low |
| Countermovement jump ^a^  *I*^2^ = 0%, SMD = 0.470, 95% CI = 0.211 to 0.729 | 6 RCTs | – 1 | 0 | 0 | 0 | 0 | 0 | ⨁⨁⨁◯  Moderate |
| Countermovement jump ^b^  *I*^2^ = 0%, SMD = 0.338, 95% CI = 0.037 to 0.640 | 5 RCTs | – 2 | 0 | 0 | 0 | 0 | 0 | ⨁⨁◯◯  Low |

RCT = randomized controlled trial, SMD = standardized mean difference, CI = confidence interval, *I*^2^ = inconsistency statistic, PEDro = Physiotherapy Evidence Database scale. Risk of bias was estimated using the Physiotherapy Evidence Database (PEDro) Scale. Risk of bias: Downgraded one place as most of the trials scored ≤ 6 on the PEDro scale and downgraded two places as most of the trials scored ≤ 4 on the PEDro scale. Indirectness: Downgraded one place due to wide ranging participant populations or interventions. Inconsistency: Downgraded one place due to an *I*^2^ statistic 50 % or *p* < 0.05 in heterogeneity test of that could not be explained in sensitivity analyses. Imprecision: Downgraded one place due to large confidence intervals and downgraded two places due to very large confidence interval and imprecision. Publication bias: Downgraded one place due to Egger's regression test find a significant publication bias (*p* < 0.05) and downgraded two places as using trim and fill change the results of the outcome. Effect size: Upgraded one place if the effect size was “large”. ^a^ Compared with non-exercise control group. ^b^ Compared with exercise control group.
