# Supplementary material for: Effects of whole-body vibration training on muscle performance in healthy women: A systematic review and meta-analysis of randomized controlled trials
Source: PLoS One. 2025 May 30;20(5):e0322010. doi: 10.1371/journal.pone.0322010 (PMC12124539; doi:10.1371/journal.pone.0322010)
Supplement: S7 Table — (DOCX) [file pone.0322010.s007.docx]

**S7 Table.** List of screened studies.

Records screened (*n* = 1680).

1. Wolfsegger T, Assar H, Topakian R. 3-week whole body vibration does not improve gait function in mildly affected multiple sclerosis patients -- a randomized controlled trial. Journal of the Neurological Sciences 2014 Dec 15;347(1-2):119-123. 2014.
2. 4th International Conference on Human Performance Development through Strength and Conditioning, NSCA 2014. Cultura, Ciencia y Deporte. 2014;9(25 SUPPL.).
3. Yeung SS, Yeung EW. A 5-week whole body vibration training improves peak torque performance but has no effect on stretch reflex in healthy adults: a randomized controlled trial. J Sports Med Phys Fitness. 2015;55(5):397-404.
4. Giacomin J. Absorbed power of small children. Clinical Biomechanics. 2005;20(4):372-80.
5. Lundström R, Holmlund P, Lindberg L. Absorption of energy during vertical whole-body vibration exposure. Journal of Biomechanics. 1998;31(4):317-26.
6. So R, Eto M, Tsujimoto T, Tanaka K. Acceleration training for improving physical fitness and weight loss in obese women. OBESITY RESEARCH & CLINICAL PRACTICE. 2014;8(3):E238-E48.
7. Donaghy A, DeMott T, Allet L, Kim H, Ashton-Miller J, Richardson JK. Accuracy of Clinical Techniques for Evaluating Lower Limb Sensorimotor Functions Associated With Increased Fall Risk. PM and R. 2016;8(4):331-9.
8. Kochman M, Kasprzak M, Kielar A. ACL reconstruction: which additional physiotherapy interventions improve early-stage rehabilitation? A systematic review. International Journal of Environmental Research & Public Health 2022 Nov;19(23):15893. 2022.
9. Knepper A, Zocchi A, Haider S, Caskey R. "An Act of Complete Care": Provider Perspectives on Linking Maternal Contraceptive Care With Well-Baby Visits in Community Health Centers. J Prim Care Community Health. 2024;15:21501319241277421.
10. Ishimatsu K, Meland A, Hansen TAS, Kåsin JI, Wagstaff AS. Action slips during whole-body vibration. Appl Ergon. 2016;55:241-7.
11. Freitas EDS, Frederiksen C, Miller RM, Heishman A, Anderson M, Pardo G, et al. Acute and chronic effects of whole-body vibration on balance, postural stability, and mobility in women with multiple sclerosis. Dose-Response 2018 Dec 27;16(4):Epub. 2018.
12. Theodorou AA, Gerodimos V, Karatrantou K, Paschalis V, Chanou K, Jamurtas AZ, et al. Acute and Chronic Whole-Body Vibration Exercise does not Induce Health-Promoting Effects on The Blood Profile. JOURNAL OF HUMAN KINETICS. 2015;46(1):107-18.
13. Paiva PC, Figueiredo CA, Reis-Silva A, Francisca-Santos A, Paineiras-Domingos LL, Martins-Anjos E, et al. Acute and cumulative effects with whole-body vibration exercises using 2 biomechanical conditions on the flexibility and rating of perceived exertion in individuals with metabolic syndrome: a randomized clinical trial pilot study. Dose-Response 2019 Oct-Dec;17(4):1559325819886495. 2019.
14. Bemben D, Sherk V, Chrisman C, Young K, Smith J, Singh H, et al. Acute bone marker responses to whole-body vibration and resistance exercise in young women. Journal of Bone and Mineral Research. 2011;26.
15. Sherk VD, Chrisman C, Smith J, Young KC, Singh H, Bemben MG, et al. Acute bone marker responses to whole-body vibration and resistance exercise in young women. J Clin Densitom. 2013;16(1):104-9.
16. Dewig DR, Lepley AS, Nilius A, Padua DA, Pietrosimone BG, Wikstrom EA, et al. An Acute Bout of Whole-Body Vibration Does Not Improve Jumping Performance in Those With Anterior Cruciate Ligament Reconstruction. J Athl Train. 2024;59(9):948-54.
17. Bullock N, Martin D, Ross A, Rosemond D, Jordan M, Marino F. An acute bout of whole-body vibration on skeleton start and 30-m sprint performance. EUROPEAN JOURNAL OF SPORT SCIENCE. 2009;9(1):35-9.
18. Dias T, Polito M. Acute Cardiovascular Response during Resistance Exercise with Whole-body Vibration in Sedentary Subjects: A Randomized Cross-over Trial. Research in Sports Medicine. 2015;23(3):253-64.
19. Miyara K, Kawamura K, Matsumoto S, Ohwatashi A, Itashiki Y, Uema T, et al. Acute changes in cortical activation during active ankle movement after whole-body vibration for spasticity in hemiplegic legs of stroke patients: a functional near-infrared spectroscopy study. Top Stroke Rehabil. 2020;27(1):67-74.
20. Rittweger J, Mutschelknauss M, Felsenberg D. Acute changes in neuromuscular excitability after exhaustive whole body vibration exercise as compared to exhaustion by squatting exercise. Clin Physiol Funct Imaging. 2003;23(2):81-6.
21. Krause A, Gollhofer A, Freyler K, Jablonka L, Ritzmann R. Acute corticospinal and spinal modulation after whole body vibration. J Musculoskelet Neuronal Interact. 2016;16(4):327-38.
22. Ameer M, Al Abbad A. Acute effect of upper body vibration on shoulder joint internal and external active position sense in healthy female university students. PHYSICAL EDUCATION OF STUDENTS. 2023;27(5):221-9.
23. n932c RBR. Acute effect of Whole Body Vibration in patients with Rheumatoid Arthritis. http://wwwwhoint/trialsearch/Trial2aspx?TrialID=RBR-2n932c. 2019.
24. Kurt C, Pekünlü E. Acute effect of whole body vibration on isometric strength, squat jump, and flexibility in well-trained combat athletes. Biology of Sport. 2015;32(2):115-22.
25. Dallas G, Kirialanis P, Mellos V. THE ACUTE EFFECT OF WHOLE BODY VIBRATION TRAINING ON FLEXIBILITY AND EXPLOSIVE STRENGTH OF YOUNG GYMNASTS. Biology of sport. 2014;31(3):233‐7.
26. Beerse M, Lelko M, Wu J. Acute effect of whole-body vibration on acceleration transmission and jumping performance in children. Clinical Biomechanics. 2021;81.
27. Brent Feland J, Stevenson DL, Hunter I, Ty Hopkins J, Cochrane DJ. Acute effect of whole-body vibration on electromechanical delay and vertical jump performance. Journal of Musculoskeletal Neuronal Interactions. 2021;21(3):373-8.
28. Bullock N, Martin DT, Ross A, Rosemond CD, Jordan MJ, Marino FE. Acute effect of whole-body vibration on sprint and jumping performance in elite skeleton athletes. Journal of strength and conditioning research / National Strength & Conditioning Association. 2008;22(4):1371-4.
29. Armstrong WJ, Nestle HN, Grinnell DC, Cole LD, Van Gilder EL, Warren GS, et al. The acute effect of whole-body vibration on the hoffmann reflex. Journal of Strength and Conditioning Research. 2008;22(2):471-6.
30. Armstrong WJ, Grinnell DC, Warren GS. The acute effect of whole-body vibration on the vertical jump height. Journal of strength and conditioning research. 2010;24(10):2835‐9.
31. Allam NM, Alenzi RM, Ali LS, Al Muteb SM, Aljabar SA, Altuwayrib HF, et al. Acute Effect of Whole-Body Vibration on Trunk Endurance and Balance in Obese Female Students: Randomized Controlled Trial. Medicina (Kaunas). 2024;60(8).
32. Dominguez-Munoz FJ, Hernandez-Mocholi MA, Villafaina S, Garcia-Gordillo MA, Collado-Mateo D, Gusi N, et al. Acute effects of a whole body vibration session on the vibration perception threshold in patients with type 2 diabetes mellitus. International Journal of Environmental Research & Public Health 2020 Jun;17(12):4356. 2020.
33. Findikoglu G, Kilic-Toprak E, Kilic-Erkek O, Senol H, Bor-Kucukatay M. Acute effects of continuous and intermittent aerobic exercises on hemorheological parameters: a pilot study. Biorheology. 2014;51(4‐5):293‐303.
34. Kim E, Okamoto T, Song J, Lee K. The acute effects of different frequencies of whole-body vibration on arterial stiffness. CLINICAL AND EXPERIMENTAL HYPERTENSION. 2020;42(4):345-51.
35. Saldiran T, Atici E, Rezaei DA, Öztürk Ö, Uslu B, Özcan BA, et al. The acute effects of different intensity whole-body vibration exposure on muscle tone and strength of the lower legs, and hamstring flexibility: A pilot study. Journal of Sport Rehabilitation. 2021;30(2):235-41.
36. Dallas G, Paradisis G, Kirialanis P, Mellos V, Argitaki P, Smirniotou A. The acute effects of different training loads of whole body vibration on flexibility and explosive strength of lower limbs in divers. Biology of Sport. 2015;32(3):235-41.
37. Gerodimos V, Zafeiridis A, Karatrantou K, Vasilopoulou T, Chanou K, Pispirikou E. The acute effects of different whole-body vibration amplitudes and frequencies on flexibility and vertical jumping performance. Journal of Science and Medicine in Sport. 2010;13(4):438-43.
38. Tucker WS, Slone SW. The acute effects of hold-relax proprioceptive neuromuscular facilitation with vibration therapy on glenohumeral internal-rotation deficit. Journal of Sport Rehabilitation 2016;25(3):248-254. 2016.
39. Peer KS, Barkley JE, Knapp DM. The Acute Effects of Local Vibration Therapy on Ankle Sprain and Hamstring Strain Injuries. PHYSICIAN AND SPORTSMEDICINE. 2009;37(4):31-8.
40. Menéndez H, Ferrero C, Martín-Hernández J, Figueroa A, Marín PJ, Herrero AJ. Acute effects of simultaneous electromyostimulation and vibration on leg blood flow in spinal cord injury. Spinal Cord. 2016;54(5):383-9.
41. Chen CH, Hsu CH, Chu LP, Chiu CH, Yang WC, Yu KW, et al. Acute Effects of Static Stretching Combined with Vibration and Nonvibration Foam Rolling on the Cardiovascular Responses and Functional Fitness of Older Women with Prehypertension. BIOLOGY-BASEL. 2022;11(7).
42. Lauper M, Kuhn A, Gerber R, Luginbuehl Greco H, Radlinger L. Acute effects of stochastic and sinusoidal whole body vibration on pelvic floor muscle activation. Physiotherapy (United Kingdom). 2011;97:eS715.
43. Johnson AW, Warcup CN, Seeley MK, Eggett D, Feland JB. The acute effects of stretching with vibration on dynamic flexibility in young female gymnasts. JOURNAL OF SPORTS MEDICINE AND PHYSICAL FITNESS. 2019;59(2):210-6.
44. Cardinale M, Lim J. The acute effects of two different whole body vibration frequencies on vertical jump performance. Medicina dello Sport. 2003;56(4):287-92.
45. Rønnestad BR. Acute effects of various whole body vibration frequencies on 1RM in trained and untrained subjects. Journal of strength and conditioning research. 2009;23(7):2068‐72.
46. Cloak R, Nevill A, Smith J, Wyon M. The acute effects of vibration stimulus following FIFA 11+on agility and reactive strength in collegiate soccer players. JOURNAL OF SPORT AND HEALTH SCIENCE. 2014;3(4):293-8.
47. Watanabe H, Takahara M, Katakami N, Kanamoto T, Nakata K, Shimomura I. Acute effects of whole body vibration exercise on post-load glucose metabolism in healthy men: a pilot randomized crossover trial. Endocrine 2022 Mar;75(3):752-759. 2022.
48. Rendos NK, Jun HP, Pickett NM, Lew Feirman K, Harriell K, Lee SY, et al. Acute effects of whole body vibration on balance in persons with and without chronic ankle instability. Research in sports medicine (Print). 2017;25(4):391‐407.
49. Licurci M, de Almeida Fagundes A, Arisawa E. Acute effects of whole body vibration on heart rate variability in elderly people. J Bodyw Mov Ther. 2018;22(3):618-21.
50. den Heijer AE, Groen Y, Fuermaier AB, van Heuvelen MJ, van der Zee EA, Tucha L, et al. Acute Effects of Whole Body Vibration on Inhibition in Healthy Children. PLoS One. 2015;10(11):e0140665.
51. Sousa-Goncalves CR, Tringali G, Tamini S, de Micheli R, Soranna D, Taiar R, et al. Acute effects of whole-body vibration alone or in combination with maximal voluntary contractions on cardiorespiratory, musculoskeletal, and neuromotor fitness in obese male adolescents. Dose-Response 2019 Oct-Dec;17(4):1559325819890492. 2019.
52. Chouza M, Arias P, Vinas S, Cudeiro J. Acute effects of whole-body vibration at 5, 6, and 9 Hz on balance and gait in patients with Parkinson's disease. Movement Disorders 2011 Apr;26(5):920-921. 2011.
53. Moura-Fernandes MC, Moreira-Marconi E, de Meirelles AG, Reis-Silva A, de Souza LFF, da Silva ALP, et al. Acute Effects of Whole-Body Vibration Exercise on Pain Level, Functionality, and Rating of Exertion of Elderly Obese Knee Osteoarthritis Individuals: A Randomized Study. APPLIED SCIENCES-BASEL. 2020;10(17).
54. Tamini S, De Micheli R, Tringali G, Bernardo-Filho M, Sartorio A. Acute Effects of Whole-Body Vibration Exercises at 2 Different Frequencies Versus an Aerobic Exercise on Some Cardiovascular, Neuromotor and Musculoskeletal Parameters in Adult Patients With Obesity. Dose-Response. 2020;18(4).
55. Kang J, Bushi JA, Ratamess NA, Faigenbaum AD, Klei S, Maconi D, et al. Acute effects of whole-body vibration on energy metabolism during aerobic exercise. J Sports Med Phys Fitness. 2016;56(7-8):834-42.
56. Dickin DC, Faust KA, Wang H, Frame J. The acute effects of whole-body vibration on gait parameters in adults with cerebral palsy. Journal of Musculoskeletal Neuronal Interactions. 2013;13(1):19-26.
57. Bagheri J, Van Den Berg-Emons RJ, Pel JJ, Horemans HL, Stam HJ. Acute effects of whole-body vibration on jump force and jump rate of force development: A comparative study of different devices. Journal of Strength and Conditioning Research. 2012;26(3):691-6.
58. Jackson KJ, Merriman HL, Vanderburgh PM, Brahler CJ. Acute effects of whole-body vibration on lower extremity muscle performance in persons with multiple sclerosis. Journal of neurologic physical therapy. 2008;32(4):171‐6.
59. Bagheri J, Van Den Berg RHJG, Pel JJ, Horemans HLD, Stam HJ. Acute effects of whole-body vibration on neuromuscular response of the vastus lateralis muscle: A comparative study of different devices. European Journal of Neurology. 2012;19:584.
60. Marín PJ, Herrero AJ, García-LóPez D, Rhea MR, López-Chicharro J, González-Gallego J, et al. Acute effects of whole-body vibration on neuromuscular responses in older individuals: Implications for prescription of vibratory stimulation. Journal of Strength and Conditioning Research. 2012;26(1):232-9.
61. Mahbub MH, Hase R, Yamaguchi N, Hiroshige K, Harada N, Bhuiyan ANH, et al. Acute effects of whole-body vibration on peripheral blood flow, vibrotactile perception and balance in older adults. International Journal of Environmental Research & Public Health 2020 Feb;17(3):1069. 2020.
62. Maciejczyk M, Bawelski M, Wiecek M, Szygula Z, Michailov ML, Vadasová B, et al. Acute Effects of Whole-Body Vibration on Resting Metabolic Rate and Substrate Utilisation in Healthy Women. BIOLOGY-BASEL. 2022;11(5).
63. Sales RM, Cerqueira MS, Bezerra de Morais AT, de Paiva Lima CRO, Lemos A, Galvao de Moura Filho A. Acute effects of whole-body vibration on spinal excitability level and ankle plantar flexion spasticity in post-stroke individuals: a randomized controlled trial. Journal of Bodywork and Movement Therapies 2020 Apr;24(2):37-42. 2020.
64. Silva AT, Dias MP, Calixto R, Jr., Carone AL, Martinez BB, Silva AM, et al. Acute effects of whole-body vibration on the motor function of patients with stroke. American Journal of Physical Medicine & Rehabilitation 2014 Apr;93(4):310-319. 2014.
65. Sa-Caputo DC, Paineiras-Domingos LL, Oliveira R, Neves MFT, Brandao A, Marin PJ, et al. Acute effects of whole-body vibration on the pain level, flexibility, and cardiovascular responses in individuals with metabolic syndrome. Dose-Response 2018 Oct 7;16(4):Epub. 2018.
66. Perchthaler D, Hauser S, Heitkamp HC, Hein T, Grau S. Acute Effects of Whole-Body Vibration on Trunk and Neck Muscle Activity in Consideration of Different Vibration Loads. JOURNAL OF SPORTS SCIENCE AND MEDICINE. 2015;14(1):155-62.
67. Ye J, Ng G, Yuen K. Acute effects of whole-body vibration on trunk muscle functioning in young healthy adults. Journal of strength and conditioning research / National Strength & Conditioning Association. 2014;28(10):2872-9.
68. Wirth B, Zurfluh S, Müller R. Acute effects of whole-body vibration on trunk muscles in young healthy adults. J Electromyogr Kinesiol. 2011;21(3):450-7.
69. Corredoira F, Durán JA. Acute effects of whole-body vibration training in female basketball players. Revista Internacional de Medicina y Ciencias de la Actividad Fisica y del Deporte. 2012;12(48):625-33.
70. Aoyama A, Yamaoka-Tojo M, Obara S, Shimizu E, Fujiyoshi K, Noda C, et al. Acute effects of whole-body vibration training on endothelial function and cardiovascular response in elderly patients with cardiovascular disease: A single-arm pilot study. International Heart Journal. 2019;60(4):854-61.
71. Andreu L, Ramos-Campo DJ, Avila-Gandia V, Freitas TT, Chung LH, Rubio-Arias JA. Acute effects of whole-body vibration training on neuromuscular performance and mobility in hypoxia and normoxia in persons with multiple sclerosis: a crossover study. Multiple sclerosis and related disorders. 2020;37:101454.
72. Zeigler ZS, Swan PD. Acute effects of whole-body vibration with resistance exercise on postexercise blood pressure and oxygen consumption in prehypertensive adults. Journal of Exercise Science and Fitness 2016 Jun;14(1):14-23. 2016.
73. García-López D, Garatachea N, Marín PJ, Martín T, Herrero AJ. Acute effects of whole-body vibrations on balance, maximal force and perceived exertion: Vertical platform versus oscillating platform. European Journal of Sport Science. 2012;12(5):425-30.
74. Annino G, Manzi V, Buselli P, Ruscello B, Franceschetti F, Romagnoli C, et al. Acute effects of whole-body vibrations on the fatigue induced by multiple repeated sprint ability test in soccer players. The Journal of Sports Medicine and Physical Fitness 2022 Jun;62(6):788-794. 2022.
75. Kilic-Toprak E, Yapici A, Kilic-Erkek O, Koklu Y, Tekin V, Alemdaroglu U, et al. Acute effects of Yo-Yo intermittent recovery test level 1 (Yo-YoIR1) on hemorheological parameters in female volleyball players. Clin Hemorheol Microcirc. 2015;60(2):191-9.
76. Dallas GC, Dallas CG, Tsolakis C. Acute enhancement of jumping performance after different plyometric stimuli in high level gymnasts is associated with postactivation potentiation. MEDICINA DELLO SPORT. 2019;72(1):25-36.
77. Jacobs PL, Burns P. Acute enhancement of lower-extremity dynamic strength and flexibility with whole-body vibration. Journal of strength and conditioning research. 2009;23(1):51‐7.
78. Kramer A, Gollhofer A, Ritzmann R. Acute exposure to microgravity does not influence the H-reflex with or without whole body vibration and does not cause vibration-specific changes in muscular activity. J Electromyogr Kinesiol. 2013;23(4):872-8.
79. Rittweger J, Beller G, Felsenberg D. Acute physiological effects of exhaustive whole-body vibration exercise in man. Clin Physiol. 2000;20(2):134-42.
80. Pessoa MF, de Souza HCM, da Silva APV, Clemente RD, Brandao DC, de Andrade AD. Acute Whole Body Vibration Decreases the Glucose Levels in Elderly Diabetic Women. REHABILITATION RESEARCH AND PRACTICE. 2018;2018.
81. Cochrane DJ, Stannard SR. Acute whole body vibration training increases vertical jump and flexibility performance in elite female field hockey players. British Journal of Sports Medicine. 2005;39(11):860-5.
82. Kavanaugh A, Ramsey MW, Sands WA, Haff GG, Stone MH. Acute whole-body vibration does not affect static jump performance. European Journal of Sport Science. 2011;11(1):19-25.
83. Yeung EW, Lau CC, Kwong APK, Sze YM, Zhang WY, Yeung SS. Acute Whole-Body Vibration Does Not Facilitate Peak Torque and Stretch Reflex in Healthy Adults. Journal of sports science & medicine. 2014;13(1):30‐5.
84. Cochrane DJ, Stannard SR, Firth EC, Rittweger J. Acute whole-body vibration elicits post-activation potentiation. European Journal of Applied Physiology. 2010;108(2):311-9.
85. Coelho-Oliveira AC, Lacerda ACR, de Souza ALC, Santos LMM, da Fonseca SF, Dos Santos JM, et al. Acute Whole-Body Vibration Exercise Promotes Favorable Handgrip Neuromuscular Modifications in Rheumatoid Arthritis: A Cross-Over Randomized Clinical. Biomed Res Int. 2021;2021:9774980.
86. Ritzmann R, Krause A, Freyler K, Gollhofer A. Acute whole-body vibration increases reciprocal inhibition. Hum Mov Sci. 2018;60:191-201.
87. Krause A, Gollhofer A, Lee K, Freyler K, Becker T, Kurz A, et al. Acute whole-body vibration reduces post-activation depression in the triceps surae muscle. Hum Mov Sci. 2020;72:102655.
88. Bosco C, Colli R, Introini E, Cardinale M, Tsarpela O, Madella A, et al. Adaptive responses of human skeletal muscle to vibration exposure. Clinical physiology (Oxford, England). 1999;19(2):183‐7.
89. Duc S, Rønnestad BR, Bertucci W. Adding Whole-Body Vibration to Preconditioning Squat Exercise Increases Cycling Sprint Performance. Journal of strength and conditioning research. 2020;34(5):1354‐61.
90. Marín PJ, García-Gutiérrez MT, Da Silva-Grigoletto ME, Hazell TJ. The addition of synchronous whole-body vibration to battling rope exercise increases skeletal muscle activity. J Musculoskelet Neuronal Interact. 2015;15(3):240-8.
91. Dickin DC, Heath JE. Additive Effect of Repeated Bouts of Individualized Frequency Whole Body Vibration on Postural Stability in Young Adults. Journal of Applied Biomechanics. 2014;30(4):529-33.
92. Qing FZ, Xie PF, Liem YS, Chen Y, Chen XN, Zhu XD, et al. Administration duration influences the effects of low-magnitude, high-frequency vibration on ovariectomized rat bone. JOURNAL OF ORTHOPAEDIC RESEARCH. 2016;34(7):1147-57.
93. Kates SL, Kates OS, Mendelson DA. Advances in the medical management of osteoporosis. Injury. 2007;38(SUPPL. 3):17-23.
94. Ghione E, Bergsland N, Dwyer MG, Hagemeier J, Jakimovski D, Paunkoski I, et al. Aging and Brain Atrophy in Multiple Sclerosis. J Neuroimaging. 2019;29(4):527-35.
95. Kimura K. Aging of bone density in the second metacarpal. Okajimas Folia Anat Jpn. 1991;68(4):251-7.
96. Donocik K, Hartman-Petrycka M, Lebiedowska A, Błońska-Fajfrowska B. Alterations in the ability to maintain balance as a result of stochastic resonance whole body vibration in women. PLoS One. 2017;12(9):e0185179.
97. Kabata-Pizuch A, Suder A, Handzlik-Waszkiewicz P, Teleglów A, Marchewka A. Alterations of Body Composition, Blood Morphology and Fibrinogen Concentration after Vibration Therapy in Older Adult Women: A Randomized Controlled Trial. JOURNAL OF CLINICAL MEDICINE. 2023;12(20).
98. Ricci I, Sofi F, Liotta AA, Fedi S, Macchi C, Pratesi G, et al. Alterations of haemorheological parameters in patients with peripheral arterial disease. Clin Hemorheol Microcirc. 2013;55(2):271-6.
99. Baessler K, Bell BE. Alternative methods to pelvic floor muscle awareness and training. Pelvic Floor Re-education: Principles and Practice2008. p. 208-12.
100. Chew SH, Meighan Smith Tomic M, Cheung AT. Alzheimer's disease: more than amyloid. Clin Hemorheol Microcirc. 2010;46(1):69-73.
101. Oosthuyse T, Viedge A, McVeigh J, Avidon I. Anaerobic power in road cyclists is improved after 10 weeks of whole-body vibration training. Journal of strength and conditioning research. 2013;27(2):485‐94.
102. Kromka-Szydek M, Chwalik-Pilszyk G, Dziechciowski Z. Analysis of vibrations transmitted to the feet of a wheelchair user. Acta Bioeng Biomech. 2024;26(3):35-45.
103. DiGiovine CP, Cooper RA, Wolf E, Fitzgerald SG, Boninger ML. Analysis of whole-body vibration during manual wheelchair propulsion: a comparison of seat cushions and back supports for individuals without a disability. Assist Technol. 2003;15(2):129-44.
104. Kuijer PP, van der Molen HF, Schop A, Moeijes F, Frings-Dresen MH, Hulshof CT. Annual incidence of non-specific low back pain as an occupational disease attributed to whole-body vibration according to the National Dutch Register 2005-2012. Ergonomics. 2015;58(7):1232-8.
105. Glattke KE, Tummala SV, Chhabra A. Anterior cruciate ligament reconstruction recovery and rehabilitation: a systematic review. Journal of Bone and Joint Surgery -- American Volume 2022 Apr 20;104(8):739-754. 2022.
106. Xie P, Cui L, Shan Y, Kang WY. Antithrombotic Effect and Mechanism of Radix Paeoniae Rubra. Biomed Res Int. 2017;2017:9475074.
107. Mansfield NJ, Holmlund P, Lundström R. Apparent mass and absorbed power during exposure to whole-body vibration and repeated shocks. Journal of Sound and Vibration. 2001;248(3):427-40.
108. Giacomin JA. Apparent mass of small children: Modelling. International Journal of Industrial Ergonomics. 2007;37(3):183-95.
109. Huang Y, Zhang P, Liang S. Apparent mass of the seated human body during vertical vibration in the frequency range 2-100 Hz. Ergonomics. 2020;63(9):1150-63.
110. Nawayseh N, Hamdan S. Apparent mass of the standing human body when using a whole-body vibration training machine: Effect of knee angle and input frequency. JOURNAL OF BIOMECHANICS. 2019;82:291-8.
111. Lau RWK, Chung FHF, Yip SP, Li LSW, Pang MYC. Application of whole body vibration therapy in subacute stroke patients: feasibility, safety, and effects on bone metabolism and neuromotor function. Osteoporosis international. 2010;21:S750‐.
112. Bidonde J, Busch AJ, Webber SC, Schachter CL, Danyliw A, Overend TJ, et al. Aquatic exercise training for fibromyalgia. COCHRANE DATABASE OF SYSTEMATIC REVIEWS. 2014(10).
113. Kucukdeveci AA. Are orthoses and physical modalities effective for osteoporosis and/or osteoporotic fractures? Osteoporosis International. 2020;31(SUPPL 1):S127.
114. Josephs KA, Whitwell JL, Parisi JE, Knopman DS, Boeve BF, Geda YE, et al. Argyrophilic grains: a distinct disease or an additive pathology? Neurobiol Aging. 2008;29(4):566-73.
115. Hansen TA, Kåsin JI, Edvardsen A, Christensen CC, Wagstaff AS. Arterial oxygen pressure following whole-body vibration at altitude. Aviat Space Environ Med. 2012;83(4):431-5.
116. Caimi G, Urso C, Brucculeri S, Amato C, Carlisi M, Lo Presti R. An assessment of the hemorheological profile in patients with subclinical carotid atherosclerosis divided in relation to the number of cardiovascular risk factors and different degrees of insulin resistance. Clin Hemorheol Microcirc. 2021;78(4):417-28.
117. Paineiras-Domingos LL, Sá-Caputo DD, Reis AS, Santos F, Sousa-Gonçalves CR, dos Anjos EM, et al. Assessment Through the Short Physical Performance Battery of the Functionality in Individuals With Metabolic Syndrome Exposed to Whole-Body Vibration Exercises. DOSE-RESPONSE. 2018;16(3).
118. Essien SK, Bath B, Koehncke N, Trask C. Association Between Farm Machinery Operation and Low Back Disorder in Farmers: A Retrospective Cohort Study. J Occup Environ Med. 2016;58(6):e212-7.
119. Li Y, Tian XX, Liu T, Wang RT. Association between whole blood viscosity and arterial stiffness in patients with type 2 diabetes mellitus. Endocrine. 2015;49(1):148-54.
120. Kwaku Essien S, Trask C, Khan M, Boden C, Bath B. Association Between Whole-Body Vibration and Low-Back Disorders in Farmers: A Scoping Review. J Agromedicine. 2018;23(1):105-20.
121. Gyawali P, Richards RS. Association of altered hemorheology with oxidative stress and inflammation in metabolic syndrome. Redox Rep. 2015;20(3):139-44.
122. de Simone G, Devereux RB, Chinali M, Best LG, Lee ET, Welty TK. Association of blood pressure with blood viscosity in american indians: the Strong Heart Study. Hypertension. 2005;45(4):625-30.
123. Ozcan Cetin EH, Cetin MS, Çağlı K, Temizhan A, Özbay MB, Ediboglu E, et al. The association of estimated whole blood viscosity with hemodynamic parameters and prognosis in patients with heart failure. Biomark Med. 2019;13(2):69-82.
124. Banerjee R, Nageswari K, Puniyani RR. Association of hemorheological parameters and risk of stroke in hypertensives of Indian origin. Clin Exp Hypertens. 2000;22(7-8):687-94.
125. Zhu W, Li M, Huang X, Neubauer H. Association of hyperviscosity and subclinical atherosclerosis in obese schoolchildren. Eur J Pediatr. 2005;164(10):639-45.
126. Juutinen L, Ahinko K, Hagman S, Basnyat P, Jääskeläinen O, Herukka SK, et al. The association of menopausal hormone levels with progression-related biomarkers in multiple sclerosis. Mult Scler Relat Disord. 2024;85:105517.
127. Roberts RO, Knopman DS, Przybelski SA, Mielke MM, Kantarci K, Preboske GM, et al. Association of type 2 diabetes with brain atrophy and cognitive impairment. Neurology. 2014;82(13):1132-41.
128. Yu XY, Li Y, Liu T, Wang RT. Association of whole blood viscosity with non-alcoholic fatty liver disease. Clin Hemorheol Microcirc. 2015;62(4):335-43.
129. Hammes MS, Watson S, Coe FL, Ahmed F, Beltran E, Dhar P. Asymmetric dimethylarginine and whole blood viscosity in renal failure. Clin Hemorheol Microcirc. 2015;59(3):245-55.
130. Han SW, Lee DY, Choi DS, Han B, Kim JS, Lee HD. Asynchronous Alterations of Muscle Force and Tendon Stiffness Following 8 Weeks of Resistance Exercise with Whole-Body Vibration in Older Women. Journal of aging and physical activity. 2017;25(2):287‐94.
131. Rapp W, Boeer J, Albrich C, Heitkamp HC. Auswirkung eines vibrations- und krafttrainings auf die beinmuskulatur bei gonarthrosepatienten (Efficiency of vibration or strength training for knee stability in osteoarthritis of the knee) Aktuelle Rheumatologie 2009 Aug;34(4):240-245. 2009.
132. Wang C, Beadnall HN, Hatton SN, Bader G, Tomic D, Silva DG, et al. Automated brain volumetrics in multiple sclerosis: a step closer to clinical application. J Neurol Neurosurg Psychiatry. 2016;87(7):754-7.
133. Ishii K, Soma T, Kono AK, Sasaki H, Miyamoto N, Fukuda T, et al. Automatic volumetric measurement of segmented brain structures on magnetic resonance imaging. Radiat Med. 2006;24(6):422-30.
134. Johanning E. Back disorders and health problems among subway train operators exposed to whole-body vibration. Scand J Work Environ Health. 1991;17(6):414-9.
135. Chen C, Cheng B, Wang Z, Chen D, Tao X, Cavanaugh JM, editors. Back muscle activity while operating a vehicle. Lecture Notes in Electrical Engineering; 2013.
136. Korman M, Herling Z, Levy I, Egbarieh N, Engel-Yeger B, Karni A. Background matters: Minor vibratory stimulation during motor skill acquisition selectively reduces off-line memory consolidation. Neurobiol Learn Mem. 2017;140:27-32.
137. Müller SM, Fischer M, Itin P, Nüesch C, Hartard M, Brandt O, et al. Bad vibrations: Insights into the mysterious on/off itch and erythema during whole body vibration exercise. Experimental Dermatology. 2018;27(3):e78.
138. Sun C, Qi B, Huang X, Chen M, Jin Z, Zhang Y, et al. Baduanjin exercise: A potential promising therapy toward osteoporosis. Frontiers in Medicine. 2022;9.
139. Maynard TS, Burns CA. Balance Rehabilitation in Blinded Elders, BRIBE. Journal of Cardiopulmonary Rehabilitation and Prevention. 2023;43(5):E12.
140. Runge M, Rehfeld G, Resnicek E. Balance training and exercise in geriatric patients. Journal of Musculoskeletal & Neuronal Interactions 2000 Sep;1(1):61-65. 2000.
141. van de Pol LA, van der Flier WM, Korf ES, Fox NC, Barkhof F, Scheltens P. Baseline predictors of rates of hippocampal atrophy in mild cognitive impairment. Neurology. 2007;69(15):1491-7.
142. Choi JS, Kim JG, Cho JH, Tack GR. A BASIC STUDY ON THE EFFECTS OF VIBRATOR-ATTACHED LEG-PRESS ON THE KNEE AND ANKLE JOINT TORQUES. JOURNAL OF MECHANICS IN MEDICINE AND BIOLOGY. 2020;20(9).
143. Sobrinho Neto SB, Moreira Marconi E, Ribeiro Kutter C, Frederico EHFF, de Castro de Paiva P, Froes Meyer P, et al. Beneficial effects of whole body mechanical vibration alone or combined with auriculotherapy in the pain and in flexion of knee of individuals with knee osteoarthritis. Acupuncture & Electro-Therapeutics Research. 2017;42(3):185-201.
144. Cardoso A, Sá-Caputo DC, Asad NR, van Heuvelen MJ, van der Zee EA, Ribeiro-Carvalho A, et al. Beneficial effects of whole-body vibration exercise for brain disorders in experimental studies with animal models: a systematic review. Behav Brain Res. 2022;431:113933.
145. Rosado H, Pereira C, Bravo J, Carvalho J, Raimundo A. Benefits of Two 24-Week Interactive Cognitive–Motor Programs on Body Composition, Lower-Body Strength, and Processing Speed in Community Dwellings at Risk of Falling: A Randomized Controlled Trial. International Journal of Environmental Research and Public Health. 2022;19(12).
146. Greulich T, Nell C, Koepke J, Fechtel J, Franke M, Schmeck B, et al. Benefits of whole body vibration training in patients hospitalised for COPD exacerbations -- a randomized clinical trial. BMC Pulmonary Medicine 2014 Apr 11;14(60):Epub. 2014.
147. Yu F, Lin H, Li Q, Jia X, Yu T, Ma J, et al. Benefits of Whole Body Vibration Training in Patients with COPD Exacerbations. European Respiratory Journal. 2022;60.
148. Furness T, Joseph C, Naughton G, Welsh L, Lorenzen C. Benefits of whole-body vibration to people with COPD: a community-based efficacy trial. BMC pulmonary medicine. 2014;14(1).
149. Alvarez-Alvarado S, Jaime SJ, Ormsbee MJ, Campbell JC, Post J, Pacilio J, et al. Benefits of whole-body vibration training on arterial function and muscle strength in young overweight/obese women. Hypertension Research. 2017;40(5):487-92.
150. Santos-Filho SD, Cameron MH, Bernardo-Filho M. Benefits of whole-body vibration with an oscillating platform for people with multiple sclerosis: a systematic review. Multiple Sclerosis International 2012 May 17;(274728):Epub. 2012.
151. Sá-Caputo D, Gonçalves CR, Morel DS, Marconi EM, Fróes P, Rufino R, et al. Benefits of Whole-Body Vibration, as a Component of the Pulmonary Rehabilitation, in Patients with Chronic Obstructive Pulmonary Disease: A Narrative Review with a Suitable Approach. EVIDENCE-BASED COMPLEMENTARY AND ALTERNATIVE MEDICINE. 2016;2016.
152. Faes Y, Salathé CR, Herlig ML, Elfering A. Beyond physiology: Acute effects of side-alternating whole-body vibration on well-being, flexibility, balance, and cognition using a light and portable platform A randomized controlled trial. FRONTIERS IN SPORTS AND ACTIVE LIVING. 2023;5.
153. Kim HJ, Martin BJ. Biodynamic characteristics of upper limb reaching movements of the seated human under whole-body vibration. J Appl Biomech. 2013;29(1):12-22.
154. Nawayseh N, AlBaiti S. Biodynamic responses to whole-body vibration training: A systematic review. Journal of Applied Biomechanics. 2021;37(5):494-507.
155. Stania M, Chmielewska D, Kwasna K, Smykla A, Taradaj J, Juras G. Bioelectrical activity of the pelvic floor muscles during synchronous whole-body vibration - a randomized controlled study. BMC UROLOGY. 2015;15.
156. Perin N, Casara D, Bertoli M, Di Landro D, Naso A, Gasparotto ML, et al. Biofiltration vs. bicarbonate dialysis: influence on plasma volume changes and extravascular fluid mobilization. Int J Artif Organs. 1986;9 Suppl 3:143-6.
157. Zhu J, Shen H, Cui Y, Fogel GR, Liao Z, Liu W. Biomechanical Evaluation of Transforaminal Lumbar Interbody Fusion with Coflex-F and Pedicle Screw Fixation: Finite Element Analysis of Static and Vibration Conditions. Orthop Surg. 2022;14(9):2339-49.
158. Li MCM, Cheng YK, Cui C, Chow SKH, Wong RMY, Kwok TCY, et al. Biophysical and nutritional combination treatment for myosteatosis in patients with sarcopenia: A study protocol for single-blinded randomised controlled trial. BMJ Open. 2024;14(1).
159. Høieggen A, Fossum E, Nesbitt SD, Palmieri V, Kjeldsen SE. Blood viscosity, plasma adrenaline and fasting insulin in hypertensive patients with left ventricular hypertrophy. ICARUS, a LIFE Substudy. Insulin CARotids US Scandinavica. Blood Press. 2000;9(2-3):83-90.
160. Runge WO, Ruppert DS, Marcellin-Little DJ, Dahners LE, Harrysson OLA, Weinhold PS. Bone changes after short-term whole body vibration are confined to cancellous bone. Journal of Musculoskeletal Neuronal Interactions. 2018;18(4):485-92.
161. Meyer NL, Shaw JM, Manore MM, Dolan SH, Subudhi AW, Shultz BB, et al. Bone mineral density of Olympic-level female winter sport athletes. MEDICINE AND SCIENCE IN SPORTS AND EXERCISE. 2004;36(9):1594-601.
162. Xie PF, Tang ZR, Qing FZ, Chen XN, Zhu XD, Fan YJ, et al. Bone mineral density, microarchitectural and mechanical alterations of osteoporotic rat bone under long-term whole-body vibration therapy. JOURNAL OF THE MECHANICAL BEHAVIOR OF BIOMEDICAL MATERIALS. 2016;53:341-9.
163. Liphardt AM, Kan M, Schipilow JD, Steinhoff A, Bauer A, Hanley D, et al. Bone quality in osteopenic post-menopausal women is not improved during 12 months of whole body vibration training. Journal of Bone and Mineral Research. 2013;28.
164. Liphardt AM, Schipilow J, Hanley DA, Boyd SK. Bone quality in osteopenic postmenopausal women is not improved after 12 months of whole-body vibration training. Osteoporosis International. 2015;26(3):911-20.
165. Stolzenberg N, Belavý DL, Beller G, Armbrecht G, Semler J, Felsenberg D. Bone strength and density via pQCT in post-menopausal osteopenic women after 9 months resistive exercise with whole body vibration or proprioceptive exercise. Journal of musculoskeletal & neuronal interactions. 2013;13(1):66‐76.
166. Knopman DS, Jack CR, Jr., Kramer JH, Boeve BF, Caselli RJ, Graff-Radford NR, et al. Brain and ventricular volumetric changes in frontotemporal lobar degeneration over 1 year. Neurology. 2009;72(21):1843-9.
167. Zoghi M, Galea M. Brain motor control assessment post intensive whole-body exercise vs. upper body exercise after spinal cord injury. Neuroimmunology and neuroinflammation. 2019;6(14).
168. Weiner MF, de la Plata CM, Fields BA, Womack KB, Rosenberg RN, Gong YH, et al. Brain MRI, apoliprotein E genotype, and plasma homocysteine in American Indian Alzheimer disease patients and Indian controls. Curr Alzheimer Res. 2009;6(1):52-8.
169. Taylor HG, Filipek PA, Juranek J, Bangert B, Minich N, Hack M. Brain volumes in adolescents with very low birth weight: effects on brain structure and associations with neuropsychological outcomes. Dev Neuropsychol. 2011;36(1):96-117.
170. Chen JH, Lee YW, Chan SW, Yeh DC, Chang RF. Breast Density Analysis with Automated Whole-Breast Ultrasound: Comparison with 3-D Magnetic Resonance Imaging. Ultrasound Med Biol. 2016;42(5):1211-20.
171. Dabbs NC, Tran TT, Garner JC, Brown LE. A Brief Review: Using Whole-Body Vibration to Increase Acute Power and Vertical Jump Performance. STRENGTH AND CONDITIONING JOURNAL. 2012;34(5):78-84.
172. Winters-Stone KM, Medysky ME, Stoyles S, Bumgarner L, Witzke K. A brief whole-body vibration intervention to avoid weight gain in college students: A randomized controlled pilot trial. JOURNAL OF AMERICAN COLLEGE HEALTH. 2022;70(4):1010-8.
173. Correa CB, Camargos GV, Chatterjee M, Mesquita MF, Del Bel Cury AA, Naert I, et al. Can the alendronate dosage be altered when combined with high-frequency loading in osteoporosis treatment? Osteoporos Int. 2017;28(4):1287-93.
174. Rosado H, Bravo J, Raimundo A, Carvalho J, Almeida G, Pereira C. Can two multimodal psychomotor exercise programs improve attention, affordance perception, and balance in community dwellings at risk of falling? A randomized controlled trial. BMC Public Health 2021 Jul 11;21(2336):Epub. 2021.
175. Paineiras-Domingos LL, de Sá-Caputo DD, Moreira-Marconi E, Morel DS, Dionello CD, Sousa-Gonçalves CR, et al. Can whole body vibration exercises affect growth hormone concentration? A systematic review. GROWTH FACTORS. 2017;35(4-5):189-200.
176. Paineiras-Domingos LL, Sá-Caputo DDC, Francisca-Santos A, Reis-Silva A, Carvalho-Lima RP, Neves MFT, et al. Can whole body vibration exercises promote improvement on quality of life and on chronic pain level of metabolic syndrome patients? A pseudorandomized crossover study. J Appl Physiol (1985). 2020;128(4):934-40.
177. ChiCtr. Can whole-body vibration treatment optimize cognitive function in older adults who have sarcopenia and are at risk of dementia: a randomized controlled trial. https://trialsearchwhoint/Trial2aspx?TrialID=ChiCTR2100053912. 2021.
178. Lantoine P, Lecocq M, Bougard C, Dousset E, Marqueste T, Bourdin C, et al. Car seat impact on driver's sitting behavior and perceived discomfort during prolonged real driving on varied road types. PLoS One. 2021;16(11):e0259934.
179. Sigusch HH, Zimmermann B, Kuntze T, Gerth J. Cardiac tamponade following aortic root erosion by an Amplatzer PFO-Occluder in a 41-year-old woman: Only a matter of sizing? J Cardiol Cases. 2021;23(3):123-6.
180. Lage VKS, Lacerda ACR, Neves CDC, Chaves MGA, Soares AA, Lima LP, et al. Cardiorespiratory responses in different types of squats and frequencies of whole body vibration in patients with chronic obstructive pulmonary disease. J Appl Physiol (1985). 2019;126(1):23-9.
181. Holsworth RE, Jr., Cho YI, Weidman JJ, Sloop GD, St Cyr JA. Cardiovascular benefits of phlebotomy: relationship to changes in hemorheological variables. Perfusion. 2014;29(2):102-16.
182. Liao LR, Ng GY, Jones AY, Pang MY. Cardiovascular Stress Induced by Whole-Body Vibration Exercise in Individuals With Chronic Stroke. Phys Ther. 2015;95(7):966-77.
183. Palmer KT, Harris CE, Griffin MJ, Bennett J, Reading I, Sampson M, et al. Case-control study of low-back pain referred for magnetic resonance imaging, with special focus on whole-body vibration. Scand J Work Environ Health. 2008;34(5):364-73.
184. Oh S, Shida T, Sawai A, Maruyama T, Eguchi K, Isobe T, et al. A cceleration training for managing nonalcoholic fatty liver disease: a pilot study. THERAPEUTICS AND CLINICAL RISK MANAGEMENT. 2014;10:925-36.
185. Ding K, Marquez de la Plata C, Wang JY, Mumphrey M, Moore C, Harper C, et al. Cerebral atrophy after traumatic white matter injury: correlation with acute neuroimaging and outcome. J Neurotrauma. 2008;25(12):1433-40.
186. Bogaerts A, Delecluse C, Boonen S, Claessens AL, Milisen K, Verschueren SM. Changes in balance, functional performance and fall risk following whole body vibration training and vitamin D supplementation in institutionalized elderly women. A 6 month randomized controlled trial. Gait & posture. 2011;33(3):466‐72.
187. Sañudo B, Carrasco L, de Hoyo M, Oliva-Pascual-Vaca Á, Rodríguez-Blanco C. Changes in body balance and functional performance following whole-body vibration training in patients with fibromyalgia syndrome: a randomized controlled trial. Journal of rehabilitation medicine. 2013;45(7):678‐84.
188. Erceg DN, Anderson LJ, Nickles CM, Lane CJ, Weigensberg MJ, Schroeder ET. Changes in bone biomarkers, BMC, and insulin resistance following a 10-week whole body vibration exercise program in overweight Latino boys. International Journal of Medical Sciences 2015;12(6):494-501. 2015.
189. Dickin DC, McClain MA, Hubble RP, Doan JB, Sessford D. Changes in postural sway frequency and complexity in altered sensory environments following whole body vibrations. Human Movement Science. 2012;31(5):1238-46.
190. Park HY, Jung WS, Kim J, Hwang H, Lim K. Changes in the Paradigm of Traditional Exercise in Obesity Therapy and Application of a New Exercise Modality: A Narrative Review Article. IRANIAN JOURNAL OF PUBLIC HEALTH. 2019;48(8):1395-404.
191. Landström U, Lundström R. Changes in wakefulness during exposure to whole body vibration. Electroencephalogr Clin Neurophysiol. 1985;61(5):411-5.
192. Kim W, Park SK, Kang KP, Lee DH, Kim SY, Jung JM, et al. Changes in whole blood viscosity at low shear rates correlate with intravascular volume changes during hemodialysis. Int J Artif Organs. 2012;35(6):425-34.
193. Jung JH, Chae YJ, Lee DH, Cho YI, Ko MM, Park SK, et al. Changes in whole blood viscosity during hemodialysis and mortality in patients with end-stage renal disease. Clin Hemorheol Microcirc. 2017;65(3):285-97.
194. Tofighi A, Saedmocheshi S. (Changes of nerve-muscle performance of elderly men in response to whole body vibration) Journal of Kerman University of Medical Sciences 2014 Mar-May;21(2):130-138. 2014.
195. Chen CH, Liu C, Chuang LR, Chung PH, Shiang TY. Chronic effects of whole-body vibration on jumping performance and body balance using different frequencies and amplitudes with identical acceleration load. J Sci Med Sport. 2014;17(1):107-12.
196. Oroszi T, de Boer SF, Nyakas C, Schoemaker RG, van der Zee EA. Chronic whole body vibration ameliorates hippocampal neuroinflammation, anxiety-like behavior, memory functions and motor performance in aged male rats dose dependently. Scientific Reports. 2022;12(1):9020.
197. Buchanan SR, Miller RM, Nguyen M, Black CD, Kellawan JM, Bemben MG, et al. Circulating microRNA responses to acute whole-body vibration and resistance exercise in postmenopausal women. Frontiers in endocrinology. 2022;13:1038371.
198. Okunribido OO, Shimbles SJ, Magnusson M, Pope M. City bus driving and low back pain: a study of the exposures to posture demands, manual materials handling and whole-body vibration. Appl Ergon. 2007;38(1):29-38.
199. Kapoor S. The clinical benefits of whole body vibration therapy: Beyond its neuromuscular effects. Journal of Sports Medicine and Physical Fitness. 2012;52(1):112.
200. Korosue K, Ishida K, Matsuoka H, Nagao T, Tamaki N, Matsumoto S. Clinical, hemodynamic, and hemorheological effects of isovolemic hemodilution in acute cerebral infarction. Neurosurgery. 1988;23(2):148-53.
201. Liu X, Dong J, Liang Q, Wang HD, Liu Z, Xu R, et al. Coagulant Effects and Mechanism of Schefflera heptaphylla (L.) Frodin. Molecules. 2019;24(24).
202. Ljungberg J, Neely G, Lundström R. Cognitive performance and subjective experience during combined exposures to whole-body vibration and noise. Int Arch Occup Environ Health. 2004;77(3):217-21.
203. Giunta M, Rigamonti AE, Agosti F, Patrizi A, Compri E, Cardinale M, et al. Combination of external load and whole body vibration potentiates the GH-releasing effect of squatting in healthy females. Hormon- und Stoffwechselforschung / Hormones et metabolisme [Hormone and metabolic research]. 2013;45(8):611‐6.
204. Wei QS, Wang HB, Wang JL, Fang B, Zhou GQ, Tan X, et al. Combination treatment with whole body vibration and a kidney-tonifying herbal Fufang prevent osteoporosis in ovariectomized rats. Orthop Surg. 2015;7(1):57-65.
205. Jepsen DB, Ryg J, Jørgensen NR, Hansen S, Masud T, Jørgensen NR. The combined effect of Parathyroid hormone (1-34) and whole-body Vibration exercise in the treatment of OSteoporosis (PaVOS)- study protocol for a randomized controlled trial. Trials. 2018;19:1-.
206. Jepsen DB, Masud T, Holsgaard-Larsen A, Hansen S, Jorgensen NR, Ryg J. The combined effect of parathyroid hormone (1-34) and whole-body vibration exercise on physical performance in OSteoporotic women (PaVOS study): a secondary analysis from a randomised controlled trial. BMC sports science, medicine and rehabilitation. 2020;12(1).
207. Jepsen DB, Ryg J, Hansen S, Jørgensen NR, Gram J, Masud T. The combined effect of Parathyroid hormone (1–34) and whole-body Vibration exercise in the treatment of postmenopausal OSteoporosis (PaVOS study): a randomized controlled trial. Osteoporosis International. 2019;30(9):1827-36.
208. Sobhani S, Sinaei E, Motealleh A, Hooshyar F, Kashkooli NS, Yoosefinejad AK. Combined effects of whole body vibration and unstable shoes on balance measures in older adults: a randomized clinical trial. Archives of Gerontology and Geriatrics 2018 Sep-Oct;78:30-37. 2018.
209. Item F, Denkinger J, Fontana P, Weber M, Boutellier U, Toigo M. Combined Effects of Whole-Body Vibration, Resistance Exercise, and Vascular Occlusion on Skeletal Muscle and Performance. International Journal of Sports Medicine. 2011;32(10):781-7.
210. Yung M, Lang AE, Stobart J, Kociolek AM, Milosavljevic S, Trask C. The combined fatigue effects of sequential exposure to seated whole body vibration and physical, mental, or concurrent work demands. PLoS One. 2017;12(12):e0188468.
211. Fisher J, Van-Dongen M, Sutherland R. Combined isometric and vibration training does not enhance strength beyond that of isometric training alone. Journal of sports medicine and physical fitness. 2015;55(9):899‐904.
212. Merkert J, Butz S, Nieczaj R, Steinhagen-Thiessen E, Eckardt R. Combined whole body vibration and balance training using Vibrosphere: improvement of trunk stability, muscle tone, and postural control in stroke patients during early geriatric rehabilitation. Zeitschrift fur Gerontologie und Geriatrie 2011 Aug;44(4):256-261. 2011.
213. Wong A, Alvarez-Alvarado S, Jaime SJ, Kinsey AW, Spicer MT, Madzima TA, et al. Combined whole-body vibration training and L-citrulline supplementation improves pressure wave reflection in obese postmenopausal women. APPLIED PHYSIOLOGY NUTRITION AND METABOLISM. 2016;41(3):292-7.
214. de Bruin ED, Baur H, Brülhart Y, Luijckx E, Hinrichs T, Rogan S. Combining Stochastic Resonance Vibration With Exergaming for Motor-Cognitive Training in Long-Term Care; A Sham-Control Randomized Controlled Pilot Trial. FRONTIERS IN MEDICINE. 2020;7.
215. Velcheva I, Antonova N, Damianov P, Dimitrov N. Common carotid artery hemodynamic factors in patients with cerebral infarctions. Clin Hemorheol Microcirc. 2010;45(2-4):233-8.
216. Wang CY, Ding HZ, Tang X, Li ZG. Comparative analysis of immune function, hemorheological alterations and prognosis in colorectal cancer patients with different traditional Chinese medicine syndromes. Cancer Biomark. 2018;21(3):701-10.
217. Hamid A, Saleem W, Yaqub G, Ghauri MUD. Comparative assessment of respiratory and other occupational health effects among elementary workers. Int J Occup Saf Ergon. 2019;25(3):394-401.
218. Walsh GS, Delextrat A, Bibbey A. The comparative effect of exercise interventions on balance in perimenopausal and early postmenopausal women: A systematic review and network meta-analysis of randomised, controlled trials. MATURITAS. 2023;175.
219. Irshad A, Abbas T, Sumaira, Ijaz M, Irshad S, Rasheed A, et al. Comparative Effects of Whole-Body Vibration and Squatting Training on Balance and Mobility in Chronic Stroke Patients. A Randomized Controlled Trial. Pakistan journal of medical and health sciences. 2022;16(7):685‐8.
220. Wang ZY, Zan XC, Li YJ, Lu Y, Xia Y, Pan XY. Comparative efficacy different resistance training protocols on bone mineral density in postmenopausal women: A systematic review and network meta-analysis. FRONTIERS IN PHYSIOLOGY. 2023;14.
221. Yuan J, Wu QH, Chen L, Zhang JN, Gu WW. [Comparative studies of hemorheological parameters in Tibet mini-pigs, Beagle dogs and human]. Nan Fang Yi Ke Da Xue Xue Bao. 2009;29(7):1428-32.
222. Palekar TJ, Das AK, Pagare VK. A comparative study between core stabilization and superficial strengthening exercises for the treatment of low back pain in two wheeler riders. International Journal of Pharma and Bio Sciences 2015 Jan;6(1):B168-B176. 2015.
223. Moezy A, Olyaei G, Hadian M, Razi M, Faghihzadeh S. A comparative study of whole body vibration training and conventional training on knee proprioception and postural stability after anterior cruciate ligament reconstruction British Journal of Sports Medicine 2008 May;42(5):373-378. 2008.
224. Irct201012285486N. A comparative study of whole body vibration training and exercise therapy in treating females stress urinary incontinence. https://trialsearchwhoint/Trial2aspx?TrialID=IRCT201012285486N1. 2012.
225. Farzinmehr A, Moezy A, Koohpayehzadeh J, Kashanian M. A comparative study of whole body vibration training and pelvic floor muscle training on women's stress urinary incontinence: three- month follow-up. Journal of family and reproductive health. 2015;9(4):147‐54.
226. Cochrane DJ, Stannard SR, Firth EC, Rittweger J. Comparing muscle temperature during static and dynamic squatting with and without whole-body vibration. Clin Physiol Funct Imaging. 2010;30(4):223-9.
227. Miller RM, Heishman AD, Freitas EDS, Bemben MG. Comparing the acute effects of intermittent and continuous whole-body vibration exposure on neuromuscular and functional measures in sarcopenia and nonsarcopenic elderly women. Dose-Response. 2018;16(3).
228. Yiu EM, Lee SCH. Comparing the Effects of Self-Generated and Platform-Generated Whole Body Vibration on Vocal Fatigue. J Voice. 2023;37(3):332-8.
229. Bazett-Jones DM, Finch HW, Dugan EL. Comparing the effects of various whole-body vibration accelerations on counter-movement jump performance. JOURNAL OF SPORTS SCIENCE AND MEDICINE. 2008;7(1):144-50.
230. Uszynski MK, Purtill H, Donnelly A, Coote S. Comparing the effects of whole-body vibration to standard exercise in ambulatory people with Multiple Sclerosis: a randomised controlled feasibility study. Clinical Rehabilitation. 2016;30(7):657-68.
231. Shadloo N, Kamali F, Salehi Dehno N. A comparison between whole-body vibration and conventional training on pain and performance in athletes with patellofemoral pain. Journal of bodywork and movement therapies. 2021;27:661‐6.
232. Suarez T, Laudani L, Giombini A, Saraceni VM, Mariani PP, Pigozzi F, et al. Comparison in Joint-Position Sense and Muscle Coactivation Between Anterior Cruciate Ligament-Deficient and Healthy Individuals. JOURNAL OF SPORT REHABILITATION. 2016;25(1):64-9.
233. Saldiran T, Mutluay FK, Yagci I, Yilmaz Y. Comparison of aerobic and combined aerobic and whole-body vibration training effects on physical fitness in non-alcoholic fatty liver patients. COMPARATIVE EXERCISE PHYSIOLOGY. 2021;17(4):393-401.
234. Toth A, Sandor B, Marton Z, Kesmarky G, Szabados E, Kehl D, et al. Comparison of hemorheological changes in patients after acute coronary events, intervention and ambulatory rehabilitation. Clin Hemorheol Microcirc. 2016;64(4):565-74.
235. Kang Y, Park S, Lee D, Song S, Choi MR, Lee G. Comparison of lower limb muscle activation according to horizontal whole-body vibration frequency and knee angle. Journal of Human Sport and Exercise. 2020;15(1):119-27.
236. Hoffmann D, Sehmisch S, Hofmann A, Eimer C, Komrakova M, Saul D, et al. Comparison of parathyroid hormone and strontium ranelate in combination with whole-body vibration in a rat model of osteoporosis. Journal of Bone & Mineral Metabolism. 2017;35(1):31-9.
237. Lienhard K, Cabasson A, Meste O, Colson SS. Comparison of sEMG processing methods during whole-body vibration exercise. J Electromyogr Kinesiol. 2015;25(6):833-40.
238. Estes S, Iddings JA, Ray S, Kirk-Sanchez NJ, Field-Fote EC. Comparison of single-session dose response effects of whole body vibration on spasticity and walking speed in persons with spinal cord injury. Neurotherapeutics 2018 Jul;15(3):684-696. 2018.
239. Wyon MA, Smith A, Koutedakis Y. A COMPARISON OF STRENGTH AND STRETCH INTERVENTIONS ON ACTIVE AND PASSIVE RANGES OF MOVEMENT IN DANCERS: A RANDOMIZED CONTROLLED TRIAL. JOURNAL OF STRENGTH AND CONDITIONING RESEARCH. 2013;27(11):3053-9.
240. Hassan Z, Hadian MR, Hussain SA, Shadmehr A, Talebian S, Bagheri H, et al. Comparison of the Conjunct Effects of Electrical Stimulation and Whole-Body Vibration Therapy with Transcranial Direct Current Stimulation and Whole-body Vibration Therapy on Balance and Function in Children With Spastic Cerebral Palsy. Cureus. 2024;16(6):e61511.
241. Lohman EB, Bains GS, Lohman T, DeLeon M, Petrofsky JS. A comparison of the effect of a variety of thermal and vibratory modalities on skin temperature and blood flow in healthy volunteers. MEDICAL SCIENCE MONITOR. 2011;17(9):MT72-MT81.
242. Atalay OT, Yilmaz A, Altinisik G, Cengiz B, Taskin H, Yalman A, et al. Comparison of the effect of aerobic exercise with whole body vibration in patients with bronchiectasis: single blind randomized controlled study. Turkish thoracic journal. 2019;20:S90‐.
243. Mingorance JA, Montoya P, Vivas Miranda JG, Riquelme I. A Comparison of the Effect of Two Types of Whole Body Vibration Platforms on Fibromyalgia. A Randomized Controlled Trial. International journal of environmental research and public health. 2021;18(6).
244. Irct20220713055455N. Comparison of the effect of whole body vibration before and after eccentric exercise on functional markers of delayed onset muscle soreness. https://trialsearchwhoint/Trial2aspx?TrialID=IRCT20220713055455N1. 2022.
245. Guadarrama-Molina E, Barrón-Gámez CE, Estrada-Bellmann I, Meléndez-Flores JD, Ramírez-Castañeda P, Hernández-Suárez RMG, et al. Comparison of the effect of whole-body vibration therapy versus conventional therapy on functional balance of patients with Parkinson's disease: adding a mixed group. Acta Neurol Belg. 2021;121(3):721-8.
246. Park YJ, Park SW, Lee HS. Comparison of the effectiveness of whole body vibration in stroke patients: a meta-analysis. BioMed Research International 2018;(5083634):Epub. 2018.
247. Durgut E, Cahid Orengul A, Algun ZC. Comparison of the effects of treadmill and vibration training in children with attention deficit hyperactivity disorder: a randomized controlled trial. Neurorehabilitation 2020;47(2):121-131. 2020.
248. Ali MS, Awad AS. Comparison of the efficacy of two interventions in ameliorating abdominal thickness and sitting function in children with diplegia. Journal of Taibah University Medical Sciences 2022 Aug;17(4):548-555. 2022.
249. Aminianfar A, Fatemy E, Ghorbani R, Mohamadi R. Comparison of the local and whole body vibration on preventation and treatment of delay-onset muscle soreness: a randomized clinical trial. Koomesh. 2019;21(4):650‐5.
250. Cochrane DI, Sartor F, Winwood K, Stannard SR, Narici MV, Rittweger J. A comparison of the Physiologic effects of acute whole-body vibration exercise in young and older people. ARCHIVES OF PHYSICAL MEDICINE AND REHABILITATION. 2008;89(5):815-21.
251. Marín PJ, Santos-Lozano A, Santin-Medeiros F, Delecluse C, Garatachea N. A comparison of training intensity between whole-body vibration and conventional squat exercise. J Electromyogr Kinesiol. 2011;21(4):616-21.
252. Vorrink SN, Van der Woude LH, Messenberg A, Cripton PA, Hughes B, Sawatzky BJ. Comparison of wheelchair wheels in terms of vibration and spasticity in people with spinal cord injury. J Rehabil Res Dev. 2008;45(9):1269-79.
253. Lohman EB, 3rd, Sackiriyas KS, Bains GS, Calandra G, Lobo C, Nakhro D, et al. A comparison of whole body vibration and moist heat on lower extremity skin temperature and skin blood flow in healthy older individuals. Med Sci Monit. 2012;18(7):Cr415-24.
254. Hand J, Verscheure S, Osternig L. A comparison of whole-body vibration and resistance training on total work in the rotator cuff. Journal of Athletic Training (National Athletic Trainers' Association). 2009;44(5):469-74.
255. Kim YY, Park SE. Comparison of whole-body vibration exercise and plyometric exercise to improve isokinetic muscular strength, jumping performance and balance of female volleyball players. Journal of Physical Therapy Science. 2016;28(11):3140-4.
256. Lai Z, Lee S, Chen Y, Wang L. Comparison of whole-body vibration training and quadriceps strength training on physical function and neuromuscular function of individuals with knee osteoarthritis: a randomised clinical trial. Journal of Exercise Science and Fitness 2021 Jul;19(3):150-157. 2021.
257. Ludat K, Paulitschke M, Riedel E, Hampl H. Complete correction of renal anemia by recombinant human erythropoietin. Clin Nephrol. 2000;53(1 Suppl):S42-9.
258. Pongrácz E, Andrikovics H, Bernát IS, Nagy Z. Connection between genetically determined blood coagulation factors and haemorheology. Clin Hemorheol Microcirc. 2008;39(1-4):333-41.
259. Lo Martire R, de Alwis MP, Äng BO, Garme K. Construction of a web-based questionnaire for longitudinal investigation of work exposure, musculoskeletal pain and performance impairments in high-performance marine craft populations. BMJ Open. 2017;7(7):e016006.
260. Luginbuehl H, Lehmann C, Gerber R, Kuhn A, Hilfiker R, Baeyens JP. Continuous versus intermittent stochastic resonance whole body vibration and its effect on pelvic floor muscle activity. Neurourology and Urodynamics 2012 Jun;31(5):683-687. 2012.
261. Monteleone G, De Lorenzo A, Sgroi M, De Angelis S, Di Renzo L. Contraindications for whole body vibration training: a case of nephrolitiasis. J Sports Med Phys Fitness. 2007;47(4):443-5.
262. Griefahn B, Bröde P, Jaschinski W. Contrast thresholds and fixation disparity during 5-Hz sinusoidal single- and dual-axis (vertical and lateral) whole-body vibration. Ergonomics. 2000;43(3):317-32.
263. Wigley RD, de Groot JA, Walls C. Contribution of vibration to musculoskeletal disorders in New Zealand. Intern Med J. 2007;37(12):822-5.
264. Sercelik A, Besnili AF. The Contribution of Whole Blood Viscosity to the Process of Aortic Valve Sclerosis. Med Princ Pract. 2018;27(2):173-8.
265. Bruyere O, Wuidart MA, di Palma E, Gourlay M, Ethgen O, Richy F, et al. Controlled whole body vibration to decrease fall risk and improve health-related quality of life of nursing home residents. Archives of Physical Medicine and Rehabilitation 2005 Feb;86(2):303-307. 2005.
266. Yang F, King GA, Dillon L, Su X. Controlled whole-body vibration training reduces risk of falls among community-dwelling older adults. Journal of Biomechanics. 2015;48(12):3206-12.
267. Lyons KD, Parks AG, Dadematthews O, Zandieh N, McHenry P, Games KE, et al. Core and whole body vibration exercise influences muscle sensitivity and posture during a military foot march. International Journal of Environmental Research & Public Health 2021 May;18(9):4966. 2021.
268. Lyons KD, Parks AG, Dadematthews OD, Zandieh NL, McHenry PA, Games KE, et al. Core and Whole-Body Vibration Exercise Improve Military Foot March Performance in Novice Trainees: a Randomized Controlled Trial. Military medicine. 2023;188(1‐2):e254‐e9.
269. Arpinar P, Simsek B, Sezgin OC, Birlik G, Korkusuz F. Correlation between mechanical vibration analysis and dual energy X-ray absorptiometry (DXA) in the measurement of in vivo human tibial bone strength. Technology and Health Care. 2005;13(2):107-13.
270. Duarte MLM, de Araújo PA, Horta FC, Vecchio SD, de Carvalho LAP. Correlation between weighted acceleration, vibration dose value and exposure time on whole body vibration comfort levels evaluation. Safety Science. 2018;103:218-24.
271. Domínguez-Muñoz FJ, Carlos-Vivas J, Garcia-Gordillo MA, Villafaina S, Pérez-Escanilla F, Jiménez-Fernández C, et al. Cost-effectiveness of a whole-body vibration program in patients with type 2 diabetes: A retrospective study protocol. Sustainability (Switzerland). 2021;13(5):1-12.
272. Moreira-Marconi E, Dionello CF, Morel DS, Sa-Caputo DC, Souza-Goncalves CR, Paineiras-Domingos LL, et al. Could whole body vibration exercises influence the risk factors for fractures in women with osteoporosis? Osteoporosis and Sarcopenia 2016 Dec;2(4):214-220. 2016.
273. Aydın T, Kesiktaş FN, Baskent A, Karan A, Karacan I, Türker KS. Cross-training effect of chronic whole-body vibration exercise: a randomized controlled study. Somatosensory & motor research. 2020;37(2):51‐8.
274. Sundstrup E, Hansen Å M, Mortensen EL, Poulsen OM, Clausen T, Rugulies R, et al. Cumulative occupational mechanical exposures during working life and risk of sickness absence and disability pension: prospective cohort study. Scand J Work Environ Health. 2017;43(5):415-25.
275. Leite HR, Camargos ACR, Mendonca VA, Lacerda ACR, Soares BA, Oliveira VC. Current evidence does not support whole body vibration in clinical practice in children and adolescents with disabilities: a systematic review of randomized controlled trial. Brazilian Journal of Physical Therapy 2019 May-Jun;23(3):196-211. 2019.
276. Guay A, Houle M, O'Shaughnessy J, Descarreaux M. Current evidence on diagnostic criteria, relevant outcome measures, and efficacy of nonpharmacologic therapy in the management of restless legs syndrome (RLS): a scoping review Journal of Manipulative and Physiological Therapeutics 2020 Nov-Dec;43(9):930-941. 2020.
277. Broniec MN, Norland K, Thomas J, Wang X, Harris RA. The decorin and myostatin response to acute whole body vibration: impact of adiposity, sex, and race. Int J Obes (Lond). 2024;48(12):1803-8.
278. Mitchell UH, Hilton SC, Hunsaker E, Ulfberg J. Decreased Symptoms without Augmented Skin Blood Flow in Subjects with RLS/WED after Vibration Treatment. J Clin Sleep Med. 2016;12(7):947-52.
279. Figueredo Ruiz M, Ferrer Semanat RF, Pérez Mora LY. [Detection of hypovolemia according to Weinreb's criteria in 30 patients previously diagnosed with relative polycythemia]. Sangre (Barc). 1999;44(6):424-8.
280. Lienhard K, Cabasson A, Meste O, Colson SS. Determination of the optimal parameters maximizing muscle activity of the lower limbs during vertical synchronous whole-body vibration. Eur J Appl Physiol. 2014;114(7):1493-501.
281. Pankoke S, Hofmann J, Wölfel HP. Determination of vibration-related spinal loads by numerical simulation. Clinical biomechanics (Bristol, Avon). 2001;16 Suppl 1:S45-56.
282. Lee J, Lee K, Song C. Determining the Posture and Vibration Frequency that Maximize Pelvic Floor Muscle Activity During Whole-Body Vibration. MEDICAL SCIENCE MONITOR. 2016;22:4030-6.
283. Tholen R, Dettmers C, Sailer M, Tallner A, Vaney C, Henze T, et al. Developing clinical practice guidelines for mobility in multiple sclerosis. Multiple Sclerosis Journal. 2019;25(7):1042.
284. Solovieva S, Pehkonen I, Kausto J, Miranda H, Shiri R, Kauppinen T, et al. Development and validation of a job exposure matrix for physical risk factors in low back pain. PLoS One. 2012;7(11):e48680.
285. de Alwis MP, Lo Martire R, Äng BO, Garme K. Development and validation of a web-based questionnaire for surveying the health and working conditions of high-performance marine craft populations. BMJ Open. 2016;6(6):e011681.
286. Jakimovski D, Weinstock-Guttman B, Gandhi S, Guan Y, Hagemeier J, Ramasamy DP, et al. Dietary and lifestyle factors in multiple sclerosis progression: results from a 5-year longitudinal MRI study. J Neurol. 2019;266(4):866-75.
287. Kat CJ, Gräbe RP, van Staden PJ, Botha T, Els PS. Difference thresholds for primary and secondary ride of a vehicle on a 4-poster test rig. Ergonomics. 2024;67(11):1702-14.
288. Vieira SS, Lemes B, Silva JA, Jr., Bocalini DS, Suzuki FS, Albertini R, et al. Different land-based exercise training programs to improve bone health in postmenopausal women. Medical Science and Technology 2013 Dec 3;54:158-163. 2013.
289. Peungsuwan P, Chatchawan U, Donpunha W, Malila P, Sriboonreung T. Different protocols for low whole-body vibration frequency for spasticity and physical performance in children with spastic cerebral palsy. Children 2023 Feb;10(3):458. 2023.
290. Cai J, Shao X, Yan ZD, Liu XY, Yang YQ, Luo EP, et al. Differential skeletal response in adult and aged rats to independent and combinatorial stimulation with pulsed electromagnetic fields and mechanical vibration. FASEB JOURNAL. 2020;34(2):3037-50.
291. Gold JE, Punnett L, Cherniack M, Wegman DH. Digital vibration threshold testing and ergonomic stressors in automobile manufacturing workers: a cross-sectional assessment. Ergonomics. 2005;48(1):66-77.
292. Huang Y, Li J. Discomfort estimation for vertical whole-body vibration in the aircraft cabin considering the duration and static sitting comfort. Ergonomics. 2024;67(3):327-38.
293. Huang Y, Griffin MJ. The discomfort produced by noise and whole-body vertical vibration presented separately and in combination. Ergonomics. 2014;57(11):1724-38.
294. Schwarze S, Blome O, Notbohm G. [Disk-related diseases of the lumbar spine as an example for the critical interaction between clinical diagnosis and occupational disease]. Orthopade. 2002;31(10):957-72.
295. Bedient AM, Adams JB, Edwards DA, Serravite DH, Huntsman E, Mow SE, et al. Displacement and frequency for maximizing power output resulting from a bout of whole-body vibration. Journal of Strength and Conditioning Research. 2009;23(6):1683-7.
296. Messing K, Tissot F, Stock S. Distal lower-extremity pain and work postures in the Quebec population. Am J Public Health. 2008;98(4):705-13.
297. Wehrle E, Wehner T, Heilmann A, Bindl R, Claes L, Jakob F, et al. Distinct frequency dependent effects of whole-body vibration on non-fractured bone and fracture healing in mice. J Orthop Res. 2014;32(8):1006-13.
298. Gómez-Bruton A, González-Agüero A, Matute-Llorente A, Julián C, Lozano-Berges G, Gómez-Cabello A, et al. Do 6 months of whole-body vibration training improve lean mass and bone mass acquisition of adolescent swimmers? ARCHIVES OF OSTEOPOROSIS. 2017;12(1).
299. Twitchett EA, Angioi M, Koutedakis Y, Wyon M. Do increases in selected fitness parameters affect the aesthetic aspects of classical ballet performance? Medical problems of performing artists. 2011;26(1):35‐8.
300. de Araújo AXP, de Araújo MDR, Fontes LAM, Braz RRS, Sañudo B, Taiar R, et al. Do two whole-body vibration amplitudes improve postural balance, gait speed, muscle strength, and functional mobility in sedentary older women? A crossover randomized controlled trial. JOURNAL OF BODYWORK AND MOVEMENT THERAPIES. 2022;32:143-8.
301. Dionello CF, De Souza PL, Sá-Caputo D, Morel DS, Moreira-Marconi E, Paineiras-Domingos LL, et al. Do whole body vibration exercises affect lower limbs neuromuscular activity in populations with a medical condition? A systematic review. Restorative Neurology and Neuroscience. 2017;35(6):667-81.
302. Frissen I, Guastavino C. Do whole-body vibrations affect spatial hearing? Ergonomics. 2014;57(7):1090-101.
303. Bordenstein SR, Werren JH. Do Wolbachia influence fecundity in Nasonia vitripennis? Heredity (Edinb). 2000;84 ( Pt 1):54-62.
304. Gómez-Cabello A, González-Agüero A, Ara I, Casajús JA, Vicente-Rodríguez G. DOES A WHOLE BODY VIBRATION INTERVENTION HAVE ANY EFFECT ON ADIPOSITY IN ELDERLY PEOPLE? EUROPEAN JOURNAL OF HUMAN MOVEMENT. 2016;36:36-47.
305. Salmon JR, Roper JA, Tillman MD. Does acute whole-body vibration training improve the physical performance of people with knee osteoarthritis. Journal of Strength and Conditioning Research. 2012;26(11):2983-9.
306. Noorloos D, Tersteeg L, Tiemessen IJ, Hulshof CT, Frings-Dresen MH. Does body mass index increase the risk of low back pain in a population exposed to whole body vibration? Appl Ergon. 2008;39(6):779-85.
307. Milosavljevic S, Bagheri N, Vasiljev RM, McBride DI, Rehn B. Does daily exposure to whole-body vibration and mechanical shock relate to the prevalence of low back and neck pain in a rural workforce? Ann Occup Hyg. 2012;56(1):10-7.
308. Simsek D. DOES EMG ACTIVATION DIFFER AMONG FATIGUE-RESISTANT LEG MUSCLES DURING DYNAMIC WHOLE-BODY VIBRATION? SOUTH AFRICAN JOURNAL FOR RESEARCH IN SPORT PHYSICAL EDUCATION AND RECREATION. 2016;38(1):149-65.
309. Calendo LR, Taeymans J, Rogan S. Does Muscle Activation during Whole-Body Vibration Induce Bone Density Improvement in Postmenopausal Women? - A Systematic Review. Sportverletzung-Sportschaden. 2014;28(3):125-31.
310. Yule CE, Stoner L, Hodges LD, Cochrane DJ. Does short-term whole-body vibration training affect arterial stiffness in chronic stroke? A preliminary study. Journal of Physical Therapy Science 2016 Mar;28(3):996-1002. 2016.
311. Rowe PL, Taflan S, Hahne AJ. Does the Addition of Whole-Body Vibration Training Improve Postural Stability and Lower Limb Strength during Rehabilitation Following Anterior Cruciate Ligament Reconstruction: A Systematic Review with Meta-analysis. Clinical Journal of Sport Medicine. 2022;32(6):627-34.
312. Parsons J, Mathieson S, Jull A, Parsons M. Does vibration training reduce the fall risk profile of frail older people admitted to a rehabilitation facility? A randomised controlled trial. DISABILITY AND REHABILITATION. 2016;38(11):1082-8.
313. Nct. Does Vitamin D Alter Bone's Response to Vibration? https://clinicaltrialsgov/show/NCT02743559. 2016.
314. Santos JM, Mendonça VA, Ribeiro VGC, Tossige-Gomes R, Fonseca SF, Prates ACN, et al. Does whole body vibration exercise improve oxidative stress markers in women with fibromyalgia? Brazilian Journal of Medical and Biological Research. 2019;52(8).
315. Alavinia SM, Omidvar M, Craven BC. Does whole body vibration therapy assist in reducing fat mass or treating obesity in healthy overweight and obese adults? A systematic review and meta-analyses. DISABILITY AND REHABILITATION. 2021;43(14):1935-47.
316. De Jong J, Frick V, Kuhn A, De Bruin E. Does whole body vibration training help in a geriatric population suffering from urinary incontinence? International Urogynecology Journal and Pelvic Floor Dysfunction. 2011;22:S958-S9.
317. Maia TO, Paiva DN, Sobral Filho DC, Cavalcanti FCB, Rocha LG, Andrade CCA, et al. Does whole body vibration training improve heart rate variability in kidney transplants patients? A randomized clinical trial. Journal of Bodywork and Movement Therapies 2020 Apr;24(2):50-56. 2020.
318. Fernandes IA, Kawchuk G, Bhambhani Y, Gomes PSC. Does whole-body vibration acutely improve power performance via increased short latency stretch reflex response? Journal of Science and Medicine in Sport. 2013;16(4):360-4.
319. Zhang J, Yu J, Tang X, Yang F, Kang Y, Zhang C, et al. Does whole-body vibration have benefits in patients with multiple sclerosis: a systematic review and meta-analysis. International Journal of Clinical and Experimental Medicine 2017;10(7):9996-10009. 2017.
320. Amorim NTS, Cavalcanti FCB, Moura E, Sobral Filho D, Leitão CCS, Almeida MM, et al. Does whole-body vibration improve risk of falls, balance, and heart rate variability in post-COVID-19 patients? A randomized clinical trial. J Bodyw Mov Ther. 2024;39:518-24.
321. Cardim AB, Marinho PE, Nascimento JF, Jr., Fuzari HK, Dornelas de Andrade A. Does Whole-Body Vibration Improve the Functional Exercise Capacity of Subjects With COPD? A Meta-Analysis. Respir Care. 2016;61(11):1552-9.
322. Harijanto C, Lim A, Vogrin S, Duque G. Does Whole-Body Vibration Training Have a Concurrent Effect on Bone and Muscle Health? A Systematic Review and Meta-Analysis. GERONTOLOGY. 2022;68(6):601-11.
323. Yin Y, Wang J, Yu Z, Zhou L, Liu X, Cai H, et al. Does whole-body vibration training have a positive effect on balance and walking function in patients with stroke? A meta-analysis. Frontiers in Human Neuroscience. 2023;16.
324. Carlucci F, Mazzà C, Cappozzo A. Does whole-body vibration training have acute residual effects on postural control ability of elderly women? Journal of Strength and Conditioning Research. 2010;24(12):3363-8.
325. Zha DS, Zhu QA, Pei WW, Zheng JC, Wu SH, Xu ZX, et al. Does whole-body vibration with alternative tilting increase bone mineral density and change bone metabolism in senior people? Aging Clinical and Experimental Research 2012 Feb;24(1):28-36. 2012.
326. Jakimovski D, Weinstock-Guttman B, Burnham A, Weinstock Z, Wicks TR, Ramanathan M, et al. Dynamic disability measures decrease the clinico-radiological gap in people with severely affected multiple sclerosis. Mult Scler Relat Disord. 2024;87:105630.
327. Hsiao YH, Chien SH, Tu HP, Fu JCM, Tsai ST, Chen YS, et al. Early post-operative intervention of whole-body vibration in patients after total knee arthroplasty: A pilot study. Journal of Clinical Medicine. 2019;8(11).
328. Stark C, Herkenrath P, Hollmann H, Waltz S, Becker I, Hoebing L, et al. Early vibration assisted physiotherapy in toddlers with cerebral palsy -- a randomized controlled pilot trial. Journal of Musculoskeletal & Neuronal Interactions 2016 Sep;16(3):183-192. 2016.
329. Castillo-Bueno I, Ramos-Campo DJ, Rubio-Arias JA. Efectos del entrenamiento vibratorio de cuerpo completo en pacientes con esclerosis multiple: una revision sistematica (Effects of whole-body vibration training in patients with multiple sclerosis: a systematic review) Neurologia 2018 Oct;33(8):534-548. 2018.
330. Boggild M, Erlandson M, Tomlinson G, Szabo E, Slatkovska L, Giangregorio L, et al. Effect of 1 year of whole-body vibration therapy on muscle density and volume in postmenopausal women: the vibration study. Journal of bone and mineral research. 2013;28.
331. Actrn. The effect of 3 vibration types on pelvic floor muscles activity. https://trialsearchwhoint/Trial2aspx?TrialID=ACTRN12618000531213. 2018.
332. Tantawy SA, Elgohary HMI, Abdelbasset WK, Kamel DM. Effect of 4 weeks of whole-body vibration training in treating stress urinary incontinence after prostate cancer surgery: a randomised controlled trial. Physiotherapy 2019 Sep;105(3):338-345. 2019.
333. Torvinen S, Sievänen H, Järvinen TAH, Pasanen M, Kontulainen S, Kannus P. Effect of 4-min vertical whole body vibration on muscle performance and body balance: A randomized cross-over study. International Journal of Sports Medicine. 2002;23(5):374-9.
334. Nct. The Effect of 6 Months of Local Vibration Training in Institutionalized Elderly. https://clinicaltrialsgov/show/NCT01499186. 2011.
335. Lai CL, Tseng SY, Chen CN, Liao WC, Wang CH, Lee MC, et al. Effect of 6 months of whole body vibration on lumbar spine bone density in postmenopausal women: a randomized controlled trial. Clinical Interventions in Aging 2013 Dec 4;8:1603-1609. 2013.
336. van den Tillaar R. The effect of 6 months of whole body vibration training on strength in postmenopausal women. Motricidade. 2012;8:41-50.
337. Rubio-Arias JA, Esteban P, Martínez F, Ramos-Campo DJ, Mendizábal S, Berdejo-del-Fresno D, et al. Effect of 6 weeks of whole body vibration training on total and segmental body composition in healthy young adults. ACTA PHYSIOLOGICA HUNGARICA. 2015;102(4):442-50.
338. Verschueren SMP, Roelants M, Delecluse C, Swinnen S, Vanderschueren D, Boonen S. Effect of 6-month whole body vibration training on hip density, muscle strength, and postural control in postmenopausal women: A randomized controlled pilot study. Journal of Bone and Mineral Research. 2004;19(3):352-9.
339. Sañudo B, de Hoyo M, Carrasco L, McVeigh JG, Corral J, Cabeza R, et al. The effect of 6-week exercise programme and whole body vibration on strength and quality of life in women with fibromyalgia: a randomised study. Clinical and experimental rheumatology. 2010;28(6 Suppl 63):S40‐5.
340. Dallas G, Savvathi A, Dallas K, Maridaki M. The effect of 6-weeks whole body vibration on muscular performance on young non-competitive female artistic gymnasts. Science of Gymnastics Journal. 2019;11(2):151-62.
341. Santin-Medeiros F, Santos-Lozano A, Cristi-Montero C, Garatachea Vallejo N. Effect of 8 months of whole-body vibration training on quality of life in elderly women. Research in sports medicine (Print). 2017;25(1):101‐7.
342. Beck BR, Norling TL. The effect of 8 mos of twice-weekly low- or higher intensity whole body vibration on risk factors for postmenopausal hip fracture. American Journal of Physical Medicine & Rehabilitation 2010 Dec;89(12):997-1009. 2010.
343. Torvinen S, Kannus P, Sievänen H, Järvinen TAH, Pasanen M, Kontulainen S, et al. Effect of 8-Month Vertical Whole Body Vibration on Bone, Muscle Performance, and Body Balance: A Randomized Controlled Study. Journal of Bone and Mineral Research. 2003;18(5):876-84.
344. Sievänen H, Piirtola M, Tokola K, Kulmala T, Tiirikainen E, Kannus P, et al. Effect of 10-Week Whole-Body Vibration Training on Falls and Physical Performance in Older Adults: A Blinded, Randomized, Controlled Clinical Trial with 1-Year Follow-Up. Int J Environ Res Public Health. 2024;21(7).
345. Slatkovska L, Alibhai SMH, Beyene J, Hu HX, Demaras A, Cheung AM. Effect of 12 Months of Whole-Body Vibration Therapy on Bone Density and Structure in Postmenopausal Women A Randomized Trial. ANNALS OF INTERNAL MEDICINE. 2011;155(10):668-+.
346. Marin-Cascales E, Rubio-Arias JA, Romero-Arenas S, Alcaraz PE. Effect of 12 weeks of whole-body vibration versus multi-component training in post-menopausal women. Rejuvenation research. 2015;18(6):508‐16.
347. Zheng YL, Wang XF, Chen BL, Gu W, Wang X, Xu B, et al. Effect of 12-week whole-body vibration exercise on lumbopelvic proprioception and pain control in young adults with nonspecific low back pain. Medical Science Monitor. 2019;25:443-52.
348. Osugi T, Iwamoto J, Yamazaki M, Takakuwa M. Effect of a combination of whole body vibration exercise and squat training on body balance, muscle power, and walking ability in the elderly. Therapeutics & Clinical Risk Management. 2014;10:131-8.
349. Alfadhel SAA, Vennu V, Alotaibi AD, Algarni AM, Bindawas SMS. The effect of a multicomponent exercise programme onelderly adults' risk of falling in nursing homes: A systematic review. JOURNAL OF THE PAKISTAN MEDICAL ASSOCIATION. 2020;70(4):699-704.
350. Unger M, Jelsma J, Stark C. Effect of a trunk-targeted intervention using vibration on posture and gait in children with spastic type cerebral palsy: A randomized control trial. Developmental Neurorehabilitation. 2013;16(2):79-88.
351. Torvinen S, Kannus P, Sievänen H, Järvinen TAH, Pasanen M, Kontulainen S, et al. Effect of a vibration exposure on muscular performance and body balance. Randomized cross-over study. Clinical Physiology and Functional Imaging. 2002;22(2):145-52.
352. Lee JH, Kim SB, Lee KW, Lee SJ, Park H, Kim DW. The effect of a whole-body vibration therapy on the sitting balance of subacute stroke patients: a randomized controlled trial. Topics in Stroke Rehabilitation 2017;24(6):457-462. 2017.
353. Martínez-Pardo E, Romero-Arenas S, Martínez-Ruiz E, Rubio-Arias JA, Alcaraz PE. Effect of a whole-body vibration training modifying the training frequency of workouts per week in active adults. Journal of strength and conditioning research / National Strength & Conditioning Association. 2014;28(11):3255-63.
354. Alentorn-Geli E, Moras G, Padilla J, Fernandez-Sola J, Bennett RM, Lazaro-Haro C, et al. Effect of acute and chronic whole-body vibration exercise on serum insulin-like growth factor-1 levels in women with fibromyalgia. Journal of Alternative & Complementary Medicine 2009 May;15(5):573-578. 2009.
355. Cochrane DJ. The Effect of Acute Vibration Exercise on Short-Distance Sprinting and Reactive Agility. JOURNAL OF SPORTS SCIENCE AND MEDICINE. 2013;12(3):497-501.
356. Betts E, Smith J, Wischmeyer S, Betts J. Effect of acute, whole-body vibration on an isometric one repetition maximum biceps curl. Physiotherapy (United Kingdom). 2011;97:eS127.
357. Bokaeian HR, Bakhtiary AH, Mirmohammadkhani M, Moghimi J. The effect of adding whole body vibration training to strengthening training in the treatment of knee osteoarthritis: a randomized clinical trial. Journal of bodywork and movement therapies. 2016;20(2):334‐40.
358. Topaloğlu M, Ketenci A, Baslo B, Şahinkaya T. The effect of adding whole-body vibration exercises to home exercise program on muscle strength in patients with post-polio syndrome. Turkish Journal of Physical Medicine & Rehabilitation (2587-1250). 2022;68(1):117-25.
359. Avelar NCP, Simão AP, Tossige-Gomes R, Neves CDC, Rocha-Vieira E, Coimbra CC, et al. The Effect of Adding Whole-Body Vibration to Squat Training on the Functional Performance and Self-Report of Disease Status in Elderly Patients with Knee Osteoarthritis: A Randomized, Controlled Clinical Study. Journal of Alternative & Complementary Medicine. 2011;17(12):1149-55.
360. Lai Z, Lee S, Hu X, Wang L. Effect of adding whole-body vibration training to squat training on physical function and muscle strength in individuals with knee osteoarthritis. Journal of musculoskeletal & neuronal interactions. 2019;19(3):333‐41.
361. Trimble A, Zeman K, Wu J, Ceppe A, Bennett W, Donaldson S. Effect of airway clearance therapies on mucociliary clearance in adults with cystic fibrosis: a randomized controlled trial. PLoS ONE 2022 May;17(5):e0268622. 2022.
362. Jurik R, Zebrowska A, Stastny P. Effect of an Acute Resistance Training Bout and Long-Term Resistance Training Program on Arterial Stiffness: A Systematic Review and Meta-Analysis. JOURNAL OF CLINICAL MEDICINE. 2021;10(16).
363. Ochi A, Abe T, Yamada K, Ibuki S, Tateuchi H, Ichihashi N. Effect of balance exercise in combination with whole-body vibration on muscle activity of the stepping limb during a forward fall in older women: a randomized controlled pilot study. Archives of gerontology and geriatrics. 2015;60(2):244‐51.
364. Karacan I, Sariyildiz MA, Bahadir C, Özen A. Effect of Bone Tissue on Vibration-Induced Electrical Activity of Muscles. TURKIYE KLINIKLERI TIP BILIMLERI DERGISI. 2010;30(4):1346-56.
365. Saggini R, Ancona E, Carmignano SM, Supplizi M, Barassi G, Bellomo RG. Effect of combined treatment with focused mechano-acoustic vibration and pharmacological therapy on bone mineral density and muscle strength in post-menopausal women. CLINICAL CASES IN MINERAL AND BONE METABOLISM. 2017;14(3):305-11.
366. Tupimai T, Peungsuwan P, Prasertnoo J, Yamauchi J. Effect of combining passive muscle stretching and whole body vibration on spasticity and physical performance of children and adolescents with cerebral palsy. Journal of Physical Therapy Science 2016 Jan;28(1):7-13. 2016.
367. Deng XG, Chen JT, Feng Y. Effect of compound vibration on bone quality of ovariectomized rats. Journal of Clinical Rehabilitative Tissue Engineering Research. 2010;14(2):196-200.
368. Wang XX, Wang LJ, Wu Y, Cai M, Wang LY. Effect of Different Exercise Interventions on Grip Strength, Knee Extensor Strength, Appendicular Skeletal Muscle Index, and Skeletal Muscle Index Strength in Patients with Sarcopenia: A Meta-Analysis of Randomized Controlled Trials. DISEASES. 2024;12(4).
369. Dabbs NC, Muñoz CX, Tran TT, Brown LE, Bottaro M. Effect of different rest intervals after whole-body vibration on vertical jump performance. Journal of strength and conditioning research. 2011;25(3):662‐7.
370. Dixit S, Gular K, Asiri F. Effect of diverse physical rehabilitative interventions on static postural control in diabetic peripheral neuropathy: a systematic review. Physiotherapy Theory and Practice 2020;36(6):679-690. 2020.
371. Fu CLA, Yung SHP, Law KYB, Leung KHH, Lui PYP, Siu HK, et al. The Effect of Early Whole-Body Vibration Therapy on Neuromuscular Control After Anterior Cruciate Ligament Reconstruction: A Randomized Controlled Trial. American Journal of Sports Medicine. 2013;41(4):804-14.
372. Januszyk D, Schafer E, Thompson HJ, Sargent B. Effect of Exercise and Motor Interventions on Physical Activity and Motor Outcomes of Adults with Cerebral Palsy: A Systematic Review. Developmental Neurorehabilitation. 2023;26(6-7):389-412.
373. Kistler-Fischbacher M, Weeks BK, Beck BR. The effect of exercise intensity on bone in postmenopausal women (part 2): A meta-analysis. BONE. 2021;143.
374. Zhang KD, Wang LY, Zhang ZH, Zhang DX, Lin XW, Meng T, et al. Effect of Exercise Interventions on Health-Related Quality of Life in Patients with Fibromyalgia Syndrome: A Systematic Review and Network Meta-Analysis. JOURNAL OF PAIN RESEARCH. 2022;15:3639-56.
375. Kang SR, Kim GW, Ko MH, Han KS, Kwon TK. The effect of exercise load deviations in whole body vibration on improving muscle strength imbalance in the lower limb. Technology and Health Care. 2020;28(S1):S103-S14.
376. Cerrillo-Urbina AJ, García-Hermoso A, Sánchez-López M, Martínez-Vizcaíno V. Effect of exercise programs on symptoms of fibromyalgia in peri-menopausal age women: A systematic review and meta-analysis of randomized controlled trials. MYOPAIN. 2015;23(1-2):56-70.
377. Cole KJ, Mahoney SE. Effect of five weeks of whole body vibration training on speed, power, and flexibility. Clinical Kinesiology. 2010;64(1):1-7.
378. Flores JG, Liang MT, Metchkoff TG, Chen E, Auslander AT, Rocha J. Effect of four weeks whole-body vibration training on bone mineral density and bending strength in young adults. FASEB journal Conference: experimental biology 2016, EB San diego, CA united states Conference start: 20160402 Conference end: 20160406 Conference publication: (varpagings). 2016;30(no pagination).
379. Torvinen S, Kannus P, Sievänen H, Järvinen TAH, Pasanen M, Kontulainen S, et al. Effect of four-month vertical whole body vibration on performance and balance. Medicine & Science in Sports & Exercise. 2002;34(9):1523-8.
380. Rhea MR, Bunker D, Marin PJ, Lunt K. Effect of iTonic whole-body vibration on delayed-onset muscle soreness among untrained individuals Journal of Strength & Conditioning Research 2009 Sep;23(6):1677-1682. 2009.
381. Vissers D, Verrijken A, Mertens I, van Gils C, van de Sompel A, Truijen S, et al. Effect of long-term whole body vibration training on visceral adipose tissue: a preliminary report. Obesity Facts 2010 Apr;3(2):93-100. 2010.
382. Rajapakse CS, Johncola AJ, Batzdorf AS, Jones BC, Al Mukaddam M, Sexton K, et al. Effect of Low-Intensity Vibration on Bone Strength, Microstructure, and Adiposity in Pre-Osteoporotic Postmenopausal Women: A Randomized Placebo-Controlled Trial. JOURNAL OF BONE AND MINERAL RESEARCH. 2021;36(4):673-84.
383. Beck B, Rubin C, Harding A, Paul S, Forwood M. The effect of low-intensity whole-body vibration with or without high-intensity resistance and impact training on risk factors for proximal femur fragility fracture in postmenopausal women with low bone mass: study protocol for the VIBMOR randomized controlled trial. Trials. 2022;22(1):1-19.
384. Chen GX, Zheng S, Qin S, Zhong ZM, Wu XH, Huang ZP, et al. Effect of Low-Magnitude Whole-Body Vibration Combined with Alendronate in Ovariectomized Rats: A Random Controlled Osteoporosis Prevention Study. PLOS ONE. 2014;9(5).
385. Mehta S, McClarren B, Aijaz A, Chalaby R, Cook-Chennault K, Olabisi RM. The effect of low-magnitude, high-frequency vibration on poly(ethylene glycol)-microencapsulated mesenchymal stem cells. JOURNAL OF TISSUE ENGINEERING. 2018;9.
386. Hornik B, Dulawa J, Marcisz C, Korchut W, Durmala J. The Effect of Mechanically-Generated Vibrations on the Efficacy of Hemodialysis; Assessment of Patients' Safety: Preliminary Reports. INTERNATIONAL JOURNAL OF ENVIRONMENTAL RESEARCH AND PUBLIC HEALTH. 2019;16(4).
387. Lee H, Kim S, Hwang D, Seo D, Kim D, Jung YJ, et al. The effect of multi-frequency whole-body vibration on night-shifted mouse model. SLEEP AND BIOLOGICAL RHYTHMS. 2018;16(4):387-98.
388. Tan TW, Tan HL, Hsu MF, Huang HL, Chung YC. Effect of non-pharmacological interventions on the prevention of sarcopenia in menopausal women: a systematic review and meta-analysis of randomized controlled trials. BMC WOMENS HEALTH. 2023;23(1).
389. Matsumoto Y, Griffin MJ. Effect of phase on discomfort caused by vertical whole-body vibration and shock - Experimental investigation. Journal of the Acoustical Society of America. 2002;111(3):1280-8.
390. Wanderley FS, Alburquerque-Sendín F, Parizotto NA, Rebelatto JR. Effect of Plantar Vibration Stimuli on the Balance of Older Women: A Randomized Controlled Trial. ARCHIVES OF PHYSICAL MEDICINE AND REHABILITATION. 2011;92(2):199-206.
391. Aksoy D, Erman A, Balci N, Sentürk Ü. Effect of protein intake on muscle strength and hypertrophy during whole-body vibration training. ISOKINETICS AND EXERCISE SCIENCE. 2017;25(4):235-42.
392. Ebid A, El-Shamy S, Thabet A, El-boshy M, Abedalla M, Ali T. Effect of pulsed electromagnetic field versus pulsed high intensity laser in the treatment of men with osteopenia or osteoporosis: a randomized controlled trial. F1000Research 2022 Jan 24;11(86):Epub. 2022.
393. Tamartash H, Bahrpeyma F, Dizaji MM. The Effect of Remote Myofascial Release on Chronic Nonspecific Low Back Pain With Hamstrings Tightness. JOURNAL OF SPORT REHABILITATION. 2023;32(5):549-56.
394. Jones GC, Blotter JD, Smallwood CD, Eggett DL, Cochrane DJ, Feland JB. Effect of resonant frequency vibration on delayed onset muscle soreness and resulting stiffness as measured by shear-wave elastography. International Journal of Environmental Research and Public Health. 2021;18(15).
395. Legerlotz K, Schjerling P, Langberg H, Brüggemann G-P, Niehoff A. The effect of running, strength, and vibration strength training on the mechanical, morphological, and biochemical properties of the Achilles tendon in rats. Journal of Applied Physiology (Bethesda, Md : 1985). 2007;102(2):564-72.
396. Elhosary EA, ElAzab DR, Hanoura EM, Othman EM. Effect of six weeks of whole-body vibration in treatment of postnatal constipation. JOURNAL OF THE PAKISTAN MEDICAL ASSOCIATION. 2023;73(4):S13-S6.
397. Rathi MA, Joshi R, Desai R, Gazbare P, Kulkarni N, Balid M. Effect of squat training with whole body vibration on balance and functional performance in elderly- a quasi-experimental study. Journal of Clinical and Diagnostic Research. 2021;15(10):YC11-YC4.
398. Park SH, Oh YJ, Seo JH, Lee MM. Effect of stabilization exercise combined with respiratory resistance and whole body vibration on patients with lumbar instability: a randomized controlled trial. Medicine 2022 Nov 18;101(46):e31843. 2022.
399. Mikhael M, Orr R, Amsen F, Greene D, Singh MAF. Effect of standing posture during whole body vibration training on muscle morphology and function in older adults: a randomised controlled trial. BMC Geriatrics. 2010;10:74-.
400. Kessler J, Radlinger L, Baur H, Rogan S. Effect of stochastic resonance whole body vibration on functional performance in the frail elderly: a pilot study. Archives of gerontology and geriatrics. 2014;59(2):305‐11.
401. Escobar-Alvarez JA, Jiménez-Reyes P, Da Conceiçao FA, Fuentes-García JP. Effect of Supplementary Physical Training on Vertical Jump Height in Professional Ballet Dancers. INTERNATIONAL JOURNAL OF SPORTS PHYSIOLOGY AND PERFORMANCE. 2022;17(8):1257-63.
402. Shamseddini Sofla F, Hadadi M, Rezaei I, Azhdari N, Sobhani S. The effect of the combination of whole body vibration and shoe with an unstable surface in chronic ankle instability treatment: a randomized clinical trial. BMC sports science, medicine and rehabilitation. 2021;13(1).
403. Kurosaka S, Takaura M, Karakawa T, Kadomasu H, Deguchi T, Yanaoka T. The effect of the Hiroshima GENKI Exercise on the bone strength and cognitive function in elderly people. Journal of science and medicine in sport. 2022;25:S27‐.
404. Hallal CZ, Marques NR, Spinoso DH, Cirqueira RT, Morcelli MH, Crozara LF, et al. EFFECT OF THE VIBRATORY POLE TRAINING ON BIOMECHANICS OF DUAL-TASK GAIT IN OLDER FEMALE ADULTS. REVISTA BRASILEIRA DE MEDICINA DO ESPORTE. 2014;20(6):465-9.
405. Escudero-Uribe S, Hochsprung A, Heredia-Camacho B, Izquierdo-Ayuso G. Effect of training exercises incorporating mechanical devices on fatigue and gait pattern in persons with relapsing-remitting multiple sclerosis. Physiotherapy Canada 2017 Fall;69(4):292-302. 2017.
406. kxkzn RBR. Effect of training in vibrating plataform in subjects with Chronic Obstructive Pulmonary Disease. https://trialsearchwhoint/Trial2aspx?TrialID=RBR-3kxkzn. 2015.
407. Choi SJ, Shin WS, Oh BK, Shim JK, Bang DH. Effect of training with whole body vibration on the sitting balance of stroke patients. Journal of Physical Therapy Science 2014 Sep;26(9):1411-1414. 2014.
408. Tseng SY, Hsu PS, Lai CL, Liao WC, Lee MC, Wang CH. Effect of two frequencies of whole-body vibration training on balance and flexibility of the elderly: a randomized controlled trial. American Journal of Physical Medicine & Rehabilitation 2016 Oct;95(10):730-737. 2016.
409. Ali MS, Awad AS, Elassal MI. The effect of two therapeutic interventions on balance in children with spastic cerebral palsy: a comparative study. Journal of Taibah University Medical Sciences 2019 Aug;14(4):350-356. 2019.
410. Niewiadomski W, Strasz A, Mróz A, Laskowska D, Langfort J, Gasiorowska A. Effect of type of static exercise and whole body vibration frequency on oxygen consumption and respiratory activity in young and middle-aged women. ARCHIVES OF BUDO. 2015;11:259-64.
411. Kramer M, Kholvadia A. The effect of vibration cycle ergometry on pulmonary <i>(V) over dotO</i><sub>2</sub> kinetics, isokinetic kneetorque, and lower extremity explosive power. ERGONOMICS. 2021;64(7):943-52.
412. Lacerda AC, Oliveira AC, Mendonça V, Santos LM, Fonseca SF, Santos JM, et al., editors. Effect of vibration exercise in the modified push-up position on hand neural efficiency in rheumatoid arthritis: Preliminary results. Advances in Intelligent Systems and Computing; 2020.
413. Sands WA, McNeal JR, Stone MH, Kimmel WL, Haff GG, Jemni M. The effect of vibration on active and passive range of motion in elite female synchronized swimmers. EUROPEAN JOURNAL OF SPORT SCIENCE. 2008;8(4):217-23.
414. Ko CY, Lee TW, Woo DG, Namgung BS, Kim HS, Lee BY, et al. Effect of vibration on lumbar bone of OVX rats compared with risedronate-dosed rat. 2006 INTERNATIONAL CONFERENCE ON BIOMEDICAL AND PHARMACEUTICAL ENGINEERING, VOLS 1 AND 22006. p. 201-+.
415. Yu CH, Seo SB, Kang SR, Kim K, Kwon TK. Effect of vibration on muscle strength imbalance in lower extremity using multi-control whole body vibration platform. Bio-medical materials and engineering. 2015;26 Suppl 1:S673‐83.
416. d8nf RBR. Effect of vibration stimulus in fibromyalgia women. http://wwwwhoint/trialsearch/Trial2aspx?TrialID=RBR-36d8nf. 2016.
417. Yavuz SC. Effect of Vibration Training on Body Composition and Flexibility in Healthy and Sedentary Women. 6TH EUROPEAN SPORTS MEDICINE CONGRESS (EFSMA)2009. p. 141-4.
418. ChiCtr. Effect of vibration training vs. conventional resistance training on sarcopenia among community-dwelling older people with sarcopenia: 3-arm randomized controlled trial protocol. https://trialsearchwhoint/Trial2aspx?TrialID=ChiCTR2100051178. 2021.
419. Kelly SB, Alvar BA, Black LE, Dodd DJ, Carothers KF, Brown LE. The effect of warm-up with whole-body vibration vs. cycle ergometry on isokinetic dynamometry. Journal of strength and conditioning research. 2010;24(11):3140‐3.
420. Kim H, Kwon BS, Park JW, Lee H, Nam K, Park T, et al. Effect of whole body horizontal vibration exercise in chronic low back pain patients: vertical versus horizontal vibration exercise. Annals of Rehabilitation Medicine 2018 Dec;42(6):804-813. 2018.
421. Martín G, De Saa Y, Da Silva-Grigoletto ME, Vaamonde D, Sarmiento S, García-Manso JM. Effect of whole body vibration (WBV) on PTH in elderly subjects. Revista Andaluza de Medicina del Deporte. 2009;2(1):1-6.
422. Lam TP, Ng BKW, Cheung LWH, Lee KM, Qin L, Cheng JCY. Effect of whole body vibration (WBV) therapy on bone density and bone quality in osteopenic girls with adolescent idiopathic scoliosis: a randomized, controlled trial. OSTEOPOROSIS INTERNATIONAL. 2013;24(5):1623-36.
423. Shim C, Lee Y, Lee D, Jeong B, Kim J, Choi Y, et al. Effect of Whole Body Vibration Exercise in the Horizontal Direction on Balance and Fear of Falling in Elderly People: A Pilot Study. JOURNAL OF PHYSICAL THERAPY SCIENCE. 2014;26(7):1083-6.
424. Jepsen DB, Thomsen K, Hansen S, Jørgensen NR, Masud T, Ryg J. The effect of whole body vibration exercise on fracture risk in adults over 50 years of age: A systematic review and meta-analysis. Osteoporosis International. 2017;28:S530-S1.
425. Trans T, Aaboe J, Henriksen M, Christensen R, Bliddal H, Lund H. Effect of whole body vibration exercise on muscle strength and proprioception in females with knee osteoarthritis. The Knee. 2009;16(4):256‐61.
426. von Stengel S, Kemmler W, Mayer S, Engelke K, Klarner A, Kalender WA. Effect of whole body vibration exercise on osteoporotic risk factors. Deutsche medizinische Wochenschrift (1946). 2009;134(30):1511‐6.
427. Orr R. The effect of whole body vibration exposure on balance and functional mobility in older adults: a systematic review and meta-analysis. Maturitas 2015 Apr;80(4):342-358. 2015.
428. Mikhael M, Orr R, Singh MAF. The effect of whole body vibration exposure on muscle or bone morphology and function in older adults: A systematic review of the literature. MATURITAS. 2010;66(2):150-7.
429. Giombini A, Menotti F, Laudani L, Piccinini A, Fagnani F, Di Cagno A, et al. Effect of whole body vibration frequency on neuromuscular activity in ACL-deficient and healthy males. BIOLOGY OF SPORT. 2015;32(3):243-7.
430. Wunderer K, Schabrun SM, Chipchase LS. The effect of whole body vibration in common neurological conditions -- a systematic review. Physical Therapy Reviews 2008;13(6):434-442. 2008.
431. Arias P, Chouza M, Vivas J, Cudeiro J. Effect of whole body vibration in Parkinson's disease: a controlled study. Movement Disorders 2009 Apr 30;24(6):891-898. 2009.
432. Yang X, Wang P, Liu C, He C, Reinhardt JD. The effect of whole body vibration on balance, gait performance and mobility in people with stroke: a systematic review and meta-analysis. Clinical Rehabilitation 2015 Jul;29(7):627-638. 2015.
433. Lam FMH, Lau RWK, Chung RCK, Pang MYC. The effect of whole body vibration on balance, mobility and falls in older adults: a systematic review and meta-analysis. Maturitas 2012 Jul;72(3):206-213. 2012.
434. Actrn. The effect of Whole Body Vibration on balance, muscle strength and falls in older persons living in residential care. http://wwwwhoint/trialsearch/Trial2aspx?TrialID=ACTRN12609000201279. 2009.
435. Nct. Effect of Whole Body Vibration on Bone and Fall Related Parameters. https://clinicaltrialsgov/show/NCT00292916. 2006.
436. Telli Atalay O, Yilmaz A, Altinişik G, Cengiz B, Taşkin H, Yalman A, et al. Effect of whole body vibration on exercise capacity in patients with bronchiectasis. Turkish Journal of Physiotherapy and Rehabilitation. 2019;30(2):S102.
437. Li G, Zhang G, Wang Y, Wang X, Zhou H, Li H, et al. The effect of whole body vibration on health-related quality of life in patients with chronic conditions: a systematic review. Quality of Life Research 2019 Nov;28(11):2859-2870. 2019.
438. Kesvatara T, Bagheri R, Khani MM, Heidari Z, Sekandari R, Pahlevan AA. The effect of whole body vibration on isokinetic torque parameters of ankle muscles during running before and after fatigue in age groups during growth and adults. Koomesh. 2023;25(4):527-34.
439. Kang SR, Min JY, Yu C, Kwon TK. Effect of whole body vibration on lactate level recovery and heart rate recovery in rest after intense exercise. TECHNOLOGY AND HEALTH CARE. 2017;25:S115-S23.
440. Ebid AA, Ahmed MT, Mahmoud Eid M, Mohamed MSE. Effect of whole body vibration on leg muscle strength after healed burns: A randomized controlled trial. Burns. 2012;38(7):1019-26.
441. Tan JW, Wu XP. (Effect of whole body vibration on low extremity functions and chronic diseases in the elderly) Chinese Journal of Tissue Engineering Research 2017 Mar 18;21(8):1288-1293. 2017.
442. Lohman EB, Petrofsky JS, Maloney-Hinds C, Betts-Schwab H, Thorpe D. The effect of whole body vibration on lower extremity skin blood flow in normal subjects. Medical science monitor. 2007;13(2):CR71‐6.
443. Pollock RD, Martin FC, Newham DJ. The effect of whole body vibration on older people: a systematic review. PHYSICAL THERAPY REVIEWS. 2012;17(2):110-23.
444. Ayvat E, Kılınç M, Ayvat F, Onursal Kılınç Ö, Aksu Yıldırım S. The Effect of Whole Body Vibration on Postural Control of Ataxic Patients: a Randomized Controlled Cross-Over Study. Cerebellum (London, England). 2021;20(4):533‐41.
445. Tan J, Wu X, Clark CCT, Barton V, Chen S, Liu S, et al. The effect of whole body vibration on sensorimotor deficits in people with chronic ankle instability: a systematic review and meta-analysis. Clinical Rehabilitation 2022 Aug;36(8):1016-1031. 2022.
446. Kemmler W, von Stengel S, Mayer S, Niedermayer M, Hentschke C, Kalender WA. Effect of whole body vibration on the neuromuscular performance of females 65 years and older. One-year results of the controlled randomized ELVIS study. ZEITSCHRIFT FUR GERONTOLOGIE UND GERIATRIE. 2010;43(2):125-32.
447. Min SK, Lee K, Lim ST. The effect of whole body vibration on the sprint ability of Korean national bobsled and skeleton athletes. PLOS ONE. 2021;16(10).
448. Zendeh Del A, Hedayati R, Bakhtiary A, Mirmohammadkhani M, Hajihasani A. The Effect of Whole Body Vibration on Torque of Evertor and Invertor Muscles of Ankle in Low Arched Feet. Journal of mazandaran university of medical sciences. 2014;24(118):189‐204.
449. Vissers D, Baeyens JP, Truijen S, Ides K, Vercruysse CC, van Gaal L. The effect of whole body vibration short-term exercises on respiratory gas exchange in overweight and obese women. The Physician and Sportsmedicine 2009 Oct;37(3):88-94. 2009.
450. Wei QS, Huang L, Chen XH, Wang HB, Sun WS, Huo SC, et al. Effect of whole body vibration therapy on circulating serotonin levels in an ovariectomized rat model of osteoporosis. Iranian Journal of Basic Medical Sciences. 2014;17(1):62-8.
451. Isrctn. The effect of whole body vibration therapy on older people. https://trialsearchwhoint/Trial2aspx?TrialID=ISRCTN19205068. 2010.
452. Lee JH, Hong MW. The effect of whole body vibration therapy on the lower extremity function in subacute stroke patients. International journal of stroke. 2020;15(1 SUPPL):185‐.
453. Zhang J, Zhang H, Kan L, Zhang C, Wang P. The effect of whole body vibration therapy on the physical function of people with type II diabetes mellitus: a systematic review. Journal of Physical Therapy Science 2016 Sep;28(9):2675-2680. 2016.
454. Matute-Llorente A, Gonzalez-Aguero A, Gomez-Cabello A, Olmedillas H, Vicente-Rodriguez G, Casajus JA. Effect of whole body vibration training on bone mineral density and bone quality in adolescents with Down syndrome: a randomized controlled trial. Osteoporosis international. 2015;26(10):2449‐59.
455. Eid MA, Aly SM. Effect of whole body vibration training on bone mineral density and functional capacity in children with thalassemia. Physiotherapy Theory and Practice 2021;37(2):279-286. 2021.
456. el-Shamy SM, Mohamed MSE. Effect of whole body vibration training on bone mineral density in cerebral palsy children. Indian Journal of Physiotherapy and Occupational Therapy 2012 Jan-Mar;6(1):139-141. 2012.
457. Myung-Sook K, Jung-Hee D, Jeong-Soo K, Hye-Seon J. Effect of whole body vibration training on gait function and activities of daily living in children with cerebral palsy. International Journal of Therapy & Rehabilitation. 2015;22(7):321-8.
458. Annino G, Padua E, Castagna C, di Salvo V, Minichella S, Tsarpela O, et al. Effect of whole body vibration training on lower limb performance in selected high-level ballet students Journal of Strength & Conditioning Research 2007 Nov;21(4):1072-1076. 2007.
459. Lee BK, Chon SC. Effect of whole body vibration training on mobility in children with cerebral palsy: a randomized controlled experimenter-blinded study. CLINICAL REHABILITATION. 2013;27(7):599-607.
460. Anwer S, Alghadir A, Zafar H, al-Eisa E. Effect of whole body vibration training on quadriceps muscle strength in individuals with knee osteoarthritis: a systematic review and meta-analysis. Physiotherapy 2016 Jun;102(2):145-151. 2016.
461. Wei N, Ng GYF. The effect of whole body vibration training on quadriceps voluntary activation level of people with age-related muscle loss (sarcopenia): a randomized pilot study. BMC geriatrics. 2018;18(1):240.
462. Sade I, Cekmece C, Inanir M, Dursun N. The effect of whole body vibration treatment on upper extremity functions compromised by stroke. Noro Psikiatri Arsivi [Archives of Neuropsychiatry] 2021 Sep;58(3):189-192. 2021.
463. Nct. Effect of Whole Body Vibration Versus Core Stability Exercises on Balance and Muscle Strength in Children With Down Syndrome. https://clinicaltrialsgov/ct2/show/NCT06036069. 2023.
464. Ahmadizadeh Z, Khalili MA, Ghalam MS, Mokhlesin M. Effect of whole body vibration with stretching exercise on active and passive range of motion in lower extremities in children with cerebral palsy: a randomized clinical trial. Iranian Journal of Pediatrics 2019;29(5):1-7. 2019.
465. Hanif H, Orooj M, Parveen A. Effect of whole-body vibration after a resistance exercise bout on heart rate variability in hypertensive population. Journal of Complementary & Integrative Medicine 2022 Jun;19(2):435-439. 2022.
466. Kawanabe K, Kawashima A, Sashimoto I, Takeda T, Sato Y, Iwamoto J. Effect of whole-body vibration exercise and muscle strengthening, balance, and walking exercises on walking ability in the elderly. Keio Journal of Medicine. 2007;56(1):28-33.
467. Jepsen DB, Thomsen K, Hansen S, Jorgensen NR, Masud T, Ryg J. Effect of whole-body vibration exercise in preventing falls and fractures: a systematic review and meta-analysis. BMJ OPEN. 2017;7(12).
468. de Oliveira Guedes-Aguiar E, da Cunha de Sá-Caputo D, Moreira-Marconi E, de Macêdo Uchôa SM, de Barros PZ, Valentin EK, et al. Effect of whole-body vibration exercise in the pelvic floor muscles of healthy and unhealthy individuals: A narrative review. Translational Andrology and Urology. 2019;8(4):395-404.
469. Sañudo B, de Hoyo M, Carrasco L, Rodríguez-Blanco C, Oliva-Pascual-Vaca Á, McVeigh JG. Effect of Whole-Body Vibration Exercise on Balance in Women with Fibromyalgia Syndrome: A Randomized Controlled Trial. Journal of Alternative & Complementary Medicine. 2012;18(2):158-64.
470. Hegazy RG, Abdel-aziem AA. Effect of Whole-Body Vibration Exercise on Hamstrings-to-Quadriceps Ratio, Walking Performance, and Postural Control in Children With Hemiparetic Cerebral Palsy: A Randomized Controlled Trial. Journal of Manipulative & Physiological Therapeutics. 2022;45(9):660-70.
471. Zhang L, Weng C, Liu M, Wang Q, Liu L, He Y. Effect of whole-body vibration exercise on mobility, balance ability and general health status in frail elderly patients: a pilot randomized controlled trial. Clinical rehabilitation. 2014;28(1):59‐68.
472. Shengqin C, Zhongfang W, Chongyang L, Qiaoli W, Cao S, Wang Z, et al. The effect of whole-body vibration exercise on postmenopausal women with osteoporosis: A protocol for systematic review and meta-analysis. Medicine. 2021;100(18):1-5.
473. ElDeeb AM, Abdel-Aziem AA. Effect of Whole-Body Vibration Exercise on Power Profile and Bone Mineral Density in Postmenopausal Women With Osteoporosis: A Randomized Controlled Trial. Journal of Manipulative & Physiological Therapeutics. 2020;43(4):384-93.
474. Lai CL, Chen HY, Tseng SY, Liao WC, Liu BT, Lee MC, et al. Effect of whole-body vibration for 3 months on arterial stiffness in the middle-aged and elderly. Clinical Interventions in Aging 2014 May 12;9:821-828. 2014.
475. Krol P, Piecha M, Slomka K, Sobota G, Polak A, Juras G. The effect of whole-body vibration frequency and amplitude on the myoelectric activity of vastus medialis and vastus lateralis. JOURNAL OF SPORTS SCIENCE AND MEDICINE. 2011;10(1):169-74.
476. Muanjai P, Haas C, Sies W, Mittag U, Zange J, Schönau E, et al. Effect of Whole-body Vibration frequency on muscle tensile state during graded plantar flexor isometric contractions. Journal of Exercise Science & Fitness. 2023;21(4):405-15.
477. Irct20090301001722N. Effect of whole-body vibration in children with cerebral palsy. https://trialsearchwhoint/Trial2aspx?TrialID=IRCT20090301001722N27. 2023.
478. He Z, Zheng J, Liu S, Guan Z, Zhou Q, Jin X, et al. The effect of whole-body vibration in osteopenic patients after total knee arthroplasty: a randomized controlled trial. Aging Clinical & Experimental Research. 2022;34(6):1381-90.
479. Ali MS, Abd el-aziz HG. Effect of whole-body vibration on abdominal thickness and sitting ability in children with spastic diplegia. Journal of taibah university medical sciences. 2021;16(3):379‐86.
480. Sucuoglu H, Tuzun S, Akbaba YA, Uludag M, Gokpinar HH. Effect of Whole-Body Vibration on Balance Using Posturography and Balance Tests in Postmenopausal Women. American journal of physical medicine & rehabilitation. 2015;94(7):499‐507.
481. Slatkovska L, Alibhai SM, Beyene J, Cheung AM. Effect of whole-body vibration on BMD: a systematic review and meta-analysis. Osteoporosis International 2010 Dec;21(12):1969-1980. 2010.
482. Slatkovska L, Beyene J, Alibhai SMH, Wong Q, Sohail QZ, Cheung AM. Effect of Whole-Body Vibration on Calcaneal Quantitative Ultrasound Measurements in Postmenopausal Women: A Randomized Controlled Trial. CALCIFIED TISSUE INTERNATIONAL. 2014;95(6):547-56.
483. Liang H, Beerse M, Ke X, Wu J. Effect of whole-body vibration on center-of-mass movement during standing in children and young adults. Gait & Posture. 2017;54:148-53.
484. Hossam A, Amin FS, Abutaleb EE. Effect of whole-body vibration on craniovertebral angle and balance control in forward head posture: Single-blinded randomized controlled trial. Fizjoterapia Polska. 2021;21(1):98-104.
485. Wheeler AA, Jacobson AH. Effect of whole-body vibration on delayed onset muscular soreness, flexibility, and power. 2013. p. 2527-32.
486. Dincher A, Becker P, Wydra G. Effect of whole-body vibration on freezing and flexibility in Parkinson's disease -- a pilot study. Neurological Sciences 2021 Jul;42(7):2795-2801. 2021.
487. Marshall LC, Wyon MA. The effect of whole-body vibration on jump height and active range of movement in female dancers. Journal of strength and conditioning research. 2012;26(3):789‐93.
488. Lindsay KG, Nichols DL, Davis RW, Marshall DD. The Effect of Whole-Body Vibration on Lower-Body Resistance Detraining in College-Age Women. Research Quarterly for Exercise & Sport. 2018;89(1):57-65.
489. Mohamed El-Shamy S. Effect of Whole-Body Vibration on Muscle Strength and Balance in Diplegic Cerebral Palsy. American Journal of Physical Medicine & Rehabilitation. 2014;93(2):114-21.
490. El-Shamy SM. Effect of whole-body vibration on muscle strength and balance in diplegic cerebral palsy: a randomized controlled trial. American journal of physical medicine & rehabilitation. 2014;93(2):114‐21.
491. Ibrahim MM, Eid MA, Moawd SA. Effect of whole-body vibration on muscle strength, spasticity, and motor performance in spastic diplegic cerebral palsy children. Egyptian journal of medical human genetics. 2014;15(2):173‐9.
492. Wang Z, Wei Z, Li X, Lai Z, Wang L. Effect of whole-body vibration on neuromuscular activation and explosive power of lower limb: A systematic review and meta-analysis. PLoS ONE. 2022;17(12 December).
493. Liao LR, Pang MYC. Effect of Whole-Body Vibration on Neuromuscular Activation of Leg Muscles During Dynamic Exercises in Individuals With Stroke. Journal of strength and conditioning research. 2017;31(7):1954-62.
494. von Stengel S, Kemmler W, Engelke K, Kalender WA. Effect of whole-body vibration on neuromuscular performance and body composition for females 65 years and older: a randomized-controlled trial. Scandinavian Journal of Medicine & Science in Sports. 2012;22(1):119-27.
495. Alam MM, Khan AA, Farooq M. Effect of whole-body vibration on neuromuscular performance: A literature review. Work. 2018;59(4):571-83.
496. Ahmed Burq HSI, Karimi H, Ahmad A, Gilani SA, Hanif A. Effect of whole-body vibration on obstacle clearance and stair negotiation time in chronic stroke patients; a randomized controlled trial. Journal of Bodywork and Movement Therapies 2021 Jul;27:698-704. 2021.
497. Ewertowska P, Formella O, Poniatowski L, Zielińska A, Krzysztofik M, Czaprowski D. Effect of whole-body vibration on postural stability in young adults with generalized joint hypermobility: A comparative study. Journal of Back and Musculoskeletal Rehabilitation. 2024;1(1).
498. Li KY, Cho YJ, Chen RS. The effect of whole-body vibration on proprioception and motor function for individuals with moderate Parkinson disease: a single-blind randomized controlled trial. Occupational Therapy International 2021;(9441366):Epub. 2021.
499. Ma CY, Liu A, Sun M, Zhu HX, Wu HB. Effect of whole-body vibration on reduction of bone loss and fall prevention in postmenopausal women: a meta-analysis and systematic review. JOURNAL OF ORTHOPAEDIC SURGERY AND RESEARCH. 2016;11.
500. Pamukoff DN, Montgomery MM, Choe KH, Moffit TJ, Vakula MN. Effect of Whole-Body Vibration on Sagittal Plane Running Mechanics in Individuals With Anterior Cruciate Ligament Reconstruction: A Randomized Crossover Trial. ARCHIVES OF PHYSICAL MEDICINE AND REHABILITATION. 2018;99(5):973-80.
501. Piotrowska A, Gattner H, Adamiak J, Metel S, Czerwinska-Ledwig O, Pilch W, et al. Effect of Whole-Body Vibration on Serum Levels of Brain Derived Neurotrophic Factor and Cortisol in Young, Healthy Women. International journal of environmental research and public health. 2022;19(23).
502. Yurttutmuş ZR, Zincirci DE, Bardak AN, Karacan İ, Türker KS. The effect of whole-body vibration on spasticity in post-stroke hemiplegia: A prospective, randomized-controlled study. Turkish Journal of Physical Medicine & Rehabilitation (2587-1250). 2022;68(4):484-92.
503. Almeida HC, Lage VKS, Taiar R, Santos JM, Paula FA, Rapin A, et al. Effect of whole-body vibration stimulation on plasma soluble TNF receptors in elderly with sarcopenia: a randomized controlled trial. Brazilian journal of medical and biological research = revista brasileira de pesquisas medicas e biologicas. 2024;57:e13282.
504. Luo XT, Zhang JF, Zhang C, He CQ, Wang P. The effect of whole-body vibration therapy on bone metabolism, motor function, and anthropometric parameters in women with postmenopausal osteoporosis. DISABILITY AND REHABILITATION. 2017;39(22):2315-23.
505. Matute-Llorente A, González-Agüero A, Gómez-Cabello A, Vicente-Rodríguez G, Mallén JAC. Effect of Whole-Body Vibration Therapy on Health-Related Physical Fitness in Children and Adolescents With Disabilities: A Systematic Review. JOURNAL OF ADOLESCENT HEALTH. 2014;54(4):385-96.
506. Jones MT, Parker BM, Cortes N. The effect of whole-body vibration training and conventional strength training on performance measures in female athletes. Journal of strength and conditioning research. 2011;25(9):2434‐41.
507. Kalfon R, Wong A, Madzima TA, Figueroa A. The Effect of Whole-Body Vibration Training on Arterial Stiffness, Blood Pressure, and Muscle Strength in Obese Postmenopausal Women. MEDICINE AND SCIENCE IN SPORTS AND EXERCISE. 2013;45(5):619-.
508. Lin CI, Huang WC, Chen WC, Kan NW, Wei L, Chiu YS, et al. Effect of whole-body vibration training on body composition, exercise performance and biochemical responses in middle-aged mice. METABOLISM-CLINICAL AND EXPERIMENTAL. 2015;64(9):1146-56.
509. Matute-Llorente A, Gonzalez-Aguero A, Gomez-Cabello A, Tous-Fajardo J, Vicente-Rodriguez G, Casajus JA. Effect of whole-body vibration training on bone mass in adolescents with and without Down syndrome: a randomized controlled trial. Osteoporosis International 2016 Jan;27(1):181-191. 2016.
510. Chen H, Ma J, Lu B, Ma XL. The effect of whole-body vibration training on lean mass. Medicine (United States). 2017;96(45).
511. Rubio-Arias JA, Marin-Cascales E, Ramos-Campo DJ, Martinez-Rodriguez A, Chung LH, Alcaraz PE. The effect of whole-body vibration training on lean mass in postmenopausal women: a systematic review and meta-analysis. Menopause 2017 Feb;24(2):225-231. 2017.
512. Hengting C, Jianxiong M, Bin L, Xin-long M, Chen H, Ma J, et al. The effect of whole-body vibration training on lean mass: A PRISMA-compliant meta-analysis. Medicine. 2017;96(45):1-8.
513. Zhang J, Wang R, Zheng Y, Xu J, Wu Y, Wang X. Effect of whole-body vibration training on muscle activation for individuals with knee osteoarthritis. BioMed Research International. 2021;2021.
514. Tofighi A, Dastah S, Babaei S, Nozad J. (Effect of whole-body vibration training on physiological indices and cardiovascular fitness in elderly veterans). Scientific Journal of Kurdistan University of Medical Sciences 2014 Spring;19(1):12-20. 2014.
515. Nawrat-Szoltysik A, Sieradzka M, Nowacka-Chmielewska M, Piejko L, Duda J, Brachman A, et al. Effect of Whole-Body Vibration Training on Selected Intrinsic Risk Factors in Women Aged 60+ at Fall Risk: a Randomized Controlled Trial. International journal of environmental research and public health. 2022;19(24).
516. Eid MA. Effect of Whole-Body Vibration Training on Standing Balance and Muscle Strength in Children with Down Syndrome. American Journal of Physical Medicine & Rehabilitation. 2015;4(8):633-43.
517. Abbasi E, Kahrizi S, Razi M, Faghihzadeh S. The effect of whole-body vibration training on the lower extremity muscles' electromyographic activities in patients with knee osteoarthritis Medical Journal of the Islamic Republic of Iran 2017 Dec 18;31(107):Epub. 2017.
518. Rodriguez-Reyes G, Garcia-Ulloa AC, Hernandez-Jimenez S, Alessi-Montero A, Nunez Carrera L, Rojas-Torres F, et al. Effect of whole-body vibration training on transcutaneous oxygen levels of the foot in patients with type 2 diabetes: a randomized controlled trial. Journal of Biomechanics 2022 Jun;139:110871. 2022.
519. Haleva Y, Dunsky A, Rubinstein M, Kleinöder H, Mester J. Effect of whole-body vibration training with two different vibration amplitudes on lower limb performance. German Journal of Exercise and Sport Research. 2023;53(2):155-62.
520. Dabbs NC, Brown LE, Coburn JW, Lynn SK, Biagini MS, Tran TT. Effect of whole-body vibration warm-up on bat speed in women softball players. Journal of strength and conditioning research. 2010;24(9):2296‐9.
521. Nct. Effect of Whole-body Vibration With or Without Localized Radiofrequency on Body Composition of Obese Female Subjects. https://clinicaltrialsgov/show/NCT01329328. 2011.
522. Tallon G, Ramdani S, Jaussent A, Decker L, Bernard PL, Blain H. Effect of whole-body-vibration training in institutionalized older adults. European geriatric medicine. 2013;4:S55.
523. Yoon J, Fujii K, Seol J, Isoda H, Okura T. Effect of whole-body-vibration with maslinic-acid on knee strength and pain in elderly with knee osteoarthritis. Archives of physical medicine and rehabilitation. 2017;98(10):e59‐e60.
524. Umin. The effect toward dynamic stability with Whole Body Vibration Training for healthy subjects. https://trialsearchwhoint/Trial2aspx?TrialID=JPRN-UMIN000032908. 2018.
525. Kordi Yoosefinejad A, Shadmehr A, Olyaei G, Talebian S, Bagheri H. The effectiveness of a single session of Whole-Body Vibration in improving the balance and the strength in type 2 diabetic patients with mild to moderate degree of peripheral neuropathy: A pilot study. Journal of Bodywork and Movement Therapies. 2014;18(1):82-6.
526. Berner K, Albertyn SCS, Dawnarain S, Hendricks LJ, Johnson J, Landman A, et al. The effectiveness of combined lower limb strengthening and whole-body vibration, compared to strengthening alone, for improving patient-centred outcomes in adults with COPD: A systematic review. South African Journal of Physiotherapy. 2020;76(1):1-12.
527. Dabbs NC, Lundahl JA, Garner JC. Effectiveness of different rest intervals following whole-body vibration on vertical jump performance between college athletes and recreationally trained females. Sports. 2015;3(3):258-68.
528. Lawrence H, Hills S, Kline N, Weems K, Doty A. Effectiveness of exercise on functional mobility in adults with cerebral palsy: a systematic review. Physiotherapy Canada 2016 Fall;68(4):398-407. 2016.
529. Benedetti MG, Furlini G, Zati A, Mauro GL. The Effectiveness of Physical Exercise on Bone Density in Osteoporotic Patients. BIOMED RESEARCH INTERNATIONAL. 2018;2018.
530. Moreau NG, Winter Bodkin A, Bjornson K, Hobbs A, Soileau M, Lahasky K. Effectiveness of Rehabilitation Interventions to Improve Gait Speed in Children With Cerebral Palsy: Systematic Review and Meta-analysis. Physical Therapy. 2016;96(12):1938-54.
531. Nipa SI, Sriboonreung T, Paungmali A, Phongnarisorn C. Effectiveness of therapeutic interventions for women with urinary incontinence: a systematic review. Critical Reviews in Physical & Rehabilitation Medicine 2020;32(1):1-22. 2020.
532. Ctri. Effectiveness of Whole body vibrator training for toning up the muscles in urine control. https://trialsearchwhoint/Trial2aspx?TrialID=CTRI/2019/07/020300. 2019.
533. Tseng SY, Lai CL, Ko CP, Chang YK, Fan HC, Wang CH. The effectiveness of whole-body vibration and heat therapy on the muscle strength, flexibility, and balance abilities of elderly groups. International Journal of Environmental Research & Public Health 2023 Jan;20(2):1650. 2023.
534. Braz RRS, Campos SL, Villela DW, Antonino GB, Batista PKA, Guerino MR, et al. Effectiveness of Whole-Body Vibration Combined with Multicomponent Training on the Risk of Falls and Quality of Life in Elderly Women with Osteoporosis: Study Protocol for a Randomized Controlled Clinical Trial. Biology. 2022;11(2).
535. Karacay BC, Sahbaz T, Gurtekin B, Yildiz S, Ozcan E. Effectiveness of whole-body vibration exercise and core stabilization exercise in chronic non-specific low back pain: A randomized-controlled study. Turkish Journal of Physical Medicine & Rehabilitation (2587-1250). 2022;68(2):184-94.
536. Han YG, Kim MK. Effectiveness of whole-body vibration in patients with cerebral palsy: A systematic review and meta-analysis. Medicine (United States). 2023;102(48):E36441.
537. de Oliveira RDJ, de Oliveira RG, de Oliveira LC, Santos SD, Sá-Caputo DC, Bernardo M. Effectiveness of whole-body vibration on bone mineral density in postmenopausal women: a systematic review and meta-analysis of randomized controlled trials. OSTEOPOROSIS INTERNATIONAL. 2023;34(1):29-52.
538. Dabbagh A, Sarvestani FK. The effectiveness of whole-body vibration on the attenuation of delayed-onset muscle soreness in healthy untrained individuals: a randomised controlled trial. Comparative Exercise Physiology. 2022;18(5):427-35.
539. Necdet Ardic F, Alkan H, Tumkaya F, Ardic F. Effectiveness of whole-body vibration or biofeedback postural training as an add-on to vestibular exercises rehabilitation therapy in chronic unilateral vestibular weakness: a randomized controlled study. Journal of Vestibular Research 2021;31(3):181-190. 2021.
540. Jo NG, Kang SR, Ko MH, Yoon JY, Kim HS, Han KS, et al. Effectiveness of whole-body vibration training to improve muscle strength and physical performance in older adults: prospective, single-blinded, randomized controlled trial. Healthcare 2021 Jun;9(6):652. 2021.
541. Beaudart C, Maquet D, Mannarino M, Buckinx F, Demonceau M, Crielaard JM, et al. Effects of 3 months of short sessions of controlled whole body vibrations on the risk of falls among nursing home residents. BMC geriatrics. 2013;13:42.
542. Claerbout M, Gebara B, Ilsbroukx S, Verschueren S, Peers K, Van Asch P, et al. Effects of 3 weeks' whole body vibration training on muscle strength and functional mobility in hospitalized persons with multiple sclerosis. Multiple sclerosis (Houndmills, Basingstoke, England). 2012;18(4):498‐505.
543. Koczulla R, Koelpin J, Kaufhold F, Vogelmeier C, Herth FJF, Boeselt T, et al. Effects of 3-months whole-body vibration training on muscle strength, lung function parameters, health-related quality of life and markers of inflammation in patients with interstitial lung disease at three different centers for lung research (DZL) in Germany. American Journal of Respiratory and Critical Care Medicine. 2017;195.
544. Marín-Cascales E, Del Cerro N, Alcaraz PE, Rubio-Arias JA, editors. Effects of 6 months of multicomponent training vs. WBV on strength in postmenopausal women. Cultura, Ciencia y Deporte; 2014.
545. Miyachi R, Kanazawa Y, Fujii Y, Kitagawa T, Yamazaki T. Effects of 6 weeks of whole-body vibration training on ankle motor control: a randomized controlled trial. J Sports Med Phys Fitness. 2024;64(7):676-84.
546. Beck BR, Norling TL. Effects of 8 months of twice-weekly high versus low intensity whole body vibration on risk factors for hip fracture in postmenopausal women: a randomized controlled trial. Bone. 2009;44:S70‐S1.
547. Eider J, Mishchenko V, Tomiak T, Sawczyn S, Kuehne T, Zasada M. Effects of 8-week intermittent whole body vibration combined with sub-maximal resistance training on strength capacities in health-related training of young females. Polish Journal of Environmental Studies. 2011;20(6):1453-64.
548. Esmaeilzadeh S, Akpinar M, Polat SE, Yildiz A, Oral A. Effects of 8-week whole-body vibration training on knee extensors strength in healthy young volunteers. Osteoarthritis and Cartilage. 2015;23:A392.
549. Dominguez-Munoz FJ, Villafaina S, Garcia-Gordillo MA, Hernandez-Mocholi MA, Collado-Mateo D, Adsuar JC, et al. Effects of 8-week whole-body vibration training on the HbA1c, quality of life, physical fitness, body composition and foot health status in people with T2DM: a double-blinded randomized controlled trial. International Journal of Environmental Research & Public Health 2020 Feb;17(4):1317. 2020.
550. de Ruiter CJ, van Raak SM, Schilperoort JV, Hollander AP, de Haan A. The effects of 11 weeks whole body vibration training on jump height, contractile properties and activation of human knee extensors. European Journal of Applied Physiology. 2003;90(5-6):595-600.
551. de Souza HCM, Pessoa MF, Clemente RDS, da Silva AV, Cardoso PRG, Fernandes J, et al. Effects of 12 weeks of inspiratory muscle training and whole body vibration on the inflammatory profile, BDNF and muscular system in pre-frail elderly women: a randomized controlled trial. Archives of gerontology and geriatrics. 2024;123.
552. Lopes-Souza P, Dionello CF, Bernardes-Oliveira CL, Moreira-Marconi E, Marchon RM, Teixeira-Silva Y, et al. Effects of 12-week whole-body vibration exercise on fatigue, functional ability and quality of life in women with systemic lupus erythematosus: A randomized controlled trial. JOURNAL OF BODYWORK AND MOVEMENT THERAPIES. 2021;27:191-9.
553. Leung KS, Li CY, Tse YK, Choy TK, Leung PC, Hung VWY, et al. Effects of 18-month low-magnitude high-frequency vibration on fall rate and fracture risks in 710 community elderly-a cluster-randomized controlled trial. OSTEOPOROSIS INTERNATIONAL. 2014;25(6):1785-95.
554. Gusso S, Vesey RM, Derraik JGB, Munns CF, Colle P, Biggs JB, et al. The Effects of 20 Weeks of Side-Alternating Vibration Therapy on Physical Function, Bone and Muscle Health in Adolescents with Down Syndrome. Physical & Occupational Therapy in Pediatrics. 2021;41(1):44-55.
555. Roelants M, Delecluse C, Goris M, Verschueren S. Effects of 24 weeks of whole body vibration training on body composition and muscle strength in untrained females. International Journal of Sports Medicine. 2004;25(1):1-5.
556. Marín-Cascales E, Alcaraz PE, Rubio-Arias JA, Marín-Cascales E. Effects of 24 Weeks of Whole Body Vibration Versus Multicomponent Training on Muscle Strength and Body Composition in Postmenopausal Women: A Randomized Controlled Trial. Rejuvenation Research. 2017;20(3):193-201.
557. Jimenez Siebert M, Boselt T, Greulich T, Alter P, Herth FJF, Kahn N, et al. Effects of a 6 week whole-body vibration training (WBVT) in stable COPD patients: a randomized clinical trial. Pneumologie (Stuttgart, Germany). 2017;71.
558. Oberste M, Großheinrich N, Wunram HL, Graf JL, Ziemendorff A, Meinhardt A, et al. Effects of a 6-week, whole-body vibration strength-training on depression symptoms, endocrinological and neurobiological parameters in adolescent inpatients experiencing a major depressive episode (the "Balancing Vibrations Study"): study protocol for a randomized placebo-controlled trial. Trials. 2018;19(1):347.
559. Pavlovic A, Nichols DL, Biggerstaff K, Davis R. The Effects Of A 10-week Whole-body Vibration Program On Balance And Lower Body Muscular Strength In Adult Women. MEDICINE AND SCIENCE IN SPORTS AND EXERCISE. 2016;48(5):610-.
560. del Pozo-Cruz B, Alfonso-Rosa RM, del Pozo-Cruz J, Sanudo B, Rogers ME. Effects of a 12-wk whole-body vibration based intervention to improve type 2 diabetes. Maturitas 2014 Jan;77(1):52-58. 2014.
561. Zheng A, Sakari R, Cheng SM, Hietikko A, Moilanen P, Timonen J, et al. Effects of a low-frequency sound wave therapy programme on functional capacity, blood circulation and bone metabolism in frail old men and women. CLINICAL REHABILITATION. 2009;23(10):897-908.
562. Dennerlein JT, Cavallari JM, Kim JHJ, Green NH. The effects of a new seat suspension system on whole body vibration exposure and driver low back pain and disability: results from a randomized controlled trial in truck drivers. Applied Ergonomics 2022 Jan;98:103588. 2022.
563. Gómez-Cabello A, González-Agüero A, Morales S, Ara I, Casajús JA, Vicente-Rodríguez G. Effects of a short-term whole body vibration intervention on bone mass and structure in elderly people. JOURNAL OF SCIENCE AND MEDICINE IN SPORT. 2014;17(2):160-4.
564. Gomez-Cabello A, Gonzalez-Aguero A, Ara I, Casajus JA, Vicente-Rodriguez G. Effects of a short-term whole body vibration intervention on lean mass in elderly people. Nutricion Hospitalaria 2013 Jul-Aug;28(4):1255-1258. 2013.
565. Gómez-Cabello A, González-Agüero A, Ara I, Casajús JA, Vicente-Rodríguez G. Effects of a short-term whole body vibration intervention on physical fitness in elderly people. Maturitas. 2013;74(3):276‐8.
566. Guedes-Aguiar EDO, Taiar R, Paineiras-Domingos LL, Monteiro-Oliveira BB, da Cunha de Sá-Caputo D, Bernardo-Filho M. Effects of a Single Session of Systemic Vibratory Therapy on Flexibility, Perception of Exertion and Handgrip Strength in Chronic Obstructive Pulmonary Disease Individuals: A Quasi-Experimental Clinical Trial. Journal of Clinical Medicine. 2023;12(9).
567. Chan KS, Liu CW, Chen TW, Weng MC, Huang MH, Chen CH. Effects of a single session of whole body vibration on ankle plantarflexion spasticity and gait performance in patients with chronic stroke: a randomized controlled trial. Clinical Rehabilitation 2012 Dec;26(12):1087-1095. 2012.
568. Blazizza MFS, Rahal SC, Santos IFC, Silva BM, Ferreira GM, Oba E, et al. Effects of a single session of whole-body vibration exercise on haematological and biochemical parameters, and serum cortisol levels in cats. COMPARATIVE EXERCISE PHYSIOLOGY. 2021;17(1):1-6.
569. Sitjà-Rabert M, Martínez-Zapata MJ, Fort Vanmeerhaeghe A, Rey Abella F, Romero-Rodríguez D, Bonfill X. Effects of a whole body vibration (WBV) exercise intervention for institutionalized older people: a randomized, multicentre, parallel, clinical trial. Journal of the American Medical Directors Association. 2015;16(2):125‐31.
570. Bissonnette DR, Weir PL, Leigh L, Kenno K. The Effects of a Whole-Body Advanced Vibration Exercise Program on Flexibility, Balance, and Strength in Seniors. Physical & Occupational Therapy in Geriatrics. 2010;28(3):225-34.
571. Otten S, Prokop A, Oschwald V, Maas V, Streckmann F, Bloch W, et al. Effects of a whole-body vibration intervention in children and adolescents after inpatient anticancer therapy on ankle dorsiflexion function and balance control. Oncology Research and Treatment. 2020;43:183.
572. Fagnani F, Giombini A, Di Cesare A, Pigozzi F, Di Salvo V. The effects of a whole-body vibration program on muscle performance and flexibility in female athletes. American Journal of Physical Medicine & Rehabilitation. 2006;85(12):956-62.
573. Tohidast SA, Bagheri R, Safavi-Farokhi Z, Khaleghi Hashemian M, Delkhosh CT. The Effects of Acute and Long-Term Whole-Body Vibration Training on the Postural Control During Cognitive Task in Patients With Chronic Ankle Instability. Journal of Sport Rehabilitation. 2021;30(8):1121-8.
574. Abdolhosseini P, Lark S, Wadsworth D, Stoner L. The effects of acute bouts of whole body vibration on central hemodynamics in frail older adults: a pilot study. Physical & Occupational Therapy in Geriatrics 2019;37(4):223-233. 2019.
575. Cochrane DJ, Hawke EJ. Effects of acute upper-body vibration on strength and power variables in climbers. Journal of Strength and Conditioning Research. 2007;21(2):527-31.
576. Lam FMH, Liao LR, Kwok TCY, Pang MYC. Effects of adding whole-body vibration to routine day activity program on physical functioning in elderly with mild or moderate dementia: a randomized controlled trial. International journal of geriatric psychiatry. 2016;(no pagination).
577. Behboudi L, Azarbayjani MA, Aghaalinejad H, Salavati M. Effects of aerobic exercise and whole body vibration on glycaemia control in type 2 diabetic males. Asian Journal of Sports Medicine 2011 Jun;2(2):83-90. 2011.
578. Cheng HY, Yu YC, Wong A-K, Tsai YS, Ju YY. Effects of an eight-week whole body vibration on lower extremity muscle tone and function in children with cerebral palsy. Research in Developmental Disabilities 2015 Mar;38:256-261. 2015.
579. Haß U, Kochlik B, Herpich C, Rudloff S, Norman K. Effects of an Omega-3 Supplemented, High-Protein Diet in Combination with Vibration and Resistance Exercise on Muscle Power and Inflammation in Old Adults: A Pilot Randomized Controlled Trial. Nutrients. 2022;14(20).
580. Smith DT, Judge S, Malone A, Moynes RC, Conviser J, Skinner JS. Effects of bioDensity Training and Power Plate Whole-Body Vibration on Strength, Balance, and Functional Independence in Older Adults. Journal of Aging & Physical Activity. 2016;24(1):139-48.
581. Fernandez P, Pasqualini M, Locrelle H, Normand M, Bonneau C, Proust MHL, et al. The effects of combined amplitude and high-frequency vibration on physically inactive osteopenic postmenopausal women. FRONTIERS IN PHYSIOLOGY. 2022;13.
582. Bemben DA, Palmer IJ, Bemben MG, Knehans AW. Effects of combined whole-body vibration and resistance training on muscular strength and bone metabolism in postmenopausal women. Bone. 2010;47(3):650-6.
583. Osawa Y, Oguma Y. Effects of combining whole-body vibration with exercise on the consequences of detraining on muscle performance in untrained adults. Journal of strength and conditioning research. 2013;27(4):1074‐82.
584. Gloeckl R, Heinzelmann I, Seeberg S, Damisch T, Hitzl W, Kenn K. Effects of complementary whole-body vibration training in patients after lung transplantation: a randomized, controlled trial. Journal of heart and lung transplantation. 2015;34(11 // (GSK) *GlaxoSmithKline*):1455‐61.
585. Yang F, Finlayson M, Bethoux F, Su X, Dillon L, Maldonado HM. Effects of controlled whole-body vibration training in improving fall risk factors among individuals with multiple sclerosis: A pilot study. Disability and rehabilitation. 2018;40(5):553-60.
586. Martínez-Pardo E, Romero-Arenas S, Alcaraz PE. Effects of different amplitudes (high vs. low) of whole-body vibration training in active adults. Journal of strength and conditioning research. 2013;27(7):1798‐806.
587. Lu LQ, Mao L, Feng YW, Ainsworth BE, Liu Y, Chen N. Effects of different exercise training modes on muscle strength and physical performance in older people with sarcopenia: a systematic review and meta-analysis. BMC GERIATRICS. 2021;21(1).
588. Sajadi N, Bagheri R, Amiri A, Maroufi N, Shadmehr A, Pourahmadi M. Effects of different frequencies of whole body vibration on repositioning error in patients with chronic low back pain in different angles of lumbar flexion. Journal of Manipulative and Physiological Therapeutics 2019 May;42(4):227-236. 2019.
589. Marín PJ, Herrero AJ, Sáinz N, Rhea MR, García-López D. Effects of different magnitudes of whole-body vibration on arm muscular performance. Journal of strength and conditioning research. 2010;24(9):2506‐11.
590. Marín PJ, García Rioja J, Bernardo M, Hazell TJ. EFFECTS OF DIFFERENT MAGNITUDES OF WHOLE-BODY VIBRATION ON DYNAMIC SQUATTING PERFORMANCE. JOURNAL OF STRENGTH AND CONDITIONING RESEARCH. 2015;29(10):2881-7.
591. Gregov C, Salaj S. THE EFFECTS OF DIFFERENT TRAINING MODALITIES ON BONE MASS: A REVIEW. KINESIOLOGY. 2014;46:10-29.
592. Marín PJ, Torres-Luque G, Hernández-García R, García-López D, Garatachea N. Effects of different vibration exercises on bench press. International Journal of Sports Medicine. 2011;32(10):743-8.
593. Yang Z, Miller T, Xiang Z, Pang MYC. Effects of different vibration frequencies on muscle strength, bone turnover and walking endurance in chronic stroke. Scientific reports. 2021;11(1):121.
594. Deak GF, Boros-Balint I, Szabo-Csifo B. EFFECTS OF DIFFERENT WHOLE BODY VIBRATION FREQUENCIES ON THE MAXIMAL ISOMETRIC FORCE GENERATED BY QUADRICEPS MUSCLES. ELEARNING VISION 2020!, VOL III2016. p. 340-5.
595. Zhao R, Zhao M, Xu Z. The effects of differing resistance training modes on the preservation of bone mineral density in postmenopausal women: a meta-analysis. OSTEOPOROSIS INTERNATIONAL. 2015;26(5):1605-18.
596. Sasso GRD, Florencio-Silva R, Santos MA, Teixeira CD, Reginato RD, Simoes MD, et al. Effects of early and late treatments of low-intensity, high-frequency mechanical vibration on bone parameters in rats. GYNECOLOGICAL ENDOCRINOLOGY. 2015;31(12):980-6.
597. Pistone EM, Laudani L, Camillieri G, Di Cagno A, Tomassi G, Macaluso A, et al. Effects of early whole-body vibration treatment on knee neuromuscular function and postural control after anterior cruciate ligament reconstruction: a randomized controlled trial. Journal of rehabilitation medicine. 2016;48(10):880‐6.
598. Santin-Medeiros F, Santos-Lozano A, Rey-Lopez JP, Garatachea N. Effects of eight months of whole body vibration training on hip bone mass in older women. Nutricion Hospitalaria 2015 Jul-Aug;31(4):1654-1659. 2015.
599. Santin-Medeiros F, Rey-López JP, Santos-Lozano A, Cristi-Montero CS, Garatachea Vallejo N. Effects of Eight Months of Whole-Body Vibration Training on the Muscle Mass and Functional Capacity of Elderly Women. Journal of strength and conditioning research. 2015;29(7):1863‐9.
600. Odenwald S, Krumm D. Effects of elastic compression sleeves on the biodynamic response to external vibration of the hand-arm system. ENGINEERING OF SPORT 102014. p. 114-9.
601. Irct20090301001722N. Effects of Electrical stimulation and Whole Body vibration therapy with transcranial direct current stimulation on spactic cerebral palsy children. https://trialsearchwhoint/Trial2aspx?TrialID=IRCT20090301001722N29. 2023.
602. Isrctn. The effects of exercise and vibration therapy on bone density and physical function in females aged 55-80 with osteoporosis. https://trialsearchwhoint/Trial2aspx?TrialID=ISRCTN17157277. 2011.
603. Yañez-Álvarez A, Bermúdez-Pulgarín B, Hernández-Sánchez S, Albornoz-Cabello M. Effects of exercise combined with whole body vibration in patients with patellofemoral pain syndrome: a randomised-controlled clinical trial. BMC Musculoskeletal Disorders. 2020;21(1):N.PAG-N.PAG.
604. Tsekoura M, Billis E, Kastrinis A, Katsoulaki M, Fousekis K, Tsepis E, et al. The effects of exercise in patients with sarcopenia. Advances in Experimental Medicine and Biology 2021;1337:281-290. 2021.
605. Xu JC, Lombardi G, Jiao W, Banfi G. Effects of Exercise on Bone Status in Female Subjects, from Young Girls to Postmenopausal Women: An Overview of Systematic Reviews and Meta-Analyses. SPORTS MEDICINE. 2016;46(8):1165-82.
606. Russo CR. The effects of exercise on bone. Basic concepts and implications for the prevention of fractures. CLINICAL CASES IN MINERAL AND BONE METABOLISM. 2009;6(3):223-8.
607. Shen Y, Liu D, Li S, He Y, Tan F, Sun X, et al. Effects of Exercise on Patients Important Outcomes in Older People With Sarcopenia: An Umbrella Review of Meta-Analyses of Randomized Controlled Trials. Frontiers in Medicine. 2022;9.
608. Salse-Batan J, Sanchez-Lastra MA, Suarez-Iglesias D, Perez CA. Effects of exercise training on obesity-related parameters in people with intellectual disabilities: systematic review and meta-analysis. Journal of Intellectual Disability Research 2022 May;66(5):413-441. 2022.
609. Kennis E, Verschueren SM, Bogaerts A, Coudyzer W, Boonen S, Delecluse C. Effects of fitness and vibration training on muscle quality: a 1-year postintervention follow-up in older men. Archives of Physical Medicine and Rehabilitation 2013 May;94(5):910-918. 2013.
610. Rabini A, de Sire A, Marzetti E, Gimigliano R, Ferriero G, Piazzini DB, et al. Effects of focal muscle vibration on physical functioning in patients with knee osteoarthritis: a randomized controlled trial European Journal of Physical and Rehabilitation Medicine 2015 Oct;51(5):513-520. 2015.
611. Brunetti O, Botti FM, Brunetti A, Biscarini A, Scarponi AM, Filippi GM, et al. Effects of focal vibration on bone mineral density and motor performance of postmenopausal osteoporotic women. JOURNAL OF SPORTS MEDICINE AND PHYSICAL FITNESS. 2015;55(1-2):118-27.
612. Canet-Vintró M, Rodríguez-Sanz J, López-de-Celis C, Campañá-Arnal E, Hidalgo-Garcia C, Pérez-Bellmunt A. Effects of focal vibration on changes in sports performance in amateur athletes: A randomized clinical trial. JOURNAL OF ORTHOPAEDIC RESEARCH. 2024.
613. Vikmoen O, Raastad T, Seynnes O, Bergstrom K, Ellefsen S, Ronnestad BR. Effects of Heavy Strength Training on Running Performance and Determinants of Running Performance in Female Endurance Athletes. PLOS ONE. 2016;11(3).
614. Di Giminiani R, Manno R, Scrimaglio R, Sementilli G, Tihanyi J. Effects of individualized whole-body vibration on muscle flexibility and mechanical power. Journal of Sports Medicine & Physical Fitness. 2010;50(2):139-51.
615. Kang S, Park I. Effects of Instability Neuromuscular Training Using an Inertial Load of Water on the Balance Ability of Healthy Older Women: A Randomized Clinical Trial. JOURNAL OF FUNCTIONAL MORPHOLOGY AND KINESIOLOGY. 2024;9(1).
616. Tankisheva E, Bogaerts A, Boonen S, Feys H, Verschueren S. Effects of Intensive Whole-Body Vibration Training on Muscle Strength and Balance in Adults With Chronic Stroke: A Randomized Controlled Pilot Study. Archives of Physical Medicine & Rehabilitation. 2014;95(3):439-46.
617. Timón R, González-Custodio A, Gusi N, Olcina G. Effects of intermittent hypoxia and whole-body vibration training on health-related outcomes in older adults. Aging Clinical and Experimental Research. 2024;36(1).
618. Nct. Effects of Kegel ex's With and Without WBV on PFM Strength, Incontinence Intensity, and QOL in Patients With SUI. https://clinicaltrialsgov/ct2/show/NCT06242093. 2024.
619. Sun LW, Luan HQ, Huang YF, Wang Y, Fan YB. Effects of Local Vibration on Bone Loss in Tail-Suspended Rats. INTERNATIONAL JOURNAL OF SPORTS MEDICINE. 2014;35(7):615-24.
620. Pietrangelo T, Mancinelli R, Toniolo L, Cancellara L, Paoli A, Puglielli C, et al. Effects of local vibrations on skeletal muscle trophism in elderly people: Mechanical, cellular, and molecular events. INTERNATIONAL JOURNAL OF MOLECULAR MEDICINE. 2009;24(4):503-12.
621. Nam SS, Sunoo S, Park HY, Moon HW. The effects of long-term whole-body vibration and aerobic exercise on body composition and bone mineral density in obese middle-aged women. Journal of Exercise Nutrition and Biochemistry 2016 Jun;20(2):19-27. 2016.
622. Damijan Z. The effects of low-frequency vibrations on hepatic profile of blood. EUROPEAN PHYSICAL JOURNAL-SPECIAL TOPICS. 2008;154:45-9.
623. Ju-Yul Y, Seung-Rok K, Hye-Seong K, Yu Hui W, Sung-Hee P, Jeong-Hwan S, et al. Effects of Low-Frequency Whole-Body Vibration on Muscle Activation, Fatigue, and Oxygen Consumption in Healthy Young Adults: A Single-Group Repeated-Measures Controlled Trial. Journal of Sport Rehabilitation. 2022;31(8):984-92.
624. Sehmisch S, Galal R, Kolios L, Tezval M, Dullin C, Zimmer S, et al. Effects of low-magnitude, high-frequency mechanical stimulation in the rat osteopenia model. Osteoporosis International. 2009;20(12):1999-2008.
625. Yue Y, Deng M, Liao S, Xiao G, Huang Y. Effects of mindfulness training combined with Tai Chi in patients with diabetic peripheral neuropathy. Journal of Visualized Experiments 2023 Jul;197:e65421. 2023.
626. Marín-Cascales E, Alcaraz PE, Ramos-Campo DJ, Rubio-Arias JA. Effects of multicomponent training on lean and bone mass in postmenopausal and older women: a systematic review. MENOPAUSE-THE JOURNAL OF THE NORTH AMERICAN MENOPAUSE SOCIETY. 2018;25(3):346-56.
627. Li FB, Lu P, Wu HT, Wang MH, Wang JD. Effects of Music, Massage, Exercise, or Acupuncture in the Treatment of Depression Among College Students: A Network Meta-Analysis. Neuropsychiatric Disease and Treatment. 2023;19:1725-39.
628. Arumugam A, Bjorklund M, Mikko S, Hager CK. Effects of neuromuscular training on knee proprioception in individuals with anterior cruciate ligament injury: a systematic review and GRADE evidence synthesis BMJ Open 2021 May;11(5):e049226. 2021.
629. Liu S, Moncada R, Schetter V, Ezenwa B. Effects of novel multi-nodal micro-vibration at multiple frequencies on bone turnover markers and bone strengthen in ovariectomized rats. FASEB Journal. 2015;29.
630. Weissenfels A, Wirtz N, Kleinoder H, Frohlich M, Kohl M, Von Stengel S, et al. Effects of novel training technologies on chronic non-specific low back pain - Preliminary results of a randomized multicenter study. European spine journal. 2018;27(11):2899‐.
631. Karamehmetoglu SS, Karacan I, Çidem M, Küçük SH, Ekmekçi H, Bahadir C. Effects of osteocytes on vibration-induced reflex muscle activity in postmenopausal women. TURKISH JOURNAL OF MEDICAL SCIENCES. 2014;44(4):630-8.
632. Adams M, Gordt-Oesterwind K, Bongartz M, Zimmermann S, Seide S, Braun V, et al. Effects of Physical Activity Interventions on Strength, Balance and Falls in Middle-Aged Adults: A Systematic Review and Meta-Analysis. SPORTS MEDICINE-OPEN. 2023;9(1).
633. Hejazi K, Askari R, Hofmeister M. Effects of physical exercise on bone mineral density in older postmenopausal women: a systematic review and meta-analysis of randomized controlled trials. ARCHIVES OF OSTEOPOROSIS. 2022;17(1).
634. Zhang N, Fard M, Bhuiyan MHU, Verhagen D, Azari MF, Robinson SR. The effects of physical vibration on heart rate variability as a measure of drowsiness. Ergonomics. 2018;61(9):1259-72.
635. Ramachandran AK, Singh U, Ramirez-Campillo R, Clemente FM, Afonso J, Granacher U. Effects of Plyometric Jump Training on Balance Performance in Healthy Participants: A Systematic Review With Meta-Analysis. Frontiers in Physiology. 2021;12.
636. Sánchez-Sixto A, Harrison AJ, Floría P. Effects of Plyometric vs. Combined Plyometric Training on Vertical Jump Biomechanics in Female Basketball Players. JOURNAL OF HUMAN KINETICS. 2021;77(1):25-35.
637. Bush JA, Blog GL, Kang J, Faigenbaum AD, Ratamess NA. Effects of quadriceps strength after static and dynamic whole-body vibration exercise. Journal of strength and conditioning research. 2015;29(5):1367‐77.
638. Karacan I, Çidem M, Özen A, Gün K, Uludag M, Bahadir C, et al. The Effects of Radius Bone Density on the Resting Myoelectrical Activity of Contralateral Wrist Flexors in Subjects Exposed to Unilateral Forearm Vibration. TURKIYE KLINIKLERI TIP BILIMLERI DERGISI. 2012;32(6):1673-80.
639. Haas CT, Turbanski S, Kessler K, Schmidtbleicher D. The effects of random whole-body-vibration on motor symptoms in Parkinson's disease. Neurorehabilitation 2006;21(1):29-36. 2006.
640. Mesquita RNO, Taylor JL, Trajano GS, Škarabot J, Holobar A, Gonçalves BAM, et al. Effects of reciprocal inhibition and whole-body relaxation on persistent inward currents estimated by two different methods. Journal of Physiology. 2022;600(11):2765-87.
641. Osawa Y, Oguma Y. Effects of resistance training with whole-body vibration on muscle fitness in untrained adults. Scandinavian journal of medicine & science in sports. 2013;23(1):84‐95.
642. Lai CC, Tu YK, Wang TG, Huang YT, Chien KL. Effects of resistance training, endurance training and whole-body vibration on lean body mass, muscle strength and physical performance in older people: A systematic review and network meta-analysis. Age and Ageing. 2018;47(3):367-73.
643. Marchand F, Laudner K, Delank KS, Schwesig R, Steinmetz A. Effects of Sensorimotor Training on Transversus Abdominis Activation in Chronic Low Back Pain Patients. Journal of Personalized Medicine. 2023;13(5).
644. Costantino C, Petraglia F, Sabetta LL, Giumelli R. Effects of single or multiple sessions of whole body vibration in stroke: is there any evidence to support the clinical use in rehabilitation? Rehabilitation Research and Practice 2018 Jul 30;(8491859):Epub. 2018.
645. Connolly LJ, Scott S, Mohr M, Ermidis G, Julian R, Bangsbo J, et al. Effects of small-volume soccer and vibration training on body composition, aerobic fitness, and muscular PCr kinetics for inactive women aged 20-45. JOURNAL OF SPORT AND HEALTH SCIENCE. 2014;3(4):284-92.
646. Tsuji T, Yoon JY, Mitsuishi Y, Someya N, Kozawa T, Okura T, et al. Effects of static acceleration training with a whole-body vibration machine in community-dwelling older adults. Japanese Journal of Physical Fitness and Sports Medicine. 2012;61(2):211-9.
647. Martín-Santana E, Hernández-Sánchez S, Herrero-Alonso AJ, García-López D. Effects of static-stretching and whole-body-vibration during warm-ups on bench-press kinematics in males and females college-aged. RICYDE: Revista Internacional de Ciencias del Deporte. 2015;11(42):348-59.
648. Herren K, Schmid S, Rogan S, Radlinger L. Effects of Stochastic Resonance Whole-Body Vibration in Individuals with Unilateral Brain Lesion: A Single-Blind Randomized Controlled Trial: Whole-Body Vibration and Neuromuscular Function. Rehabilitation Research & Practice. 2018:1-11.
649. Roschel H, Barroso R, Tricoli V, Batista MA, Acquesta FM, Serrão JC, et al. Effects of Strength Training Associated With Whole-Body Vibration Training on Running Economy and Vertical Stiffness. J Strength Cond Res. 2015;29(8):2215-20.
650. Alvarez-Barbosa F, del Pozo-Cruz J, del Pozo-Cruz B, Alfonso-Rosa RM, Rogers ME, Zhang YX. Effects of supervised whole body vibration exercise on fall risk factors, functional dependence and health-related quality of life in nursing home residents aged 80+. MATURITAS. 2014;79(4):456-63.
651. Yadolahi F, Rezasoltani A. Effects of tdcs plus whole body vibration training on standing posture in individuals with chronic stroke: a randomized controlled trial. International journal of stroke. 2021;16(2 SUPPL):113‐.
652. Edwards WB, Simonian N, Haider IT, Anschel AS, Chen D, Gordon KE, et al. Effects of Teriparatide and Vibration on Bone Mass and Bone Strength in People with Bone Loss and Spinal Cord Injury: A Randomized, Controlled Trial. JOURNAL OF BONE AND MINERAL RESEARCH. 2018;33(10):1729-40.
653. Oliveira LC, Oliveira RG, Pires-Oliveira DAA. Effects of the Pilates exercise compared to whole body vibration and no treatment controls on muscular strength and quality of life in postmenopausal women: A randomized controlled trial. Isokinetics & Exercise Science. 2018;26(2):149-61.
654. Torres-Nunes L, da Costa-Borges PP, Paineiras-Domingos LL, Bachur JA, Coelho-Oliveira AC, de Sá-Caputo DD, et al. Effects of the Whole-Body Vibration Exercise on Sleep Disorders, Body Temperature, Body Composition, Tone, and Clinical Parameters in a Child with Down Syndrome Who Underwent Total Atrioventricular Septal Defect Surgery: A Case-Report. CHILDREN-BASEL. 2023;10(2).
655. Manzi V, Iellamo F, Alashram AR, d'Onofrio R, Padua E, Casasco M, et al. Effects of three different stretching protocols on hamstring muscle flexibility in professional soccer players: a randomized study. The Journal of Sports Medicine and Physical Fitness 2020 Jul;60(7):999-1004. 2020.
656. Ko MS, Sim YJ, Kim DH, Jeon HS. Effects of three weeks of whole-body vibration training on joint-position sense, balance, and gait in children with cerebral palsy: a randomized controlled study Physiotherapy Canada 2016 Spring;68(2):99-105. 2016.
657. Gómez-Cabello A, Ara I, González-Agüero A, Casajüs JA, Vicente-Rodríguez G. Effects of Training on Bone Mass in Older Adults: A Systematic Review. Sports Medicine. 2012;42(4):301-25.
658. Jaime SJ. The effects of twelve weeks of whole-body vibration training and low-intensity resistance exercise training on arterial function, muscle strength, and physical performance in dynapenic postmenopausal women. 2017:122.
659. Rosado H, Bravo J, Raimundo A, Carvalho J, Marmeleira J, Pereira C. Effects of two 24-week multimodal exercise programs on reaction time, mobility, and dual-task performance in community-dwelling older adults at risk of falling: a randomized controlled trial. BMC Public Health 2021 Nov 10;21(408):Epub. 2021.
660. Esmaeilzadeh S, Akpinar M, Polat S, Yildiz A, Oral A. The effects of two different frequencies of whole-body vibration on knee extensors strength in healthy young volunteers: a randomized trial. Journal of musculoskeletal & neuronal interactions. 2015;15(4):333‐40.
661. Marin-Cascales E, Rubio-Arias JA, Alcaraz PE. Effects of two different neuromuscular training protocols on regional bone mass in postmenopausal women: a randomized controlled trial. Frontiers in Physiology 2019 Jul 10;10(846):Epub. 2019.
662. Klarner A, Stengel SV, Kemmler W, Kladny B, Kalender W. Effects of two different types of whole body vibration on neuromuscular performance and body composition in postmenopausal women:results of the controlled and randomized ELVIS-II-study. Deutsche Medizinische Wochenschrift. 2011;136(42):2133-9.
663. Corrie H, Brooke-Wavell K, Mansfield NJ, Cowley A, Morris R, Masud T. Effects of vertical and side-alternating vibration training on fall risk factors and bone turnover in older people at risk of falls. Age & Ageing. 2015;44(1):115-22.
664. Karakiriou SK, Douda HT, Smilios IG, Volaklis KA, Tokmakidis SP. Effects of vibration and exercise training on bone mineral density and muscle strength in post-menopausal women. EUROPEAN JOURNAL OF SPORT SCIENCE. 2012;12(1):81-8.
665. Rees S, Murphy A, Watsford M. Effects of vibration exercise on muscle performance and mobility in an older population. Journal of Aging and Physical Activity. 2007;15(4):367-81.
666. Başol F, Kara İ, Saldıran TÇ. The Effects of Vibration Exposure on Lower-Limb Extensor Muscles' Stiffness, Elasticity, and Strength Responses in Untrained Young Individuals: A Randomized Controlled Trial. Journal of Sport Rehabilitation. 2023;32(4):415-23.
667. Liao LR, Ng GYF, Jones AYM, Chung RCK, Pang MYC. Effects of vibration intensity, exercise, and motor impairment on leg muscle activity induced by whole-body vibration in people with stroke. Physical Therapy. 2015;95(12):1617-27.
668. Di Giminiani R, Tihanyi J, Safar S, Scrimaglio R. The effects of vibration on explosive and reactive strength when applying individualized vibration frequencies. Journal of Sports Sciences. 2009;27(2):169-77.
669. Sadeghi M, Sawatzky B. Effects of vibration on spasticity in individuals with spinal cord injury. American Journal of Physical Medicine & Rehabilitation 2014 Nov;93(11):995-1007. 2014.
670. Wu S, Ning HT, Xiao SM, Hu MY, Wu XY, Deng HW, et al. Effects of vibration therapy on muscle mass, muscle strength and physical function in older adults with sarcopenia: a systematic review and meta-analysis. European Reviews of Aging and Physical Activity 2020 Sep 17;17(14):Epub. 2020.
671. Marín PJ, Martín-López A, Vicente-Campos D, Angulo-Carrere MT, García-Pastor T, Garatachea N, et al. Effects of vibration training and detraining on balance and muscle strength in older adults. JOURNAL OF SPORTS SCIENCE AND MEDICINE. 2011;10(3):559-64.
672. Koczulla AR, Boeselt T, Koelpin J, Kaufhold F, Veith M, Nell C, et al. Effects of vibration training in interstitial lung diseases: a randomized controlled trial. Respiration 2020 Sep;99(8):658-666. 2020.
673. Yang F, Munoz J, Han LZ, Yang F. Effects of vibration training in reducing risk of slip-related falls among young adults with obesity. Journal of biomechanics. 2017;57:87‐93.
674. Fernandez-Rio J, Terrados N, Fernandez-Garcia B, Suman OE. Effects of vibration training on force production in female basketball players. Journal of strength and conditioning research. 2010;24(5):1373‐80.
675. Marín PJ, Rhea MR. Effects of vibration training on muscle power: A meta-analysis. Journal of Strength and Conditioning Research. 2010;24(3):871-8.
676. Buehler R, Simpkins C, Yang F. Effects of vibration training on quality of life in older adults: a preliminary systematic review and meta-analysis. QUALITY OF LIFE RESEARCH. 2022;31(11):3109-22.
677. Deng W. Effects of Vibration Training on Weight Loss and Heart Rate Variability in the Obese Female College Students. BIOMED RESEARCH INTERNATIONAL. 2022;2022.
678. Lu L, He X, Ma L, Liu Y, Chen N. Effects of vibration training vs. conventional resistance training among community-dwelling older people with sarcopenia: three-arm randomized controlled trial protocol. Frontiers in aging neuroscience. 2022;14.
679. Brouwers JEM, Van Rietbergen B, Ito K, Huiskes R. Effects of vibration treatment on tibial bone of ovariectomized rats analyzed by in vivo micro-CT. Journal of Orthopaedic Research. 2010;28(1):62-9.
680. Marazzi S, Kiper P, Palmer K, Agostini M, Turolla A. Effects of vibratory stimulation on balance and gait in Parkinson's disease: a systematic review and meta-analysis. European Journal of Physical and Rehabilitation Medicine 2021 Apr;57(2):254-264. 2021.
681. Silva AT, Carvalho AJB, Andrades MF, Calixto R, Dias MPF, Silva AM, et al. Effects of vibratory training on plantar impression in patients affected by stroke. INTERNATIONAL JOURNAL OF THERAPY AND REHABILITATION. 2016;23(3):108-13.
682. Linqian L, Xiangfeng H, Lin M, Yu L, Nan C. Effects of viration training vs. conventional resistance training among community-dwelling older people with sarcopenia: three-arm randomized controlled trial protocol. Frontiers in Aging Neuroscience. 2022;14:01-12.
683. Choi ET, Kim YN, Cho WS, Lee DK. The effects of visual control whole body vibration exercise on balance and gait function of stroke patients. Journal of Physical Therapy Science 2016 Nov;28(11):3149-3152. 2016.
684. Nct. Effects of WBV Associated With IMT on Inflammatory Markers, Body Composition, Muscle Strength and Thickness. https://clinicaltrialsgov/show/NCT03689322. 2018.
685. Lin S. Effects of weight-bearing activities on bone density in children and young adults with down syndrome: a systematic review. Biomedical Sciences Instrumentation 2021 Oct;57(4):444-450. 2021.
686. Nct. Effects of Whole Body Vibration and Pilates on Bone Mineral Density in Postmenopausal Women. https://clinicaltrialsgov/show/NCT02769143. 2016.
687. Nct. Effects of Whole Body Vibration and Resistance Exercise on Carotis Intima Media and Muscle Architecture in Hypertension. https://clinicaltrialsgov/show/NCT05775835. 2023.
688. Zaki ME. Effects of Whole Body Vibration and Resistance Training on Bone Mineral Density and Anthropometry in Obese Postmenopausal Women. JOURNAL OF OSTEOPOROSIS. 2014;2014.
689. Wang P, Yang L, Liu C, Wei X, Yang X, Zhou Y, et al. Effects of Whole Body Vibration Exercise associated with Quadriceps Resistance Exercise on functioning and quality of life in patients with knee osteoarthritis: a randomized controlled trial. Clinical Rehabilitation. 2016;30(11):1074-87.
690. Srisaphonphusitti L, Manimmanakorn N, Manimmanakorn A, Hamlin MJ. Effects of whole body vibration exercise combined with weighted vest in older adults: a randomized controlled trial. BMC Geriatrics. 2022;22(1).
691. Cidem M, Karacan I, Diracoglu D, Yildiz A, Kucuk SH, Uludag M, et al. Effects of whole body vibration exercise on muscle strength and reflex myoelectrical activity: bone myoregulation reflex as a potential neuromuscular mechanism. Osteoporosis international. 2012;23:S673‐S4.
692. Chi CI. Effects of whole body vibration exercise on neuromuscular function for individuals with knee osteoarthritis. https://trialsearchwhoint/Trial2aspx?TrialID=ChiCTR-IOR-16009234. 2016.
693. Zhangqi L, Xueqiang W, Seullee L, Xihe H, Lin W, Lai Z, et al. Effects of whole body vibration exercise on neuromuscular function for individuals with knee osteoarthritis: study protocol for a randomized controlled trial. Trials. 2017;18:1-8.
694. Dionello CF, Sa-Caputo D, Pereira H, Sousa-Goncalves CR, Maiworm AI, Morel DS, et al. Effects of whole body vibration exercises on bone mineral density of women with postmenopausal osteoporosis without medications: novel findings and literature review. Journal of Musculoskeletal & Neuronal Interactions 2016 Sep;16(3):193-203. 2016.
695. Nct. Effects of Whole Body Vibration in Diabetic Peripheral Neuropathy. https://clinicaltrialsgov/show/NCT05058807. 2021.
696. Gloeckl R, Heinzelmann I, Baeuerle S, Damm E, Schwedhelm AL, Diril M, et al. Effects of whole body vibration in patients with chronic obstructive pulmonary disease--a randomized controlled trial. Respiratory medicine. 2012;106(1):75‐83.
697. Salhi B, Malfait TJ, Van Maele G, Joos G, van Meerbeeck JP, Derom E. Effects of Whole Body Vibration in Patients With COPD. COPD. 2015;12(5):525‐32.
698. Robinson CC, Barreto RPG, Sbruzzi G, Plentz RDM. The effects of whole body vibration in patients with type 2 diabetes: a systematic review and meta-analysis of randomized controlled trials. Brazilian Journal of Physical Therapy 2016 Jan-Feb;20(1):4-14. 2016.
699. Kienberger Y, Sassmann R, Rieder F, Johansson T, Kässmann H, Pirich C, et al. Effects of whole body vibration in postmenopausal osteopenic women on bone mineral density, muscle strength, postural control and quality of life: the T-bone randomized trial. European Journal of Applied Physiology. 2022;122(11):2331-42.
700. Nct. Effects of Whole Body Vibration in Subacute Stroke Patients. https://clinicaltrialsgov/show/NCT00796237. 2008.
701. Pollock RD, Provan S, Martin FC, Newham DJ. The effects of whole body vibration on balance, joint position sense and cutaneous sensation. European journal of applied physiology. 2011;111(12):3069‐77.
702. von Stengel S, Kemmler W, Engelke K, Kalender WA. Effects of whole body vibration on bone mineral density and falls: results of the randomized controlled ELVIS study with postmenopausal women. Osteoporosis international. 2011;22(1):317‐25.
703. Davis R, Sanborn C, Nichols D, Bazett-Jones DM, Dugan EL. The Effects of Whole Body Vibration on Bone Mineral Density for a Person With a Spinal Cord Injury: A Case Study. ADAPTED PHYSICAL ACTIVITY QUARTERLY. 2010;27(1):60-72.
704. Oliveira LC, Oliveira RG, Pires-Oliveira DAA. Effects of whole body vibration on bone mineral density in postmenopausal women: a systematic review and meta-analysis. Osteoporosis International 2016 Oct;27(10):2913-2933. 2016.
705. Chen SW, Yi J. (Effects of whole body vibration on bone strength and physical fitness in elderly COPD patients complicated with osteoporosis) Zhongguo Ying Yong Sheng Li Xue Za Zhi [Chinese Journal of Applied Physiology] 2022 Nov;38(6):690-695. 2022.
706. Duray M, Cetisli-Korkmaz N, Cavlak U. Effects of whole body vibration on functional capacity and respiratory functions in individuals with stroke: a randomized controlled study. Neurorehabilitation 2023;53(1):71-82. 2023.
707. Burq H, Karimi H, Ahmad A, Gilani SA, Hanif A. The effects of whole body vibration on gait after chronic stroke: a randomized controlled clinical trial. The Journal of the Pakistan Medical Association 2021 Nov;71(11):2511-2514. 2021.
708. Manimmanakorn N, Manimmanakorn A, Phuttharak W, Hamlin MJ. Effects of whole body vibration on glycemic indices and peripheral blood flow in type II diabetic patients. The Malaysian Journal of Medical Sciences 2017 Aug;24(4):55-63. 2017.
709. Ebrahimi A, Eftekhari E, Etemadifar M. Effects of whole body vibration on hormonal & functional indices in patients with multiple sclerosis. INDIAN JOURNAL OF MEDICAL RESEARCH. 2015;142:450-8.
710. Liao LR, Jones AYM, Ng GYF, Pang MYC. The effects of whole body vibration on leg muscle activity and oxygen consumption in individuals with stroke. Cerebrovascular Diseases. 2012;33:527.
711. Saquetto M, Carvalho V, Silva C, Conceicao C, Gomes-Neto M. The effects of whole body vibration on mobility and balance in children with cerebral palsy: a systematic review with meta-analysis. Journal of Musculoskeletal & Neuronal Interactions 2015 Jun;15(2):137-144. 2015.
712. Sharififar S, Coronado RA, Romero S, Azari H, Thigpen M. The effects of whole body vibration on mobility and balance in Parkinson disease: a systematic review. Iranian Journal of Medical Sciences 2014 Jul;39(4):318-326. 2014.
713. Dabbs NC, Black CD, Garner JC. Effects of whole body vibration on muscle contractile properties in exercise induced muscle damaged females. Journal of Electromyography and Kinesiology 2016 Oct;30:119-125. 2016.
714. Huang M, Liao LR, Pang MYC. Effects of whole body vibration on muscle spasticity for people with central nervous system disorders: a systematic review Clinical Rehabilitation 2017 Jan;31(1):23-33. 2017.
715. Florentino Pessoa M, Cunha Brandao D, Barros de Sa R, Muniz de Souza H, Bastos Fuzari H, Dornelas de Andrade A. Effects of whole body vibration on muscle strength and quality of life in health elderly: a meta-analysis. Fisioterapia em Movimento [Physical Therapy in Movement] 2017 Dec;30(Suppl 1):S171-S182. 2017.
716. Wang P, Yang X, Yang Y, Yang L, Zhou Y, Liu C, et al. Effects of whole body vibration on pain, stiffness and physical functions in patients with knee osteoarthritis: a systematic review and meta-analysis Clinical Rehabilitation 2015 Oct;29(10):939-951. 2015.
717. Madou KH, Cronin JB. The effects of whole body vibration on physical and physiological capability in special populations. Hong Kong Physiotherapy Journal. 2008;26:24-38.
718. Nct. Effects of Whole Body Vibration on Postmenopausal Risk-factors in Elderly Women. https://clinicaltrialsgov/show/NCT00667667. 2008.
719. Rees SS, Murphy AJ, Watsford ML. Effects of whole body vibration on postural steadiness in an older population. JOURNAL OF SCIENCE AND MEDICINE IN SPORT. 2009;12(4):440-4.
720. Aminian-Far A, Hadian MR, Olyaei G, Talebian S, Bakhtiary AH. (Effects of whole body vibration on prevention and attenuation of delayed-onset muscle soreness following eccentric exercises). Koomesh 2012 Spring;13(3):313-321. 2012.
721. Yang X, Zhou Y, Wang P, He C, He H. Effects of whole body vibration on pulmonary function, functional exercise capacity and quality of life in people with chronic obstructive pulmonary disease: a systematic review. Clinical Rehabilitation 2016 May;30(5):419-431. 2016.
722. Yang J, Seo D. The effects of whole body vibration on static balance, spinal curvature, pain, and disability of patients with low back pain. Journal of Physical Therapy Science 2015 Mar;27(3):805-808. 2015.
723. Wunderer K, Schabrun SM, Chipchase LS. Effects of whole body vibration on strength and functional mobility in multiple sclerosis. Physiotherapy theory and practice. 2010;26(6):374-84.
724. Young S, Wallmann HW, Quiambao KL, Grimes BM. The effects of whole body vibration on the limits of stability in adults with subacute ankle injury. International Journal of Sports Physical Therapy 2021 Jun;16(3):749-755. 2021.
725. Borges DT, Macedo LB, Lins CAA, Sousa CO, Brasileiro JS. Effects of Whole Body Vibration on the Neuromuscular Amplitude of Vastus Lateralis Muscle. JOURNAL OF SPORTS SCIENCE AND MEDICINE. 2017;16(3):414-20.
726. Prisby RD, Lafage-Proust MH, Malaval L, Belli A, Vico L. Effects of whole body vibration on the skeleton and other organ systems in man and animal models: What we know and what we need to know. AGEING RESEARCH REVIEWS. 2008;7(4):319-29.
727. Gómez-Bruton A, González-Agüero A, Matute-Llorente A, Julián C, Lozano-Berges G, Gómez-Cabello A, et al. Effects of Whole Body Vibration on Tibia Strength and Structure of Competitive Adolescent Swimmers: A Randomized Controlled Trial. PM & R: Journal of Injury, Function & Rehabilitation. 2018;10(9):889-97.
728. Wallmann HW, Bell DL, Evans BL, Hyman AA, Goss GK, Paicely AM. THE EFFECTS OF WHOLE BODY VIBRATION ON VERTICAL JUMP, POWER, BALANCE, AND AGILITY IN UNTRAINED ADULTS. International Journal of Sports Physical Therapy. 2019;14(1):55-64.
729. Von Stengel S, Kemmler W, Kalender WA, Engelke K. Effects of Whole Body Vibration on vertical versus rotational devices on osteoporotic risk factors - Preliminary results of the ELVIS II study. Osteoporosis International. 2010;21:S380-S1.
730. Wegener V, Rarack S, Tiffe T, Grill E, Melcher C, Birkenmaier C, et al. Effects of whole body vibration therapy and classic physiotherapy on postural stability in people with back pain: a randomized trial. Clinical Spine Surgery 2019 May;32(4):E214-E220. 2019.
731. Alev A, Mihriban A, Bilge E, Ayca E, Merve K, Seyma C, et al. Effects of whole body vibration therapy in pain, function and depression of the patients with fibromyalgia. Complementary Therapies in Clinical Practice 2017 Aug;28:200-203. 2017.
732. Liao LR, Huang M, Lam FML, Pang MYC. Effects of whole body vibration therapy on body functions and structures, activity, and participation poststroke: a systematic review. Physical Therapy 2014 Sep;94(9):1232-1251. 2014.
733. Lau RW, Liao LR, Yu F, Teo T, Chung RC, Pang MY. The effects of whole body vibration therapy on bone mineral density and leg muscle strength in older adults: a systematic review and meta-analysis. Clinical rehabilitation. 2011;25(11):975-88.
734. del Pozo-Cruz B, Hernandez Mocholi MA, Adsuar JC, Parraca JA, Muro I, Gusi N. Effects of whole body vibration therapy on main outcome measures for chronic non-specific low back pain: a single-blind randomized controlled trial. Journal of Rehabilitation Medicine 2011 Jul;43(8):689-694. 2011.
735. Lau WK. The effects of whole body vibration therapy on neuromotor performance and bone metabolism in individuals with chronic stroke: A randomized controlled trial: Hong Kong Polytechnic University (Hong Kong); 2011.
736. Omidvar M, Alavinia SM, Craven BC. The effects of whole body vibration therapy on reducing fat mass in the adult general population: A systematic review and meta-analyses. JOURNAL OF MUSCULOSKELETAL & NEURONAL INTERACTIONS. 2019;19(4):455-64.
737. Goudarzian M, Ghavi S, Shariat A, Shirvani H, Rahimi M. Effects of whole body vibration training and mental training on mobility, neuromuscular performance, and muscle strength in older men. Journal of Exercise Rehabilitation 2017 Oct;13(5):573-580. 2017.
738. Cai Z-Y, Wang W-Y, Lin J-D, Wu C-M. Effects of whole body vibration training combined with blood flow restriction on muscle adaptation. European Journal of Sport Science. 2021;21(2):204-12.
739. Pleguezuelos E, Perez ME, Guirao L, Samitier B, Costea M, Ortega P, et al. Effects of whole body vibration training in patients with severe chronic obstructive pulmonary disease. Respirology 2013 Aug;18(6):1028-1034. 2013.
740. Gonzalez-Aguero A, Matute-Llorente T, Gomez-Cabello A, Casajus JA, Vicente-Rodriguez G. Effects of whole body vibration training on body composition in adolescents with Down syndrome Research in Developmental Disabilities 2013 May;34(5):1426-1433. 2013.
741. Park SY, Son WM, Kwon OS. Effects of whole body vibration training on body composition, skeletal muscle strength, and cardiovascular health. JOURNAL OF EXERCISE REHABILITATION. 2015;11(6):289-95.
742. Bogaerts AC, Delecluse C, Claessens AL, Troosters T, Boonen S, Verschueren SM. Effects of whole body vibration training on cardiorespiratory fitness and muscle strength in older individuals (a 1-year randomised controlled trial). Age and ageing. 2009;38(4):448‐54.
743. Corum M, Basoglu C, Yakal S, Sahinkaya T, Aksoy C. Effects of whole body vibration training on isokinetic muscular performance, pain, function, and quality of life in female patients with patellofemoral pain: a randomized controlled trial. Journal of Musculoskeletal & Neuronal Interactions 2018 Dec;18(4):473-484. 2018.
744. Delecluse C, Roelants M, Diels R, Koninckx E, Verschueren S. Effects of whole body vibration training on muscle strength and sprint performance in sprint-trained athletes. International journal of sports medicine. 2005;26(8):662‐8.
745. Lu J, Xu G, Wang Y. Effects of whole body vibration training on people with chronic stroke: a systematic review and meta-analysis. Topics in Stroke Rehabilitation 2015;22(3):161-168. 2015.
746. Bogaerts A, Verschueren S, Delecluse C, Claessens AL, Boonen S. Effects of whole body vibration training on postural control in older individuals: a 1 year randomized controlled trial. Gait & posture. 2007;26(2):309‐16.
747. Seo KH, Sin D, Ju EP, Lim JY. Effects of whole body vibration training using side-alternating vibration platform with tilt table in hospitalized older adults with sarcopenia : a randomized controlled pilot study. Age and ageing. 2019;48(2):iv18‐.
748. Lee JM, Lim JY. Effects of whole body vibration training using side-alternating vibration platform with tilt table in hospitalized older adults with sarcopenia: a randomized controlled pilot study. Aging medicine and healthcare. 2019;10:35‐.
749. Rasti E, Rojhani-Shirazi Z, Ebrahimi N, Sobhan MR. Effects of whole body vibration with exercise therapy versus exercise therapy alone on flexibility, vertical jump height, agility and pain in athletes with patellofemoral pain: a randomized clinical trial. BMC musculoskeletal disorders. 2020;21(1):705.
750. Shin S, Lee K, Song C. Effects of whole body vibration with load stimulation in postmenopausal women. Medical science technology. 2018;59(pp 4‐12).
751. Weissenfels A, Teschler M, Willert S, Hettchen M, Frohlich M, Kleinoder H, et al. Effects of whole-body electromyostimulation on chronic nonspecific low back pain in adults: a randomized controlled study. Journal of pain research. 2018;11:1949‐57.
752. Timon R, Tejero J, Brazo-Sayavera J, Crespo C, Olcina G. Effects of whole-body vibration after eccentric exercise on muscle soreness and muscle strength recovery. Journal of Physical Therapy Science 2016 Jun;28(6):1781-1785. 2016.
753. Sanni AA, Blanks AM, Derella CC, Horsager C, Crandall RH, Looney J, et al. The effects of whole-body vibration amplitude on glucose metabolism, inflammation, and skeletal muscle oxygenation. Physiological Reports. 2022;10(5).
754. Chang WD, Chen S, Tsou YA. Effects of whole-body vibration and balance training on female athletes with chronic ankle instability. Journal of clinical medicine. 2021;10(11).
755. Sen EI, Esmaeilzadeh S, Eskiyurt N. Effects of whole-body vibration and high impact exercises on the bone metabolism and functional mobility in postmenopausal women. Journal of Bone and Mineral Metabolism 2020 May;38(3):392-404. 2020.
756. Artero EG, Espada-Fuentes JC, Argüelles-Cienfuegos J, Román A, Gómez-López PJ, Gutiérrez A, et al. Effects of whole-body vibration and resistance training on knee extensors muscular performance. European Journal of Applied Physiology. 2012;112(4):1371-8.
757. Tian SJ, Gao JZ, Gong H, Zhang X, Wang S. Effects of whole-body vibration at different periods on lumbar vertebrae in female rats. MEDICAL ENGINEERING & PHYSICS. 2022;110.
758. Yan Z, Cardinal BJ, Guzman RJ, Maddalozzo WA, Maddalozzo GF. Effects of whole-body vibration compared to a community-based exercise program for improving older adults' balance and mood. Health Science Journal. 2012;6(1):151-60.
759. Jepsen DDB, Masud TT, Larsen AH, Hansen S, Jorgensen NR, Ryg J. Effects of whole-body vibration exercise in combination with parathyroid hormone (1-34) on physical performance measures in osteoporotic women: a secondary analysis from a randomized controlled trial. European geriatric medicine. 2018;9:S99‐.
760. Zheng YL, Hu HY, Liu XC, Su X, Chen PJ, Wang XQ. The effects of whole-body vibration exercise on anticipatory delay of core muscles in patients with nonspecific low back pain. Pain Research & Management 2021 Aug 4;(9274964):Epub. 2021.
761. Edionwe J, Hess C, Fernandez-Rio J, Herndon DN, Andersen CR, Klein GL, et al. Effects of whole-body vibration exercise on bone mineral content and density in thermally injured children. Burns (03054179). 2016;42(3):605-13.
762. Moura ECSCD, Cunha BLM, Oliveira TGD, Amorim NTS, Leitão CCDS, Cavalcanti FCB, et al. Effects of whole-body vibration exercise on functional capacity, muscle strength and thickness, and quality of life of post-COVID-19 patients: Case report. Journal of Bodywork and Movement Therapies. 2024;39:231-6.
763. Rehn B, Nilsson P, Norgren M. Effects of whole-body vibration exercise on human bone density–systematic review. Physical Therapy Reviews. 2008;13(6):427-33.
764. Rees SS, Murphy AJ, Watsford ML. Effects of whole-body vibration exercise on lower-extremity muscle strength and power in an older population: a randomized clinical trial. Physical Therapy. 2008;88(4):462-70.
765. Tsuji T, Yoon J, Aiba T, Kanamori A, Okura T, Tanaka K. Effects of whole-body vibration exercise on muscular strength and power, functional mobility and self-reported knee function in middle-aged and older Japanese women with knee pain. Knee. 2014;21(6):1088-95.
766. Blizzard RR, Young JL. Effects of whole-body vibration exercise on prevention of the negative effects of prolonged bed rest. Physical Therapy Reviews. 2010;15(5):391-8.
767. Figueroa A, Kalfon R, Madzima TA, Wong A. Effects of whole-body vibration exercise training on aortic wave reflection and muscle strength in postmenopausal women with prehypertension and hypertension. Journal of human hypertension. 2014;28(2):118‐22.
768. Preatoni E, Colombo A, Verga M, Galvani C, Faina M, Rodano R, et al. The effects of whole-body vibration in isolation or combined with strength training in female athletes. Journal of Strength and Conditioning Research. 2012;26(9):2495-506.
769. Gomes-Neto M, de Sa-Caputo DDC, Paineiras-Domingos LL, Brandao AA, Neves MF, Marin PJ, et al. Effects of whole-body vibration in older adult patients with type 2 diabetes mellitus: a systematic review and meta-analysis. Canadian Journal of Diabetes 2019 Oct;43(7):524-529. 2019.
770. Schuhfried O, Mittermaier C, Jovanovic T, Pieber K, Paternostro-Sluga T. Effects of whole-body vibration in patients with multiple sclerosis: a pilot study. Clinical Rehabilitation 2005 Dec;19(8):834-842. 2005.
771. Yoosefinejad AK, Shadmehr A, Olyaei G, Talebian S, Bagheri H, Mohajeri-Tehrani MR. Effects of whole-body vibration on a diabetic type 2 patient with peripheral neuropathy. Health Science Journal. 2012;6(3):576-83.
772. Lam FM, Chan PF, Liao LR, Woo J, Hui E, Lai CW, et al. Effects of whole-body vibration on balance and mobility in institutionalized older adults: a randomized controlled trial. Clinical rehabilitation. 2018;32(4):462‐72.
773. Liao LR, Ng GYF, Jones AYM, Huang MZ, Pang MYC. Effects of whole-body vibration on body functions and structures, activity and participation in individuals with stroke: a randomized controlled trial. Physiotherapy (united kingdom). 2015;101:eS870‐eS1.
774. Minematsu A, Nishii Y, Sakata S. Effects of whole-body vibration on bone properties in aged rats. JOURNAL OF MUSCULOSKELETAL & NEURONAL INTERACTIONS. 2021;21(2):287-97.
775. Alvarez-Barbosa F, del Pozo-Cruz J, del Pozo-Cruz B, Garcia-Hermoso A, Alfonso-Rosa RM. Effects of whole-body vibration on functional mobility, balance, gait strength, and quality of life in institutionalized older people: a systematic review and meta-analysis of randomized controlled trials. Journal of Aging and Physical Activity 2020 Apr;28(2):219-230. 2020.
776. Wong A, Figueroa A. Effects of whole-body vibration on heart rate variability: acute responses and training adaptations. CLINICAL PHYSIOLOGY AND FUNCTIONAL IMAGING. 2019;39(2):115-21.
777. Aslam F, Baig MO. Effects of Whole-Body Vibration on Lower Extremity with Diplegic spastic Cerebral Palsy. Pakistan Paediatric Journal. 2022;46(3):329-35.
778. Chen BL, Dong YL, Guo JB, Zheng YL, Zhang J, Wang XQ. Effects of Whole-Body Vibration on Lumbar-Abdominal Muscles Activation in Healthy Young Adults: A Pilot Study. MEDICAL SCIENCE MONITOR. 2019;25:1945-51.
779. Alashram AR, Padua E, Annino G. Effects of whole-body vibration on motor impairments in patients with neurological disorders: a systematic review. American Journal of Physical Medicine & Rehabilitation 2019 Dec;98(12):1084-1098. 2019.
780. Martin PJ, Ferrero CM, Menéndez H, Martín J, Herrero AJ. Effects of Whole-Body Vibration on Muscle Architecture, Muscle Strength, and Balance in Stroke Patients: A Randomized Controlled Trial. American Journal of Physical Medicine & Rehabilitation. 2013;92(10):881-8.
781. Osawa Y, Oguma Y, Ishii N. The effects of whole-body vibration on muscle strength and power: a meta-analysis. Journal of Musculoskeletal & Neuronal Interactions 2013 Sep;13(3):342-352. 2013.
782. Alam MM, Khan AA, Farooq M. Effects of whole-body vibration on muscle strength, balance and functional mobility in patients with multiple sclerosis: a systematic review and meta-analysis. Journal of Musculoskeletal Research 2020 Dec;23(4):2050019. 2020.
783. Saquetto MB, Pereira FF, Queiroz RS, da Silva CM, Conceicao CS, Gomes Neto M. Effects of whole-body vibration on muscle strength, bone mineral content and density, and balance and body composition of children and adolescents with Down syndrome: a systematic review. Osteoporosis International 2018 Mar;29(3):527-533. 2018.
784. Marinho PEM, Rocha LG, Araújo Filho JC, Araújo AXP, Andrade MDA, Taiar R, et al. Effects of whole-body vibration on muscle strength, quadriceps muscle thickness and functional capacity in kidney transplant recipients: a randomized controlled trial. Journal of bodywork and movement therapies. 2021;26:101‐7.
785. Ji Q, He H, Zhang C, Lu C, Zheng Y, Luo XT, et al. Effects of whole-body vibration on neuromuscular performance in individuals with spinal cord injury: a systematic review Clinical Rehabilitation 2017 Oct;31(10):1279-1291. 2017.
786. Wong ML, Widerstrom-Noga E, Field-Fote EC. Effects of whole-body vibration on neuropathic pain and the relationship between pain and spasticity in persons with spinal cord injury. Spinal Cord 2022 Nov;60(11):963-970. 2022.
787. Rogan S, Hilfiker R, Herren K, Radlinger L, de Bruin ED. Effects of whole-body vibration on postural control in elderly: a systematic review and meta-analysis. BMC Geriatrics 2011 Nov 3;11(72):Epub. 2011.
788. Rogan S, Taeymans J, Radlinger L, Naepflin S, Ruppen S, Bruelhart Y, et al. Effects of whole-body vibration on postural control in elderly: an update of a systematic review and meta-analysis. Archives of Gerontology and Geriatrics 2017 Nov-Dec;73:95-112. 2017.
789. Rogan S, de Bruin ED, Radlinger L, Joehr C, Wyss C, Stuck NJ, et al. Effects of whole-body vibration on proxies of muscle strength in old adults: a systematic review and meta-analysis on the role of physical capacity level. European Review of Aging and Physical Activity 2015 Dec 8;12(12):Epub. 2015.
790. Osawa Y, Oguma Y. Effects of whole-body vibration on resistance training for untrained adults. J Sports Sci Med. 2011;10(2):328-37.
791. Lau RWK, Teo T, Yu F, Chung RCK, Pang MYC. Effects of whole-body vibration on sensorimotor performance in people with Parkinson disease: a systematic review Physical Therapy 2011 Feb;91(2):198-209. 2011.
792. Goodwill AM, Kidgell DJ. The effects of whole-body vibration on the cross-transfer of strength. The Scientific World Journal. 2012;2012.
793. Abdulghani S, Raimundo A, Rodrigues A, Fatela A, Canhao H. The effects of whole-body vibration on the management of osteoporosis in early menopausal women. Portuguese journal of public health. 2022;40:6.
794. Ahn J-Y, Kim H, Park C-B. Effects of Whole-Body Vibration on Upper Extremity Function and Grip Strength in Patients with Subacute Stroke: A Randomised Single-Blind Controlled Trial. Occupational Therapy International. 2019:1-5.
795. Wu Z, Zou Z, Zhong J, Fu X, Yu L, Wang J, et al. Effects of whole-body vibration plus hip-knee muscle strengthening training on adult patellofemoral pain syndrome: a randomized controlled trial. Disability and Rehabilitation 2022 Oct;44(20):6017-6025. 2022.
796. Ramachandran S, Paul J, Sundaram MS, Varalakshmi S, Sudhakar S. Effects of whole-body vibration therapy among type II diabetes mellitus -- a pilot single blind randomized clinical trial. Research Journal of Pharmacy and Technology 2021 Mar;14(3):1465-1468. 2021.
797. Collado-Mateo D, Adsuar JC, Olivares PR, del Pozo-Cruz B, Parraca JA, del Pozo-Cruz J, et al. Effects of whole-body vibration therapy in patients with fibromyalgia: a systematic literature review. Evidence-Based Complementary and Alternative Medicine 2015;(719082):Epub. 2015.
798. Pang MY, Lau RW, Yip SP. The effects of whole-body vibration therapy on bone turnover, muscle strength, motor function, and spasticity in chronic stroke: a randomized controlled trial. European journal of physical and rehabilitation medicine. 2013;49(4):439‐50.
799. Boggild MK, Tomlinson G, Erlandson MC, Szabo E, Giangregorio LM, Craven BC, et al. Effects of Whole-Body Vibration Therapy on Distal Tibial Myotendinous Density and Volume: a Randomized Controlled Trial in Postmenopausal Women. JBMR plus. 2019;3(5).
800. Lee K. Effects of whole-body vibration therapy on perception thresholds of type 2 diabetic patients with peripheral neuropathy: a randomized controlled trial. Journal of Physical Therapy Science 2017 Sep;29(9):1684-1688. 2017.
801. Gusso S, Colle P, Biggs J, Derraik J, Hofman PL. The effects of whole-body vibration therapy on physical function, bone and muscle mass in adolescents with mild to moderate musculoskeletal disability. International Journal of Pediatric Endocrinology. 2017;2017.
802. Centner C, Ritzmann R, Gollhofer A, König D. Effects of Whole-Body Vibration Training and Blood Flow Restriction on Muscle Adaptations in Women: a Randomized Controlled Trial. Journal of strength and conditioning research. 2020;34(3):603‐8.
803. Verschueren SM, Bogaerts A, Delecluse C, Claessens AL, Haentjens P, Vanderschueren D, et al. The effects of whole-body vibration training and vitamin D supplementation on muscle strength, muscle mass, and bone density in institutionalized elderly women: a 6-month randomized, controlled trial. Journal of bone and mineral research. 2011;26(1):42‐9.
804. Xiong W, Liu X. Effects of whole-body vibration training combined with KAATSU training on lower limb joint muscle strength in older women. Frontiers in Physiology. 2023;14.
805. Merriman H, Jackson K. The effects of whole-body vibration training in aging adults: a systematic review. Journal of Geriatric Physical Therapy 2009 Jul-Sep;32(3):134-145. 2009.
806. Liu Y, Fan Y, Chen X. Effects of whole-body vibration training in static and dynamic semi-squat patterns on the lower limb muscle activity. Scientific reports. 2023;13(1):14432.
807. Chen P, Wang L, Dong S, Ding Y, Jia S, Kou X, et al. Effects of whole-body vibration training on anterior cruciate ligament reconstruction: a meta-analysis. Chinese Journal of Tissue Engineering Research. 2023;27(36):5875-83.
808. Broga˚rdh C, Flansbjer UB, Lexell J. Effects of whole-body vibration training on balance, muscle function, gait performance and perceived participation after stroke: a randomized controlled study. Physiotherapy (united kingdom). 2011;97:eS157‐.
809. Martínez-Pardo E, Martínez-Ruiz E, Alcaraz PE, Rubio-Arias JA. Effects of whole-body vibration training on body composition and physical fitness in recreationally active young adults. Nutricion Hospitalaria. 2015;32(5):1949-59.
810. Rubio-Arias JÁ, Martínez-Aranda LM, Andreu-Caravaca L, Sanz G, Benito PJ, Ramos-Campo DJ. Effects of Whole-Body Vibration Training on Body Composition, Cardiometabolic Risk, and Strength in the Population Who Are Overweight and Obese: A Systematic Review With Meta-analysis. Archives of Physical Medicine & Rehabilitation. 2021;102(12):2442-53.
811. Osawa Y, Oguma Y, Onishi S. Effects of whole-body vibration training on bone-free lean body mass and muscle strength in young adults. Journal of Sports Science and Medicine. 2011;10(1):97-104.
812. Rubio-Arias J, Ramos-Campo DJ, Alcaraz PE, Jiménez Díaz JF, Blazevich AJ. Effects of whole-body vibration training on calf muscle function during maximal isometric voluntary contractions. Scandinavian journal of medicine & science in sports. 2021;31(6):1268‐75.
813. Von Stengel S, Kemmler W, Bebenek M, Engelke K, Kalender WA. Effects of whole-body vibration training on different devices on bone mineral density. Medicine and science in sports and exercise. 2011;43(6):1071‐9.
814. Fort A, Romero D, Bagur C, Guerra M. Effects of whole-body vibration training on explosive strength and postural control in young female athletes. Journal of strength and conditioning research. 2012;26(4):926‐36.
815. Lindberg J, Carlsson J. The effects of whole-body vibration training on gait and walking ability -- a systematic review comparing two quality indexes. Physiotherapy Theory and Practice 2012;28(7):485-498. 2012.
816. Yoon J, Tsuji T, Kanamori A, Tanaka K, Okura T. The effects of whole-body vibration training on knee function and physical performance of middle-aged and elderly woman with knee osteoarthritis and chronic knee pain. Japanese Journal of Physical Fitness and Sports Medicine. 2014;63(4):371-82.
817. Domagalska-Szopa M, Szopa A, Siwiec A, Kwiecien-Czerwieniec I, Schreiber L, Dabek J. Effects of whole-body vibration training on lower limb blood flow in children with myelomeningocele -- a randomized trial. Journal of Clinical Medicine 2021 Sep;10(18):4273. 2021.
818. Zhang M, Wei J, Wu X. Effects of whole-body vibration training on lower limb motor function and neural plasticity in patients with stroke: protocol for a randomised controlled clinical trial. BMJ Open. 2022;12(6).
819. Tan X, Jiang G, Zhang L, Wang D, Wu X. Effects of Whole-Body Vibration Training on Lower Limb Muscle Strength and Physical Performance Among Older Adults: A Systematic Review and Meta-analysis. Archives of Physical Medicine and Rehabilitation. 2023;104(11):1954-65.
820. Hilgers C, Mündermann A, Riehle H, Dettmers C. Effects of whole-body vibration training on physical function in patients with Multiple Sclerosis. NeuroRehabilitation. 2013;32(3):655-63.
821. Paradisis G, Zacharogiannis E. Effects of whole-body vibration training on sprint running kinematics and explosive strength performance. Journal of Sports Science and Medicine. 2007;6(1):44-9.
822. Wadsworth D, Lark S. Effects of whole-body vibration training on the physical function of the frail elderly: an open, randomised control trial. Archives of Physical Medicine and Rehabilitation 2020 Jul;101(7):1111-1119. 2020.
823. Lachance CC, Kenno K, Weir PL, Carr KM, McNevin N, Horton S. The effects of whole-body vibration training on upper and lower body strength in older adults. Critical Reviews in Physical & Rehabilitation Medicine. 2012;24(1-2):35-50.
824. Liu Y, Fan Y, Chen X. Effects of Whole-Body Vibration Training with Different Body Positions and Amplitudes on Lower Limb Muscle Activity in Middle-Aged and Older Women. Dose-Response. 2022;20(3).
825. Wang P, Yang L, Li H, Lei Z, Yang X, Liu C, et al. Effects of whole-body vibration training with quadriceps strengthening exercise on functioning and gait parameters in patients with medial compartment knee osteoarthritis: a randomised controlled preliminary study. Physiotherapy. 2016;102(1):86-92.
826. de Oliveira LC, de Oliveira RG, de Almeida Pires-Oliveira DA. Effects of whole-body vibration versus Pilates exercise on bone mineral density in postmenopausal women: a randomized and controlled clinical trial. Journal of Geriatric Physical Therapy 2019 Apr-Jun;42(2):E23-E31. 2019.
827. Milanese C, Piscitelli F, Simoni C, Pugliarello R, Zancanaro C. Effects of Whole-Body Vibration With or Without Localized Radiofrequency on Anthropometry, Body Composition, and Motor Performance in Young Nonobese Women. Journal of Alternative & Complementary Medicine. 2012;18(1):69-75.
828. Usgu G, Yüksel İ. The effects of whole-body vibration with plyometric training on physical performance in basketball players. Spor Hekimligi Dergisi/Turkish Journal of Sports Medicine. 2022;57(4):164-70.
829. Rogan S, Hilfiker R, Schenk A, Vogler A, Taeymans J. Effects of whole-body vibration with stochastic resonance on balance in persons with balance disability and falls history -- a systematic review. Research in Sports Medicine 2014;22(3):294-313. 2014.
830. Kemmler W, Engelke K, von Stengel S. Effects of Whole-Body-Electromyostimulation on Sarcopenia in Lean, Elderly Sedentary Women. The TEST-III Study. DEUTSCHE ZEITSCHRIFT FUR SPORTMEDIZIN. 2012;63(12):343-+.
831. Rubio‐Arias JÁ, Ramos‐Campo DJ, Alcaraz PE, Jiménez Díaz JF, Blazevich AJ. Effects of whole‐body vibration training on calf muscle function during maximal isometric voluntary contractions. Scandinavian Journal of Medicine & Science in Sports. 2021;31(6):1268-75.
832. Rehn B, Lidström J, Skoglund J, Lindström B. Effects on leg muscular performance from whole-body vibration exercise: A systematic review. Scandinavian Journal of Medicine and Science in Sports. 2007;17(1):2-11.
833. Klarner A, von Stengel S, Kemmler W, Kladny B, Kalender W. Effekte unterschiedlicher ganzkorpervibrationssysteme auf die neuromuskulare leistungsfahigkeit und die korperzusammensetzung postmenopausaler frauen (Effects of two different types of whole body vibration on neuromuscular performance and body composition in postmenopausal women) Deutsche Medizinische Wochenschrift 2011 Oct;136(42):2133-2139. 2011.
834. Kemmler W, von Stengel S, Mayer S, Niedermayer M, Hentschke C, Kalender WA. Effekte von Ganzkorpervibrationen auf die neuromuskulare Leistungsfahigkeit von Frauen uber dem 65. Lebensjahr (Effect of whole body vibration on the neuromuscular performance of females 65 years and older. One-year results of the controlled randomized ELVIS study) Zeitschrift fur Gerontologie und Geriatrie 2010 Apr;43(2):125-132. 2010.
835. Zhi YJ, Zhao J, Zhao H, Yu DD. Efficacy and safety of breviscapine injection in treatment of unstable angina pectoris: systematic review and Meta-analysis. Zhongguo Zhongyao Zazhi. 2018;43(19):3940-55.
836. Vetrovsky T, Steffl M, Stastny P, Tufano JJ. The Efficacy and Safety of Lower-Limb Plyometric Training in Older Adults: A Systematic Review. SPORTS MEDICINE. 2019;49(1):113-31.
837. Seefried L, Genest F, Luksche N, Schneider M, Fazeli G, Brandl M, et al. Efficacy and safety of whole body vibration in maintenance hemodialysis patients - A pilot study. Journal of Musculoskeletal Neuronal Interactions. 2017;17(4):268-74.
838. Furness TP, Maschette WE, Lorenzen C, Naughton GA, Williams MD. Efficacy of a whole-body vibration intervention on functional performance of community-dwelling older adults. Journal of Alternative & Complementary Medicine 2010 Jul;16(7):795-797. 2010.
839. Yang F, Butler AJ. Efficacy of Controlled Whole-Body Vibration Training on Improving Fall Risk Factors in Stroke Survivors: A Meta-analysis. Neurorehabilitation and Neural Repair. 2020;34(4):275-88.
840. Wang HL, Huang W, Zhao YN. Efficacy of Exercise on Muscle Function and Physical Performance in Older Adults with Sarcopenia: An Updated Systematic Review and Meta-Analysis. INTERNATIONAL JOURNAL OF ENVIRONMENTAL RESEARCH AND PUBLIC HEALTH. 2022;19(13).
841. Rajapakse CS, Leonard MB, Kobe EA, Slinger MA, Borges KA, Billig E, et al. The Efficacy of Low-intensity Vibration to Improve Bone Health in Patients with End-stage Renal Disease Is Highly Dependent on Compliance and Muscle Response. ACADEMIC RADIOLOGY. 2017;24(11):1332-42.
842. Chow SKH, Ho CY, Wong HW, Chim YN, Wong RWMY, Cheung WH. Efficacy of low-magnitude high-frequency vibration (LMHFV) on musculoskeletal health of participants on wheelchair: A study protocol for a single-blinded randomised controlled study. BMJ Open. 2020;10(12).
843. De Souza H, Pessoa M, Clemente R, Silva A, Andrade E, Reinaux C, et al. Efficacy of the association of inspiratory muscle training with whole body vibration on respiratory muscle strength, functionality, balance and physical performance in prefrail older women: a randomized double-blind clinical trial. European respiratory journal. 2019;54.
844. Moretti E, Tenório A, Holanda L, Campos A, Lemos A. Efficacy of the whole-body vibration for pain, fatigue and quality of life in women with fibromyalgia: a systematic review. DISABILITY AND REHABILITATION. 2018;40(9):988-96.
845. Sandler EB, Condon K, Field-Fote EC. Efficacy of transcutaneous spinal stimulation versus whole body vibration for spasticity reduction in persons with spinal cord injury. Journal of Clinical Medicine 2021 Aug;10(15):3267. 2021.
846. Tapp LR, Signorile JF. Efficacy of WBV as a modality for inducing changes in body composition, aerobic fitness, and muscular strength: a pilot study. Clinical interventions in aging. 2014;9:63‐72.
847. Sitjà-Rabert M, Rigau D, Fort Vanmeerghaeghe A, Romero-Rodríguez D, Bonastre Subirana M, Bonfill X. Efficacy of whole body vibration exercise in older people: a systematic review. Disability and rehabilitation. 2012;34(11):883-93.
848. Fereydounnia S, Shadmehr A. Efficacy of whole body vibration on neurocognitive parameters in women with and without lumbar hyper-lordosis. Journal of Bodywork and Movement Therapies. 2020;24(1):182-9.
849. Wang W, Wang S, Lin W, Li X, Andersen LL, Wang Y. Efficacy of whole body vibration therapy on pain and functional ability in people with non-specific low back pain: a systematic review. BMC Complementary Medicine and Therapies 2020 May 27;20(158):Epub. 2020.
850. Kim GW, Jo NK, Yun JE, Kim HS, Won YH, Park SH, et al. Efficacy of whole body vibration training for improving muscle strength and physical performance in the elderly. Osteoporosis international. 2020;31(SUPPL 1):S319‐S20.
851. Costantino C, Bertuletti S, Romiti D. Efficacy of Whole-Body Vibration Board Training on Strength in Athletes After Anterior Cruciate Ligament Reconstruction: A Randomized Controlled Study. Clinical Journal of Sport Medicine. 2018;28(4):339-49.
852. Mortada H, Zahreldin AA, Saleh MS, Shahien M, Elfeky A, Abdelhamed AI, et al. The Efficacy of Whole-Body Vibration in Managing Post-burn victims' complications: A Systematic Review. Journal of burn care & research : official publication of the American Burn Association. 2023.
853. Abdel-Aal NM, Allam NM, Eladl HM. Efficacy of whole-body vibration on balance control, postural stability, and mobility after thermal burn injuries: a prospective randomized controlled trial. Clinical Rehabilitation 2021 Nov;35(11):1555-1565. 2021.
854. Mahran HG. Efficacy of whole-body vibration on low bone density in post-burn patients. Physiotherapy Quarterly 2021;29(2):38-43. 2021.
855. Ribeiro VGC, Lacerda ACR, Santos JM, Coelho-Oliveira AC, Fonseca SF, Prates ACN, et al. Efficacy of Whole-Body Vibration Training on Brain-Derived Neurotrophic Factor, Clinical and Functional Outcomes, and Quality of Life in Women with Fibromyalgia Syndrome: a Randomized Controlled Trial. Journal of healthcare engineering. 2021;2021:7593802.
856. Blasimann A, Fleuti U, Rufener M, Elfering A, Radlinger L. Electromyographic activity of back muscles during stochastic whole body vibration. Journal of Musculoskeletal Neuronal Interactions. 2014;14(3):311-7.
857. Cardinale M, Lim J. Electromyography activity of vastus lateralis muscle during whole-body vibrations of different frequencies. JOURNAL OF STRENGTH AND CONDITIONING RESEARCH. 2003;17(3):621-4.
858. Chisari E, Pavone, Sessa G, Ravalli S, Musumeci G. Electromyostimulation and whole-body vibration effects in elder sarcopenic patients. MLTJ-MUSCLES LIGAMENTS AND TENDONS JOURNAL. 2019;9(3):433-41.
859. Da Silva USLG, Villagra HA, Oliva LL, Marconi NF. EMG activity of upper limb on spinal cord injury individuals during whole-body vibration. Acta Physiologica Hungarica. 2016;103(3):361-7.
860. Fares EJ, Charrière N, Montani JP, Schutz Y, Dulloo AG, Miles-Chan JL. Energy Expenditure and Substrate Oxidation in Response to Side-Alternating Whole Body Vibration across Three Commonly-Used Vibration Frequencies. PLOS ONE. 2016;11(3).
861. Raulino RS, de Aguiar FM, de Avelar NCP, Costa IG, Soares JD, Lacerda ACR. ENERGY EXPENDITURE AND SUBSTRATE UTILIZATION DURING WHOLE BODY VIBRATION. REVISTA BRASILEIRA DE MEDICINA DO ESPORTE. 2015;21(2):122-6.
862. Brantberg K, Verrecchia L, Westin M. Enhanced Auditory Sensitivity to Body Vibrations in Superior Canal Dehiscence Syndrome. Audiology and Neurotology. 2017;21(6):365-71.
863. Xie LQ, Rubin C, Judex S. Enhancement of the adolescent murine musculoskeletal system using low-level mechanical vibrations. JOURNAL OF APPLIED PHYSIOLOGY. 2008;104(4):1056-62.
864. Abbas A, Srinivasan J, Bumstead B, Zarif M, Buhse M, Fafard L, et al. Enhancing multiple sclerosis management: Investigation into the relationship between visual evoked potential latency and digital MRI measurements. Multiple Sclerosis Journal. 2019;25:631-2.
865. Pop PA, Lazar L, Marcu FM, Asme. ENHANCING THE SPORT PERFORMANCE AND QUALITY LIFE OF ATHLETES BY APPLYING OF WBV METHOD. PROCEEDINGS OF THE ASME INTERNATIONAL MECHANICAL ENGINEERING CONGRESS AND EXPOSITION, 2016, VOL 32017.
866. Skovron ML. Epidemiology of low back pain. Baillieres Clin Rheumatol. 1992;6(3):559-73.
867. Shelerud RA. Epidemiology of Occupational Low Back Pain. Clinics in Occupational and Environmental Medicine. 2006;5(3):501-28.
868. Ruzene JRS, Morcelli MH, Navega MT. Equilibrium in women with osteoporosis submitted to balance training with and without an oscillating vibratory pole. JOURNAL OF BODYWORK AND MOVEMENT THERAPIES. 2016;20(1):35-41.
869. Giombini A, Macaluso A, Laudani L, DiCesare A, Piccinini A, Pigozzi F, et al. Erratum...Acute Effect of Whole-Body Vibration at Optimal Frequency on Muscle Power Output of the Lower Limbs in Older Women: Erratum. American Journal of Physical Medicine & Rehabilitation. 2014;93(9):738-.
870. Verver MM, Van Hoof J, Oomens CWJ, Van De Wouw N, Wismans JSHM. Estimation of spinal loading in vertical vibrations by numerical simulation. Clinical Biomechanics. 2003;18(9):800-11.
871. Camacho-Cardenosa M, Camacho-Cardenosa A, Brazo-Sayavera J, Olcina G, Tomas-Carus P, Timón R. Evaluation of 18-Week Whole-Body Vibration Training in Normobaric Hypoxia on Lower Extremity Muscle Strength in an Elderly Population. High altitude medicine & biology. 2019;20(2):157‐64.
872. Evaluation of a six-week whole-body vibration intervention on neuromuscular performance in older adults. Journal of strength and conditioning research / national strength & conditioning association 29 (1) (pp 86-95), 2015 Date of publication: 01 jan 2015. 2015.
873. Solak S, Yesil H, Dündar Ü, Tokta H, Yesil M, Korkmaz M. Evaluation of Balance Exercises on Balance, Fall Risk, and Quality of Life in Postmenopausal Women. TURK OSTEOPOROZ DERGISI-TURKISH JOURNAL OF OSTEOPOROSIS. 2022;28(1):32-40.
874. Yoon J, Kanamori A, Fujii K, Isoda H, Okura T. Evaluation of maslinic acid with whole-body vibration training in elderly women with knee osteoarthritis. PloS one. 2018;13(3):e0194572.
875. Hazell TJ, Kenno KA, Jakobi JM. EVALUATION OF MUSCLE ACTIVITY FOR LOADED AND UNLOADED DYNAMIC SQUATS DURING VERTICAL WHOLE-BODY VIBRATION. JOURNAL OF STRENGTH AND CONDITIONING RESEARCH. 2010;24(7):1860-5.
876. Tofighi A, Saedmocheshi S, Ghafari G. Evaluation of neuromuscular response to chronic whole body vibration training in elderly women. Scientific journal of kurdistan university of medical sciences. 2013;18(3):64‐70.
877. Buckinx F, Beaudart C, Maquet D, Demonceau M, Reginster JY, Bruyère O. Evaluation of the impact of 6-month training by whole body vibration on the risk of falls among nursing home residents, observed over a 12-month period: A single blind, randomized controlled trial. Aging Clinical and Experimental Research. 2014;26(4):369-76.
878. Moreira-Marconi E, Moura-Fernandes MC, Lopes-Souza P, Teixeira-Silva Y, Reis-Silva A, Marchon RM, et al. Evaluation of the temperature of posterior lower limbs skin during the whole body vibration measured by infrared thermography: Cross-sectional study analysis using linear mixed effect model. PLoS ONE. 2019;14(3).
879. Yokota A, Hirao Y. Evaluation of whole-body road traffic vibration in building. Industrial Health. 1998;36(2):120-6.
880. Kaneko C, Hagiwara T, Maeda S. Evaluation of whole-body vibration by the category judgment method. Ind Health. 2005;43(1):221-32.
881. Watson A, Wilkinson TMA, Freeman A. Evidence around the impact of pulmonary rehabilitation and exercise on redox status in COPD: a systematic review. Frontiers in Sports and Active Living 2021 Nov 26;3(782590):Epub. 2021.
882. Belavy DL, Beller G, Armbrecht G, Perschel FH, Fitzner R, Bock O, et al. Evidence for an additional effect of whole-body vibration above resistive exercise alone in preventing bone loss during prolonged bed rest. OSTEOPOROSIS INTERNATIONAL. 2011;22(5):1581-91.
883. Abdel-Aziz K, Schneider T, Solanky BS, Yiannakas MC, Altmann DR, Wheeler-Kingshott CAM, et al. Evidence for early neurodegeneration in the cervical cord of patients with primary progressive multiple sclerosis. Brain. 2015;138(6):1568-82.
884. Reis-Silva A, Coelho-Oliveira AC, Moura-Fernandes MC, Bessa MOB, Batouli-Santos D, Bernardo M, et al. Evidence of whole-body vibration exercises on body composition changes in older individuals: a systematic review and meta-analysis. FRONTIERS IN PHYSIOLOGY. 2023;14.
885. Patterson K, Barker A. Evidence review: Whole body vibration training-Evidence based exercise or fitness fad? Journal of Science and Medicine in Sport. 2010;13:e103-e4.
886. Rudisill SS, Varady NH, Kucharik MP, Eberlin CT, Martin SD. Evidence-Based Hamstring Injury Prevention and Risk Factor Management: A Systematic Review and Meta-analysis of Randomized Controlled Trials. American Journal of Sports Medicine. 2023;51(7):1927-42.
887. Niewiadomski W, Gąsiorowska A, Żyliński M, Karbowniczek A, Cebrat J, Stępniewska A. Exaggerated pressor response to static squats in Parkinson's disease (PD) and healthy subjects is likely an individual trait, not influenced by whole body vibration (WBV). NeuroRehabilitation. 2023;52(2):289-98.
888. Moore SA, Hrisos N, Errington L, Rochester L, Rodgers H, Witham M, et al. Exercise as a treatment for sarcopenia: an umbrella review of systematic review evidence. PHYSIOTHERAPY. 2020;107:189-201.
889. Marques EA, Mota J, Carvalho J. Exercise effects on bone mineral density in older adults: a meta-analysis of randomized controlled trials. AGE. 2012;34(6):1493-515.
890. Davis SA, Davis BL. Exercise Equipment Used in Microgravity: Challenges and Opportunities. CURRENT SPORTS MEDICINE REPORTS. 2012;11(3):142-7.
891. Howe TE, Rochester L, Neil F, Skelton DA, Ballinger C. Exercise for improving balance in older people. COCHRANE DATABASE OF SYSTEMATIC REVIEWS. 2011(11).
892. Williams AD, Bird ML, Hardcastle SGK, Kirschbaum M, Ogden KJ, Walters JAE. Exercise for reducing falls in people living with and beyond cancer. COCHRANE DATABASE OF SYSTEMATIC REVIEWS. 2018(10).
893. Karunaratne A, King A. Exercise interventions for balance in people with diabetic peripheral neuropathy to reduce falls: A systematic review. Physiotherapy (United Kingdom). 2017;103:e77.
894. Karimanasseri C. Exercise Interventions for Osteoporosis in Postmenopausal Women: A Review. Critical Reviews in Physical and Rehabilitation Medicine. 2022;34(3):41-53.
895. Babatunde OO, Bourton AL, Hind K, Paskins Z, Forsyth JJ. Exercise Interventions for Preventing and Treating Low Bone Mass in the Forearm: A Systematic Review and Meta-analysis. ARCHIVES OF PHYSICAL MEDICINE AND REHABILITATION. 2020;101(3):487-511.
896. Koshy FS, George K, Poudel P, Chalasani R, Goonathilake MR, Waqar S, et al. Exercise Prescription and the Minimum Dose for Bone Remodeling Needed to Prevent Osteoporosis in Postmenopausal Women: A Systematic Review. CUREUS JOURNAL OF MEDICAL SCIENCE. 2022;14(6).
897. Bao W, Sun Y, Zhang T, Zou L, Wu X, Wang D, et al. Exercise programs for muscle mass, muscle strength and physical performance in older adults with sarcopenia: A systematic review and meta-analysis. Aging and Disease. 2020;11(4):863-73.
898. Mohebbi R, Shojaa M, Kohl M, von Stengel S, Jakob F, Kerschan-Schindl K, et al. Exercise training and bone mineral density in postmenopausal women: an updated systematic review and meta-analysis of intervention studies with emphasis on potential moderators. OSTEOPOROSIS INTERNATIONAL. 2023;34(7):1145-78.
899. Wu PY, Huang KS, Chen KM, Chou CP, Tu YK. Exercise, Nutrition, and Combined Exercise and Nutrition in Older Adults with Sarcopenia: A Systematic Review and Network Meta-analysis. Maturitas. 2021;145:38-48.
900. Broekmans T, Roelants M, Alders G, Feys P, Thijs H, Eijnde BO. Exploring the effects of a 20-week whole-body vibration training programme on leg muscle performance and function in persons with multiple sclerosis. Journal of rehabilitation medicine. 2010;42(9):866‐72.
901. Pitukcheewanont P, Safani D. Extremely low-level, short-term mechanical stimulation increases cancellous and cortical bone density and muscle mass of children with low bone density - A pilot study. ENDOCRINOLOGIST. 2006;16(3):128-32.
902. Pliner EM, Seo NJ, Beschorner KE. Factors affecting fall severity from a ladder: Impact of climbing direction, gloves, gender and adaptation. APPLIED ERGONOMICS. 2017;60:163-70.
903. Matsumoto Y, Maeda S, Iwane Y, Iwata Y. Factors affecting perception thresholds of vertical whole-body vibration in recumbent subjects: Gender and age of subjects, and vibration duration. Journal of Sound and Vibration. 2011;330(8):1810-28.
904. Huber G, Nagel K, Skrzypiec D, Klein A, Püschel K, Morlock MM. Fatigue failure of lumbar functional spinal units caused by high cyclic axial loading. Journal of Biomechanics. 2012;45:S615.
905. Hamrick MW, McGee-Lawrence ME, Frechette DM. Fatty Infiltration of Skeletal Muscle: Mechanisms and Comparisons with Bone Marrow Adiposity. Frontiers in Endocrinology. 2016;7.
906. Kitamoto T, Saegusa R, Tashiro T, Sakurai T, Yokote K, Tokuyama T. Favorable Effects of 24‐Week Whole-Body Vibration on Glycemic Control and Comprehensive Diabetes Therapy in Elderly Patients with Type 2 Diabetes. Diabetes Therapy. 2021;12(6):1751-61.
907. Rogan S, Radlinger L, Hilfiker R, Schmidtbleicher D, de Bie RA, de Bruin ED. Feasibility and effects of applying stochastic resonance whole-body vibration on untrained elderly: a randomized crossover pilot study. BMC Geriatrics 2015 Mar 12;15(25):Epub. 2015.
908. Petryk A, Polgreen LE, Grames M, Lowe DA, Hodges JS, Karachunski P. Feasibility and tolerability of whole-body, low-intensity vibration and its effects on muscle function and bone in patients with dystrophinopathies: a pilot study. Muscle & Nerve. 2017;55(6):875-83.
909. Heumann K, Swan PD. Feasibility of measuring acute changes in Os Calcis Stiffness Index following whole-body vibration with resistance and jump training in young women. Osteoporosis International. 2010;21:S229.
910. Genest F, Lindström S, Scherer S, Schneider M, Seefried L. Feasibility of simple exercise interventions for men with osteoporosis-A prospective randomized controlled pilot study. BONE REPORTS. 2021;15.
911. Heesterbeek M, van der Zee EA, van Heuvelen MJG. Feasibility of Three Novel Forms of Passive Exercise in a Multisensory Environment in Vulnerable Institutionalized Older Adults with Dementia. Journal of Alzheimer's disease. 2019;70(3):681‐90.
912. Miyara K, Matsumoto S, Uema T, Hirokawa T, Noma T, Shimodozono M, et al. Feasibility of using whole body vibration as a means for controlling spasticity in post-stroke patients: a pilot study. Complement Ther Clin Pract. 2014;20(1):70-3.
913. Pahl A, Wehrle A, Kneis S, Gollhofer A, Bertz H. Feasibility of whole body vibration during intensive chemotherapy in patients with hematological malignancies - a randomized controlled pilot study. BMC Cancer. 2018;18(1):1-12.
914. Bautmans I, Van Hees E, Lemper JC, Mets T. The feasibility of Whole Body Vibration in institutionalised elderly persons and its influence on muscle performance, balance and mobility: a randomised controlled trial. BMC geriatrics. 2005;5:17.
915. Brunner S, Brunner D, Winter H, Kneidinger N. Feasibility of whole-body vibration as an early inpatient rehabilitation tool after lung transplantation--a pilot study. Clin Transplant. 2016;30(2):93-8.
916. Sievänen H, Karinkanta S, Moisio-Vilenius P, Ripsaluoma J. Feasibility of whole-body vibration training in nursing home residents with low physical function: a pilot study. Aging Clinical & Experimental Research. 2014;26(5):511-7.
917. Windberger U, Grohmann K, Goll A, Plasenzotti R, Losert U. Fetal and juvenile animal hemorheology. Clin Hemorheol Microcirc. 2005;32(3):191-7.
918. Pinto ND, Monteiro MDB, Paiva DN, Santos SD, Misssailidis S, Thompson D, et al. Fibromyalgia and the Relevance of the Whole-Body Vibration Exercises in Vibratory Platforms: A Short Review. BRAZILIAN ARCHIVES OF BIOLOGY AND TECHNOLOGY. 2012;55(1):61-7.
919. Xu M, Yang J, Lieberman I, Haddas R. Finite element method-based study for effect of adult degenerative scoliosis on the spinal vibration characteristics. Comput Biol Med. 2017;84:53-8.
920. Raimundo AM, Gusi N, Tomas-Carus P. Fitness efficacy of vibratory exercise compared to walking in postmenopausal women. European journal of applied physiology. 2009;106(5):741‐8.
921. Danna-dos-Santos A, Degani AM, Latash ML. Flexible muscle modes and synergies in challenging whole-body tasks. Experimental Brain Research. 2008;189(2):171-87.
922. Paolucci T, Pezzi L, La Verde R, Latessa PM, Bellomo RG, Saggini R. The Focal Mechanical Vibration for Balance Improvement in Elderly-A Systematic Review. CLINICAL INTERVENTIONS IN AGING. 2021;16:2009-21.
923. Brunetti O, Botti FM, Roscini M, Brunetti A, Panichi R, Filippi GM, et al. Focal vibration of quadriceps muscle enhances leg power and decreases knee joint laxity in female volleyball players. JOURNAL OF SPORTS MEDICINE AND PHYSICAL FITNESS. 2012;52(6):596-605.
924. Ferrario M, Moissl U, Garzotto F, Cruz DN, Tetta C, Signorini MG, et al. The forgotten role of central volume in low frequency oscillations of heart rate variability. PLoS ONE. 2015;10(3).
925. Ozcan Cetin EH, Cetin MS, Canpolat U, Kalender E, Topaloglu S, Aras D, et al. The Forgotten Variable of Shear Stress in Mitral Annular Calcification: Whole Blood Viscosity. Med Princ Pract. 2015;24(5):444-50.
926. Warden SJ, Dick A, Simon JE, Manini TM, Russ DW, Lyssikatos C, et al. Fracture discrimination capability of ulnar flexural rigidity measured via Cortical Bone Mechanics Technology: Study protocol for the STRONGER Study. JBMR Plus. 2024;8(1).
927. von Stengel S, Kemmler W. Fracture reduction through physical training - Which training for whom? An evidence-based overview of training strategies for osteoporosis. OSTEOLOGIE. 2020;29(03):207-14.
928. Maikala RV, Bhambhani YN. Functional changes in cerebral and paraspinal muscle physiology of healthy women during exposure to whole-body vibration. Accident Analysis and Prevention. 2008;40(3):943-53.
929. Simao AP, Avelar NC, Tossige-Gomes R, Neves CD, Mendonça VA, Miranda AS, et al. Functional Performance and Inflammatory Cytokines After Squat Exercises and Whole-Body Vibration in Elderly Individuals With Knee Osteoarthritis. ARCHIVES OF PHYSICAL MEDICINE AND REHABILITATION. 2012;93(10):1692-700.
930. Manthou M, Nohroudi K, Moscarino S, Rehberg F, Stein G, Jansen R, et al. Functional recovery after experimental spinal cord compression and whole body vibration therapy requires a balanced revascularization of the injured site. Restor Neurol Neurosci. 2015;33(2):233-49.
931. Yu CH, Kang SR, Kwon TK, editors. Fundamental study of lower limb muscle activity using an angled whole body vibration exercise instrument. Bio-Medical Materials and Engineering; 2014.
932. Godinez A, Liston DB, Ayzenberg R, Toscano WB, Cowings PA, Stone LS. G-loading and vibration effects on heart and respiration rates. Aviat Space Environ Med. 2014;85(9):949-53.
933. Dewangan KN, Rakheja S, Marcotte P. Gender and anthropometric effects on whole-body vibration power absorption of the seated body. Journal of Low Frequency Noise Vibration and Active Control. 2018;37(2):167-90.
934. Shibata N, Maeda S, Vdi. Gender difference in psychological evaluation of subjective responses to different-axis whole-body vibration. Human Vibration: Effects on Health - Performance - Comfort2007. p. 425-31.
935. Shibata N, Ishimatsu K, Maeda S. Gender difference in subjective response to whole-body vibration under standing posture. International Archives of Occupational and Environmental Health. 2012;85(2):171-9.
936. Bogar L, Juricskay I, Kesmarky G, Feher G, Kenyeres P, Toth K. Gender differences in hemorheological parameters of coronary artery disease patients. Clin Hemorheol Microcirc. 2006;35(1-2):99-103.
937. Sañudo B, Feria A, Carrasco L, De Hoyo M, Santos R, Gamboa H. Gender differences in knee stability in response to whole-body vibration. Journal of Strength and Conditioning Research. 2012;26(8):2156-65.
938. Ross AE, Flaa A, Høieggen A, Reims H, Eide IK, Kjeldsen SE. Gender specific sympathetic and hemorrheological responses to mental stress in healthy young subjects. Scand Cardiovasc J. 2001;35(5):307-12.
939. He L, Roie EV, Bogaerts A, Verschueren S, Delecluse C, Morse CI, et al. The genetic effect on muscular changes in an older population: A follow-up study after one-year cessation of structured training. Genes. 2020;11(9):1-15.
940. He L, Van Roie E, Bogaerts A, Morse CI, Delecluse C, Verschueren S, et al. Genetic predisposition score predicts the increases of knee strength and muscle mass after one-year exercise in healthy elderly. Experimental gerontology. 2018;111:17‐26.
941. Sartorio A, Agosti F, Patrizi A, Gattico A, Tringali G, Giunta M, et al. GH and cortisol responses following an acute session of respiratory muscle endurance training in severely obese patients. Horm Metab Res. 2013;45(3):239-44.
942. Wang L, Jiang J, Li Y, Huang J, Wang R, Liang Y, et al. Global trends and hotspots in research on osteoporosis rehabilitation: A bibliometric study and visualization analysis. Frontiers in public health. 2022;10:1022035.
943. Actrn. Good Vibrations Study: a comparison of whole body vibration exposure using locked or soft knees in older adults. https://trialsearchwhoint/Trial2aspx?TrialID=ACTRN12609000353291. 2009.
944. Fuermaier AB, Tucha L, Koerts J, van Heuvelen MJ, van der Zee EA, Lange KW, et al. Good vibrations--effects of whole body vibration on attention in healthy individuals and individuals with ADHD. PLoS One. 2014;9(2):e90747.
945. Mueller S, Fischer M, Herger S, Nüesch C, Egloff C, Itin P, et al. Good vibrations: Itch induction by whole body vibration exercise without the need of a pruritogen. Exp Dermatol. 2019;28(12):1390-6.
946. Drks. „Good vibrations“ Efficiency of vibration to treat patellar tendinopathy. https://trialsearchwhoint/Trial2aspx?TrialID=DRKS00011338. 2016.
947. Dercon Q, Nicholas JM, James SN, Schott JM, Richards M. Grip strength from midlife as an indicator of later-life brain health and cognition: evidence from a British birth cohort. BMC Geriatrics. 2021;21(1).
948. Dercon Q, Nicholas J, James SN, Schott J, Richards M. Grip strength from midlife as an indicator of later-life cognition and brain health: Evidence from a British birth cohort. Age and Ageing. 2021;50(SUPPL 2).
949. Giunta M, Cardinale M, Agosti F, Patrizi A, Compri E, Rigamonti AE, et al. Growth hormone-releasing effects of whole body vibration alone or combined with squatting plus external load in severely obese female subjects. Obes Facts. 2012;5(4):567-74.
950. Wiek J, Schade M, Wiederholt M, Arntz HR, Hansen LL. Haemorheological changes in patients with retinal vein occlusion after isovolaemic haemodilution. Br J Ophthalmol. 1990;74(11):665-9.
951. Wiek J, Krause M, Schade M, Wiederholt M, Hansen LL. Haemorheological parameters in patients with retinal artery occlusion and anterior ischaemic optic neuropathy. Br J Ophthalmol. 1992;76(3):142-5.
952. Awodu OA, Ajayi IO, Famodu AA. Haemorheological variables in Nigeria pulmonary tuberculosis patients undergoing therapy. Clin Hemorheol Microcirc. 2007;36(4):267-75.
953. Biancalana V, Lucchetti C, Boemi M. Hba1c: miglior controllo con meno tempo d'esercizio fisico nei diabetici over 65 anni (Hba1c: best control with shorter exercise for type 2 diabetes patients over 65 years) Giornale Italiano di Diabetologia e Metabolismo 2018 Mar;38(1):33-39. 2018.
954. Kct. Health benefits of whole-body vibration exercise in women with obesity. https://trialsearchwhoint/Trial2aspx?TrialID=KCT0009128. 2024.
955. Garcia-Mendez Y, Pearlman JL, Boninger ML, Cooper RA. Health risks of vibration exposure to wheelchair users in the community. J Spinal Cord Med. 2013;36(4):365-75.
956. Medeiros AM, Assunção A, Santos JN. [Hearing loss in urban transportation workers in Greater Metropolitan Belo Horizonte, Minas Gerais State, Brazil]. Cad Saude Publica. 2015;31(9):1953-63.
957. Vékási J, Márton Z, Késmárky G, Cser A, Russai R, Kovács B. [Hemorheologic factors in hypertensive and diabetic retinopathy]. Orv Hetil. 2001;142(20):1045-8.
958. Késmárky G, Tóth K, Habon L, Vajda G, Juricskay I. [Hemorheologic parameters in coronary artery disease]. Orv Hetil. 1998;139(24):1465-8.
959. Fatini C, Mannini L, Sticchi E, Rogai V, Guiducci S, Conforti ML, et al. Hemorheologic profile in systemic sclerosis: role of NOS3 -786T > C and 894G >T polymorphisms in modulating both the hemorheologic parameters and the susceptibility to the disease. Arthritis Rheum. 2006;54(7):2263-70.
960. Yi JE, Youn HJ. Hemorheological abnormalities and their associations with coronary blood flow in patients with cardiac syndrome X: a comparison between males and females. Perfusion. 2017;32(1):57-67.
961. Gal R, Praksch D, Kenyeres P, Rabai M, Toth K, Halmosi R, et al. Hemorheological Alterations in Patients with Heart Failure with Reduced Ejection Fraction Treated by Resveratrol. Cardiovasc Ther. 2020;2020:7262474.
962. Toth K, Kesmarky G, Vekasi J, Nemes J, Czopf L, Kapronczay P, et al. Hemorheological and hemodynamic parameters in patients with essential hypertension and their modification by alpha-1 inhibitor drug treatment. Clin Hemorheol Microcirc. 1999;21(3-4):209-16.
963. Mangalani M, Lokeshwar MR, Banerjee R, Nageswari K, Puniyani RR. Hemorheological changes in blood transfusion-treated beta thalassemia major patients. Clin Hemorheol Microcirc. 1998;18(2-3):99-102.
964. Velcheva I, Antonova N, Titianova E, Damianov P, Dimitrov N, Dimitrova V. Hemorheological disturbances in cerebrovascular diseases. Clin Hemorheol Microcirc. 2008;39(1-4):391-6.
965. Santos MJ, Pedro LM, Canhão H, Fernandes EFJ, Canas da Silva J, Fonseca JE, et al. Hemorheological parameters are related to subclinical atherosclerosis in systemic lupus erythematosus and rheumatoid arthritis patients. Atherosclerosis. 2011;219(2):821-6.
966. Clivillé X, Bofill C, Joven J, Monasterio J, Viscor G, Vernis M, et al. Hemorheological, coagulative and fibrinolytic changes during autologous blood donation. Clin Hemorheol Microcirc. 1998;18(4):265-72.
967. Velcheva I, Damianov P, Mantarova S, Antonova N. Hemorheology and heart rate variability in patients with diabetes mellitus type 2. Clinical Hemorheology and Microcirculation. 2011;49(1-4):513-8.
968. Gyawali P, Richards RS, Tinley P, Nwose EU. Hemorheology, ankle brachial pressure index (ABPI) and toe brachial pressure index (TBPI) in metabolic syndrome. Microvascular Research. 2014;95(1):31-6.
969. Ma R, Zhu D, Gong H, Gu G, Huang X, Gao J, et al. High-frequency and low-magnitude whole body vibration with rest days is more effective in improving skeletal micro-morphology and biomechanical properties in ovariectomised rodents. Hip Int. 2012;22(2):218-26.
970. Chatterjee M, Hatori K, Duyck J, Sasaki K, Naert I, Vandamme K. High-frequency loading positively impacts titanium implant osseointegration in impaired bone. Osteoporosis International. 2015;26(1):281-90.
971. Cardinale M. High-frequency vibration training able to increase muscle power in postmenopausal women. ARCHIVES OF PHYSICAL MEDICINE AND REHABILITATION. 2004;85(4):687-8.
972. Russo CR, Lauretani F, Bandinelli S, Bartali B, Cavazzini C, Guralnik JM, et al. High-frequency vibration training increases muscle power in postmenopausal women. ARCHIVES OF PHYSICAL MEDICINE AND REHABILITATION. 2003;84(12):1854-7.
973. Kalaoǧlu E, Bucak ÖF, Kökçe M, Özkan M, Çetin M, Atasoy M, et al. High-frequency whole-body vibration activates tonic vibration reflex. Turkish Journal of Physical Medicine and Rehabilitation. 2023;69(1):46-51.
974. Cheung WH, Mok HW, Qin L, Sze PC, Lee KM, Leung KS. High-frequency whole-body vibration improves balancing ability in elderly women. Archives of Physical Medicine and Rehabilitation 2007 Jul;88(7):852-857. 2007.
975. Reyes ML, Hernández M, Holmgren LJ, Sanhueza E, Escobar RG. High-Frequency, Low-Intensity Vibrations Increase Bone Mass and Muscle Strength in Upper Limbs, Improving Autonomy in Disabled Children. JOURNAL OF BONE AND MINERAL RESEARCH. 2011;26(8):1759-66.
976. Manske SL, Good CA, Zernicke RF, Boyd SK. High-Frequency, Low-Magnitude Vibration Does Not Prevent Bone Loss Resulting from Muscle Disuse in Mice following Botulinum Toxin Injection. PLOS ONE. 2012;7(5).
977. De Nardi M, Facheris C, Ruggeri P, La Torre A, Codella R. High-impact Routines to Ameliorate Trunk and Lower Limbs Flexibility in Women. INTERNATIONAL JOURNAL OF SPORTS MEDICINE. 2020;41(14):1039-46.
978. Bhat SZ, Goyal I, Chaudhuri A. Hollow Bones: A Case Report of Immobilization Osteoporosis in a Young Female. Journal of the Endocrine Society. 2020;4:A34-A5.
979. Langensiepen S, Stark C, Sobottke R, Semler O, Franklin J, Schraeder M, et al. Home-based vibration assisted exercise as a new treatment option for scoliosis -- a randomised controlled trial. Journal of Musculoskeletal & Neuronal Interactions 2017 Dec;17(4):259-267. 2017.
980. Cardinale M, Soiza RL, Leiper JB, Gibson A, Primrose WR. Hormonal responses to a single session of whole-body vibration exercise in older individuals. BRITISH JOURNAL OF SPORTS MEDICINE. 2010;44(4):284-8.
981. Jalowiec KA, Pabst T, Bocksrucker C, Brunold C, Jeker B, Baerlocher GM, et al. How to Collect the Minimum-Targeted CD3+ Cells for CAR-T Therapy- the Bern Approach. Blood. 2019;134:2457.
982. Pleguezuelos E, Casarramona P, Guirao L, Samitier B, Ortega P, Vila X, et al. How whole-body vibration can help our COPD patients. Physiological changes at different vibration frequencies. International Journal of COPD. 2018;13:3373-80.
983. Pekesen Kurtça M, Baş Aslan U, Kuyucu E. HPR a comparison the effectiveness of wholebody vibration, progressive resistive exercise and home-based exercise in patients with knee osteoarthr it is. Annals of the Rheumatic Diseases. 2017;76:1484.
984. Sonza A, Maurer C, Achaval M, Zaro MA, Nigg BM. Human cutaneous sensors on the sole of the foot: altered sensitivity and recovery time after whole body vibration. Neurosci Lett. 2013;533:81-5.
985. Alshabi M, Nawayseh N, Bettayeb M. Hybrid artificial genetic - neural network model to predict the transmission of vibration to the head during whole-body vibration training. JOURNAL OF VIBROENGINEERING. 2020;22(3):705-20.
986. Krutki P, Mrówczyński W, Celichowski J, Bączyk M. Ia EPSPs in rat spinal motoneurons are potentiated after a 5-week whole body vibration. Journal of Applied Physiology (Bethesda, Md : 1985). 2022;132(1):178-86.
987. Komrakova M, Sehmisch S, Tezval M, Ammon J, Lieberwirth P, Sauerhoff C, et al. Identification of a vibration regime favorable for bone healing and muscle in estrogen-deficient rats. Calcif Tissue Int. 2013;92(6):509-20.
988. Azizi M, Shadmehr A, Malmir K, Qotbi N, Khazaei Z. The Immediate Effect of Muscle Energy Technique and Whole Body Vibration on Hamstring Muscle Flexibility and Stiffness in Healthy Young Females. MLTJ-MUSCLES LIGAMENTS AND TENDONS JOURNAL. 2021;11(3):409-15.
989. Pamukoff DN, Pietrosimone B, Lewek MD, Ryan ED, Weinhold PS, Lee DR, et al. Immediate effect of vibratory stimuli on quadriceps function in healthy adults. Muscle & nerve. 2016;54(3):469‐78.
990. Karim A, Roddey T, Mitchell K, Ortiz A, Olson S. Immediate effect of whole body vibration on saute height and balance in female professional contemporary dancers a randomized controlled trial. Journal of Dance Medicine & Science 2019 Mar;23(1):3-10. 2019.
991. Shargh MH, Aminian-Far A, Mirmohammadkhani M. (Immediate effect of whole body vibration on trunk proprioception in non-specific chronic low back pain). Koomesh 2020 Spring;22(2):282-290. 2020.
992. Taghizadeh Delkhoush C, Bagheri R, Mashhadi Hashemi H, Fatemy E, Hedayati R. The immediate effect of whole body vibration training on the electromyographic activity of contralateral hand muscles; a randomized controlled trial. Journal of Bodywork & Movement Therapies. 2020;24(3):293-9.
993. Gonçalves AF, Matias FL, Parizotto NA, dos Santos HH, Ferreira JJDA, de Andrade PR. The immediate effect of whole-body vibration on rectus abdominis muscle activity and cutaneous temperature: A randomized controlled trial. Journal of Bodywork and Movement Therapies. 2021;25:46-52.
994. Rogan S, Schmidtbleicher D, Radlinger L. Immediate effects after stochastic resonance whole-body vibration on physical performance on frail elderly for skilling-up training: a blind cross-over randomised pilot study. AGING CLINICAL AND EXPERIMENTAL RESEARCH. 2014;26(5):519-27.
995. Nantakool S, Punturee K, Konghakote S, Sitthichoke C, Phirom K, Chuatrakoon B. Immediate Effects of Aerobic Exercise and Whole-Body Vibration on Fat Oxidation, Lipid Mobilization, and Cardiovascular Response in Individuals with Obesity. J Clin Med. 2023;13(1).
996. Ozvar GB, Ayvat E, Kilinc M. Immediate effects of local vibration and whole-body vibration on postural control in patients with ataxia: an assessor-blind, cross-over randomized trial. The Cerebellum 2021 Feb;20(1):83-91. 2021.
997. Dickerson C, Gabler G, Hopper K, Kirk D, McGregor CJ. IMMEDIATE EFFECTS OF LOCALIZED VIBRATION ON HAMSTRING AND QUADRICEP MUSCLE PERFORMANCE. INTERNATIONAL JOURNAL OF SPORTS PHYSICAL THERAPY. 2012;7(4):381-7.
998. Salami A, Roostayi MM, Naimi SS, Shadmehr A, Baghban AA. The immediate effects of whole body vibration on cervical joint position sense in subjects with forward head posture. MLTJ-MUSCLES LIGAMENTS AND TENDONS JOURNAL. 2018;8(2):255-60.
999. Nct. Immediate Effects of Whole Body Vibration on Neuromuscular Performance of Quadriceps Femoris and Balance. https://clinicaltrialsgov/show/NCT02416362. 2015.
1000. Rieder F, Wiesinger HP, Kösters A, Müller E, Seynnes OR. Immediate effects of whole body vibration on patellar tendon properties and knee extension torque. European journal of applied physiology. 2016;116(3):553‐61.
1001. de Paula FA, Mendonca VA, Lage V, da Silva GP, de Almeida HC, Lima LP, et al. Immediate effects of whole-body vibration associated with squatting exercises on hemodynamic parameters in sarcopenic older people: a randomized controlled trial. International Journal of Environmental Research & Public Health 2021 Nov;18(22):11852. 2021.
1002. Saldiran T, Mutluay FK. Immediate effects of whole-body vibration exposure on perceived exertion and peripheral hemodynamics in overweight and obese individuals. FATIGUE-BIOMEDICINE HEALTH AND BEHAVIOR. 2020;8(4):214-25.
1003. Borges DT, Macedo LB, Lins CAA, Brasileiro JS. Immediate effects of whole-body vibration on neuromuscular performance of quadriceps and oscillation of the center of pressure: A randomized controlled trial. Manual Therapy. 2016;25:62-8.
1004. Çevik Saldiran T, Azim Rezaei D, Atici E, Öztürk Ö, Okudan B, Akgül B, et al. Immediate responses of the different whole body vibration training frequencies on muscles properties. Turkish Journal of Physiotherapy and Rehabilitation. 2019;30(2):S48-S9.
1005. Shirolapov I, Pyatin V, Zhestkov A, Nikitin O, Alekseeva A, Merkulova S. Immune responses to 6-month whole body vibration (acceleration) training in elderly women: A pilot study. Allergy: European Journal of Allergy and Clinical Immunology. 2011;66:534.
1006. Wunram HL, Oberste M, Hamacher S, Neufang S, Grote N, Krischer MK, et al. Immunological Effects of an Add-On Physical Exercise Therapy in Depressed Adolescents and Its Interplay with Depression Severity. Int J Environ Res Public Health. 2021;18(12).
1007. Cevik Saldiran T, Mutluay FK, Yagci I, Yilmaz Y. Impact of aerobic training with and without whole-body vibration training on metabolic features and quality of life in non-alcoholic fatty liver disease patients. Annales d'Endocrinologie 2020 Oct;81(5):493-499. 2020.
1008. Raina AK, Baheti M, Haldar A, Ramulu M, Chakraborty AK, Sahu PB, et al. Impact of blast induced transitory vibration and air-overpressure/noise on human brain--an experimental study. Int J Environ Health Res. 2004;14(2):143-9.
1009. Xu ZJ, Wang HW, Shi Y, Shen QM, Tsamlag L, Wang ZZ, et al. Impact of calcium, vitamin D, vitamin K, oestrogen, isoflavone and exercise on bone mineral density for osteoporosis prevention in postmenopausal women: a network meta-analysis. BRITISH JOURNAL OF NUTRITION. 2020;123(1):84-103.
1010. Rahimi GRM, Smart NA, Liang MTC, Bijeh N, Albanaqi AL, Fathi M, et al. The Impact of Different Modes of Exercise Training on Bone Mineral Density in Older Postmenopausal Women: A Systematic Review and Meta-analysis Research. CALCIFIED TISSUE INTERNATIONAL. 2020;106(6):577-90.
1011. Figueroa A, Alvarez-Alvarado S, Ormsbee MJ, Madzima TA, Campbell JC, Wong A. Impact of L-citrulline supplementation and whole-body vibration training on arterial stiffness and leg muscle function in obese postmenopausal women with high blood pressure. Experimental gerontology. 2015;63:35‐40.
1012. Ciematnieks U, Poda J. IMPACT OF LOCAL VIBROSTIMULATION ON PARAMETERS OF LEG MUSCLES STRENGTH ENDURANCE. SOCIETY, INTEGRATION, EDUCATION, VOL IV: SPORTS AND HEALTH - ART AND DESIGN2019. p. 84-90.
1013. Jaime SJ, Maharaj A, Alvarez-Alvarado S, Figueroa A. Impact of low-intensity resistance and whole-body vibration training on aortic hemodynamics and vascular function in postmenopausal women. Hypertension research. 2019;42(12):1979‐88.
1014. Wehrle E, Liedert A, Heilmann A, Wehner T, Bindl R, Fischer L, et al. The impact of low-magnitude high-frequency vibration on fracture healing is profoundly influenced by the oestrogen status in mice. Dis Model Mech. 2015;8(1):93-104.
1015. Boito SM, Struijk PC, Pop GA, Visser W, Steegers EA, Wladimiroff JW. The impact of maternal plasma volume expansion and antihypertensive treatment with intravenous dihydralazine on fetal and maternal hemodynamics during pre-eclampsia: a clinical, echo-Doppler and viscometric study. Ultrasound Obstet Gynecol. 2004;23(4):327-32.
1016. Carbon NM, Engelhardt LJ, Wollersheim T, Grunow JJ, Spies CD, Märdian S, et al. Impact of protocol-based physiotherapy on insulin sensitivity and peripheral glucose metabolism in critically ill patients. Journal of Cachexia, Sarcopenia and Muscle. 2022;13(2):1045-53.
1017. Debenedictis TA, Billing D, Milanese S, Furnell A, Tomkinson G, Thewlis D. The impact of the mechanical whole-body vibration experienced during military land transit on the physical attributes underpinning dismounted combatant physical performance: a randomised controlled trial. Journal of science and medicine in sport. 2021;24(4):380‐5.
1018. Reis-Silva A, Coelho-Oliveira AC, Martins-Anjos E, Moura-Fernandes MC, Mulder A, Xavier VL, et al. Impact of Two Whole-Body Vibration Exercise Protocols on Body Composition of Patients with Metabolic Syndrome: A Randomized Controlled Trial. INTERNATIONAL JOURNAL OF ENVIRONMENTAL RESEARCH AND PUBLIC HEALTH. 2023;20(1).
1019. Arjunan SP, Kumar DK. Impact of vibration on the muscle endurance and fatigue during strengthening exercise. International Journal of Medical Engineering and Informatics. 2015;7(2):167-74.
1020. Kostyshyn NM, Swietlicka I, Tomaszewska E, Dobrowolski P, Muszynski S. Impact of Whole Body Vibration and Zoledronic Acid on Femoral Structure after Ovariectomy: Morphological Evaluation. JOURNAL OF CLINICAL MEDICINE. 2022;11(9).
1021. Seefried L, Strömsdörfer J, Genest F, Engelmann B, Lapa C, Baumann F, et al. Impact of whole body vibration exercise on bone turnover and physical performance in patients with monoclonal gammopathy of undetermined significance. Oncology Research and Treatment. 2019;42:191.
1022. Dudoniene V, Sakaliene R, Svediene L, Kazlauskiene D, Szczegielniak J, Krutulyte G. Impact of whole body vibration on balance improvement in elderly women. JOURNAL OF VIBROENGINEERING. 2013;15(3):1112-8.
1023. Hebbel H, Dalferth R, Sallach N, Maass N, Letsch A, Schmidt T. The impact of whole body vibration on the incidence and the severity of chemotherapy-induced fatigue and quality of life in breast carcinoma patients receiving paclitaxel therapy. Oncology research and treatment. 2022;45:240.
1024. Alp A, Efe B, Korukluoğlu M, Bilgiç A, Demir Türe S, Coşkun Ş, et al. The Impact of Whole Body Vibration Therapy on Spasticity and Disability of the Patients with Poststroke Hemiplegia. Rehabilitation Research & Practice. 2018:1-6.
1025. Seefried L, Genest F, Strömsdörfer J, Engelmann B, Lapa C, Jakob F, et al. Impact of whole-body vibration exercise on physical performance and bone turnover in patients with monoclonal gammopathy of undetermined significance. JOURNAL OF BONE ONCOLOGY. 2020;25.
1026. Li SQ, Yu WB, Li W, Wang JC, Gao LL, Li SM. The Impact of Whole-Body Vibration Training on Bone Minerals and Lean Mass in Children and Adolescents with Motor Disabilities: A Systematic Review and Meta-Analysis. CHILDREN-BASEL. 2022;9(2).
1027. Dalferth R, Hebbel H, Sallach N, Maass N, Letsch A, Schmidt T. The impact of whole-body vibration training on the incidence and the severity of chemotherapy-induced peripheral neuropathy in breast cancer patients receiving paclitaxel therapy. Oncology research and treatment. 2022;45:240.
1028. Bogaerts A, Delecluse C, Claessens AL, Coudyzer W, Boonen S, Verschueren SM. Impact of whole-body vibration training versus fitness training on muscle strength and muscle mass in older men: a 1-year randomized controlled trial. Journals of gerontology Series A, Biological sciences and medical sciences. 2007;62(6):630‐5.
1029. Gonçalves de Oliveira R, Coutinho HMEL, Martins MNM, Bernardo-Filho M, de Sá-Caputo DDC, Campos de Oliveira L, et al. Impacts of Whole-Body Vibration on Muscle Strength, Power, and Endurance in Older Adults: A Systematic Review and Meta-Analysis. Journal of Clinical Medicine. 2023;12(13).
1030. Castrogiovanni P, Trovato FM, Szychlinska MA, Nsir H, Imbesi R, Musumeci G. The importance of physical activity in osteoporosis. From the molecular pathways to the clinical evidence. Histology and histopathology. 2016;31(11):1183-94.
1031. Althoff I, Brinckmann P, Frobin W, Sandover J, Burton K. An improved method of stature measurement for quantitative determination of spinal loading. Application to sitting postures and whole body vibration. Spine (Phila Pa 1976). 1992;17(6):682-93.
1032. Cheung WH, Li CY, Zhu TY, Leung KS. Improvement in muscle performance after one-year cessation of low-magnitude high-frequency vibration in community elderly. JOURNAL OF MUSCULOSKELETAL & NEURONAL INTERACTIONS. 2016;16(1):4-11.
1033. Tezval M, Biblis M, Sehmisch S, Schmelz U, Kolios L, Rack T, et al. Improvement of femoral bone quality after low-magnitude, high-frequency mechanical stimulation in the ovariectomized rat as an osteopenia model. Calcified Tissue International. 2011;88(1):33-40.
1034. Rippetoe J, Wang HW, James SA, Dionne C, Block B, Beckner M. Improvement of Gait after 4 Weeks of Wearable Focal Muscle Vibration Therapy for Individuals with Diabetic Peripheral Neuropathy. JOURNAL OF CLINICAL MEDICINE. 2020;9(11).
1035. Komrakova M, Rechholtz C, Hoffmann D, Wicke M, Sehmisch S. Improvement of muscle tissue properties in ovariectomized rats undergoing anti-osteoporosis therapies. Osteologie. 2017;26(1):A23.
1036. Liu XF, Gao XH, Tong J, Yu LY, Xu ML, Zhang JB. Improvement of Osteoporosis in Rats With Hind-Limb Unloading Treated With Pulsed Electromagnetic Field and Whole-Body Vibration. PHYSICAL THERAPY. 2022;102(10).
1037. Filippi GM, Brunetti O, Botti FM, Panichi R, Roscini M, Camerota F, et al. Improvement of Stance Control and Muscle Performance Induced by Focal Muscle Vibration in Young-Elderly Women: A Randomized Controlled Trial. ARCHIVES OF PHYSICAL MEDICINE AND REHABILITATION. 2009;90(12):2019-25.
1038. Zheng J, Wang X, Li H, Gu Y, Tu P, Wen Z. Improving abnormal hemorheological parameters in ApoE-/- mice by Ilex kudingcha total saponins. Clin Hemorheol Microcirc. 2009;42(1):29-36.
1039. Mahieu NN, Witvrouw E, Van de Voorde D, Michilsens D, Arbyn V, Van den Broecke W. Improving strength and postural control in young skiers: whole-body vibration versus equivalent resistance training. Journal of Athletic Training (National Athletic Trainers' Association). 2006;41(3):286-93.
1040. Prioreschi A, Makda MA, Tikly M, McVeigh JA. In patients with established RA, positive effects of a randomised three month WBV therapy intervention on functional ability, bone mineral density and fatigue are sustained for up to six months. PloS ONE 2016 Apr;11(4):e0153470. 2016.
1041. Giacomin JA, Gallo S. In-vehicle vibration study of child safety seats. Ergonomics. 2003;46(15):1500-12.
1042. von Stengel S, Kemmler W. Increase of bone strength by Whole Body Vibration Training. OSTEOLOGIE. 2015;24(1):30-41.
1043. Shin DW, Gu JY, Kim JS, Jung JS, Shin DY, Koh Y, et al. Increased plasma viscosity in plasma cell dyscrasia and whole blood viscosity in polycythemia vera. Clin Hemorheol Microcirc. 2018;70(1):59-67.
1044. Xie C, Tang B, Wu K, Meng Q, Wang F. Increased serum LOXL2 concentration in pelvic inflammatory disease with pelvic adhesion. BMC Womens Health. 2022;22(1):59.
1045. Yu KJ, Zhang MJ, Li Y, Wang RT. Increased whole blood viscosity associated with arterial stiffness in patients with non-alcoholic fatty liver disease. J Gastroenterol Hepatol. 2014;29(3):540-4.
1046. Li RY, Cao ZG, Li Y, Wang RT. Increased whole blood viscosity is associated with silent cerebral infarction. Clin Hemorheol Microcirc. 2015;59(4):301-7.
1047. Matsumoto T, Sato D, Hashimoto Y. Individual and combined effects of noise-like whole-body vibration and parathyroid hormone treatment on bone defect repair in ovariectomized mice. Proc Inst Mech Eng H. 2016;230(1):30-8.
1048. Carlucci F, Felici F, Piccinini A, Haxhi J, Sacchetti M. Individual Optimal Frequency in Whole-Body Vibration: Effect of Protocol, Joint Angle, and Fatiguing Exercise. Journal of strength and conditioning research. 2016;30(12):3503-11.
1049. Ribeiro VGC, Mendonça VA, Souza ALC, Fonseca SF, Camargos ACR, Lage VKS, et al. Inflammatory biomarkers responses after acute whole body vibration in fibromyalgia. Braz J Med Biol Res. 2018;51(4):e6775.
1050. Wilson SJ, Williams CC, Gdovin JR, Eason JD, 2nd, Luginsland LA, Hill CM, et al. The Influence of an Acute Bout of Whole Body Vibration on Human Postural Control Responses. J Mot Behav. 2018;50(5):590-7.
1051. Zámbó K, Tóth K. Influence of blood viscosity on circulatory parameters determined by first-pass radionuclide angiocardiography in cor pulmonale. Nuklearmedizin. 1993;32(6):288-91.
1052. Fan W, Guo LX. Influence of different frequencies of axial cyclic loading on time-domain vibration response of the lumbar spine: A finite element study. Comput Biol Med. 2017;86:75-81.
1053. Sandor B, Csiszar B, Galos G, Funke S, Kevey DK, Meggyes M, et al. The Influence of Early Onset Preeclampsia on Perinatal Red Blood Cell Characteristics of Neonates. Int J Mol Sci. 2023;24(10).
1054. Velcheva I, Titianova E, Antonova N. Influence of hemorheological parameters and mean blood pressure on carotid blood flow asymmetry in patients with chronic unilateral cerebral infarctions. Clin Hemorheol Microcirc. 2010;45(2-4):239-44.
1055. Ciematnieks U, Gasjune R, Saulite S. INFLUENCE OF LOCAL VIBROSTIMULATION ON FOCUS PERSISTENCE. SOCIETY, INTEGRATION, EDUCATION, VOL IV: SPORTS AND HEALTH - ART AND DESIGN2019. p. 78-83.
1056. Amin A, El-Kareem MA, Yahia AB. Influence of low grade exercise on skeletal scintigraphy using Tc-99m methylene diphosphonate. Nucl Med Rev Cent East Eur. 2015;18(2):61-4.
1057. Rydzyk A, Blaszczyk J, Rydzyk R. The Influence of Low-Frequency Vibrations on the Lipid Profile in Women. ACTA BALNEOLOGICA. 2019;61(1):24-8.
1058. Zondervan HA, Oosting J, Hardeman MR, Smorenberg-Schoorl ME, Treffers PE. The influence of maternal whole blood viscosity on fetal growth. Eur J Obstet Gynecol Reprod Biol. 1987;25(3):187-94.
1059. Ireland A, Rittweger J, Degens H. The Influence of Muscular Action on Bone Strength Via Exercise. CLINICAL REVIEWS IN BONE AND MINERAL METABOLISM. 2014;12(2):93-102.
1060. Papp J, Toth A, Sandor B, Kiss R, Rabai M, Kenyeres P, et al. The influence of on-pump and off-pump coronary artery bypass grafting on hemorheological parameters. Clin Hemorheol Microcirc. 2011;49(1-4):331-46.
1061. Berschin G, Sommer HM. The influence of posture on transmission and absorption of vibration energy in whole body vibration exercise. Sportverletzung-Sportschaden. 2010;24(1):36-9.
1062. Paddan GS, Mansfield NJ, Arrowsmith CI, Rimell AN, King SK, Holmes SR. The influence of seat backrest angle on perceived discomfort during exposure to vertical whole-body vibration. Ergonomics. 2012;55(8):923-36.
1063. Majernik J, Zivcak J. Influence of single whole body vibration training unit on kinematics of human gait in children with neurological disorders. Communications - Scientific Letters of the University of Žilina. 2014;16(1):105-8.
1064. Da Silva ME, Fernandez JM, Castillo E, Nuñez VM, Vaamonde DM, Poblador MS, et al. Influence of vibration training on energy expenditure inactive men. JOURNAL OF STRENGTH AND CONDITIONING RESEARCH. 2007;21(2):470-5.
1065. Montoro MVP, Montilla JAP, Checa MA, Aguilera EDL. Influence of vibration training on pain and quality of life in women older than 65 years old. REVISTA DE PSICOLOGIA DEL DEPORTE. 2018;27(2):133-40.
1066. Robbins D, Yoganathan P, Goss-Sampson M. The influence of whole body vibration on the central and peripheral cardiovascular system. Clin Physiol Funct Imaging. 2014;34(5):364-9.
1067. Furness TP, Maschette WE. Influence of whole body vibration platform frequency on neuromuscular performance of community-dwelling older adults. Journal of strength and conditioning research. 2009;23(5):1508‐13.
1068. Ewertowska P, Zapadka BZ, Głażewska M, Poniatowski Ł, Tuptanowski K, Ossowski Z, et al. Influence of whole-body vibration and drop jump on the range of motion in the ankle joint and running parameters-A randomized crossover study. Physiother Res Int. 2024;29(4):e2132.
1069. Fachina R, da Silva A, Falcão W, Montagner P, Borin J, Minozzo F, et al. The influence of whole-body vibration on creatine kinase activity and jumping performance in young basketball players. Res Q Exerc Sport. 2013;84(4):503-11.
1070. Li X, Xie R, Peng X, Cheng L, Rochester CA. Influence of whole-body vibration training and stopping training on bone mineral density of old women's proximal femur. Basic & clinical pharmacology & toxicology. 2018;124:116‐.
1071. Shiuan-Yu T, Chung-Liang L, Kai-Ling C, Pi-Shan H, Meng-Chih L, Chun-Hou W, et al. Influence of Whole-Body Vibration Training Without Visual Feedback on Balance and Lower-Extremity Muscle Strength of the Elderly: A Randomized Controlled Trial. Medicine. 2016;95(5):1-6.
1072. Melcher C, Veronika W, Jansson V, Birkenmaier C, Rarak S, Wegener B. Influence on non-specific back pain and postural trunk control by whole-body vibration therapy. Global Spine Journal. 2017;7(2):110S.
1073. Zellner M. Inkontinenz nach radikaler prostatektomie und zystektomie: sind apparatives kombinationstraining und ganzkorpervibration effektiv? (Incontinence after radical prostatectomy and cystectomy: are combined training with mechanical devices and whole body vibration effective?) Der Urologe Ausg A 2011 Apr;50(4):433-444. 2011.
1074. de Souza HCM, Pessoa MF, Dos Santos Clemente R, da Silva AV, de Andrade EAM, Neves PAF, et al. Inspiratory muscle training in addition to whole body vibration for functional and physical outcomes in pre-frail older women: a randomized controlled trial. Age and ageing. 2022;51(4).
1075. Prusak K, Prusak K, Mahoney J. An integrated mind-body approach to arthritis: A pilot study. Journal of Traditional and Complementary Medicine. 2014;4(2):99-107.
1076. Luo Y, Wang J, Pei J, Rong Y, Liu W, Tang P, et al. Interactions between the MMP-3 gene rs591058 polymorphism and occupational risk factors contribute to the increased risk for lumbar disk herniation: A case-control study. J Clin Lab Anal. 2020;34(7):e23273.
1077. Zivadinov R, Treu CN, Weinstock-Guttman B, Turner C, Bergsland N, O'Connor K, et al. Interdependence and contributions of sun exposure and vitamin D to MRI measures in multiple sclerosis. J Neurol Neurosurg Psychiatry. 2013;84(10):1075-81.
1078. De Sousa RR, Ferreira LC, Ferreira AC, De Barros CPV, Gontijo APB. Interventions to improve balance in children and adolescents with down syndrome: A systematic review. Developmental Medicine and Child Neurology. 2019;61:45.
1079. Vela JI, Andreu D, Díaz-Cascajosa J, Buil JA. Intraocular lens dislocation after whole-body vibration. J Cataract Refract Surg. 2010;36(10):1790-1.
1080. Salzmann A, James SN, Williams DM, Richards M, Cadar D, Schott JM, et al. Investigating the Relationship Between IGF-I, IGF-II, and IGFBP-3 Concentrations and Later-Life Cognition and Brain Volume. J Clin Endocrinol Metab. 2021;106(6):1617-29.
1081. Nor JJ, Ummi NNA, Asyraf M, Norashiken O, Salleh AF. Investigation on young adult hand grip strength. Malaysian Journal of Public Health Medicine. 2020;20(Specialissue1):45-50.
1082. IOF Regionals, 2nd Middle East and Africa Osteoporosis Meeting, 6th Pan Arab Osteoporosis Congress PAOC'6. Osteoporosis International. 2012;23.
1083. Huh JY, Mougios V, Skraparlis A, Kabasakalis A, Mantzoros CS. Irisin in response to acute and chronic whole-body vibration exercise in humans. Metabolism. 2014;63(7):918-21.
1084. Huberman MA, d'Adesky ND, Niazi QB, Perez-Pinzon MA, Bramlett HM, Raval AP. Irisin-Associated Neuroprotective and Rehabilitative Strategies for Stroke. Neuromolecular Med. 2022;24(2):62-73.
1085. Mason RR, Cochrane DJ, Denny GJ, Firth EC, Stannard SR. Is 8 weeks of side-alternating whole-body vibration a safe and acceptable modality to improve functional performance in multiple sclerosis? DISABILITY AND REHABILITATION. 2012;34(8):647-54.
1086. Tseng SY, Ko CP, Tseng CY, Huang WC, Lai CL, Wang CH. Is 20 Hz Whole-Body Vibration Training Better for Older Individuals than 40 Hz? INTERNATIONAL JOURNAL OF ENVIRONMENTAL RESEARCH AND PUBLIC HEALTH. 2021;18(22).
1087. Okamoto T, Kobayashi R, Hashimoto Y, Kikuchi N, Ogoh S. Is individual day-to-day variation of arterial stiffness associated with variation of maximal aerobic performance? BMC SPORTS SCIENCE MEDICINE AND REHABILITATION. 2021;13(1).
1088. Chen YC, Chen WC, Liu CW, Huang WY, Lu IC, Lin CW, et al. Is moderate resistance training adequate for older adults with sarcopenia? A systematic review and network meta-analysis of RCTs. EUROPEAN REVIEW OF AGING AND PHYSICAL ACTIVITY. 2023;20(1).
1089. Iyigun G, Aksu Yildirim S, Snowdon N. Is physiotherapy effective in improving balance and gait in patients with multiple sclerosis? A systematic review. Turkiye Klinikleri Tip Bilimleri Dergisi [Turkish Journal of Medical Science] 2010;30(2):482-493. 2010.
1090. Francio V, Raum G, Barndt B, Cohen E, Eubanks JE, Batri A. Is there a role for whole body vibration (WBV) therapy in neuromusculo-skeletal (NMS) rehabilitation? PM and R. 2019;11:S8.
1091. Marin-Puyalto J, Gomez-Cabello A, Gonzalez-Aguero A, Gomez-Bruton A, Matute-Llorente A, Casajus JA, et al. Is vibration training good for your bones? An overview of systematic reviews. BioMed Research International 2018;(5178284):Epub. 2018.
1092. Melo Marinho PÉ, Rocha LG, Araújo Filho JC, Sañudo B, Seixas A, Andrade AD. Is whole body vibration an alternative physical training method for renal transplant recipients? Physiotherapy Research International. 2020;25(3):1-8.
1093. Ching KE. Is whole body vibration treatment more effective than traditional physical therapy treatment for increasing lower extremity muscle strength in a female toddler with developmental delays? Is Whole Body Vibration Treatment More Effective Than Traditional Physical Therapy Treatment for Increasing Lower Extremity Muscle Strength in a Female Toddler with Developmental Delays? 2016:1-.
1094. Lachance C, Weir P, Kenno K, Horton S. Is whole-body vibration beneficial for seniors? EUROPEAN REVIEW OF AGING AND PHYSICAL ACTIVITY. 2012;9(1):51-62.
1095. Dabbs NC, Svoboda SM. Is Whole-Body Vibration Training Effective? STRENGTH AND CONDITIONING JOURNAL. 2016;38(4):72-4.
1096. Myers BJ. Isokinetic testing of muscle strength in older adults with knee osteoarthritis: An integrative review. ISOKINETICS AND EXERCISE SCIENCE. 2020;28(3):269-90.
1097. Merrigan JJ, Dabbs NC, Jones MT. Isometric Mid-thigh Pull Kinetics: Sex Differences and Response to Whole-Body Vibration. Journal of strength and conditioning research. 2020;34(9):2407-11.
1098. Baldinotti I, Timmann D, Kolb FP, Kutz DF. Jerk analysis of active body-weight-transfer. Gait Posture. 2010;32(4):667-72.
1099. Bellver M, Drobnic F, Jovell E, Ferrer-Roca V, Abalos X, Del Rio L, et al. Jumping rope and whole-body vibration program effects on bone values in Olympic artistic swimmers. Journal of Bone & Mineral Metabolism. 2021;39(5):858-67.
1100. Liao LR, Lam FM, Pang MY, Jones AY, Ng GY. Leg muscle activity during whole-body vibration in individuals with chronic stroke. Med Sci Sports Exerc. 2014;46(3):537-45.
1101. Schumann B, Bolm-Audorff U, Bergmann A, Ellegast R, Elsner G, Grifka J, et al. Lifestyle factors and lumbar disc disease: Results of a German multi-center case-control study (EPILIFT). Arthritis Research and Therapy. 2010;12(5).
1102. Luginbuehl Greco H, Lehmann C, Gerber R, Lauper M, Kuhn A, Hilfiker R, et al. Load duration during stochastic whole body vibration in its effect on pelvic floor muscle activation. Physiotherapy (United Kingdom). 2011;97:eS715-eS6.
1103. Bergmann G, Kutzner I, Bender A, Dymke J, Trepczynski A, Duda GN, et al. Loading of the hip and knee joints during whole body vibration training. PLoS ONE. 2018;13(12).
1104. Friesenbichler B, Nigg BM, Dunn JF. Local metabolic rate during whole body vibration. JOURNAL OF APPLIED PHYSIOLOGY. 2013;114(10):1421-5.
1105. Verbeke FH, Agharazii M, Boutouyrie P, Pannier B, Guérin AP, London GM. Local shear stress and brachial artery functions in end-stage renal disease. J Am Soc Nephrol. 2007;18(2):621-8.
1106. Percival S, Sims DT, Stebbings GK. Local Vibration Therapy, Oxygen Resaturation Rate, and Muscle Strength After Exercise-Induced Muscle Damage. JOURNAL OF ATHLETIC TRAINING. 2022;57(5):502-9.
1107. Tomiak T, Niewiadomska MA, Zasada M, Melnik LJ, Mishchenko VS, Sawczyn S. The Long Lasting Effects of Whole Body Rotation Vibration in a Fitness Exercise Program on Flexibility and Motor Coordination in Young Females. BALTIC JOURNAL OF HEALTH AND PHYSICAL ACTIVITY. 2012;4(4):221-30.
1108. Jakimovski D, Bergsland N, Dwyer MG, Hagemeier J, Ramasamy DP, Szigeti K, et al. Long-standing multiple sclerosis neurodegeneration: volumetric magnetic resonance imaging comparison to Parkinson's disease, mild cognitive impairment, Alzheimer's disease, and elderly healthy controls. Neurobiol Aging. 2020;90:84-92.
1109. Manimmanakorn N, Hamlin MJ, Ross JJ, Manimmanakorn A. LONG-TERM EFFECT OF WHOLE BODY VIBRATION TRAINING ON JUMP HEIGHT: META-ANALYSIS. JOURNAL OF STRENGTH AND CONDITIONING RESEARCH. 2014;28(6):1739-50.
1110. van Nes IJ, Latour H, Schils F, Meijer R, van Kuijk A, Geurts AC. Long-term effects of 6-week whole-body vibration on balance recovery and activities of daily living in the postacute phase of stroke: a randomized, controlled trial. Stroke 2006 Sep;37(9):2331-2335. 2006.
1111. Muniz-Pardos B, Gómez-Bruton A, Matute-Llorente Á, González-Agüero A, Gómez-Cabello A, Casajús JA, et al. Long-Term Effects of Whole-Body Vibration in Trained Adolescent Swimmers: Does It Increase Strength, Power, and Swimming Performance? International Journal of Sports Physiology & Performance. 2020;15(3):416-22.
1112. Irct20230304057612N. Long-Term Effects of Whole-Body Vibration on Sarcopenia in Geriatric Population. https://trialsearchwhoint/Trial2aspx?TrialID=IRCT20230304057612N1. 2023.
1113. Fernandez-Rio J, Terrados N, Suman O. Long-term effects of whole-body vibration training in high-level female basketball players. Journal of Sports Medicine & Physical Fitness. 2012;52(1):18-26.
1114. Seidel H, Heide R. Long-term effects of whole-body vibration: a critical survey of the literature. Int Arch Occup Environ Health. 1986;58(1):1-26.
1115. Kennis E, Verschueren SM, Bogaerts A, Van Roie E, Boonen S, Delecluse C. Long-Term Impact of Strength Training on Muscle Strength Characteristics in Older Adults. Archives of Physical Medicine & Rehabilitation. 2013;94(11):2054-60.
1116. Schimanko EK, Rossato J, Hidalgo KA, Baioco RA, de Carvalho AR, Bertolini GRF. Long-Term Whole-Body Vibration on Knee Extensor Function: A Randomized Clinical Trial. Muscles, Ligaments & Tendons Journal (MLTJ). 2023;13(3):376-82.
1117. Montagnese F, Thiele S, Wenninger S, Schoser B. Long-term whole-body vibration training in two late-onset Pompe disease patients. Neurological Sciences. 2016;37(8):1357-60.
1118. Murtezani A, Hundozi H, Orovcanec N, Berisha M, Meka V. Low back pain predict sickness absence among power plant workers. Indian Journal of Occupational & Environmental Medicine. 2010;14(2):49-53.
1119. Kelsey JL, Golden AL, Mundt DJ. Low back pain/prolapsed lumbar intervertebral disc. Rheumatic Disease Clinics of North America. 1990;16(3):699-716.
1120. Crow JF, Buttifant D, Kearny SG, Hrysomallis C. Low load exercises targeting the gluteal muscle group acutely enhance explosive power output in elite athletes. Journal of Strength & Conditioning Research 2012 Feb;26(2):438-442. 2012.
1121. Li H, Li L. Low magnitude whole-body vibration and postmenopausal osteoporosis. Sheng wu yi xue gong cheng xue za zhi = Journal of biomedical engineering = Shengwu yixue gongchengxue zazhi. 2018;35(2):301-6.
1122. Tihanyi J, Di Giminiani R, Tihanyi T, Gyulai G, Trzaskoma L, Horváth M. Low resonance frequency vibration affects strength of paretic and non-paretic leg differently in patients with stroke. Acta physiologica Hungarica. 2010;97(2):172‐82.
1123. Ibrahim ZM, Ali OI, Moawd SA, Eid MM, Taha MM. Low vibrational training as an additional intervention for postural balance, balance confidence and functional mobility in type 2 diabetic patients with lower limb burn injury: a randomized clinical trial. Diabetes, Metabolic Syndrome and Obesity 2021 Aug 11;14:3617-3626. 2021.
1124. Castillo AB, Alam I, Tanaka SM, Levenda J, Li J, Warden SJ, et al. Low-amplitude, broad-frequency vibration effects on cortical bone formation in mice. Bone. 2006;39(5):1087-96.
1125. Liu KC, Wang JS, Hsu CY, Liu CH, Chen CP, Huang SC. Low-Frequency Vibration Facilitates Post-Exercise Cardiovascular Autonomic Recovery. JOURNAL OF SPORTS SCIENCE AND MEDICINE. 2021;20(3):431-7.
1126. Gusi N, Raimundo A, Leal A. Low-frequency vibratory exercise reduces the risk of bone fracture more than walking: a randomized controlled trial. BMC Musculoskeletal Disorders 2006 Nov 30;7(92):Epub. 2006.
1127. Chen YF, Sun SX, Zhou XY, He M, Li Y, Liu CC, et al. Low-intensity pulsed ultrasound and parathyroid hormone improve muscle atrophy in estrogen deficiency mice. ULTRASONICS. 2023;132.
1128. Calder CG, Mannion J, Metcalf PA. Low-intensity whole-body vibration training to reduce fall risk in active, elderly residents of a retirement village. Journal of the American Geriatrics Society 2013 Aug;61(8):1424-1426. 2013.
1129. Oxlund BS, Ørtoft G, Andreassen TT, Oxlund H. Low-intensity, high-frequency vibration appears to prevent the decrease in strength of the femur and tibia associated with ovariectomy of adult rats. Bone. 2003;32(1):69-77.
1130. Rubin C, Judex S, Qin YX. Low-level mechanical signals and their potential as a non-pharmacological intervention for osteoporosis. AGE AND AGEING. 2006;35:32-6.
1131. Xie LQ, Jacobson JM, Choi ES, Busa B, Donahue LR, Miller LM, et al. Low-level mechanical vibrations can influence bone resorption and bone formation in the growing skeleton. BONE. 2006;39(5):1059-66.
1132. Gilsanz V, Wren TAL, Sanchez M, Dorey F, Judex S, Rubin C. Low-level, high-frequency mechanical signals enhance musculoskeletal development of young women with low BMD. Journal of Bone and Mineral Research. 2006;21(9):1464-74.
1133. Chen B, Li Y, Xie D, Yang X. Low-magnitude high-frequency loading via whole body vibration enhances bone-implant osseointegration in ovariectomized rats. Journal of Orthopaedic Research. 2012;30(5):733-9.
1134. Leung KS, Shi HF, Cheung WH, Qin L, Ng WK, Tam KF, et al. Low-Magnitude High-Frequency Vibration Accelerates Callus Formation, Mineralization, and Fracture Healing in Rats. JOURNAL OF ORTHOPAEDIC RESEARCH. 2009;27(4):458-65.
1135. Judex S, Lei X, Han D, Rubin C. Low-magnitude mechanical signals that stimulate bone formation in the ovariectomized rat are dependent on the applied frequency but not on the strain magnitude. JOURNAL OF BIOMECHANICS. 2007;40(6):1333-9.
1136. Kiel DP, Hannan MT, Barton BA, Bouxsein ML, Sisson E, Lang T, et al. Low-Magnitude Mechanical Stimulation to Improve Bone Density in Persons of Advanced Age: A Randomized, Placebo-Controlled Trial. Journal of Bone and Mineral Research. 2015;30(7):1319-28.
1137. Yu X, Zeng Y, Bao M, Wen J, Zhu G, Cao C, et al. Low-magnitude vibration induces osteogenic differentiation of bone marrow mesenchymal stem cells via miR-378a-3p/Grb2 pathway to promote bone formation in a rat model of age-related bone loss. FASEB Journal. 2020;34(9):11754-71.
1138. van der Jagt OP, van der Linden JC, Waarsing JH, Verhaar JAN, Weinans H. Low-magnitude whole body vibration does not affect bone mass but does affect weight in ovariectomized rats. JOURNAL OF BONE AND MINERAL METABOLISM. 2012;30(1):40-6.
1139. Spielmanns M, Boeselt T, Gloeckl R, Klutsch A, Fischer H, Polanski H, et al. Low-volume whole-body vibration training improves exercise capacity in subjects with mild to severe COPD. Respiratory Care 2017 Mar;62(3):315-323. 2017.
1140. Colson SS, Petit PD. Lower Limbs Power and Stiff ness after Whole-Body Vibration. International Journal of Sports Medicine. 2013;34(4):318-23.
1141. Kang DK, Kim TH, Han TS, Kim KS, Yim H. Magnetic resonance imaging enhancement features before and after neoadjuvant chemotherapy in patients with breast cancer: a predictive value for responders. J Comput Assist Tomogr. 2013;37(3):432-9.
1142. Rizzoli R, Bruyere O, Cannata-Andia JB, Devogelaer JP, Lyritis G, Ringe JD, et al. Management of osteoporosis in the elderly. CURRENT MEDICAL RESEARCH AND OPINION. 2009;25(10):2373-87.
1143. Negm AM, Lee J, Hamidian R, Jones CA, Khadaroo RG. Management of Sarcopenia: A Network Meta-Analysis of Randomized Controlled Trials. Journal of the American Medical Directors Association. 2022;23(5):707-14.
1144. Senderovich H, Bayeva N, Montagnese B, Yendamuri A. Managing Fall Prevention through Exercise in Older Adults Afflicted by Cognitive and Strength Impairment. Dementia & Geriatric Cognitive Disorders. 2021;50(6):507-18.
1145. Kaplangoray M, Toprak K, Aydın C, Cekici Y, Yıldırım A, Ozcan Abacıoglu O. The MAPH Score Predicts Coronary Slow Flow. A Retrospective Case-Controlled Study. Kardiologiia. 2024;64(2):67-72.
1146. Dong D, Sun ML, Xu D, Han S, Cui LY, Cao S, et al. Mapping the Hot Spots and Evolution Main Path of Whole-Body Vibration Training Since the 21st Century: A Bibliometric Analysis. FRONTIERS IN BIOENGINEERING AND BIOTECHNOLOGY. 2022;10.
1147. Nurudeen S, Shin D, Herring D, Heinrichs K. The Measure of Success: Quantifying the Acute Effects of Whole Body Vibration on Hamstring Flexibility. Advances in Human Factors and Ergonomics in Healthcare2010. p. 855-64.
1148. Magnusson M, Almqvist M, Broman H, Pope M, Hansson T. Measurement of height loss during whole body vibrations. J Spinal Disord. 1992;5(2):198-203.
1149. Pedersen ME, Wilmerding MV, Milani J, Mancha J. Measures of plantar flexion and dorsiflexion strength in flamenco dancers. Medical Problems of Performing Artists. 1999;14(3):107-12.
1150. Goutsis DK, Hall TL, Anderson AA, Crawford MA, Hoover DL. Measures of power but not fatigue are influenced by whole body vibration prior to vigorous cycling. Physiotherapy (United Kingdom). 2015;101:eS474-eS5.
1151. Camargos GV, Bhattacharya P, van Lenthe GH, Del Bel Cury AA, Naert I, Duyck J, et al. Mechanical competence of ovariectomy-induced compromised bone after single or combined treatment with high-frequency loading and bisphosphonates. Scientific reports. 2015;5:10795.
1152. Shojaei I, Salt EG, Hooker Q, Bazrgari B. Mechanical demands on the lower back in patients with non-chronic low back pain during a symmetric lowering and lifting task. Journal of Biomechanics. 2018;70:255-61.
1153. Holmlund P, Lundström R. Mechanical impedance of the sitting human body in single-axis compared to multi-axis whole-body vibration exposure. Clin Biomech (Bristol). 2001;16 Suppl 1:S101-10.
1154. Kasturi G, Adler RA. Mechanical means to improve bone strength: ultrasound and vibration. Curr Rheumatol Rep. 2011;13(3):251-6.
1155. Chow SK, Leung KS, Qin J, Guo A, Sun M, Qin L, et al. Mechanical stimulation enhanced estrogen receptor expression and callus formation in diaphyseal long bone fracture healing in ovariectomy-induced osteoporotic rats. Osteoporos Int. 2016;27(10):2989-3000.
1156. Cheung AM, Giangregorio L. Mechanical stimuli and bone health: What is the evidence? Current Opinion in Rheumatology. 2012;24(5):561-6.
1157. Salvarani A, Agosti M, Zanrè A, Ampollini A, Montagna L, Franceschini M. Mechanical vibration in the rehabilitation of patients with reconstructed anterior cruciate ligament. Europa Medicophysica. 2003;39(1):19-25.
1158. Jin FY, Ruan XY. Mechanical vibration in the treatment of postmenopausal women with knee osteoarthritis. Journal of Clinical Rehabilitative Tissue Engineering Research. 2007;11(40):8099-102.
1159. Debenedictis T, Milanese S, Billing DC, Furnell A, Tomkinson G, Fraysse F, et al. Mechanical whole-body vibration during military vehicle transit and lower limb explosive power. Journal of science and medicine in sport. 2017;20:S167‐.
1160. Yokoi H, Take Y, Mae T, Shimomura K, Hanai T, Okamoto T, et al. The mechanism responsible for the promotive effects of vibration acceleration on fracture healing in a rat model. Journal of Orthopaedic Research. 2017;35.
1161. d'Errico A, Gore R, Gold JE, Park JS, Punnett L. Medium- and long-term reproducibility of self-reported exposure to physical ergonomics factors at work. Appl Ergon. 2007;38(2):167-75.
1162. Bhak Y, Tenesa A. Mendelian randomization study of whole blood viscosity and cardiovascular diseases. PLoS One. 2024;19(4):e0294095.
1163. Kang J, Porfido T, Ismaili C, Selamie S, Kuper J, Bush JA, et al. Metabolic responses to whole-body vibration: effect of frequency and amplitude. EUROPEAN JOURNAL OF APPLIED PHYSIOLOGY. 2016;116(9):1829-39.
1164. Beijer Å, Degens H, Weber T, Rosenberger A, Gehlert S, Herrera F, et al. Microcirculation of skeletal muscle adapts differently to a resistive exercise intervention with and without superimposed whole-body vibrations. Clin Physiol Funct Imaging. 2015;35(6):425-35.
1165. Bidonde J, Busch AJ, Schachter CL, Webber SC, Musselman KE, Overend TJ, et al. Mixed exercise training for adults with fibromyalgia. Cochrane Database Syst Rev. 2019;5(5):Cd013340.
1166. Goudarzian M, Rahimi M, Karimi N, Samadi A, Ajudani R, Sahaf R, et al. Mobility, Balance, and Muscle Strength Adaptations to Short-Term Whole Body Vibration Training Plus Oral Creatine Supplementation in Elderly Women. Asian Journal of Sports Medicine. 2017;8(1):1-9.
1167. Yue Z, Mester J. A model analysis of internal loads, energetics, and effects of wobbling mass during the whole-body vibration. Journal of Biomechanics. 2002;35(5):639-47.
1168. Mansfield NJ, Lundström R. Models of the apparent mass of the seated human body exposed to horizontal whole-body vibration. Aviat Space Environ Med. 1999;70(12):1166-72.
1169. Boughdadi FA, Said HA, Salama-Younes M, Qatwan NHS. Modern therapeutic methods and high technology (power plate and resistance exercise) for osteoporosis hindered. Journal of Men's Health. 2010;7(3):286.
1170. Swiggum M, Grant L. Monitoring Procedural Pain and Distress in a Child With Rett Syndrome: A Case Report. Pediatr Phys Ther. 2019;31(4):E1-e5.
1171. Nobili L, Schiavi G, Bozano E, De Carli F, Ferrillo F, Nobili F. Morning increase of whole blood viscosity in obstructive sleep apnea syndrome. Clin Hemorheol Microcirc. 2000;22(1):21-7.
1172. Kakihata CMM, Peretti AL, Tavares ALF, Wutzke MLS, Ribeiro LFC, Costa RM, et al. Morphometric Effects of Whole-Body Vibration on the Bone in a Rat Model of Postmenopausal Osteoporosis. J Manipulative Physiol Ther. 2020;43(5):551-7.
1173. Tang R, Gungor C, Sesek RF, Foreman KB, Gallagher S, Davis GA. Morphometry of the lower lumbar intervertebral discs and endplates: comparative analyses of new MRI data with previous findings. EUROPEAN SPINE JOURNAL. 2016;25(12):4116-31.
1174. Otzel DM, Hass CJ, Wikstrom EA, Bishop MD, Borsa PA, Tillman MD. Motoneuron Function Does not Change Following Whole-Body Vibration in Individuals With Chronic Ankle Instability. Journal of Sport Rehabilitation. 2019;28(6):614-22.
1175. Pope MH, Svensson M, Broman H, Andersson GB. Mounting of the transducers in measurement of segmental motion of the spine. J Biomech. 1986;19(8):675-7.
1176. Lombardi J, Anderl-Straub S, Semler E, Uttner I, Kassubek J, Diehl-Schmid J, et al. MRI AS OBJECTIVE FOLLOW-UP PARAMETER IN PATIENTS OF PRIMARY PROGRESSIVE APHASIAS. Alzheimer's and Dementia. 2019;15(7):P1402-P3.
1177. Duan SZ, Asme. Multibody Dynamics Approaches for Study on Good and Bad Whole-Body Vibrations. PROCEEDINGS OF THE ASME INTERNATIONAL MECHANICAL ENGINEERING CONGRESS AND EXPOSITION, 2018, VOL 4B2019.
1178. Duan S, Mattison L, Binkley T, Asme. Multibody Dynamics Model for Analysis of Human Body Response to Vibrations. INTERNATIONAL MECHANICAL ENGINEERING CONGRESS AND EXPOSITION - 2012, VOL 4, PTS A AND B2013. p. 1083-8.
1179. Seidel E, Rother M, Fischer A, Regenspurger K, Rother I, Kneer W, et al. Multicentered, Randomized, Cross-Over Study to Investigate Safety and Potency of Whole-Body-Vibrations as Add-on Therapy to Pharmacological Treatment of Postmenopausal Women with Osteoporosis. PHYSIKALISCHE MEDIZIN REHABILITATIONSMEDIZIN KURORTMEDIZIN. 2014;24(1):9-13.
1180. Silva DdS, Braz RRS, Silva DdA, de Oliveira KCV, Campos SL, de Araújo MdGR. MULTICOMPONENT TRAINING ASSOCIATED WITH WHOLE BODY VIBRATION: EFFECT ON FUNCTIONAL CAPACITY AND QUALITY OF LIFE IN ELDERLY WOMEN WITH OSTEOPOROSIS...1st Student Scientific Conference of the Brazilian Association for Research and Postgraduate in Physiotherapy (ABRAPG-FT), May 19-21, 2023 (Online). Brazilian Journal of Physical Therapy. 2024;28:N.PAG-N.PAG.
1181. Ezenwa B, Burns E, Wilson C, Ieee. Multiple Vibration Intensities and Frequencies For Bone Mineral Density Improvement. 2008 30TH ANNUAL INTERNATIONAL CONFERENCE OF THE IEEE ENGINEERING IN MEDICINE AND BIOLOGY SOCIETY, VOLS 1-82008. p. 4186-+.
1182. Yung M, Tennant LM, Milosavljevic S, Trask C. The Multisystem Effects of Simulated Agricultural Whole Body Vibration on Acute Sensorimotor, Physical, and Cognitive Performance. Ann Work Expo Health. 2018;62(7):884-98.
1183. Huang M, Pang MYC. Muscle activity and vibration transmissibility during whole-body vibration in chronic stroke. Scand J Med Sci Sports. 2019;29(6):816-25.
1184. Masani K, Alizadeh-Meghrazi M, Sayenko DG, Zariffa J, Moore C, Giangregorio L, et al. Muscle activity, cross-sectional area, and density following passive standing and whole body vibration: A case series. JOURNAL OF SPINAL CORD MEDICINE. 2014;37(5):575-81.
1185. Cronin JB, Oliver M, McNair PJ. Muscle stiffness and injury effects of whole body vibration. Physical Therapy in Sport. 2004;5(2):68-74.
1186. Bellomo RG, Iodice P, Maffulli N, Maghradze T, Coco V, Saggini R. MUSCLE STRENGTH AND BALANCE TRAINING IN SARCOPENIC ELDERLY: A PILOT STUDY WITH RANDOMIZED CONTROLLED TRIAL. EUROPEAN JOURNAL OF INFLAMMATION. 2013;11(1):193-201.
1187. Wollersheim T, Grunow JJ, Carbon NM, Haas K, Malleike J, Ramme SF, et al. Muscle wasting and function after muscle activation and early protocol-based physiotherapy: an explorative trial. Journal of Cachexia, Sarcopenia and Muscle. 2019;10(4):734-47.
1188. Wilder DG, Aleksiev AR, Magnusson ML, Pope MH, Spratt KF, Goel VK. Muscular response to sudden load. A tool to evaluate fatigue and rehabilitation. Spine (Phila Pa 1976). 1996;21(22):2628-39.
1189. Walker-Bone K, Palmer KT. Musculoskeletal disorders in farmers and farm workers. Occup Med (Lond). 2002;52(8):441-50.
1190. Stuermer EK, Komrakova M, Werner C, Wicke M, Kolios L, Sehmisch S, et al. Musculoskeletal Response to Whole-Body Vibration During Fracture Healing in Intact and Ovariectomized Rats. CALCIFIED TISSUE INTERNATIONAL. 2010;87(2):168-80.
1191. Burström L, Aminoff A, Björ B, Mänttäri S, Nilsson T, Pettersson H, et al. Musculoskeletal symptoms and exposure to whole-body vibration among open-pit mine workers in the Arctic. Int J Occup Med Environ Health. 2017;30(4):553-64.
1192. Barbero M, Corti G, Fiorentini R, Tettamanti A, Gatti R. Myoelectric and perceived fatigue during a semi-squat performed on whole body vibration plate. Physiotherapy (United Kingdom). 2011;97:eS105.
1193. de Winkel KN, Nesti A, Ayaz H, Bülthoff HH. Neural correlates of decision making on whole body yaw rotation: An fNIRS study. Neuroscience Letters. 2017;654:56-62.
1194. Amonette WE, Boyle M, Psarakis MB, Barker J, Dupler TL, Ott SD. Neurocognitive responses to a single session of static squats with whole body vibration. Journal of Strength and Conditioning Research. 2015;29(1):96-100.
1195. Filippi P, Vestenická V, Siarnik P, Sivakova M, Čopíková-Cudráková D, Belan V, et al. Neurofilament light chain and MRI volume parameters as markers of neurodegeneration in multiple sclerosis. Neuro Endocrinol Lett. 2020;41(1):17-26.
1196. Paul RH, Gunstad J, Poppas A, Tate DF, Foreman D, Brickman AM, et al. Neuroimaging and cardiac correlates of cognitive function among patients with cardiac disease. Cerebrovasc Dis. 2005;20(2):129-33.
1197. Sweet LH, Paul RH, Cohen RA, Moser D, Ott BR, Gordon N, et al. Neuroimaging correlates of dementia rating scale performance at baseline and 12-month follow-up among patients with vascular dementia. J Geriatr Psychiatry Neurol. 2003;16(4):240-4.
1198. Merkert J, Butz S, Nieczaj R, Steinhagen-Thiessen E, Eckardt R. [Neurological lower torso function test. A new assessment]. Z Gerontol Geriatr. 2013;46(2):151-9.
1199. Melnyk M, Schloz C, Schmitt S, Gollhofer A. Neuromuscular ankle joint stabilisation after 4-weeks WBV training. Int J Sports Med. 2009;30(6):461-6.
1200. Maffiuletti NA, Saugy J, Cardinale M, Micallef JP, Place N. Neuromuscular fatigue induced by whole-body vibration exercise. Eur J Appl Physiol. 2013;113(6):1625-34.
1201. Feria-Madueño A, Hewett TE, Sánchez-Arteaga A, Sañudo B. Neuromuscular Response during Different Side-Cutting Maneuvers and Its Influence on the Risk of Knee Injuries. SPORTS. 2023;11(10).
1202. Liang V, Henderson G, Wu J. Neuromuscular response to a single session of whole-body vibration in children with cerebral palsy: A pilot study. Clin Biomech (Bristol). 2020;80:105170.
1203. Chang CM, Tsai CH, Lu MK, Tseng HC, Lu G, Liu BL, et al. The neuromuscular responses in patients with Parkinson's disease under different conditions during whole-body vibration training. BMC Complement Med Ther. 2022;22(1):2.
1204. Harwood B, Scherer J, Brown RE, Cornett KMD, Kenno KA, Jakobi JM. Neuromuscular responses of the plantar flexors to whole-body vibration. Scand J Med Sci Sports. 2017;27(12):1569-75.
1205. Stark C, Hoyer-Kuhn HK, Semler O, Hoebing L, Duran I, Cremer R, et al. Neuromuscular training based on whole body vibration in children with spina bifida: a retrospective analysis of a new physiotherapy treatment program. Childs Nerv Syst. 2015;31(2):301-9.
1206. Yin J, Lu X, Qian Z, Xu W, Zhou X. New insights into the pathogenesis and treatment of sarcopenia in chronic heart failure. Theranostics. 2019;9(14):4019-29.
1207. Winkelmann A, Schilling S, Neuerburg C, Mutschler W, Böcker W, Felsenberg D, et al. New strategies for exercise training in osteoporosis. Unfallchirurg. 2015;118(11):933-7.
1208. Park W, Park HY. New Trend of Physical Activity and Exercise for Health Promotion and Functional Ability. INTERNATIONAL JOURNAL OF ENVIRONMENTAL RESEARCH AND PUBLIC HEALTH. 2022;19(13).
1209. Oliveira MP, Cochrane D, Drummond MDM, Albuquerque MR, Almeida PAS, Couto BP. No acute effect of whole-body vibration on roundhouse kick and countermovement jump performance of competitive taekwondo athletes. Revista Brasileira de Cineantropometria e Desempenho Humano. 2018;20(6):576-84.
1210. Segal NA, Glass NA, Wallace R. No detectable effect of vibration exercise on thigh strength or power: A randomized controlled 12-week study of women at risk for knee osteoarthritis. PM and R. 2011;3(10):S217.
1211. Brogårdh C, Flansbjer U, Lexell J. No Effects of Whole-Body Vibration Training on Muscle Strength and Gait Performance in Persons With Late Effects of Polio: A Pilot Study. Archives of Physical Medicine & Rehabilitation. 2010;91(9):1474-7.
1212. Brogårdh C, Flansbjer U-B, Lexell J. No Specific Effect of Whole-Body Vibration Training in Chronic Stroke: A Double-Blind Randomized Controlled Study. Archives of Physical Medicine & Rehabilitation. 2012;93(2):253-8.
1213. Dobler CC, Morrow AS, Farah MH, Beuschel B, Majzoub AM, Wilson ME, et al. Nonpharmacologic therapies in patients with exacerbation of chronic obstructive pulmonary disease: a systematic review with meta-analysis. Mayo Clinic Proceedings 2020 Jun;95(6):1169-1183. 2020.
1214. Rosado H, Bravo J, Raimundo A, Carvalho J, Leite N, Pereira C. Novel approaches to reduce the risk of falling in community dwellings: effects of two multimodal programs in lower-body strength—a pilot study...Coimbra Health School Annual Meeting, June 17-19, 2021. European Journal of Public Health. 2021;31:1-.
1215. Jiang Z, Zhao B, Shang HF, Song W. A novel nonsense mutation in the TYMP gene causing MNGIE with multiple intracranial hemorrhages on brain MRI. Neurological Sciences. 2021;42(5):2119-22.
1216. Pujari AN, Neilson RD, Cardinale M, Ieee. A Novel Vibration Device for Neuromuscular Stimulation for Sports and Rehabilitation Applications. 2009 ANNUAL INTERNATIONAL CONFERENCE OF THE IEEE ENGINEERING IN MEDICINE AND BIOLOGY SOCIETY, VOLS 1-202009. p. 839-+.
1217. Beck BR, Kent K, Holloway L, Marcus R. Novel, high-frequency, low-strain mechanical loading for premenopausal women with low bone mass: Early findings. Journal of Bone and Mineral Metabolism. 2006;24(6):505-7.
1218. Sadowski T, Bielfeldt S, Wilhelm KP, Sukopp S, Gordon C. Objective and subjective reduction of cellulite volume using a localized vibrational massage device in a 24-week randomized intra-individual single-blind regression study. Int J Cosmet Sci. 2020;42(3):277-88.
1219. Yanik EL, Keener JD, Stevens MJ, Walker-Bone KE, Dale AM, Ma Y, et al. Occupational demands associated with rotator cuff disease surgery in the UK Biobank. Scandinavian Journal of Work, Environment and Health. 2023;49(1):53-63.
1220. Yanik E, Evanoff B, Walker-Bone K, Dale AM, Ma Y, Keener J, et al. Occupational demands associated with rotator cuff disease surgery: Results from a novel linkage of a job-exposure matrix to the UK biobank. Occupational and Environmental Medicine. 2021;78(SUPPL 1):A32-A3.
1221. Mosconi G, Borleri D, Mandelli G, Prandi E, Belotti L. [Occupational disease in construction work]. Med Lav. 2003;94(3):296-311.
1222. Harazin B. [Occupational exposure of whole-body vibration in Poland]. Med Pr. 2002;53(6):465-72.
1223. Zeng D, Ling XY, Fang ZL, Lu YF. Optimal exercise to improve physical ability and performance in older adults with sarcopenia: a systematic review and network meta-analysis. GERIATRIC NURSING. 2023;52:199-207.
1224. Kim GW, Yoon JY, Kang SR, Lee EA, Won YH, Park SH, et al. Optimal frequency of sonic whole body vibration on muscle function and muscle fatigue. Osteoporosis International. 2020;31(SUPPL 1):S319.
1225. Wei N, Cai M. Optimal frequency of whole body vibration training for improving balance and physical performance in the older people with chronic stroke: a randomized controlled trial. Clinical Rehabilitation 2022 Mar;36(3):342-349. 2022.
1226. Adams JB, Edwards D, Serviette D, Bedient AM, Huntsman E, Jacobs KA, et al. OPTIMAL FREQUENCY, DISPLACEMENT, DURATION, AND RECOVERY PATTERNS TO MAXIMIZE POWER OUTPUT FOLLOWING ACUTE WHOLE-BODY VIBRATION. JOURNAL OF STRENGTH AND CONDITIONING RESEARCH. 2009;23(1):237-45.
1227. Wei N, Pang MYC, Ng SSM, Ng GYF. Optimal frequency/time combination of whole body vibration training for developing physical performance of people with sarcopenia: a randomized controlled trial. CLINICAL REHABILITATION. 2017;31(10):1313-21.
1228. Wei N, Pang MYC, Ng SSM, Ng GYF. Optimal frequency/time combination of whole-body vibration training for improving muscle size and strength of people with age-related muscle loss (sarcopenia): A randomized controlled trial. Geriatrics and Gerontology International. 2017;17(10):1412-20.
1229. Bowers AS, Pepple DJ, Reid HL. Optimal haematocrit in subjects with normal haemoglobin genotype (HbAA), sickle cell trait (HbAS), and homozygous sickle cell disease (HbSS). Clin Hemorheol Microcirc. 2011;47(4):253-60.
1230. Drks. Optimizing currant exercise strategies of public compulsory health insurances for musculoskeletal prevention. A randomized controlled pilot study with postmenopausal women. https://trialsearchwhoint/Trial2aspx?TrialID=DRKS00009331. 2015.
1231. Gerhardt F, Dumitrescu D, Gärtner C, Beccard R, Viethen T, Kramer T, et al. Oscillatory whole-body vibration improves exercise capacity and physical performance in pulmonary arterial hypertension: a randomised clinical study. Heart (British Cardiac Society). 2017;103(8):592‐8.
1232. Roos EM, Juhl C. Osteoarthritis year in review: Rehabilitation and outcomes. Osteoarthritis and Cartilage. 2012;20:S8.
1233. Schatz M, Saravanan S, d'Adesky ND, Bramlett H, Perez-Pinzon MA, Raval AP. Osteocalcin, ovarian senescence, and brain health. Front Neuroendocrinol. 2020;59:100861.
1234. Nct. Osteogenesis Imperfecta and Balance. https://clinicaltrialsgov/ct2/show/NCT06010134. 2023.
1235. Boonen S, Dejaeger E, Vanderschueren D, Venken K, Bogaerts A, Verschueren S, et al. Osteoporosis and osteoporotic fracture occurrence and prevention in the elderly: a geriatric perspective. BEST PRACTICE & RESEARCH CLINICAL ENDOCRINOLOGY & METABOLISM. 2008;22(5):765-85.
1236. Hita-Contreras F, Martínez-Amat A, Cruz-Díaz D, Pérez-López FR. Osteosarcopenic obesity and fall prevention strategies. MATURITAS. 2015;80(2):126-32.
1237. Rubinacci A, Marenzana M, Cavani F, Colasante F, Villa I, Willnecker J, et al. Ovariectomy sensitizes rat cortical bone to whole-body vibration. Calcified Tissue International. 2008;82(4):316-26.
1238. Cetin MS, Ozcan Cetin EH, Canpolat U, Aydin S, Temizhan A, Topaloglu S, et al. An overlooked parameter in coronary slow flow phenomenon: Whole blood viscosity. Biomarkers in Medicine. 2015;9(12):1311-21.
1239. Avelar NCP, Simão AP, Tossige-Gomes R, Neves CDC, Mezencio B, Szmuchrowski L, et al. Oxygen consumption and heart rate during repeated squatting exercises with or without whole-body vibration in the elderly. Journal of Strength and Conditioning Research. 2011;25(12):3495-500.
1240. Rittweger J, Schiessl H, Felsenberg D. Oxygen uptake during whole-body vibration exercise: Comparison with squatting as a slow voluntary movement. European Journal of Applied Physiology. 2001;86(2):169-73.
1241. Compare A, Zarbo C, Marín E, Meloni A, Rubio-Arias JA, Berengüí R, et al. PAHA study: psychological active and healthy aging: psychological wellbeing, proactive attitude and happiness effects of whole-body vibration versus Multicomponent Training in aged women: study protocol for a randomized controlled trial. Trials. 2014;15:177.
1242. Holsgrove TP, Zeeman ME, Welch WC, Winkelstein BA. Pain After Whole-Body Vibration Exposure Is Frequency Dependent and Independent of the Resonant Frequency: Lessons From an In Vivo Rat Model. JOURNAL OF BIOMECHANICAL ENGINEERING-TRANSACTIONS OF THE ASME. 2020;142(6).
1243. Puhl RM, Luedicke J, Depierre JA. Parental concerns about weight-based victimization in youth. Child Obes. 2013;9(6):540-8.
1244. Suh Y, Puhl R, Liu S, Fleming Milici F. Parental support for policy actions to reduce weight stigma toward youth in schools and children's television programs: trends from 2011 to 2013. Child Obes. 2014;10(6):533-41.
1245. Puhl RM, Luedicke J. Parental support for policy measures and school-based efforts to address weight-based victimization of overweight youth. Int J Obes (Lond). 2014;38(4):531-8.
1246. Hu Y, Fang B, Tian X, Wang H, Tian X, Yu F, et al. Passive exercise is an effective alternative to HRT for restoring OVX induced mitochondrial dysfunction in skeletal muscle. Frontiers in Endocrinology. 2024;15.
1247. Heesterbeek M, Van der Zee EA, van Heuvelen MJG. Passive exercise to improve quality of life, activities of daily living, care burden and cognitive functioning in institutionalized older adults with dementia - a randomized controlled trial study protocol. BMC Geriatrics. 2018;18(1):1-9.
1248. Alouini S, Memic S, Couillandre A. Pelvic floor muscle training for urinary incontinence with or without biofeedback or electrostimulation in women: a systematic review. International Journal of Environmental Research & Public Health 2022 Mar;19(5):2789. 2022.
1249. Lauper M, Kuhn A, Gerber R, Luginbühl H, Radlinger L. Pelvic Floor Stimulation: What Are The Good Vibrations? NEUROUROLOGY AND URODYNAMICS. 2009;28(5):405-10.
1250. Forta NG, Schust M. Perception of fore-and-aft whole-body vibration intensity measured by two methods. Ergonomics. 2015;58(11):1800-12.
1251. Golding JF, Benson AJ. Perceptual scaling of whole-body low frequency linear oscillatory motion. Aviation Space and Environmental Medicine. 1993;64(7):636-40.
1252. Lee JY, Oh SH, Kim HR. Performance evaluation of the ZL 6000i cone-plate rotational viscometer. Clin Hemorheol Microcirc. 2020;75(2):143-9.
1253. Paul RH, Cohen RA, Moser D, Ott BR, Zawacki T, Gordon N, et al. Performance on the Mattis Dementia Rating Scale in patients with vascular dementia: relationships to neuroimaging findings. J Geriatr Psychiatry Neurol. 2001;14(1):33-6.
1254. Zanone MM, Marinucci C, Ciancio A, Cocito D, Zardo F, Spagone E, et al. Peripheral neuropathy after viral eradication with direct-acting antivirals in chronic HCV hepatitis: A prospective study. Liver International. 2021;41(11):2611-21.
1255. Tim-Yun Ong M, Fu SC, Mok SW, Franco-Obregón A, Lok-Sze Yam S, Shu-Hang Yung P. Persistent quadriceps muscle atrophy after anterior cruciate ligament reconstruction is associated with alterations in exercise-induced myokine production. Asia-Pacific Journal of Sports Medicine, Arthroscopy, Rehabilitation and Technology. 2022;29:35-42.
1256. Bergmann G, Bender A, Dymke J, Duda GN, Damm P. Physical Activities That Cause High Friction Moments at the Cup in Hip Implants. J Bone Joint Surg Am. 2018;100(19):1637-44.
1257. Skelton DA. Physical activity and osteoporosis. Bone. 2009;44:S200.
1258. Selph SS, Skelly AC, Wasson N, Dettori JR, Brodt ED, Ensrud E, et al. Physical Activity and the Health of Wheelchair Users: A Systematic Review in Multiple Sclerosis, Cerebral Palsy, and Spinal Cord Injury. Archives of Physical Medicine and Rehabilitation. 2021;102(12):2464-81.e33.
1259. Romo-Perez V, Barcala-Furelos R. PHYSICAL ACTIVITY RECOMMENDATIONS FOR THE ELDERLY: EFFECT OF RESISTANCE TRAINING ON PHYSICAL FITNESS. REVISTA DE PSICOLOGIA DEL DEPORTE. 2012;21(2):373-8.
1260. Galhardas L, Raimundo A, Del Pozo-Cruz J, Marmeleira J. Physical and Motor Fitness Tests for Older Adults Living in Nursing Homes: A Systematic Review. INTERNATIONAL JOURNAL OF ENVIRONMENTAL RESEARCH AND PUBLIC HEALTH. 2022;19(9).
1261. Vandergrift JL, Gold JE, Hanlon A, Punnett L. Physical and psychosocial ergonomic risk factors for low back pain in automobile manufacturing workers. Occupational and Environmental Medicine. 2012;69(1):29-34.
1262. Thorbjörnsson CB, Alfredsson L, Fredriksson K, Michélsen H, Punnett L, Vingård E, et al. Physical and psychosocial factors related to low back pain during a 24-year period. A nested case-control analysis. Spine (Phila Pa 1976). 2000;25(3):369-74; discussion 75.
1263. Moreira LDF, de Oliveira ML, Lirani-Galvao AP, Marin-Mio RV, dos Santos RN, Lazaretti-Castro M. Physical exercise and osteoporosis: effects of different types of exercises on bone and physical function of postmenopausal women. ARQUIVOS BRASILEIROS DE ENDOCRINOLOGIA E METABOLOGIA. 2014;58(5):514-22.
1264. Sánchez-Delgado JC, Jácome-Hortúa AM, Yoshida de Melo K, Aguilar BA, Vieira Philbois S, Dutra de Souza HC. Physical Exercise Effects on Cardiovascular Autonomic Modulation in Postmenopausal Women-A Systematic Review and Meta-Analysis. Int J Environ Res Public Health. 2023;20(3).
1265. de Sire A, Lippi L, Ammendolia A, Cisari C, Venetis K, Sajjadi E, et al. Physical exercise with or without whole-body vibration in breast cancer patients suffering from aromatase inhibitor—induced musculoskeletal symptoms: a pilot randomized clinical study. Journal of personalized medicine. 2021;11(12).
1266. Hoogendoorn WE, van Poppel MN, Bongers PM, Koes BW, Bouter LM. Physical load during work and leisure time as risk factors for back pain. Scand J Work Environ Health. 1999;25(5):387-403.
1267. Savvakis I, Adamakidou T, Kleisiaris C. Physical-activity interventions to reduce fear of falling in frail and pre-frail older adults: a systematic review of randomized controlled trials. European Geriatric Medicine. 2024;15(2):333-44.
1268. Lark SD, Wadsworth DP. Physiological, psychological and functional changes with whole body vibration exercise in the elderly: FEVER methodology and protocols. CONTEMPORARY CLINICAL TRIALS. 2015;44:129-33.
1269. Kwasńa K, Chmielewska D, Piecha M, Halski T, Taradaj J, Juras G, et al. Physiotherapy treatment of urinary stress incontinence in women. Part 2. Przeglad Menopauzalny. 2012;16(5):372-5.
1270. Lorenzetti V, Allen NB, Fornito A, Pantelis C, De Plato G, Ang A, et al. Pituitary gland volume in currently depressed and remitted depressed patients. Psychiatry Res. 2009;172(1):55-60.
1271. Velcheva I, Antonova N, Dimitrova V, Dimitrov N, Ivanov I. Plasma lipids and blood viscosity in patients with cerebrovascular disease. Clin Hemorheol Microcirc. 2006;35(1-2):155-7.
1272. Wu HC, Lee LC, Wang WJ. Plasmapheresis for hypertriglyceridemia: The association between blood viscosity and triglyceride clearance rate. J Clin Lab Anal. 2019;33(2):e22688.
1273. Nct. Platform Exercise Training. https://clinicaltrialsgov/show/NCT01239823. 2010.
1274. Haelterman E, Marcoux S, Croteau A, Dramaix M. Population-based study on occupational risk factors for preeclampsia and gestational hypertension. Scand J Work Environ Health. 2007;33(4):304-17.
1275. Burdorf A, Sorock G. Positive and negative evidence of risk factors for back disorders. Scandinavian Journal of Work, Environment and Health. 1997;23(4):243-56.
1276. Pahl A, Wehrle A, Kneis S, Gollhofer A, Finke J, Bertz H. Positive effects of whole body vibration on patient's physical capacity and quality of life during allogeneic hematopoietic cell transplantation-A randomized controlled study. Bone marrow transplantation. 2019;54:240‐1.
1277. George D, Mavvidis A, Kosmadaki I, Tsoumani S, Dallas K. THE POST ACTIVATION POTENTIATION EFFECT OF TWO DIFFERENT CONDITIONING STIMULI ON DROP JUMP PARAMETERS ON YOUNG FEMALE ARTISTIC GYMNASTS. SCIENCE OF GYMNASTICS JOURNAL. 2019;11(1):103-13.
1278. Park JH, Kia K, Srinivasan D, Kim JH. Postural balance effects from exposure to multi-axial whole-body vibration in mining vehicle operation. Appl Ergon. 2021;91:103307.
1279. Stolzenberg N, Felsenberg D, Belavy DL. Postural control is associated with muscle power in post-menopausal women with low bone mass. OSTEOPOROSIS INTERNATIONAL. 2018;29(10):2283-8.
1280. Kosar AC, Candow DG, Putland JT. Potential beneficial effects of whole-body vibration formuscle recovery after exercise. Journal of Strength and Conditioning Research. 2012;26(10):2907-11.
1281. Sebastian S, Marano G, Lee YWW, Lam TP, Müller R, Christen P. Potential of whole body vibration therapy to increase bone strength in adolescents with idiopathic scoliosis. Osteologie. 2018;27(2):A64-A5.
1282. Nawayseh N, Hamdan S. Power Absorbed by the Standing Human Body During Whole-Body Vibration Training. J Biomech Eng. 2020;142(7).
1283. Di Giminiani R, Petricola S. The Power Output-Drop Height Relationship to Determine the Optimal Dropping Intensity and to Monitor the Training Intervention. Journal of Strength and Conditioning Research. 2016;30(1):117-25.
1284. Galarneau JM, Beach J, Cherry N. Pregnancy Outcome in Women Exposed to Metal Fume in Welding: A Canadian Cohort Study. Ann Work Expo Health. 2022;66(9):1099-110.
1285. Chang S-F, Lin P-C, Yang R-S, Yang R-J. The preliminary effect of whole-body vibration intervention on improving the skeletal muscle mass index, physical fitness, and quality of life among older people with sarcopenia. BMC Geriatrics. 2018;18:1-N.PAG.
1286. Semler O, Fricke O, Vezyroglou K, Stark C, Schoenau E. Preliminary results on the mobility after whole body vibration in immobilized children and adolescents. J Musculoskelet Neuronal Interact. 2007;7(1):77-81.
1287. Nakajima S, Ino S, Ifukube T. A preliminary study of MR sickness evaluation using visual motion aftereffect for advanced driver assistance systems. Annu Int Conf IEEE Eng Med Biol Soc. 2007;2007:3044-7.
1288. Foti C, Annino G, D'Ottavio S, Sensi F, Tsarpela O, Masala S, et al. Preliminary study on the effects of high magnitude, low frequency of whole body vibration in physical activity of osteoporotic women. MEDICINA DELLO SPORT. 2009;62(1):97-106.
1289. Sciarra T, Annino G, D'Ottavio S, Sensi F, Tsarpela O, Masala S, et al. Preliminary study on the effects of high-magnitude low-frequency whole body vibration in active osteoporotic women. Clinical and Experimental Rheumatology. 2009;27(5):727.
1290. McMillan LB, Zengin A, Ebeling PR, Scott D. Prescribing Physical Activity for the Prevention and Treatment of Osteoporosis in Older Adults. HEALTHCARE. 2017;5(4).
1291. Palmer KT, Griffin MJ, Bendall H, Pannett B, Coggon D. Prevalence and pattern of occupational exposure to whole body vibration in Great Britain: findings from a national survey. Occup Environ Med. 2000;57(4):229-36.
1292. Bouchard C. Prevention of falls, prevention of osteoporosis, or both: What is the best strategy for preventing fractures in older women? Menopause. 2013;20(10):995-6.
1293. Streckmann F, Balke M, Lehmann HC, Rustler V, Koliamitra C, Elter T, et al. The preventive effect of sensorimotor- and vibration exercises on the onset of Oxaliplatin- or vinca-alkaloid induced peripheral neuropathies - STOP. BMC Cancer. 2018;18(1):62.
1294. del Pozo-Cruz JD, Alfonso-Rosa RM, Ugia JL, McVeigh JG, del Pozo-Cruz B, Sanudo B. A primary care-based randomized controlled trial of 12-weeks whole body-vibration for balance improvement in type 2 diabetes mellitus. Archives of Physical Medicine and Rehabilitation 2013 Nov;94(11):2112-2118. 2013.
1295. Cetin EHO, Cetin MS, Canpolat U, Aydin S, Aras D, Topaloglu S, et al. Prognostic significance of whole blood viscosity estimated by de Simone's formula in ST-elevation myocardial infarction. Biomarkers in Medicine. 2016;10(5):495-511.
1296. Gunstad J, Brickman AM, Paul RH, Browndyke J, Moser DJ, Ott BR, et al. Progressive morphometric and cognitive changes in vascular dementia. Arch Clin Neuropsychol. 2005;20(2):229-41.
1297. Jones MT. Progressive-overload whole-body vibration training as part of periodized, off-season strength training in trained women athletes. Journal of strength and conditioning research. 2014;28(9):2461‐9.
1298. Celletti C, Suppa A, Bianchini E, Lakin S, Toscano M, La Torre G, et al. Promoting post-stroke recovery through focal or whole body vibration: criticisms and prospects from a narrative review. Neurological sciences. 2020;41(1):11‐24.
1299. Wadsworth D, Turnbull J, Lark S. Psychological effects of whole-body vibration training in frail older adults: an open, randomized control trial. Journal of Aging and Physical Activity 2022 Feb;30(1):54-64. 2022.
1300. Poliachik SL, Khokhlova TD, Wang YN, Simon JC, Bailey MR. Pulsed focused ultrasound treatment of muscle mitigates paralysis-induced bone loss in the adjacent bone: a study in a mouse model. Ultrasound Med Biol. 2014;40(9):2113-24.
1301. Carvalho-Lima RP, Sa-Caputo DC, Moreira-Marconi E, Dionello C, Paineiras-Domingos LL, Sousa-Goncalves CR, et al. Quality of life of patients with metabolic syndrome is improved after whole body vibration exercises. African Journal of Traditional, Complementary, and Alternative Medicines 2017;14(4 Suppl):59-65. 2017.
1302. Rahmatalla S, Qiao G, DeShaw J, Kinsler R. Quantifying supine human discomfort in off-road whole-body vibration. Ergonomics. 2023;66(4):479-91.
1303. Calandrelli R, Pilato F, Massimi L, Panfili M, Di Rocco C, Colosimo C. Quantitative analysis of cranial-orbital changes in infants with anterior synostotic plagiocephaly. Childs Nerv Syst. 2018;34(9):1725-33.
1304. Pollock RD, Newham DJ, Martin FC. Randomised clinical trial of the effects of whole body vibration therapy, in addition to a supervised exercise programme, on measures of Falls related physical performance. Age and ageing. 2011;40:ii52.
1305. Tubic B, Zeijlon R, Wennergren G, Obermayer‐Pietsch B, Mårild S, Dahlgren J, et al. Randomised study of children with obesity showed that whole body vibration reduced sclerostin. Acta Paediatrica. 2019;108(3):502-13.
1306. Turner S, Torode M, Climstein M, Naughton G, Greene D, Baker MK, et al. A Randomized Controlled Trial of Whole Body Vibration Exposure on Markers of Bone Turnover in Postmenopausal Women. JOURNAL OF OSTEOPOROSIS. 2011;2011.
1307. Schonsteiner SS, Bauder Missbach H, Benner A, Mack S, Hamel T, Orth M, et al. A randomized exploratory phase 2 study in patients with chemotherapy-related peripheral neuropathy evaluating whole-body vibration training as adjunct to an integrated program including massage, passive mobilization and physical exercises. Experimental Hematology & Oncology 2017 Feb 7;6(5):Epub. 2017.
1308. Kaut O, Jacobi H, Coch C, Prochnicki A, Minnerop M, Klockgether T, et al. A randomized pilot study of stochastic vibration therapy in spinocerebellar ataxia. The Cerebellum 2014 Apr;13(2):237-242. 2014.
1309. Cidem M, Karacan I, Diracoglu D, Yildiz A, Hayri Kucuk S, Uludag M, et al. A randomized trial on the effect of bone tissue on vibration-induced muscle strength gain and vibration-induced reflex muscle activity. Balkan medical journal. 2014;31(1):11‐22.
1310. Brogårdh C, Flansbjer UB, Lexell J. Rapidly Emerging Role of Whole Body Vibration Therapy in the Management of Neurologic Diseases Besides Polio Response. ARCHIVES OF PHYSICAL MEDICINE AND REHABILITATION. 2011;92(4):677-8.
1311. Cochrane DJ, Stannard SR, Sargeant AJ, Rittweger J. The rate of muscle temperature increase during acute whole-body vibration exercise. European Journal of Applied Physiology. 2008;103(4):441-8.
1312. Friesenbichler B, Coza A, Nigg BM. Reduced elbow extension torque during vibrations. JOURNAL OF BIOMECHANICS. 2012;45(13):2203-7.
1313. Muceli S, Farina D, Kirkesola G, Katch F, Falla D. Reduced force steadiness in women with neck pain and the effect of short term vibration. JOURNAL OF ELECTROMYOGRAPHY AND KINESIOLOGY. 2011;21(2):283-90.
1314. Streckmann F, Bloch W, Lehmann H, Faude O, Baumann FT. Reducing the symptoms of chemotherapy-induced peripheral neuropathy with specific exercise interventions. Oncology research and treatment. 2016;39:10‐1.
1315. Culvenor AG, Girdwood MA, Juhl CB, Patterson BE, Haberfield MJ, Holm PM, et al. Rehabilitation after anterior cruciate ligament and meniscal injuries: a best-evidence synthesis of systematic reviews for the OPTIKNEE consensus. British journal of sports medicine. 2022;56(24):1445-53.
1316. Forster A, Lambley R, Hardy J, Young J, Smith J, Green J, et al. Rehabilitation for older people in long-term care. Cochrane Database of Systematic Reviews. 2009(1).
1317. Salhi B, Haenebalcke C, Perez-Bogerd S, Nguyen MD, Ninane V, Malfait TLA, et al. Rehabilitation in patients with radically treated respiratory cancer: a randomised controlled trial comparing two training modalities. Lung cancer (Amsterdam, Netherlands). 2015;89(2):167‐74.
1318. Arienti C, Lazzarini SG, Pollock A, Negrini S. Rehabilitation interventions for improving balance following stroke: an overview of systematic reviews. PLoS ONE 2019 Jul;14(7):e0219781. 2019.
1319. Bemben D, Stark C, Taiar R, Bernardo-Filho M. Relevance of Whole-Body Vibration Exercises on Muscle Strength/Power and Bone of Elderly Individuals. Dose-Response. 2018;16(4).
1320. Song BG, Kang N. Removal of movement artifacts and assessment of mental stress analyzing electroencephalogram of non-driving passengers under whole-body vibration. Frontiers in Neuroscience. 2024;18.
1321. McCann MR, Patel P, Pest MA, Ratneswaran A, Lalli G, Beaucage KL, et al. Repeated Exposure to High-Frequency Low-Amplitude Vibration Induces Degeneration of Murine Intervertebral Discs and Knee Joints. ARTHRITIS & RHEUMATOLOGY. 2015;67(8):2164-75.
1322. Bracko MR. Research Bites. ACSM's Health & Fitness Journal. 2013;17(3):24-5.
1323. Busch AJ, Webber SC, Richards RS, Bidonde J, Schachter CL, Schafer LA, et al. Resistance exercise training for fibromyalgia. COCHRANE DATABASE OF SYSTEMATIC REVIEWS. 2013(12).
1324. Sarabon N, Kozinc Z, Lofler S, Hofer C. Resistance exercise, electrical muscle stimulation, and whole-body vibration in older adults: systematic review and meta-analysis of randomized controlled trials. Journal of Clinical Medicine 2020 Sep;9(9):2902. 2020.
1325. Eftekhari E, Mostahfezian M, Etemadifar M, Zafari A. Resistance training and vibration improve muscle strength and functional capacity in female patients with multiple sclerosis. Asian Journal of Sports Medicine. 2012;3(4):279-84.
1326. Kalapotharakos VI, Tokmakidis SP, Smilios I, Michalopoulos M, Gliatis J, Godolias G. Resistance training in older women: effect on vertical jump and functional performance. JOURNAL OF SPORTS MEDICINE AND PHYSICAL FITNESS. 2005;45(4):570-5.
1327. Moreira AM, Galvão MLC, de Araújo HAG, Silva AT, Dos Reis LM, Silva AM, et al. Respiratory muscle training and vibrational therapy in patients with Parkinson’s disease. Revista Neurociencias. 2015;23(4):479-85.
1328. Stewart VH, Saunders DH, Greig CA. Responsiveness of muscle size and strength to physical training in very elderly people: A systematic review. Scandinavian Journal of Medicine and Science in Sports. 2014;24(1):e1-e10.
1329. Xie L, Yi SX, Peng QF, Liu P, Jiang H. Retrospective study of effect of whole-body vibration training on balance and walking function in stroke patients. World Journal of Clinical Cases. 2021;9(22):6268-77.
1330. Foster H, Weatherman MN, Hudson J, de Vera Barredo R. Review of research evidence on the effectiveness of whole body vibration on the muscle strength and muscle mass of adults 55 years old and older. Journal of the National Society of Allied Health 2015 Fall;12(1):36-44. 2015.
1331. Rodrigues F, Domingos C, Monteiro D, Morouco P. A Review on Aging, Sarcopenia, Falls, and Resistance Training in Community-Dwelling Older Adults. INTERNATIONAL JOURNAL OF ENVIRONMENTAL RESEARCH AND PUBLIC HEALTH. 2022;19(2).
1332. Hughes JM, Charkoudian N, Barnes JN, Morgan BJ. Revisiting the Debate: Does Exercise Build Strong Bones in the Mature and Senescent Skeleton? FRONTIERS IN PHYSIOLOGY. 2016;7.
1333. Sadeh M, Dory A, Lev D, Yosovich K, Dabby R. Riboflavin-responsive lipid-storage myopathy in elderly patients. Journal of the Neurological Sciences. 2024;456.
1334. Schmidt AL, Paskoff G, Shender BS, Bass CR. Risk of lumbar spine injury from cyclic compressive loading. Spine (03622436). 2012;37(26):E1614-21.
1335. Brooke-Wavell K, Mansfield NJ. Risks and benefits of whole body vibration training in older people. AGE AND AGEING. 2009;38(3):254-5.
1336. Simonovich IT, Rinot N, Keren Y. ROAD CYCLING, DECREASED BONE DENSITY AND PROXIMAL FEMORAL FRACTURES. Harefuah. 2022;161(6):361-6.
1337. Wang W, Rakheja S, Boileau PÉ. The role of seat geometry and posture on the mechanical energy absorption characteristics of seated occupants under vertical vibration. International Journal of Industrial Ergonomics. 2006;36(2):171-84.
1338. Swe M, Benjamin B, Tun AA, Sugathan S. Role of the whole body vibration machine in the prevention and management of osteoporosis in old age: A systematic review. Malaysian Journal of Medical Sciences. 2016;23(5):8-16.
1339. Kaeding TS. Sarcopenia and whole body vibration training: An overview. ZEITSCHRIFT FUR GERONTOLOGIE UND GERIATRIE. 2009;42(2):88-92.
1340. Bauer JM, Kaiser MJ, Sieber CC. Sarcopenia in nursing home residents. Journal of the American Medical Directors Association. 2008;9(8):545-51.
1341. Papadopoulou SK. Sarcopenia: A contemporary health problem among older adult populations. Nutrients. 2020;12(5).
1342. Batsis JA, Villareal DT. Sarcopenic obesity in older adults: aetiology, epidemiology and treatment strategies. Nature Reviews Endocrinology. 2018;14(9):513-37.
1343. Sharma-Ghimire P, Chen Z, Sherk V, Bemben M, Bemben D. Sclerostin and parathyroid hormone responses to acute whole-body vibration and resistance exercise in young women. Journal of Bone & Mineral Metabolism. 2019;37(2):358-67.
1344. Dewangan KN, Shahmir A, Rakheja S, Marcotte P. Seated body apparent mass response to vertical whole body vibration: Gender and anthropometric effects. INTERNATIONAL JOURNAL OF INDUSTRIAL ERGONOMICS. 2013;43(4):375-91.
1345. Seidel H. Selected health risks caused by long‐term, whole‐body vibration. American Journal of Industrial Medicine. 1993;23(4):589-604.
1346. Aguilera-Castells J, Buscà B, Arboix-Alió J, Miró A, Fort-Vanmeerhaeghe A, Peña J. sEMG Activity in Superimposed Vibration on Suspended Supine Bridge and Hamstring Curl. FRONTIERS IN PHYSIOLOGY. 2021;12.
1347. Bähr FS, Gess B, Müller M, Romanzetti S, Gadermayr M, Kuhl C, et al. Semi-automatic mri muscle volumetry to diagnose and monitor hereditary and acquired polyneuropathies. Brain Sciences. 2021;11(2):1-14.
1348. Backman M, Wengström Y. SensiEx: A randomized pilot trial Sensorimotor training using whole body vibration exercise to reduce chemotherapy-induced peripheral neuropathy after treatment for breast cancer. Annals of Oncology. 2023;34:S1246.
1349. Schenk A, Oberste M, Heller A, Schürhörster A, Bloch W, Streckmann F, et al. Sensorimotor training and whole-body vibration training have the potential to reduce motor and sensory symptoms of chemotherapy-induced peripheral neuropathy-a randomized controlled pilot trial. Supportive Care in Cancer. 2019;27(7):2471-8.
1350. Rogan S, Radlinger L, Baur H, Schmidtbleicher D, de Bie RA, de Bruin ED. Sensory-motor training targeting motor dysfunction and muscle weakness in long-term care elderly combined with motivational strategies: a single blind randomized controlled study. European Review of Aging and Physical Activity 2016 May 28;13(4):Epub. 2016.
1351. Hartard M, Seiler A, Spitzenpfeil P, Engel L, Hartard D, Fenneni MA, et al. Sex-specific response to whole-body vibration training: a randomized controlled trial. Biology of Sport. 2022;39(1):207-17.
1352. Ray JJ, Alvarez AD, Ulbrich SL, Lessner-Eisenberg S, Satahoo SS, Meizoso JP, et al. Shake It Off: a Randomized Pilot Study of the Effect of Whole Body Vibration on Pain in Healing Burn Wounds. Journal of burn care & research. 2017;38(4):e756‐e64.
1353. Tekin F, Kavlak E. Short and long-term effects of whole-body vibration on spasticity and motor performance in children with hemiparetic cerebral palsy. Perceptual and Motor Skills 2021 Jun;128(3):1107-1129. 2021.
1354. Despina T, George D, George T, Sotiris P, Alessandra DC, George K, et al. Short-term effect of whole-body vibration training on balance, flexibility and lower limb explosive strength in elite rhythmic gymnasts. Hum Mov Sci. 2014;33:149-58.
1355. Cochrane DJ, Legg SJ, Hooker MJ. The short-term effect of whole-body vibration training on vertical jump, sprint, and agility performance. Journal of strength and conditioning research. 2004;18(4):828‐32.
1356. Arauz YLA, van der Zee EA, Kamsma YPT, van Heuvelen MJG. Short-term effects of side-alternating Whole-Body Vibration on cognitive function of young adults. PLOS ONE. 2023;18(1).
1357. Kordi Yoosefinejad A, Shadmehr A, Olyaei G, Talebian S, Bagheri H, Mohajeri-Tehrani MR. Short-term effects of the whole-body vibration on the balance and muscle strength of type 2 diabetic patients with peripheral neuropathy: a quasi-randomized-controlled trial study. Journal of diabetes and metabolic disorders. 2015;14(1).
1358. King LK, Almeida QJ, Ahonen H. Short-term effects of vibration therapy on motor impairments in Parkinson's disease. Neurorehabilitation 2009;25(4):297-306. 2009.
1359. Jung-Sun L, Chang-Yong K, Hyeong-Dong K. Short-Term Effects of Whole-Body Vibration Combined with Task-Related Training on Upper Extremity Function, Spasticity, and Grip Strength in Subjects with Poststroke Hemiplegia. American Journal of Physical Medicine & Rehabilitation. 2016;95(8):608-17.
1360. Tsuji T, Kitano N, Tsunoda K, Himori E, Okura T, Tanaka K. Short-term effects of whole-body vibration on functional mobility and flexibility in healthy, older adults: a randomized crossover study. Journal of geriatric physical therapy (2001). 2014;37(2):58‐64.
1361. de Ruiter CJ, van der Linden RM, van der Zijden MJA, Hollander AP, de Haan A. Short-term effects of whole-body vibration on maximal voluntary isometric knee extensor force and rate of force rise. European Journal of Applied Physiology. 2003;88(4-5):472-5.
1362. Gusso S, Munns CF, Cutfield WS, Hofman PL. Short-term whole body vibration therapy improves bone density and muscle function in adolescents with cerebral palsy. Endocrine Reviews. 2013;34(3).
1363. Spiliopoulou SI, Amiridis IG, Tsigganos G, Hatzitaki V. Side-alternating vibration training for balance and ankle muscle strength in untrained women. Journal of Athletic Training. 2013;48(5):590-600.
1364. Khan A, Ramage B, Robu I, Benard L. Side-Alternating Vibration Training ImprovesMuscle Performance in a Patient with Late-Onset Pompe Disease. CASE REPORTS IN MEDICINE. 2009;2009.
1365. Micke F, Weissenfels A, Wirtz N, von Stengel S, Dormann U, Kohl M, et al. Similar Pain Intensity Reductions and Trunk Strength Improvements Following Whole-Body Electromyostimulation vs. Whole-Body Vibration vs. Conventional Back-Strengthening Training in Chronic Non-specific Low Back Pain Patients: a Three-Armed Randomized Controlled Trial. Frontiers in physiology. 2021;12.
1366. Hatori K, Camargos G, Chatterjee M, Faot F, Sasaki K, Duyck J, et al. Single and combined effect of high-frequency loading and bisphosphonate treatment on the bone micro-architecture of ovariectomized rats. OSTEOPOROSIS INTERNATIONAL. 2015;26(1):303-13.
1367. Ramos LAX, Rodrigues FTM, Shirahige L, de Fatima Alcantara Barros M, de Carvalho AGC, Guerino MR, et al. A single whole body vibration session influences quadriceps muscle strength, functional mobility and balance of elderly with osteopenia and/or osteoporosis? Pragmatic clinical trial. Journal of diabetes and metabolic disorders. 2019;18(1):73‐80.
1368. Bosveld R, Field-Fote EC. Single-dose effects of whole body vibration on quadriceps strength in individuals with motor-incomplete spinal cord injury. Journal of Spinal Cord Medicine. 2015;38(6):784-91.
1369. Alentorn-Geli E, Padilla J, Moras G, Haro CL, Fernandez-Sola J. Six weeks of whole-body vibration exercise improves pain and fatigue in women with fibromyalgia. Journal of Alternative & Complementary Medicine 2008 Oct;14(8):975-981. 2008.
1370. Alvarez-Alvarado S, Pacilio J, Jaime SJ, Campbell JC, Post J, Figueroa A. Six-Weeks Loaded Versus Unloaded Whole-Body Vibration Training on Arterial Function and Muscle Strength in Overweight/Obese Young Women. MEDICINE AND SCIENCE IN SPORTS AND EXERCISE. 2016;48(5):416-.
1371. Huseman CJ, Sigler DH, Welsh TH, Suva LJ, Vogelsang MM, Dominguez BJ, et al. Skeletal response to whole body vibration and dietary calcium and phosphorus in growing pigs. Journal of animal science. 2019;97(8):3369-78.
1372. Palop-Montoro MV, Lozano-Aguilera E, Arteaga-Checa M, Serrano-Huete V, Párraga-Montilla JA, Manzano-Sánchez D. Sleep Quality in Older Women: Effects of a Vibration Training Program. APPLIED SCIENCES-BASEL. 2020;10(23).
1373. Hortobágyi T, Lesinski M, Fernandez-Del-Olmo M, Granacher U. Small and inconsistent effects of whole body vibration on athletic performance: a systematic review and meta-analysis. European Journal of Applied Physiology. 2015;115(8):1605-25.
1374. Lin K, Hunter SM. Somatosensory stimulation to improve function in the upper limb after stroke: A systematic review. International Journal of Stroke. 2018;13(3):42.
1375. McKay MJ, Baldwin JN, Ferreira P, Simic M, Burns J, Vanicek N, et al. Spatiotemporal and plantar pressure patterns of 1000 healthy individuals aged 3–101 years. Gait and Posture. 2017;58:78-87.
1376. Kipp K, Johnson ST, Hoffman MA. Spectral properties of H-reflex recordings after an acute bout of whole-body vibration. Journal of Strength and Conditioning Research. 2012;26(7):1915-9.
1377. Gómez MPA, Weidekamm C, Aparisi F, Bazzocchi A. Sports and Metabolic Bone Disease. SEMINARS IN MUSCULOSKELETAL RADIOLOGY. 2020;24(03):277-89.
1378. Cheung WH, Sun MH, Zheng YP, Chu WCW, Leung AHC, Qin L, et al. STIMULATED ANGIOGENESIS FOR FRACTURE HEALING AUGMENTED BY LOW-MAGNITUDE, HIGH-FREQUENCY VIBRATION IN A RAT MODEL-EVALUATION OF PULSED-WAVE DOPPLER, 3-D POWER DOPPLER ULTRASONOGRAPHY AND MICRO-CT MICROANGIOGRAPHY. ULTRASOUND IN MEDICINE AND BIOLOGY. 2012;38(12):2120-9.
1379. Kaut O, Allert N, Coch C, Paus S, Grzeska A, Minnerop M, et al. Stochastic resonance therapy in Parkinson's disease. Neurorehabilitation 2011;28(4):353-358. 2011.
1380. Faes Y, Maguire C, Notari M, Elfering A. Stochastic resonance training improves balance and musculoskeletal well-being in office workers: a controlled preventive intervention study. Rehabilitation Research and Practice 2018 Sep 13;(5070536):Epub. 2018.
1381. Burger C, Schade V, Lindner C, Radlinger L, Elfering A. Stochastic resonance training reduces musculoskeletal symptoms in metal manufacturing workers: a controlled preventive intervention study. Work 2012;42(2):269-278. 2012.
1382. Elfering A, Thomann J, Schade V, Radlinger L. Stochastic resonance whole body vibration reduces musculoskeletal pain: a randomized controlled trial. World Journal of Orthopedics 2011 Dec 18;2(12):116-120. 2011.
1383. Rogan S, Hilfiker R, Schmid S, Radlinger L. Stochastic resonance whole-body vibration training for chair rising performance on untrained elderly: a pilot study. Archives of gerontology and geriatrics. 2012;55(2):468‐73.
1384. Ceklic U, Sarabon N. STRENGTH AND JUMPING ASYMMETRIES IN GYMNAST AND THEIR NON-GYMNAST PEERS. SCIENCE OF GYMNASTICS JOURNAL. 2021;13(3):411-24.
1385. Delecluse C, Roelants M, Verschueren S. Strength increase after whole-body vibration compared with resistance training. Medicine & Science in Sports & Exercise. 2003;35(6):1033-41.
1386. Nordlund MM, Thorstensson A. Strength training effects of whole-body vibration? SCANDINAVIAN JOURNAL OF MEDICINE & SCIENCE IN SPORTS. 2007;17(1):12-7.
1387. Weier AT, Kidgell DJ. Strength training with superimposed whole body vibration does not preferentially modulate cortical plasticity. Scientific World Journal. 2012:876328-.
1388. Rui M, Shuai Y, YaWen J, Yan L, Nan W, Xiaojuan G. Study On The Effect Of WBV On Physical Function Of Postmenopausal Women From 45 To 65 Years Old...2021 ACSM Annual Meeting & World Congresses. Medicine & Science in Sports & Exercise. 2021;53(8S):25-.
1389. Hacaambwa TM, Giacomin J. Subjective response to seated fore-and-aft direction whole-body vibration. International Journal of Industrial Ergonomics. 2007;37(1):61-72.
1390. Maeda S, Shibata N. Subjective scaling of hand-arm vibration. Industrial Health. 2008;46(2):118-24.
1391. el-Bagalaty AE, Ismaeel MMI. Suit therapy versus whole-body vibration on bone mineral density in children with spastic diplegia. Journal of Musculoskeletal & Neuronal Interactions 2021 Mar;21(1):79-84. 2021.
1392. Carson RG, Popple AE, Verschueren SMP, Riek S. Superimposed vibration confers no additional benefit compared with resistance training alone. Scandinavian Journal of Medicine and Science in Sports. 2010;20(6):827-33.
1393. Buscà B, Aguilera-Castells J, Arboix-Alió J, Miró A, Fort-Vanmeerhaeghe A, Huertas P, et al. Superimposed vibration on suspended push-ups. PEERJ. 2022;10.
1394. Ambegaonkar JP, Chong L, Joshi P. Supplemental Training in Dance: A Systematic Review. Physical Medicine and Rehabilitation Clinics of North America. 2021;32(1):117-35.
1395. Gómez-Bruton A, Gonzalez-Agüero A, Casajús JA, Rodríguez GV. Swimming training repercussion on metabolic and structural bone development; benefits of the incorporation of whole body vibration or pilometric training; the RENACIMIENTO project. Nutricion Hospitalaria. 2014;30(2):399-409.
1396. Hazell TJ, Lemon PWR. Synchronous whole-body vibration increases VO 2 during and following acute exercise. European Journal of Applied Physiology. 2012;112(2):413-20.
1397. DadeMatthews OO, Agostinelli PJ, Neal FK, Oladipupo SO, Hirschhorn RM, Wilson AE, et al. Systematic review and meta-analyses on the effects of whole-body vibration on bone health. COMPLEMENTARY THERAPIES IN MEDICINE. 2022;65.
1398. Vernetta M, Peláez-Barrios EM, López-Bedoya J. Systematic review of flexibility tests in gymnastics. JOURNAL OF HUMAN SPORT AND EXERCISE. 2022;17(1):58-73.
1399. Glickman LB, Geigle PR, Paleg GS. A systematic review of supported standing programs. Journal of Pediatric Rehabilitation Medicine 2010;3(3):197-213. 2010.
1400. Ahmed SS, Weber T, Sirek A, editors. Systematic Review of the Effectiveness of Spaceflight Passive Countermeasures. Proceedings of the International Astronautical Congress, IAC; 2022.
1401. Ahmed SS, Goswami N, Sirek A, Green DA, Winnard A, Fiebig L, et al. Systematic review of the effectiveness of standalone passive countermeasures on microgravity-induced physiologic deconditioning. npj Microgravity. 2024;10(1).
1402. Morgan PE, Dobson FL, McGinley JL. A systematic review of the efficacy of conservative interventions on the gait of ambulant adults with cerebral palsy. Journal of Developmental and Physical Disabilities 2014 Oct;26(5):633-654. 2014.
1403. Sanudo B, de Hoyo M, del Pozo-Cruz J, Carrasco L, del Pozo-Cruz B, Tejero S, et al. A systematic review of the exercise effect on bone health: the importance of assessing mechanical loading in perimenopausal and postmenopausal women. Menopause (10723714). 2017;24(10):1208-16.
1404. Sa-Caputo DC, Costa-Cavalcanti R, Carvalho-Lima RP, Arnobio A, Bernardo RM, Ronikeile-Costa P, et al. Systematic review of whole body vibration exercises in the treatment of cerebral palsy: brief report. Developmental Neurorehabilitation 2016;19(5):327-333. 2016.
1405. Peitz M, Behringer M, Granacher U. A systematic review on the effects of resistance and plyometric training on physical fitness in youth-What do comparative studies tell us? PLOS ONE. 2018;13(10).
1406. Zhu YQ, Peng N, Zhou M, Liu PP, Qi XL, Wang N, et al. Tai Chi and whole-body vibrating therapy in sarcopenic men in advanced old age: a clinical randomized controlled trial. European Journal of Ageing 2019 Sep;16(3):273-282. 2019.
1407. Zhu YQ, Peng N. Tai chi and whole-body vibration therapy in the elderly: a randomized controlled trial. Journal of the American Geriatrics Society. 2016;64:S373‐.
1408. Liu L, Duan JA, Tang Y, Guo J, Yang N, Ma H, et al. Taoren-Honghua herb pair and its main components promoting blood circulation through influencing on hemorheology, plasma coagulation and platelet aggregation. J Ethnopharmacol. 2012;139(2):381-7.
1409. Milanese C, Piscitelli F, Zenti MG, Moghetti P, Sandri M, Zancanaro C. Ten-week whole-body vibration training improves body composition and muscle strength in obese women. International Journal of Medical Sciences. 2013;10(3):307-11.
1410. Vingren JL, Kraemer WJ, Ratamess NA, Anderson JM, Volek JS, Maresh CM. Testosterone Physiology in Resistance Exercise and Training The Up-Stream Regulatory Elements. SPORTS MEDICINE. 2010;40(12):1037-53.
1411. Fatima M, Brennan-Olsen SL, Duque G. Therapeutic approaches to osteosarcopenia: insights for the clinician. THERAPEUTIC ADVANCES IN MUSCULOSKELETAL DISEASE. 2019;11.
1412. Park YG, Kwon BS, Park JW, Cha DY, Nam KY, Sim KB, et al. Therapeutic effect of whole body vibration on chronic knee osteoarthritis. Annals of Rehabilitation Medicine 2013 Aug;37(4):505-515. 2013.
1413. Zafar H, Alghadir A, Anwer S, al-Eisa E. Therapeutic effects of whole body vibration training in knee osteoarthritis: a systematic review and meta-analysis Archives of Physical Medicine and Rehabilitation 2015 Aug;96(8):1525-1532. 2015.
1414. Mingorance JA, Montoya P, Miranda JGV, Riquelme I. The Therapeutic Effects of Whole-Body Vibration in Patients With Fibromyalgia. A Randomized Controlled Trial. Frontiers in neurology. 2021;12.
1415. Chen J, Ruan H, Liu Y, Bao J, Xu H, Yao M, et al. Therapeutic effects of whole-body vibration on fracture healing in ovariectomized rats: a systematic review and meta-analysis. Menopause. 2018;26(6):677-86.
1416. Li Q, Liang L, Gao C, Zong B. Therapeutic effects of whole-body vibration on postmenopausal women with osteoporosis: a systematic review and meta-analysis. Braz J Med Biol Res. 2024;57:e13996.
1417. Fabregat-Fernández J, Rodríguez-Pérez V, Llamas-Ramos R, López-Rodríguez AF, Seco-Calvo J, Llamas-Ramos I. Therapeutic Exercise Intervention Using Vibration Platforms for Glycemic Control in Type 2 Diabetes: A Pilot Study. JOURNAL OF CLINICAL MEDICINE. 2023;12(20).
1418. Vanleene M, Shefelbine SJ. Therapeutic impact of low amplitude high frequency whole body vibrations on the osteogenesis imperfecta mouse bone. Bone. 2013;53(2):507-14.
1419. Prioreschi A, Tikly M, McVeigh JA. A three month controlled intervention of intermittent whole body vibration designed to improve functional ability and attenuate bone loss in patients with rheumatoid arthritis. BMC Musculoskeletal Disorders. 2014;15(1):403-.
1420. Edwards PI, Holsgrove TP. Thunder road - whole-body vibration during road cycling, and the effect of different seatpost designs to minimise it. J Sports Sci. 2021;39(5):489-95.
1421. Gusi N, Parraca JA, Olivares PR, Leal A, Adsuar JC. Tilt vibratory exercise and the dynamic balance in fibromyalgia: a randomized controlled trial. Arthritis Care & Research 2010 Aug;62(8):1072-1078. 2010.
1422. Olivares PR, Gusi N, Parraca JA, Adsuar JC, del Pozo-Cruz B. Tilting whole body vibration improves quality of life in women with fibromyalgia: a randomized controlled trial. Journal of Alternative & Complementary Medicine 2011 Aug;17(8):723-728. 2011.
1423. Troy Blackburn J, Dewig DR, Johnston CD. Time course of the effects of vibration on quadriceps function in individuals with anterior cruciate ligament reconstruction. J Electromyogr Kinesiol. 2021;56:102508.
1424. Maikala RV. Tissue oxygenation and blood volume and whole-body metabolic responses during low-back intensive occupational tasks: University of Alberta (Canada); 2002.
1425. Imtiyaz S, Veqar Z, Shareef MY. To Compare the Effect of Vibration Therapy and Massage in Prevention of Delayed Onset Muscle Soreness (DOMS). JOURNAL OF CLINICAL AND DIAGNOSTIC RESEARCH. 2014;8(1):133-6.
1426. Rubak TS, Svendsen SW, Søballe K, Frost P. Total hip replacement due to primary osteoarthritis in relation to cumulative occupational exposures and lifestyle factors: a nationwide nested case-control study. Arthritis Care Res (Hoboken). 2014;66(10):1496-505.
1427. Wuestefeld A, Fuermaier ABM, Bernardo-Filho M, da Cunha de Sá-Caputo D, Rittweger J, Schoenau E, et al. Towards reporting guidelines of research using whole-body vibration as training or treatment regimen in human subjects-A Delphi consensus study. PLoS One. 2020;15(7):e0235905.
1428. Perez-Gomez J, Calbet JAL. Training methods to improve vertical jump performance. JOURNAL OF SPORTS MEDICINE AND PHYSICAL FITNESS. 2013;53(4):339-57.
1429. Almstedt HC, Grote S, Perez SE, Shoepe TC, Strand SL, Tarleton HP. Training-related improvements in musculoskeletal health and balance: a 13-week pilot study of female cancer survivors. EUROPEAN JOURNAL OF CANCER CARE. 2017;26(2).
1430. Macdermid PW, Fink PW, Stannard SR. Transference of 3D accelerations during cross country mountain biking. Journal of Biomechanics. 2014;47(8):1829-37.
1431. Marcucci R, Mannini L, Andrei V, Bandinelli B, Gori AM, Fatucchi S, et al. Transient stress-related hyperviscosity and endothelial dysfunction in Takotsubo syndrome: a time course study. Heart Vessels. 2022;37(10):1776-84.
1432. Lam FMH, Tang CY, Kwok TCY, Pang MYC. Transmissibility and waveform purity of whole-body vibrations in older adults. Clinical Biomechanics. 2018;51:82-90.
1433. Rubin C, Pope M, Fritton JC, Magnusson M, Hansson T, McLeod K. Transmissibility of 15-hertz to 35-hertz vibrations to the human hip and lumbar spine: determining the physiologic feasibility of delivering low-level anabolic mechanical stimuli to skeletal regions at greatest risk of fracture because of osteoporosis. Spine (Phila Pa 1976). 2003;28(23):2621-7.
1434. Kiiski J, Heinonen A, Jaervinen TL, Kannus P, Sievänen H. Transmission of vertical whole body vibration to the human body. JOURNAL OF BONE AND MINERAL RESEARCH. 2008;23(8):1318-25.
1435. Nawayseh N. Transmission of vibration from a vibrating plate to the head of standing people. SPORTS BIOMECHANICS. 2019;18(5):482-500.
1436. Bressel E, Smith G, Branscomb J. Transmission of whole body vibration in children while standing. Clin Biomech (Bristol). 2010;25(2):181-6.
1437. Spain L, Yang L, Wilkinson JM, McCloskey E. Transmission of whole body vibration-Comparison of three vibration platforms in healthy subjects. BONE. 2021;144.
1438. Tankisheva E, Jonkers I, Boonen S, Delecluse C, V Lenthe G, Druyts HLJ, et al. Transmission of whole-body vibration and its effect on muscle activation. 2013. p. 2533-41.
1439. Yao W, Yang C, Wen Y, Zhang W, Zhang X, Ma Q, et al. Treatment effects and mechanisms of Yujin Powder on rat model of large intestine dampness-heat syndrome. J Ethnopharmacol. 2017;202:265-80.
1440. Brown CD, Kieran M, Thomas LL, Zhao ZH, Larsen R, Friedman EA. Treatment of azotemic, nonoliguric, anemic patients with human recombinant erythropoietin raises whole-blood viscosity proportional to hematocrit. Nephron. 1991;59(3):394-8.
1441. Rittweger J, Just K, Kautzsch K, Reeg P, Felsenberg D, Rittweger J, et al. Treatment of chronic lower back pain with lumbar extension and whole-body vibration exercise: a randomized controlled trial. Spine (03622436). 2002;27(17):1829-34.
1442. Boucher JA, Abboud J, Dubois JD, Legault E, Descarreaux M, Henchoz Y. Trunk neuromuscular responses to a single whole-body vibration session in patients with chronic low back pain: a cross-sectional study. J Manipulative Physiol Ther. 2013;36(9):564-71.
1443. Pérez-Gómez J, Adsuar JC, García-Gordillo M, Muñoz P, Romo L, Maynar M, et al. Twelve Weeks of Whole Body Vibration Training Improve Regucalcin, Body Composition and Physical Fitness in Postmenopausal Women: A Pilot Study. Int J Environ Res Public Health. 2020;17(11).
1444. Handler A, Bergo C, Dominik B, Bier E, Caskey R. A two-generation approach to postpartum care: Building on the well-baby visit. Birth. 2021;48(3):347-56.
1445. Howard B, Sesek R, Bloswick D. Typical whole body vibration exposure magnitudes encountered in the open pit mining industry. Work. 2009;34(3):297-303.
1446. Kasser SL, Jacobs JV. Understanding and treating balance impairment in multiple sclerosis. Journal of Clinical Outcomes Management. 2014;21(9):419-32.
1447. Nct. Use of a Reproductive Life Planning Tool at the Pediatric Well-Baby Visit With Postpartum Women. https://clinicaltrialsgov/show/NCT03448289. 2018.
1448. Dessy LA, Monarca C, Grasso F, Saggini A, Buccheri EM, Saggini R, et al. The use of mechanical acoustic vibrations to improve abdominal contour. AESTHETIC PLASTIC SURGERY. 2008;32(2):339-45.
1449. Monteiro-Oliveira BB, Coelho-Oliveira AC, Paineiras-Domingos LL, Sonza A, Sá-Caputo DDC, Bernardo-Filho M. Use of surface electromyography to evaluate effects of whole-body vibration exercises on neuromuscular activation and muscle strength in the elderly: a systematic review. Disability and rehabilitation. 2022;44(24):7368-77.
1450. Cardinale M, Bosco C. The use of vibration as an exercise intervention. Exercise and Sport Sciences Reviews. 2003;31(1):3-7.
1451. Tomás R, Lee V, Going S. THE USE OF VIBRATION EXERCISE IN CLINICAL POPULATIONS. ACSMS HEALTH & FITNESS JOURNAL. 2011;15(6):25-31.
1452. Chulvi-Medrano I, Sañudo B, Masiá-Tortosa L, Da Silva-Grigoletto M. The Use of vibration platforms in fibromyalgia syndrome: Future prospects. Journal of Musculoskeletal Pain. 2013;21(2):165-72.
1453. Nowak-Lis A, Nowak Z, Gabrys T, Szmatlan-Gabrys U, Batalik L, Knappova V. The Use of Vibration Training in Men after Myocardial Infarction. International Journal of Environmental Research and Public Health. 2022;19(6).
1454. Drks. Use of whole body vibration training as a company-facilitated sports activitiy fpr people with chronic back disorders. https://trialsearchwhoint/Trial2aspx?TrialID=DRKS00003352. 2011.
1455. Peretti AL, Ciqueleiro RT, Flores LJF, Bertolini GRF. Use of whole-body vibration as osteoporosis treatment in postmenopausal women: a systematic review. European Journal of Clinical and Experimental Medicine. 2019;17(2):146-52.
1456. Ekizler FA, Cay S, Tak BT, Kanat S, Kafes H, Cetin EHO, et al. Usefulness of the whole blood viscosity to predict stent thrombosis in ST-elevation myocardial infarction. Biomark Med. 2019;13(15):1307-20.
1457. Ramage B, Robu I, Khan A. Usefulness of three-dimensional gait assessment in monitoring function in patients with lysosomal storage diseases. Molecular Genetics and Metabolism. 2009;98(1-2):86.
1458. Wolf EJ, Cooper MSRA, Digiovine CP, Boninger ML, Guo S. Using the absorbed power method to evaluate effectiveness of vibration absorption of selected seat cushions during manual wheelchair propulsion. Medical Engineering and Physics. 2004;26(9 SPEC.ISS.):799-806.
1459. del Pozo-Cruz B, Adsuar JC, Parraca JA, del Pozo-Cruz J, Olivares PR, Gusi N. Using whole-body vibration training in patients affected with common neurological diseases: a systematic literature review. Journal of Alternative & Complementary Medicine 2012 Jan;18(1):29-41. 2012.
1460. Doyle A, Chalmers K, Chinn D, Dall N, McNeill F, Grant C. The utility of whole body vibration exercise in haemodialysis patients: A pilot study. Nephrology Dialysis Transplantation. 2016;31:i546.
1461. Majerník J, Dziaková M, Živcák J, editors. Utilization of single whole body vibration training unit in rehabilitation of elderly patients with neurological disorders. Proceedings of the 2015 Federated Conference on Computer Science and Information Systems, FedCSIS 2015; 2015.
1462. Abercromby AFJ, Amonette WE, Layne CS, McFarlin BK, Hinman MR, Paloski WH. Variation neuromuscular responses during acute whole-body vibration exercise. Medicine & Science in Sports & Exercise. 2007;39(9):1642-50.
1463. Perchthaler D, Horstmann T, Grau S. Variations in neuromuscular activity of thigh muscles during whole-body vibration in consideration of different biomechanical variables. Journal of Sports Science and Medicine. 2013;12(3):439-46.
1464. Chung P, Liu C, Wang H, Liu Y, Chuang L, Shiang TY. Various performance-enhancing effects from the same intensity of whole-body vibration training. Journal of Sport and Health Science. 2017;6(3):333-9.
1465. Pessoa MF, Brandão DC, Sá RB, Barcelar JM, Rocha TDS, Souza HCM, et al. Vibrating Platform Training Improves Respiratory Muscle Strength, Quality of Life, and Inspiratory Capacity in the Elderly Adults: a Randomized Controlled Trial. Journals of gerontology Series A, Biological sciences and medical sciences. 2017;72(5):683‐8.
1466. Yokoi H, Take Y, Uchida R, Magome T, Shimomura K, Mae T, et al. Vibration acceleration promotes endochondral formation during fracture healing through cellular chondrogenic differentiation. PLoS ONE. 2020;15(3).
1467. Irct2014061517743N. Vibration and Creatine Effects on Physical Fitness. https://trialsearchwhoint/Trial2aspx?TrialID=IRCT2014061517743N2. 2014.
1468. Jones A, Duran I, Stark C, Spiess K, Semler O, Schoenau E. Vibration assisted rehabilitation in patients with Pompe disease: A case series. Journal of Musculoskeletal Neuronal Interactions. 2022;22(2):284-91.
1469. Spiliopoulou SI, Amiridis IG, Tsigganos G, Economides D, Kellis E. Vibration Effects on Static Balance and Strength. INTERNATIONAL JOURNAL OF SPORTS MEDICINE. 2010;31(9):610-6.
1470. Cardinale M, Rittweger J. Vibration exercise makes your muscles and bones stronger: Fact or fiction? Journal of the British Menopause Society. 2006;12(1):12-8.
1471. Abercromby AFJ, Amonette WE, Layne CS, McFarlin BK, Hinman MR, Paloski WH. Vibration exposure and biodynamic responses during whole-body vibration training. Medicine and Science in Sports and Exercise. 2007;39(10):1794-800.
1472. Wolf E, Pearlman J, Cooper RA, Fitzgerald SG, Kelleher A, Collins DM, et al. Vibration exposure of individuals using wheelchairs over sidewalk surfaces. Disability & Rehabilitation. 2005;27(23):1443-9.
1473. Blackburn T, Padua DA, Pietrosimone B, Schwartz TA, Spang JT, Goodwin JS, et al. Vibration improves gait biomechanics linked to posttraumatic knee osteoarthritis following anterior cruciate ligament injury. Journal of Orthopaedic Research. 2021;39(5):1113-22.
1474. Stolzenberg N, BelavÃ½ DL, Rawer R, Felsenberg D. Vibration or Balance Training on Neuromuscular Performance in Osteopenic Women. International Journal of Sports Medicine. 2013;34(11):956-62.
1475. Novotny SA, Mehta H, Lowe DA, Nuckley DJ. Vibration platform for mice to deliver precise, low intensity mechanical signals to the musculoskeleton. JOURNAL OF MUSCULOSKELETAL & NEURONAL INTERACTIONS. 2013;13(4):412-7.
1476. Segal NA, Glass NA, Shakoor N, Wallace R. Vibration platform training in women at risk for symptomatic knee osteoarthritis. PM & R: Journal of Injury, Function & Rehabilitation. 2013;5(3):201-9.
1477. Nct. Vibration Therapy as a Rehabilitation Intervention for Postural Training and Fall Prevention. https://clinicaltrialsgov/show/NCT03380884. 2017.
1478. Schyns F, Paul L, Finlay K, Ferguson C, Noble E. Vibration therapy in multiple sclerosis: a pilot study exploring its effects on tone, muscle force, sensation and functional performance. Clinical rehabilitation. 2009;23(9):771‐81.
1479. Beck BR. Vibration Therapy to Prevent Bone Loss and Falls: Mechanisms and Efficacy. CURRENT OSTEOPOROSIS REPORTS. 2015;13(6):381-9.
1480. Thompson WR, Yen SS, Rubin J. Vibration therapy: clinical applications in bone. CURRENT OPINION IN ENDOCRINOLOGY DIABETES AND OBESITY. 2014;21(6):447-53.
1481. Montoro MVP, Montilla JAP, Aguilera EL, Checa MA. THE VIBRATION TRAINING AS SARCOPENIA INTERVENTION: IMPACT ON THE NEUROMUSCULAR SYSTEM OF THE ELDERLY. NUTRICION HOSPITALARIA. 2015;32(4):1454-61.
1482. Cloak R, Nevill AM, Clarke F, Day S, Wyon MA. Vibration Training Improves Balance in Unstable Ankles. International Journal of Sports Medicine. 2010;31(12):894-900.
1483. Yang F, Estrada EF, Sanchez MC. Vibration training improves disability status in multiple sclerosis: A pretest-posttest pilot study. Journal of the Neurological Sciences. 2016;369:96-101.
1484. Huber G. Vibration training into sport therapy. BEWEGUNGSTHERAPIE UND GESUNDHEITSSPORT. 2006;22(2):46-51.
1485. Yang F, Su X, Sanchez MC, Hackney ME, Butler AJ. Vibration training reducing falls in community-living older adults: a pilot randomized controlled trial. Aging Clinical and Experimental Research. 2023;35(4):803-14.
1486. Jordan MJ, Norris SR, Smith DJ, Herzog W. Vibration training: an overview of the area, training consequences, and future considerations. J Strength Cond Res. 2005;19(2):459-66.
1487. Mester J, Kleinöder H, Yue Z. Vibration training: Benefits and risks. Journal of Biomechanics. 2006;39(6):1056-65.
1488. Ruck J, Chabot G, Rauch F. Vibration treatment in cerebral palsy: a randomized controlled pilot study. Journal of musculoskeletal & neuronal interactions. 2010;10(1):77‐83.
1489. b4bzq RBR. Vibrations to treat rheumatism. https://trialsearchwhoint/Trial2aspx?TrialID=RBR-2b4bzq. 2017.
1490. Rodrigues MP, Paiva LL, Ramos JGL, Ferla L. Vibratory perineal stimulation for the treatment of female stress urinary incontinence: a systematic review. INTERNATIONAL UROGYNECOLOGY JOURNAL. 2018;29(4):555-62.
1491. Di Rienzo M, Vaini E, Castiglioni P, Merati G, Meriggi P, Parati G, et al. Wearable seismocardiography: Towards a beat-by-beat assessment of cardiac mechanics in ambulant subjects. Autonomic Neuroscience: Basic and Clinical. 2013;178(1-2):50-9.
1492. Himmelstein MS, Puhl RM, Watson RJ. Weight-based victimization, eating behaviors, and weight-related health in Sexual and Gender Minority Adolescents. Appetite. 2019;141:104321.
1493. Puhl RM, Peterson JL, Luedicke J. Weight-based victimization: bullying experiences of weight loss treatment-seeking youth. Pediatrics. 2013;131(1):e1-9.
1494. Schlacht TZ, Haque I, Skelton DA. What are the Effects of Exercise on Trabecular Microarchitecture in Older Adults? A Systematic Review and Meta-analysis of HR-pQCT Studies. Calcified Tissue International. 2023;113(4):359-82.
1495. Raschilas F, Blain H. What can we think about whole-body-vibration in elderly people? Presse Medicale. 2010;39(10):1032-7.
1496. Gloeckl R, Jarosch I, Bengsch U, Claus M, Schneeberger T, Andrianopoulos V, et al. What's the secret behind the benefits of whole-body vibration training in patients with COPD? A randomized, controlled trial. Respiratory medicine. 2017;126:17‐24.
1497. Booth S, Chohan S, Curran JC, Karrison T, Schmitz A, Utset TO. Whole blood viscosity and arterial thrombotic events in patients with systemic lupus erythematosus. Arthritis Rheum. 2007;57(5):845-50.
1498. Sandhagen B, Lind L. Whole blood viscosity and erythrocyte deformability are related to endothelium-dependent vasodilation and coronary risk in the elderly. The prospective investigation of the vasculature in Uppsala seniors (PIVUS) study. Clin Hemorheol Microcirc. 2012;50(4):301-11.
1499. Savov Y, Antonova N, Zvetkova E, Gluhcheva Y, Ivanov I, Sainova I. Whole blood viscosity and erythrocyte hematometric indices in chronic heroin addicts. Clin Hemorheol Microcirc. 2006;35(1-2):129-33.
1500. Pamukoff DN, Pietrosimone B, Lewek MD, Ryan ED, Weinhold PS, Lee DR, et al. Whole body and local muscle vibration immediately improves quadriceps function in individuals with anterior cruciate ligament reconstruction. Archives of Physical Medicine and Rehabilitation 2016 Jul;97(7):1121-1129. 2016.
1501. Blackburn JT, Pamukoff DN, Sakr M, Vaughan AJ, Berkoff DJ. Whole body and local muscle vibration reduce artificially induced quadriceps arthrogenic inhibition. Archives of physical medicine and rehabilitation. 2014;95(11):2021‐8.
1502. Wilms B, Frick J, Ernst B, Mueller R, Wirth B, Schultes B. Whole Body Vibration Added to Endurance Training in Obese Women - A Pilot Study. INTERNATIONAL JOURNAL OF SPORTS MEDICINE. 2012;33(9):740-3.
1503. Wunram HL, Hamacher S, Hellmich M, Volk M, Janicke F, Reinhard F, et al. Whole body vibration added to treatment as usual is effective in adolescents with depression: a partly randomized, three-armed clinical trial in inpatients. European Child & Adolescent Psychiatry 2018 May;27(5):645-662. 2018.
1504. Anderson AA, Goutsis DK, Hall TL, Crawford MA, Hoover DL. Whole body vibration affects gross motor performance measures during the Wingate anaerobic test. Physiotherapy (United Kingdom). 2015;101:eS76-eS7.
1505. Halsberghe BT, Gordon-Ross P, Peterson R. Whole body vibration affects the cross-sectional area and symmetry of the <i>m.multifidus</i> of the thoracolumbar spine in the horse. EQUINE VETERINARY EDUCATION. 2017;29(9):493-9.
1506. Eloá MM, Carla FD, Danielle SM, Danubia CS, Cintia RS, Laisa LP, et al. Whole body vibration and auriculotherapy improve handgrip strength in individuals with knee osteoarthritis. J Tradit Chin Med. 2019;39(5):707-15.
1507. Duquette SA, Guiliano AM, Starmer DJ. Whole body vibration and cerebral palsy: a systematic review. Journal of the Canadian Chiropractic Association 2015 Sep;59(3):245-252. 2015.
1508. Dallas G, Colson SS, Pappas P, Dallas C, Paradisis G. Whole body vibration and drop jumps induce post-activation performance enhancement. Human Movement. 2023;24(2):78-84.
1509. Hopkins T, Pak JO, Robertshaw AE, Feland JB, Hunter I, Gage M. Whole body vibration and dynamic restraint. International Journal of Sports Medicine. 2008;29(5):424-8.
1510. Nct. Whole Body Vibration and External Load Exercise Training on Cardiovascular and Autonomic Function in Obese Individuals. https://clinicaltrialsgov/show/NCT02679898. 2016.
1511. Naghii MR, Hedayati M. Whole body vibration as a safe exercise training method induces no impaired alterations on rat plasma antioxidant biomarkers. ACTA PHYSIOLOGICA HUNGARICA. 2013;100(3):321-8.
1512. Feland JB, Hawks M, Hopkins JT, Hunter I, Johnson AW, Eggett DL. Whole body vibration as an adjunct to static stretching. International Journal of Sports Medicine 2010 Aug;31(8):584-589. 2010.
1513. Minematsu A, Nishii Y, Imagita H, Sakata S. Whole body vibration at low-frequency can increase trabecular thickness and width in adult rats. JOURNAL OF MUSCULOSKELETAL & NEURONAL INTERACTIONS. 2019;19(2):169-77.
1514. Hopkins JT, Fredericks D, Guyon PW, Parker S, Gage M, Feland JB, et al. Whole body vibration does not potentiate the stretch reflex. International Journal of Sports Medicine. 2009;30(2):124-9.
1515. Stuermer EK, Komrakova M, Sehmisch S, Tezval M, Dullin C, Schaefer N, et al. Whole body vibration during fracture healing intensifies the effects of estradiol and raloxifene in estrogen-deficient rats. BONE. 2014;64:187-94.
1516. Song GE, Kim K, Lee DJ, Joo NS. Whole body vibration effects on body composition in the postmenopausal Korean obese women: Pilot study. Korean Journal of Family Medicine. 2011;32(7):399-405.
1517. Wang X-Q, Pi Y-L, Chen P-J, Chen B-L, Liang L-C, Li X, et al. Whole body vibration exercise for chronic low back pain: study protocol for a single-blind randomized controlled trial. Trials. 2014;15(1):104-.
1518. Dong YL, Wang W, Zheng JJ, Chen S, Qiao J, Wang XQ. Whole Body Vibration Exercise for Chronic Musculoskeletal Pain: A Systematic Review and Meta-analysis of Randomized Controlled Trials. ARCHIVES OF PHYSICAL MEDICINE AND REHABILITATION. 2019;100(11):2167-78.
1519. Iwamoto J, Sato Y, Takeda T, Matsumoto H. Whole body vibration exercise improves body balance and walking velocity in postmenopausal osteoporotic women treated with alendronate: Galileo and Alendronate Intervention Trail (GAIT). Journal of Musculoskeletal & Neuronal Interactions 2012 Sep;12(3):136-143. 2012.
1520. Lopes-Souza P, Dionello CF, Sá-Caputo DDC, Moreira-Marconi E, Frederico E, Marchon RM, et al. Whole body vibration exercise in the management of cancer therapy-related morbidities: A systematic review. Drug Discov Ther. 2018;12(4):239-47.
1521. Berschin G, Sommer B, Behrens A, Sommer H-M. Whole Body Vibration Exercise Protocol versus a Standard Exercise Protocol after ACL Reconstruction: A Clinical Randomized Controlled Trial with Short Term Follow-Up. Journal of Sports Science & Medicine. 2014;13(3):580-9.
1522. Bidonde J, Busch AJ, van der Spuy I, Tupper S, Kim SY, Boden C. Whole body vibration exercise training for fibromyalgia. Cochrane Database of Systematic Reviews. 2017;2017(9).
1523. Dolny DG, Reyes GFC. Whole Body Vibration Exercise: Training and Benefits. CURRENT SPORTS MEDICINE REPORTS. 2008;7(3):152-7.
1524. Da Cunha Sá-Caputo D, Ronikeili-Costa P, Carvalho-Lima RP, Bernardo LC, Bravo-Monteiro MO, Costa R, et al. Whole body vibration exercises and the improvement of the flexibility in patient with metabolic syndrome. Rehabilitation Research and Practice. 2014;2014.
1525. Baker MK, Peddle-McIntyre CJ, Galvão DA, Hunt C, Spry N, Newton RU. Whole Body Vibration Exposure on Markers of Bone Turnover, Body Composition, and Physical Functioning in Breast Cancer Patients Receiving Aromatase Inhibitor Therapy: a Randomized Controlled Trial. Integrative cancer therapies. 2018;17(3):968‐78.
1526. Rieder F, Wiesinger HP, Herfert J, Lampl K, Hecht S, Niebauer J, et al. Whole body vibration for chronic patellar tendinopathy: a randomized equivalence trial. Frontiers in physiology. 2022;13.
1527. Abbasi M, Kordi Yoosefinejad A, Poursadeghfard M, Parsaei Jahromi F, Motealleh A, Sobhani S. Whole body vibration improves core muscle strength and endurance in ambulant individuals with multiple sclerosis: a randomized clinical trial. Multiple sclerosis and related disorders. 2019;32:88‐93.
1528. Barros CESR, Fuzari HKB, Dornelas De Andrade A, Medeiros AIC, Leite JC, Lima AMS, et al. Whole body vibration improves distance walked and muscle strength in patients with chronic kidney disease: A randomized controlled trial. American Journal of Respiratory and Critical Care Medicine. 2017;195.
1529. Fuzari HK, Dornelas de Andrade A, A Rodrigues M, I Medeiros A, F Pessoa M, Lima AM, et al. Whole body vibration improves maximum voluntary isometric contraction of knee extensors in patients with chronic kidney disease: A randomized controlled trial. Physiotherapy Theory & Practice. 2019;35(5):409-18.
1530. Oroszi T, Geerts E, de Boer SF, Schoemaker RG, van der Zee EA, Nyakas C. Whole Body Vibration Improves Spatial Memory, Anxiety-Like Behavior, and Motor Performance in Aged Male and Female Rats. Frontiers in Aging Neuroscience. 2022;14:1-12.
1531. Kessler NJ, Lockard MM, Fischer J. Whole body vibration improves symptoms of diabetic peripheral neuropathy. Journal of Bodywork and Movement Therapies 2020 Apr;24(2):1-3. 2020.
1532. Adsuar JC, del Pozo-Cruz B, Parraca JA, Olivares PR, Gusi N. Whole body vibration improves the single-leg stance static balance in women with fibromyalgia: a randomized controlled trial. The Journal of Sports Medicine and Physical Fitness 2012 Feb;52(1):85-91. 2012.
1533. Cristino de Souza AL, Mendonca VA, Coelho de Oliveira AC, Ferreira da Fonseca S, Mello Santos LM, Cunha Fernandes JS, et al. Whole body vibration in the static modified push-up position in untrained healthy women stimulates neuromuscular system potentiating increased handgrip myogenic response. Journal of bodywork and movement therapies. 2020;24(4):233‐8.
1534. Moreira-Marconi E, Dionelo CF, Sá-Caputo DC, Paineiras-Domingos LL, Souza-Gonçalves CR, Bernardo-Filho M. Whole body vibration increase functionality in individuals with knee osteoartrhitis. Osteoporosis International. 2018;29(1):S523-S4.
1535. Sandhu E, Miles JD, Dahners LE, Keller BV, Weinhold PS. Whole body vibration increases area and stiffness of the flexor carpi ulnaris tendon in the rat. JOURNAL OF BIOMECHANICS. 2011;44(6):1189-91.
1536. Tubic B, Zejlon R, Wennergren G, Obermayer-Pietsch B, Marild S, Dahlgren J, et al. Whole body vibration intervention: a randomized, prospective, controlled study in children with obesity. Journal of bone and mineral research. 2017;32:S215‐.
1537. Naghii MR, Ghanizadeh G, Darvishi P, Ebrahimpour Y, Mofid M, Torkaman G, et al. Whole body vibration is a safe exercise training method and induces no impaired alterations on rat plasma parameters. ACTA PHYSIOLOGICA HUNGARICA. 2011;98(4):442-8.
1538. Hwang KJ, Ryu YU. Whole body vibration may have immediate adverse effects on the postural sway of stroke patients. Journal of Physical Therapy Science 2016 Jan;28(2):473-477. 2016.
1539. Da Silva CP. Whole Body Vibration Methods with Survivors of Polio. Journal of visualized experiments : JoVE. 2018(140).
1540. Fuzari HKB, de Andrade AD, Cerqueira MS, Pereira R, Medeiros AIC, Leite JC, et al. Whole body vibration of attenuate reduction of explosive force in chronic kidney disease patients: a randomized controlled trial. JOURNAL OF EXERCISE REHABILITATION. 2018;14(5):883-90.
1541. Da Silva CP, Szot CL, deSa N. Whole body vibration on people with sequelae of polio. Physiotherapy Theory & Practice. 2019;35(6):554-64.
1542. da Costa KSA, Borges DT, de Brito Macedo L, de Almeida Lins CA, Brasileiro JS. Whole body vibration on performance of quadriceps after ACL reconstruction: a blinded randomized controlled trial. Journal of Sport Rehabilitation 2019 Jan;28(1):52-58. 2019.
1543. Bulhões LCC, Vieira ER, Borges DT, Melo SA, Cavalcanti RL, da Costa KSA, et al. Whole Body Vibration on the Neuromuscular Performance of Elderls: Randomized Controlled Trial. Physical and Occupational Therapy in Geriatrics. 2023;41(1):75-88.
1544. Nct, Duckett JRA, Balachandran AA. Whole Body Vibration Plate Therapy and Pelvic Floor Muscle Strength in Females With Stress Urinary Incontinence. Http://clinicaltrialsgov/show/nct02319096. 2014.
1545. Jamal A, Ahmad I, Ahamed N, Azharuddin M, Alam F, Hussain ME. Whole body vibration showed beneficial effect on pain, balance measures and quality of life in painful diabetic peripheral neuropathy: a randomized controlled trial. Journal of Diabetes and Metabolic Disorders 2020 Jun;19(1):61-69. 2020.
1546. Raval AP, Schatz M, Bhattacharya P, d'Adesky N, Rundek T, Dietrich WD, et al. Whole Body Vibration Therapy after Ischemia Reduces Brain Damage in Reproductively Senescent Female Rats. INTERNATIONAL JOURNAL OF MOLECULAR SCIENCES. 2018;19(9).
1547. Christen P, Marano G, Wayne Lee YW, Lam TP, Müller R. Whole body vibration therapy triggers load-driven bone formation in adolescents with idiopathic scoliosis. Journal of Bone and Mineral Research. 2017;32:S187.
1548. Nct. Whole Body Vibration Training and Breast Cancer Risk Factors. https://clinicaltrialsgov/show/NCT04708093. 2021.
1549. Nct. Whole Body Vibration Training Applied With Different Frequencies in Hypertensive Patients. https://clinicaltrialsgov/show/NCT05768555. 2023.
1550. Bertz H, Wehrle A, Kneis S, Gollhofer A, Finke J, Pahl A. Whole body vibration training during allogeneic hematopoietic cell transplantation-the effects on patients' physical capacity, fatigue, body compsition and quality of life. Bone marrow transplantation. 2020;55:619‐.
1551. Pahl A, Wehrle A, Kneis S, Gollhofer A, Bertz H. Whole body vibration training during allogeneic hematopoietic cell transplantationâ€”the effects on patientsâ€™ physical capacity. Annals of hematology. 2020;99(3):635‐48.
1552. Gojanovic B, Feihl F, Liaudet L, Gremion G, Waeber B. Whole body vibration training elevates creatine kinase levels in sedentary subjects. SWISS MEDICAL WEEKLY. 2011;141.
1553. Middleton A, Selvadurai H, Christodoulou J, Munns C. Whole body vibration training for children with cystic fibrosis. Journal of Cystic Fibrosis. 2012;11:S105.
1554. Nct. Whole Body Vibration Training for Pelvic Floor Muscle. https://clinicaltrialsgov/show/NCT02341976. 2014.
1555. Sanudo B, Alfonso-Rosa R, del Pozo-Cruz B, del Pozo-Cruz J, Galiano D, Figueroa A. Whole body vibration training improves leg blood flow and adiposity in patients with type 2 diabetes mellitus. European Journal of Applied Physiology 2013 Sep;113(9):2245-2252. 2013.
1556. Guo C, Mi X, Liu S, Yi W, Gong C, Zhu L, et al. Whole body vibration training improves walking performance of stroke patients with knee hyperextension: a randomized controlled pilot study. CNS & Neurological Disorders Drug Targets 2015;14(9):1110-1115. 2015.
1557. Gloeckl R, Jarosch I, Seeberg S, Damisch T, Kenn K. Whole body vibration training in long-term (>1 year) lung transplant patients-A randomized, controlled trial. European respiratory journal. 2017;50.
1558. Gloeckl R, Heinzelmann I, Kenn K. Whole body vibration training in patients with COPD: a systematic review. Chronic Respiratory Disease 2015 Aug;12(3):212-221. 2015.
1559. Nct. Whole Body Vibration Training in the Treatment for Children's Incontinence - a Randomized-Controlled Trial. https://clinicaltrialsgov/show/NCT04737447. 2021.
1560. Neves CDC, Lacerda ACR, Lage VKS, Soares AA, Chaves MGA, Lima LP, et al. Whole body vibration training increases physical measures and quality of life without altering inflammatory-oxidative biomarkers in patients with moderate COPD. Journal of applied physiology (Bethesda, Md : 1985). 2018;125(2):520-8.
1561. Ligouri GC, Shoepe TC, Almstedt HC. Whole Body Vibration Training is Osteogenic at the Spine in College-Age Men and Women. JOURNAL OF HUMAN KINETICS. 2012;31:55-68.
1562. Nct. Whole Body Vibration Training on Body Composition. https://clinicaltrialsgov/show/NCT02571322. 2015.
1563. Simao AP, Mendonca VA, Avelar NCP, Fonseca SFD, Santos JM, Oliveira ACC, et al. Whole body vibration training on muscle strength and brain-derived neurotrophic factor levels in elderly woman with knee osteoarthritis: A randomized clinical trial study. Frontiers in Physiology. 2019;10(JUN).
1564. Ritzmann R, Kramer A, Bernhardt S, Gollhofer A. Whole body vibration training--improving balance control and muscle endurance. PLoS One. 2014;9(2):e89905.
1565. Fratini A, Bonci T, Bull AMJ. Whole body vibration treatments in postmenopausal women can improve bone mineral density: results of a stimulus focussed meta-analysis. PLoS ONE 2016 Dec;11(12):e0166774. 2016.
1566. Ebersbach G, Edler D, Kaufhold O, Wissel J. Whole body vibration versus conventional physiotherapy to improve balance and gait in Parkinson's disease. Archives of Physical Medicine and Rehabilitation 2008 Mar;89(3):399-403. 2008.
1567. Shanb AA, Youssef EF, Muaidi QI, Alothman AA. Whole body vibration versus magnetic therapy on bone mineral density in elderly osteoporotic individuals. Journal of Back and Musculoskeletal Rehabilitation 2017;30(4):903-912. 2017.
1568. Shehata MA, Maged AM, Kotb A, Ogila AI, Lasheen Y, Salah N, et al. Whole body vibration versus supervised aerobic exercise on hormonal parameters and inflammatry status in females with premenstrual syndrome: a randomized controlled trial. International Journal of Gynaecology and Obstetrics 2023 Aug;162(2):493-501. 2023.
1569. von Stengel S, Kemmler W, Engelke K. Whole Body Vibration: a new concept in the prevention of osteoporosis? OSTEOLOGIE. 2008;17(1):24-30.
1570. Rietschel E, van Koningsbruggen S, Fricke O, Semler O, Schoenau E. Whole body vibration: a new therapeutic approach to improve muscle function in cystic fibrosis? International Journal of Rehabilitation Research. 2008;31(3):253-6.
1571. Emerenziani GP, Meucci M, Gallotta MC, Buzzachera CF, Guidetti L, Baldari C. Whole body vibration: unsupervised training or combined with a supervised multi-purpose exercise for fitness? Journal of sports sciences. 2014;32(11):1033‐41.
1572. Nct. Whole Body Vibrations on Functional Capacity, Muscular Strength, and Biochemical Profile in Elders. https://clinicaltrialsgov/show/NCT03030456. 2016.
1573. Johnell O, Eisman J. Whole lotta shakin' goin' on. Journal of Bone and Mineral Research. 2004;19(8):1205-7.
1574. Canaud B, Rodriguez A, Chenine L, Morena M, Jaussent I, Leray-Moragues H, et al. Whole-blood viscosity increases significantly in small arteries and capillaries in hemodiafiltration. Does acute hemorheological change trigger cardiovascular risk events in hemodialysis patient? Hemodialysis International. 2010;14(4):433-40.
1575. Pamukoff DN, Pietrosimone B, Lewek MD, Ryan ED, Weinhold PS, Lee DR, et al. Whole-Body and Local Muscle Vibration Immediately Improve Quadriceps Function in Individuals With Anterior Cruciate Ligament Reconstruction. Archives of Physical Medicine & Rehabilitation. 2016;97(7):1121-9.
1576. Li L, Lamis F, Wilson SE. Whole-body vibration alters proprioception in the trunk. International Journal of Industrial Ergonomics. 2008;38(9-10):792-800.
1577. Games KE, Sefton JM, Wilson AE. Whole-Body Vibration and Blood Flow and Muscle Oxygenation: A Meta-Analysis. JOURNAL OF ATHLETIC TRAINING. 2015;50(5):542-9.
1578. Chanou K, Gerodimos V, Karatrantou K, Jamurtas A. Whole-body vibration and rehabilitation of chronic diseases: a review of the literature. Journal of Sports Science & Medicine 2012 Jun;11(2):187-200. 2012.
1579. Li ZL, Tan C, Wu YH, Ding Y, Wang HJ, Chen WJ, et al. Whole-body vibration and resistance exercise prevent long-term hindlimb unloading-induced bone loss: independent and interactive effects. EUROPEAN JOURNAL OF APPLIED PHYSIOLOGY. 2012;112(11):3743-53.
1580. Feland JB, Thalman L, Hunter I, Cochrane DJ, Hopkins FT. Whole-body vibration and stretching enhances dorsiflexion range of motion in individuals with chronic ankle instability Physical Therapy in Sport 2020 Jul;44:1-7. 2020.
1581. Aminian-Far A, Hadian MR, Olyaei G, Talebian S, Bakhtiary AH. Whole-body vibration and the prevention and treatment of delayed-onset muscle soreness. Journal of athletic training. 2011;46(1):43‐9.
1582. Marín PJ, Herrero AJ, Milton JG, Hazell TJ, García-López D. Whole-body vibration applied during upper body exercise improves performance. Journal of Strength and Conditioning Research. 2013;27(7):1807-12.
1583. Verhulst ALJ, Savelberg H, Vreugdenhil G, Mischi M, Schep G. Whole-body vibration as a modality for the rehabilitation of peripheral neuropathies: implications for cancer survivors suffering from chemotherapy-induced peripheral neuropathy. ONCOLOGY REVIEWS. 2015;9(1).
1584. Tóth K, Oroszi T, Nyakas C, van der Zee EA, Schoemaker RG. Whole-body vibration as a passive alternative to exercise after myocardial damage in middle-aged female rats: Effects on the heart, the brain, and behavior. Frontiers in Aging Neuroscience. 2023;15.
1585. Figueroa A, Jaime SJ, Alvarez-Alvarado S. Whole-body vibration as a potential countermeasure for dynapenia and arterial stiffness. INTEGRATIVE MEDICINE RESEARCH. 2016;5(3):204-11.
1586. Magoffin RD, Parcell AC, Hyldahl RD, Fellingham GW, Hopkins JT, Feland JB. Whole-Body Vibration as a Warm-up Before Exercise-Induced Muscle Damage on Symptoms of Delayed-Onset Muscle Soreness in Trained Subjects. Journal of strength and conditioning research. 2020;34(4):1123-32.
1587. Totosy de Zepetnek JO, Giangregorio LM, Craven BC. Whole-body vibration as potential intervention for people with low bone mineral density and osteoporosis: A review. JOURNAL OF REHABILITATION RESEARCH AND DEVELOPMENT. 2009;46(4):529-42.
1588. Rodrigues FTM, Ferreira APD, Alves KFP, Marques TV, de Lima DF, de Lucena LC, et al. Whole-Body Vibration Associated with Strength Training on the Lower-Limb Blood Flow and Mobility in Older Adults with Type 2 Diabetes: A Study Protocol for a Randomized Controlled Trial. DIAGNOSTICS. 2022;12(7).
1589. Fjeldstad C, Palmer IJ, Bemben MG, Bemben DA. Whole-body vibration augments resistance training effects on body composition in postmenopausal women. Maturitas. 2009;63(1):79‐83.
1590. Okechukwu CE. Whole-body vibration combined with exercise may be more effective in lowering blood pressure and arterial stiffness than exercise alone in older adults with hypertension. MEDICAL HYPOTHESES. 2021;157.
1591. Lippi L, De Sire A, Ammendolia A, Cisari C, Venetis K, Sajjadi E, et al. Whole-body vibration combined with physical exercise to treat aromatase inhibitor-induced musculoskeletal symptoms in breast cancer women: results of a pilot randomized controlled study. Cancer research. 2022;82(4 SUPPL).
1592. Choi W, Han D, Kim J, Lee S. Whole-body vibration combined with treadmill training improves walking performance in post-stroke patients: a randomized controlled trial. Medical Science Monitor 2017 Oct 14;23:4918-4925. 2017.
1593. Akehurst H, Grice JE, Angioi M, Morrissey D, Migliorini F, Maffulli N. Whole-body vibration decreases delayed onset muscle soreness following eccentric exercise in elite hockey players: a randomised controlled trial. Journal of Orthopaedic Surgery and Research 2021 Oct 12;16(589):Epub. 2021.
1594. Tossige-Gomes R, Avelar NCP, Simão AP, Neves CDC, Brito-Melo GEA, Coimbra CC, et al. Whole-body vibration decreases the proliferative response of TCD4+ cells in elderly individuals with knee osteoarthritis. Brazilian Journal of Medical and Biological Research. 2012;45(12):1262-8.
1595. Lythgo N, Eser P, de Groot P, Galea M. Whole-body vibration dosage alters leg blood flow. CLINICAL PHYSIOLOGY AND FUNCTIONAL IMAGING. 2009;29(1):53-9.
1596. Yang P, Jia B, Ding C, Wang Z, Qian AR, Shang P. Whole-Body Vibration Effects on Bone Before and After Hind-Limb Unloading in Rats. AVIATION SPACE AND ENVIRONMENTAL MEDICINE. 2009;80(2):88-93.
1597. Humphries B, Fenning A, Dugan E, Guinane J, MacRae K. Whole-Body Vibration Effects on Bone Mineral Density in Women With or Without Resistance Training. AVIATION SPACE AND ENVIRONMENTAL MEDICINE. 2009;80(12):1025-31.
1598. Đorđević D, Paunović M, Čular D, Vlahović T, Franić M, Sajković D, et al. Whole-Body Vibration Effects on Flexibility in Artistic Gymnastics-A Systematic Review. Medicina (Kaunas, Lithuania). 2022;58(5).
1599. Mikami Y, Amano J, Kawamura M, Nobiro M, Kamijyo Y, Kawae T, et al. Whole-body vibration enhances effectiveness of "locomotion training" evaluated in healthy young adult women. J Phys Ther Sci. 2019;31(11):895-900.
1600. Li X, Wang XQ, Chen BL, Huang LY, Liu Y. Whole-body vibration exercise for knee osteoarthritis: a systematic review and meta-analysis. Evidence-Based Complementary and Alternative Medicine 2015;(758147):Epub. 2015.
1601. Sá-Caputo DC, Dionello CF, Frederico ÉHFF, Paineiras-Domingos LL, Sousa-Gonçalves CR, Morel DS, et al. Whole-body vibration exercise improves functional parameters in patients with Osteogenesis Imperfecta: A systematic review with a suitable approach. African Journal of Traditional, Complementary and Alternative Medicines. 2017;14(3):199-208.
1602. De Aguiar EDG, Marconi EM, Monteiro-Oliveira BB, Gomes-Santos AC, Oliveira ACC, Paineiras-Domingos LL, et al. Whole-Body Vibration Exercise Improves the Functionality in Postmenopausal Women: A Systematic Review. IRANIAN JOURNAL OF PUBLIC HEALTH. 2023;52(3):476-87.
1603. Santos LMM, Oliveira ACC, Fonseca SF, Silva AF, Santos JNV, Souza ALC, et al. Whole-Body Vibration Exercise in Different Postures on Handgrip Strength in Healthy Women: A Cross-Over Study. Frontiers in Physiology. 2021;11.
1604. Weber-Rajek M, Mieszkowski J, Niespodziński B, Ciechanowska K. Whole-body vibration exercise in postmenopausal osteoporosis. Przeglad Menopauzalny. 2015;14(1):41-7.
1605. Kerschan-Schindl K, Grampp S, Henk C, Resch H, Preisinger E, Fialka-Moser V, et al. Whole-body vibration exercise leads to alterations in muscle blood volume. Clinical Physiology. 2001;21(3):377-82.
1606. Wong A, Alvarez-Alvarado S, Kinsey AW, Figueroa A. Whole-Body Vibration Exercise Therapy Improves Cardiac Autonomic Function and Blood Pressure in Obese Pre- and Stage 1 Hypertensive Postmenopausal Women. Journal of Alternative & Complementary Medicine. 2016;22(12):970-6.
1607. Figueroa A, Kalfon R, Madzima TA, Wong A. Whole-body vibration exercise training reduces arterial stiffness in postmenopausal women with prehypertension and hypertension. Menopause (10723714). 2014;21(2):131-6.
1608. Sa-Caputo DC, Coelho-Oliveira AC, Pessanha-Freitas J, Paineiras-Domingos LL, Lacerda ACR, Mendonça VA, et al. Whole-Body Vibration Exercise: A Possible Intervention in the Management of Post COVID-19 Complications? APPLIED SCIENCES-BASEL. 2021;11(12).
1609. Seixas A, Sanudo B, Sa-Caputo D, Taiar R, Bernardo-Filho M. Whole-body vibration for individuals with reconstructed anterior cruciate ligament: a systematic review. BioMed Research International 2020;(7362069):Epub. 2020.
1610. Oh S, Oshida N, Someya N, Maruyama T, Isobe T, Okamoto Y, et al. Whole-body vibration for patients with nonalcoholic fatty liver disease: a 6-month prospective study. Physiological reports. 2019;7(9):e14062.
1611. Lau RW, Yip SP, Pang MY. Whole-body vibration has no effect on neuromotor function and falls in chronic stroke. Medicine and science in sports and exercise. 2012;44(8):1409‐18.
1612. Abd-Eltawab AE, Elbandrawy AM, Ghanem HB, Ebrahim HA, El-Sherbiny M, Ibrahim AM, et al. Whole-Body Vibration Impacts on the Degree of Toe Angle and Its Correlation to the Knee Osteoarthritis Index during Level Walking among Female University Students: A Randomized Controlled Trial. Journal of Clinical Medicine. 2023;12(17).
1613. In T, Jung K, Lee MG, Cho HY. Whole-body vibration improves ankle spasticity, balance, and walking ability in individuals with incomplete cervical spinal cord injury. Neurorehabilitation 2018;42(4):491-497. 2018.
1614. Pamukoff DN, Pietrosimone B, Ryan ED, Lee DR, Brown LE, Blackburn JT. Whole-Body Vibration Improves Early Rate of Torque Development in Individuals With Anterior Cruciate Ligament Reconstruction. Journal of strength and conditioning research. 2017;31(11):2992‐3000.
1615. Butezloff MM, Zamarioli A, Leoni GB, Sousa-Neto MD, Volpon JB. Whole-body vibration improves fracture healing and bone quality in rats with ovariectomy-induced osteoporosis. ACTA CIRURGICA BRASILEIRA. 2015;30(11):727-35.
1616. Braz DS, de Andrade AD, Teixeira AS, Cavalcanti CA, Morais AB, Marinho PEM. Whole-body vibration improves functional capacity and quality of life in patients with severe chronic obstructive pulmonary disease (COPD): A pilot study. International Journal of COPD. 2015;10:125-32.
1617. Dutra MC, de Oliveira ML, Marin RV, Kleine HCR, Silva OL, Lazaretti-Castro M. Whole-body vibration improves neuromuscular parameters and functional capacity in osteopenic postmenopausal women. Menopause (10723714). 2016;23(8):870-5.
1618. Rodriguez-Miguelez P, Fernandez-Gonzalo R, Collado PS, Almar M, Martinez-Florez S, de Paz JA, et al. Whole-body vibration improves the anti-inflammatory status in elderly subjects through toll-like receptor 2 and 4 signaling pathways. Mechanisms of Ageing and Development 2015 Sep;150:12-19. 2015.
1619. Sá-Caputo D, Paineiras-Domingos LL, Francisca-Santos A, Dos Anjos EM, Reis AS, Neves MFT, et al. Whole-body vibration improves the functional parameters of individuals with metabolic syndrome: An exploratory study. BMC Endocrine Disorders. 2019;19(1).
1620. Pollock RD, Martin FC, Newham DJ. Whole-body vibration in addition to strength and balance exercise for falls-related functional mobility of frail older adults: a single-blind randomized controlled trial. Clinical rehabilitation. 2012;26(10):915‐23.
1621. Rustler V, Daggelmann J, Streckmann F, Bloch W, Baumann FT. Whole-body vibration in children with disabilities demonstrates therapeutic potentials for pediatric cancer populations: a systematic review. Supportive Care in Cancer 2019 Feb;27(2):395-406. 2019.
1622. Lee G. Whole-body vibration in horizontal direction for stroke rehabilitation: a randomized controlled trial. Medical Science Monitor 2019 Mar 2;25:1621-1628. 2019.
1623. Bernstein JL, Reader B, Strenk ML, Mersich KM. WHOLE-BODY VIBRATION IN ONCOLOGY REHABILITATION: A CLINICIAN SURVEY. Rehabilitation Oncology. 2023;41(2):108.
1624. Cristovam DN, Botelho S, Andrade MF, Marques J, Sousa L. Whole-body vibration in the reduction of the cellulite. JOURNAL OF COSMETIC AND LASER THERAPY. 2019;21(5):278-85.
1625. Pessoa MF, Brandao DC, De Sa RB, Aguiar MIR, De Souza HCM, De Melo Barcelar J, et al. Whole-Body Vibration Increases Cardiopulmonary Performance in the Elderly: a Randomized Double-Blind Clinical Trial. Topics in geriatric rehabilitation. 2018;34(4):245‐50.
1626. Savelberg HH, Keizer HA, Meijer K. Whole-body vibration induced adaptation in knee extensors; consequences of initial strength, vibration frequency, and joint angle. Journal of strength and conditioning research. 2007;21(2):589‐93.
1627. Liao LR, Ng GY, Jones AY, Huang MZ, Pang MY. Whole-Body Vibration Intensities in Chronic Stroke: a Randomized Controlled Trial. Medicine and science in sports and exercise. 2016;48(7):1227‐38.
1628. Huang M, Miller T, Ying M, Pang MYC. Whole-body vibration modulates leg muscle reflex and blood perfusion among people with chronic stroke: a randomized controlled crossover trial. Scientific reports. 2020;10(1):1473.
1629. da Silva Morais CC, Misiak GF, Santin LM, de Carvalho AR, Bertolini GRF. Whole-body vibration on lower limb flexibility and extensibility – a randomized clinical trial. European Journal of Clinical and Experimental Medicine. 2023;21(2):224-9.
1630. Atalay OT, Yilmaz A, Bahtiyar BC, Altinisik G. Whole-body vibration or aerobic exercise in patients with bronchiectasis? A randomized controlled study. Medicina (Kaunas) 2022 Dec;58(12):1790. 2022.
1631. Reijne AC, Ciapaite J, van Dijk TH, Havinga R, van der Zee EA, Groen AK, et al. Whole-Body Vibration Partially Reverses Aging-Induced Increases in Visceral Adiposity and Hepatic Lipid Storage in Mice. PLOS ONE. 2016;11(2).
1632. Isrctn. A whole-body vibration program in type 2 diabetic patients. https://trialsearchwhoint/Trial2aspx?TrialID=ISRCTN16866781. 2013.
1633. Johnson AW, Myrer JW, Hunter I, Feland JB, Hopkins JT, Draper DO, et al. Whole-body vibration strengthening compared to traditional strengthening during physical therapy in individuals with total knee arthroplasty. Physiotherapy theory and practice. 2010;26(4):215‐25.
1634. Sañudo B, Reverte-Pagola G, Seixas A, Masud T. Whole-Body Vibration to Improve Physical Function Parameters in Nursing Home Residents Older than 80 Years: A Systematic Review with Meta-Analysis. Physical therapy. 2024.
1635. Zhou J, Pang L, Chen N, Wang Z, Wang C, Hai Y, et al. Whole-body vibration training -- better care for COPD patients: a systematic review and meta-analysis. International Journal of Chronic Obstructive Pulmonary Disease 2018 Oct 10;13:3243-3254. 2018.
1636. Marín-Cascales E, Alcaraz PE, Ramos-Campo DJ, Martinez-Rodriguez A, Chung LH, Rubio-Arias JA. Whole-body vibration training and bone health in postmenopausal women A systematic review and meta-analysis. MEDICINE. 2018;97(34).
1637. Hawkey A, Griffiths K, Babraj J, Cobley JN. Whole-Body Vibration Training and Its Application to Age-Related Performance Decrements: an Exploratory Analysis. Journal of strength and conditioning research. 2016;30(2):555‐60.
1638. Nct. Whole-Body Vibration Training and Pilates Exercises for Healthy Women. https://clinicaltrialsgov/show/NCT04490577. 2020.
1639. Kaeding TS, Karch A, Schwarz R, Flor T, Wittke TC, Kück M, et al. Whole-body vibration training as a workplace-based sports activity for employees with chronic low-back pain. SCANDINAVIAN JOURNAL OF MEDICINE & SCIENCE IN SPORTS. 2017;27(12):2027-39.
1640. Kaeding TS, Bieneck M, Tegtbur U, Kück M, Karch A, Böselt G, et al. Whole-body vibration training as a workplace-based sports activity: a randomized, controlled trial. MEDICINA DELLO SPORT. 2018;71(2):268-+.
1641. Cristi-Montero C, Cuevas MJ, Collado PS. Whole-body vibration training as complement to programs aimed at weight loss. NUTRICION HOSPITALARIA. 2013;28(5):1365-71.
1642. Ahlborg L, Andersson C, Julin P. Whole-body vibration training compared with resistance training: effect on spasticity, muscle strength and motor performance in adults with cerebral palsy. Journal of rehabilitation medicine. 2006;38(5):302‐8.
1643. Figueroa A, Kalfon R, Wong A. Whole-body vibration training decreases ankle systolic blood pressure and leg arterial stiffness in obese postmenopausal women with high blood pressure. Menopause 2015 Apr;22(4):423-427. 2015.
1644. Rustler V, Prokop A, Baumann FT, Streckmann F, Bloch W, Daeggelmann J. Whole-Body Vibration Training Designed to Improve Functional Impairments After Pediatric Inpatient Anticancer Therapy: A Pilot Study. Pediatric physical therapy : the official publication of the Section on Pediatrics of the American Physical Therapy Association. 2018;30(4):341-9.
1645. Jiang Y, Zhang S. Whole-body vibration training does not improve the static balance of older women in the eyes-open state: A randomized trial. Technology and Health Care. 2023;31(3):911-9.
1646. Amaral PC, Miranda MLJ, Rica RL, Junior Figueira A, Evangelista AL, Junior Pontes FL, et al. Whole-body vibration training does not modify anthropometric parameters and lower limb strength in elderly people. Clinical and experimental medical letters. 2014;55:6‐10.
1647. Huang CC, Tseng TL, Huang WC, Chung YH, Chuang HL, Wu JH. Whole-Body Vibration Training Effect on Physical Performance and Obesity in Mice. INTERNATIONAL JOURNAL OF MEDICAL SCIENCES. 2014;11(12):1218-27.
1648. Colson SS, Pensini M, Espinosa J, Garrandes F, Legros P. Whole-body vibration training effects on the physical performance of basketball players. Journal of strength and conditioning research. 2010;24(4):999‐1006.
1649. Zhou JM, Guo LF, Chang M, Lan ZS, Li SQ. WHOLE-BODY VIBRATION TRAINING FOR CHILDREN WITH NEUROLOGICAL DISABILITIES: A META-ANALYSIS. KINESIOLOGY. 2023;55(1):162-73.
1650. Rabert MS, Comas DR, Vanmeerhaeghe AF, Medina CS, Figuls MRI, Romero-Rodríguez D, et al. Whole-body vibration training for patients with neurodegenerative disease. COCHRANE DATABASE OF SYSTEMATIC REVIEWS. 2012(2).
1651. Ko MC, Wu LS, Lee S, Wang CC, Lee PF, Tseng CY, et al. Whole-body vibration training improves balance control and sit-to-stand performance among middle-aged and older adults: a pilot randomized controlled trial. European Review of Aging and Physical Activity 2017 Jul 18;14(11):Epub. 2017.
1652. Lee K, Lee S, Song C. Whole-body vibration training improves balance, muscle strength and glycosylated hemoglobin in elderly patients with diabetic neuropathy. Tohoku journal of experimental medicine. 2013;231(4):305‐14.
1653. Karatrantou K, Gerodimos V, Dipla K, Zafeiridis A. Whole-body vibration training improves flexibility, strength profile of knee flexors, and hamstrings-to-quadriceps strength ratio in females. Journal of Science & Medicine in Sport. 2013;16(5):477-81.
1654. Severino G, Sanchez-Gonzalez M, Walters-Edwards M, Nordvall M, Chernykh O, Adames J, et al. Whole-body vibration training improves heart rate variability and body fat percentage in obese Hispanic postmenopausal women. Journal of Aging and Physical Activity 2017 Jul;25(3):395-401. 2017.
1655. Chinoy A, Ireland A, Montgomery G, Vassallo G, Roberts S, Eelloo J, et al. Whole-body vibration training in addition to muscle-strengthening exercises alone in improving muscle function in children with Neurofibromatosis Type 1 a randomised interventional trial. JBMR Plus. 2022;6:136-7.
1656. Vry J, Schubert IJ, Semler O, Haug V, Schönau E, Kirschner J. Whole-body vibration training in children with Duchenne muscular dystrophy and spinal muscular atrophy. European Journal of Paediatric Neurology. 2014;18(2):140-9.
1657. Grubbs BF, Figueroa A, Kim JS, Contreras RJ, Schmitt K, Panton LB. Whole-body vibration training in frail, skilled nursing home residents. International Journal of Exercise Science. 2020;13(3):140-56.
1658. Gerodimos V, Zafeiridis A, Chanou K, Karatrantou K, Dipla K. Whole-body vibration training in middle-aged females: improving muscle flexibility and the power of lower limbs. Sport Sciences for Health. 2015;11(3):287-94.
1659. Zago M, Capodaglio P, Ferrario C, Tarabini M, Galli M. Whole-body vibration training in obese subjects: A systematic review. PLoS ONE. 2018;13(9).
1660. Carr K, Lachance CC, Kenno K, McNevin N, Horton S, Weir P. Whole-body vibration training in older adults: Retention of the strengthening effects. Critical Reviews in Physical and Rehabilitation Medicine. 2012;24(1-2):51-67.
1661. Machado A, García-López D, González-Gallego J, Garatachea N. Whole-body vibration training increases muscle strength and mass in older women: a randomized-controlled trial. Scandinavian journal of medicine & science in sports. 2010;20(2):200‐7.
1662. Cristi C, Collado PS, Márquez S, Garatachea N, Cuevas MJ. Whole-body vibration training increases physical fitness measures without alteration of inflammatory markers in older adults. European journal of sport science. 2014;14(6):611-9.
1663. Wyon M, Guinan D, Hawkey A. Whole-body vibration training increases vertical jump height in a dance population. Journal of strength and conditioning research. 2010;24(3):866‐70.
1664. Rieder F, Wiesinger HP, Kösters A, Müller E, Seynnes OR. Whole-body vibration training induces hypertrophy of the human patellar tendon. Scandinavian journal of medicine & science in sports. 2016;26(8):902‐10.
1665. Nct. Whole-body Vibration Training on Functional Performance of the Elderly With Knee Osteoarthritis. https://clinicaltrialsgov/show/NCT03918291. 2019.
1666. de Melo FAT, de Melo GF, Neto SLD, da Silva RW, de França NM, da Silva AA, et al. WHOLE-BODY VIBRATION TRAINING PROTOCOLS IN OBESE INDIVIDUALS: A SYSTEMATIC REVIEW. REVISTA BRASILEIRA DE MEDICINA DO ESPORTE. 2019;25(6):527-33.
1667. Figueroa A, Gil R, Wong A, Hooshmand S, Park SY, Vicil F, et al. Whole-body vibration training reduces arterial stiffness, blood pressure and sympathovagal balance in young overweight/obese women. Hypertension research. 2012;35(6):667‐72.
1668. Gloeckl R, Schneeberger T, Leitl D, Reinold T, Nell C, Jarosch I, et al. Whole-body vibration training versus conventional balance training in patients with severe COPD—a randomized, controlled trial. Respiratory Research. 2021;22(1).
1669. Gojanovic B, Gremion G, Waeber B. Whole-body vibration training: Fact or fiction? Revue Medicale Suisse. 2008;4(166):1712-6.
1670. Gojanovic B, Henchoz Y. Whole-body vibration training: Metabolic cost of synchronous, side-alternating or no vibrations. Journal of Sports Sciences. 2012;30(13):1397-403.
1671. Horstmann T, Jud HM, FrÖHlich V, MÜNdermann A, Grau S. Whole-Body Vibration Versus Eccentric Training or a Wait-and-See Approach for Chronic Achilles Tendinopathy: A Randomized Clinical Trial. Journal of Orthopaedic & Sports Physical Therapy. 2013;43(11):794-803.
1672. Stolzenberg N, Belavý DL, Rawer R, Felsenberg D. Whole-body vibration versus proprioceptive training on postural control in post-menopausal osteopenic women. Gait & posture. 2013;38(3):416‐20.
1673. Shehata MMA, Maged AM, Kotb A, Ogila AI, Lasheen Y, Salah N, et al. Whole-body vibration versus supervised aerobic exercise on hormonal parameters and inflammatory status in women with premenstrual syndrome: A randomized controlled trial. INTERNATIONAL JOURNAL OF GYNECOLOGY & OBSTETRICS. 2023;162(2):493-501.
1674. Dabbs NC, Black CD, Garner J. Whole-body vibration while squatting and delayed-onset muscle soreness in women. Journal of Athletic Training 2015 Dec;50(12):1233-1239. 2015.
1675. Sierra-Guzman R, Jimenez-Diaz F, Ramirez C, Esteban P, Abian-Vicen J. Whole-body-vibration training and balance in recreational athletes with chronic ankle instability. Journal of Athletic Training 2018 Apr;53(4):355-363. 2018.
1676. Whole-body-vibration training improves knee-extension, speed of movement. Geriatrics and Aging. 2004;7(9):11.
1677. Roelants M, Delecluse C, Verschueren SM. Whole-body-vibration training increases knee-extension strength and speed of movement in older women. Journal of the American Geriatrics Society. 2004;52(6):901-8.
1678. Van Den Tillaar R. Will whole-body vibration training help increase the range of motion of the hamstrings? Journal of Strength and Conditioning Research. 2006;20(1):192-6.
1679. Szeto GPY, Lam PG. Work-related musculoskeletal disorders in urban bus drivers of Hong Kong. JOURNAL OF OCCUPATIONAL REHABILITATION. 2007;17(2):181-98.
1680. Silverstein BA, Stetson DS, Keyserling WM, Fine LJ. Work-related musculoskeletal disorders: Comparison of data sources for surveillance. American Journal of Industrial Medicine. 1997;31(5):600-8.

Excluded study titles with specific reasons (*n* = 132).

1. Whole-body vibration training induces hypertrophy of the human patellar tendon
2. Long-Term Effects of Whole-Body Vibration in Trained Adolescent Swimmers: Does It Increase Strength, Power, and Swimming Performance?
3. WHOLE-BODY VIBRATION TRAINING EFFECTS ON THE PHYSICAL PERFORMANCE OF BASKETBALL PLAYERS
4. The short-term effect of whole-body vibration training on vertical jump, sprint, and agility performance
5. Effects of whole body vibration training on cardiorespiratory fitness and muscle strength in older individuals (a 1-year randomised controlled trial)
6. Effects of vibration training in reducing risk of slip-related falls among young adults with obesity
7. Influence of whole-body vibration training without visual feedback on balance and lower-extremity muscle strength of the elderly
8. Effect of four-month vertical whole body vibration on performance and balance
9. Effects of a whole body vibration (WBV) exercise intervention for institutionalized older people: a randomized, multicentre, parallel, clinical trial
10. Effects of whole-body vibration exercise on lower-extremity muscle strength and power in an older population: A randomized clinical trial
11. Effect of Squat Training with Whole Body Vibration on Balance and Functional Performance in Elderly-A Quasi-experimental Study
12. Immediate effect of vibratory stimuli on quadriceps function in healthy adults
13. Effects of combining whole-body vibration with exercise on the consequences of detraining on muscle performance in untrained adults
14. Improving strength and postural control in young skiers: Whole-body vibration versus equivalent resistance training
15. Whole-body vibration training improves balance control and sit-to-stand performance among middle-aged and older adults: a pilot randomized controlled trial
16. Long-Term Impact of Strength Training on Muscle Strength Characteristics in Older Adults
17. The effect of exercise load deviations in whole body vibration on improving muscle strength imbalance in the lower limb
18. Effects of a short-term whole body vibration intervention on physical fitness in elderly people
19. INFLUENCE OF WHOLE BODY VIBRATION PLATFORM FREQUENCY ON NEUROMUSCULAR PERFORMANCE OF COMMUNITY-DWELLING OLDER ADULTS
20. The effects of vibration on explosive and reactive strength when applying individualized vibration frequencies
21. The effects of 11 weeks whole body vibration training on jump height, contractile properties and activation of human knee extensors
22. Short-term effects of whole-body vibration on maximal voluntary isometric knee extensor force and rate of force rise
23. EFFECT OF DIFFERENT REST INTERVALS AFTER WHOLE-BODY VIBRATION ON VERTICAL JUMP PERFORMANCE
24. Effects of vertical and side-alternating vibration training on fall risk factors and bone turnover in older people at risk of falls
25. Effect of five weeks of whole body vibration training on speed, power, and flexibility
26. Superimposed vibration on suspended push-ups
27. Evaluation of the impact of 6-month training by whole body vibration on the risk of falls among nursing home residents, observed over a 12-month period: a single blind, randomized controlled trial
28. Effects of supervised whole body vibration exercise on fall risk factors, functional dependence and health-related quality of life in nursing home residents aged 80+
29. Vibration exposure and biodynamic responses during whole-body vibration training
30. Effects of Whole-Body Vibration Training on the Physical Function of the Frail Elderly: An Open, Randomized Controlled Trial
31. Effects of whole-body vibration training on calf muscle function during maximal isometric voluntary contractions
32. ACUTE EFFECTS OF VARIOUS WHOLE BODY VIBRATION FREQUENCIES ON 1RM IN TRAINED AND UNTRAINED SUBJECTS
33. Acute Effects of Whole-Body Vibration on Trunk and Neck Muscle Activity in Consideration of Different Vibration Loads
34. EVALUATION OF A SIX-WEEK WHOLE-BODY VIBRATION INTERVENTION ON NEUROMUSCULAR PERFORMANCE IN OLDER ADULTS
35. Effects of whole-body vibration training on sprint running kinematics and explosive strength performance
36. Effect of 4-min vertical whole body vibration on muscle performance and body balance: a randomized cross-over study
37. Effects of whole-body vibration training on bone-free lean body mass and muscle strength in young adults
38. Effects of resistance training with whole-body vibration on muscle fitness in untrained adults
39. Effects of whole-body vibration on resistance training for untrained adults
40. A comparison of training intensity between whole-body vibration and conventional squat exercise
41. Effects of vibration training and detraining on balance and muscle strength in older adults
42. Effects of whole-body vibration on balance and mobility in institutionalized older adults: a randomized controlled trial
43. The effects of whole-body vibration training on upper and lower body strength in older adults
44. Beyond physiology: Acute effects of side-alternating whole-body vibration on well-being, flexibility, balance, and cognition using a light and portable platform A randomized controlled trial
45. Effects of whole body vibration training on muscle strength and sprint performance in sprint-trained athletes
46. The acute effects of different training loads of whole body vibration on flexibility and explosive strength of lower limbs in divers
47. Evaluation of 18-Week Whole-Body Vibration Training in Normobaric Hypoxia on Lower Extremity Muscle Strength in an Elderly Population
48. Effects of whole body vibration training combined with blood flow restriction on muscle adaptation
49. Effects of whole-body vibration training on balance, muscle function, gait performance and perceived participation after stroke: A randomized controlled study
50. The feasibility of Whole Body Vibration in institutionalised elderly persons and its influence on muscle performance, balance and mobility: a randomised controlled trial
51. Effects of whole-body vibration and resistance training on knee extensors muscular performance
52. Variation in neuromuscular responses during acute whole-body vibration exercise
53. The Effectiveness of Whole-Body Vibration and Heat Therapy on the Muscle Strength, Flexibility, and Balance Abilities of Elderly Groups
54. Stochastic resonance whole-body vibration training for chair rising performance on untrained elderly: a pilot study
55. The effects of whole-body vibration on the cross-transfer of strength
56. Effect of whole body vibration training on lower limb performance in selected high-level ballet students
57. Effects of vibration exercise on muscle performance and mobility in an older population
58. The Effects of Vibration Exposure on Lower-Limb Extensor Muscles' Stiffness, Elasticity, and Strength Responses in Untrained Young Individuals: a Randomized Controlled Trial
59. Cross-training effect of chronic whole-body vibration exercise: a randomized controlled study
60. A randomized trial on the effect of bone tissue on vibration-induced muscle strength gain and vibration-induced reflex muscle activity
61. Strength training with superimposed whole body vibration does not preferentially modulate cortical plasticity
62. Benefits of Two 24-Week Interactive Cognitive-Motor Programs on Body Composition, Lower-Body Strength, and Processing Speed in Community Dwellings at Risk of Falling: a Randomized Controlled Trial
63. THE EFFECT OF WHOLE-BODY VIBRATION ON JUMP HEIGHT AND ACTIVE RANGE OF MOVEMENT IN FEMALE DANCERS
64. Comparing the effects of various whole-body vibration accelerations on countermovement jump performance
65. EFFECTS OF QUADRICEPS STRENGTH AFTER STATIC AND DYNAMIC WHOLE-BODY VIBRATION EXERCISE
66. Acute effects of whole-body vibrations on balance, maximal force and perceived exertion: Vertical platform versus oscillating platform
67. Acute effects of whole-body vibration on trunk muscle functioning in young healthy adults
68. Whole-Body-Vibration Training and Balance in Recreational Athletes With Chronic Ankle Instability
69. Circulating microRNA responses to acute whole-body vibration and resistance exercise in postmenopausal women
70. Effect of 10-Week Whole-Body Vibration Training on Falls and Physical Performance in Older Adults: A Blinded, Randomized, Controlled Clinical Trial with 1-Year Follow-Up
71. Effects of 6 weeks of whole-body vibration training on ankle motor control: a randomized controlled trial
72. Effects of Strength Training Associated With Whole-Body Vibration Training on Running Economy and Vertical Stiffness
73. Combined isometric and vibration training does not enhance strength beyond that of isometric training alone
74. A brief whole-body vibration intervention to avoid weight gain in college students: A randomized controlled pilot trial
75. Whole-body vibration and the prevention and treatment of delayed-onset muscle soreness
76. Effects of Low-Frequency Whole-Body Vibration on Muscle Activation, Fatigue, and Oxygen Consumption in Healthy Young Adults: A Single-Group Repeated-Measures Controlled Trial
77. THE EFFECTS OF WHOLE BODY VIBRATION ON VERTICAL JUMP, POWER, BALANCE, AND AGILITY IN UNTRAINED ADULTS
78. The acute effects of different whole-body vibration amplitudes and frequencies on flexibility and vertical jumping performance
79. Whole body vibration in the static modified push-up position in untrained healthy women stimulates neuromuscular system potentiating increased handgrip myogenic response
80. Immediate effects of whole-body vibration on neuromuscular performance of quadriceps and oscillation of the center of pressure: A randomized controlled trial
81. Isometric Mid-thigh Pull Kinetics: Sex Differences and Response to Whole-Body Vibration
82. Short-term effect of whole-body vibration training on balance, flexibility and lower limb explosive strength in elite rhythmic gymnasts
83. Acute whole body vibration training increases vertical jump and flexibility performance in elite female field hockey players
84. Acute effect of whole body vibration on isometric strength, squat jump, and flexibility in well-trained combat athletes
85. THE ACUTE EFFECT OF WHOLE BODY VIBRATION TRAINING ON FLEXIBILITY AND EXPLOSIVE STRENGTH OF YOUNG GYMNASTS
86. Acute Effect of Whole-Body Vibration at Optimal Frequency on Muscle Power Output of the Lower Limbs in Older Women
87. ACUTE EFFECT OF WHOLE-BODY VIBRATION ON SPRINT AND JUMPING PERFORMANCE IN ELITE SKELETON ATHLETES
88. THE ACUTE EFFECT OF WHOLE-BODY VIBRATION ON THE VERTICAL JUMP HEIGHT
89. The Acute Effects of Different Intensity Whole-Body Vibration Exposure on Muscle Tone and Strength of the Lower Legs, and Hamstring Flexibility: A Pilot Study
90. Acute effects of stochastic and sinusoidal whole body vibration on pelvic floor muscle activation
91. The acute effects of two different whole body vibration frequencies on vertical jump performance
92. Acute effects of various whole-body vibration frequencies on lower-body power in trained and untrained subjects
93. Acute Effects of Whole-Body Vibration Exercises at 2 Different Frequencies Versus an Aerobic Exercise on Some Cardiovascular, Neuromotor and Musculoskeletal Parameters in Adult Patients With Obesity
94. ACUTE EFFECTS OF WHOLE-BODY VIBRATION ON JUMP FORCE AND JUMP RATE OF FORCE DEVELOPMENT: A COMPARATIVE STUDY OF DIFFERENT DEVICES
95. Acute effects of whole-body vibration on neuromuscular response of the vastus lateralis muscle: A comparative study of different devices
96. The Effect of Whole-Body Vibration on Lower-Body Resistance Detraining in College-Age Women
97. Effect of whole-body vibration on neuromuscular performance and body composition for females 65 years and older: a randomized-controlled trial
98. Impact of whole body vibration on balance improvement in elderly women
99. Combined Effects of Whole-Body Vibration, Resistance Exercise, and Vascular Occlusion on Skeletal Muscle and Performance
100. PROGRESSIVE-OVERLOAD WHOLE-BODY VIBRATION TRAINING AS PART OF PERIODIZED, OFF-SEASON STRENGTH TRAINING IN TRAINED WOMEN ATHLETES
101. A Comparison of Whole-Body Vibration and Resistance Training on Total Work in the Rotator Cuff
102. Effects of Whole-Body Vibration Training and Blood Flow Restriction on Muscle Adaptations in Women: A Randomized Controlled Trial
103. Effects of combined whole-body vibration and resistance training on muscular strength and bone metabolism in postmenopausal women
104. The acute effects of stretching with vibration on dynamic flexibility in young female gymnasts
105. Adding Whole-Body Vibration to Preconditioning Squat Exercise Increases Cycling Sprint Performance
106. Effects of Whole Body Vibration on the Neuromuscular Amplitude of Vastus Lateralis Muscle
107. Effect of Whole-Body Vibration on Delayed Onset Muscular Soreness, Flexibility, and Power
108. Acute and Chronic Whole-Body Vibration Exercise does not Induce Health-Promoting Effects on The Blood Profile
109. The immediate effect of whole body vibration training on the electromyographic activity of contralateral hand muscles; a randomized controlled trial
110. Effects of whole-body vibration and high impact exercises on the bone metabolism and functional mobility in postmenopausal women
111. Effect of Whole-Body Vibration Training on Selected Intrinsic Risk Factors in Women Aged 60+at Fall Risk: A Randomized Controlled Trial
112. Influence of vibration training on pain and quality of life in women older than 65 years old
113. Effects of Whole-Body Vibration Training with Different Body Positions and Amplitudes on Lower Limb Muscle Activity in Middle-Aged and Older Women
114. Determining the Posture and Vibration Frequency that Maximize Pelvic Floor Muscle Activity During Whole-Body Vibration
115. The effect of whole-body vibration frequency and amplitude on the myoelectric activity of vastus medialis and vastus lateralis
116. The Effects of A 10-week Whole-body Vibration Program on Balance And Lower Body Muscular Strength In Adult Women
117. The effects of twelve weeks of whole-body vibration training and low-intensity resistance exercise training on arterial function, muscle strength, and physical performance in dynapenic postmenopausal women
118. The Effect of Whole-Body Vibration Training on Arterial Stiffness, Blood Pressure, and Muscle Strength in Obese Postmenopausal Women
119. Whole body vibration affects gross motor performance measures during the Wingate anaerobic test
120. Effects of 8 months of twice-weekly high versus low intensity whole body vibration on risk factors for hip fracture in postmenopausal women: A randomized controlled trial
121. Long-term effects of whole-body vibration training in high-level female basketball players
122. EFFECTS OF VIBRATION TRAINING ON FORCE PRODUCTION IN FEMALE BASKETBALL PLAYERS
123. High-impact Routines to Ameliorate Trunk and Lower Limbs Flexibility in Women
124. High-frequency whole-body vibration improves balancing ability in elderly women
125. Effects of small-volume soccer and vibration training on body composition, aerobic fitness, and muscular PCr kinetics for inactive women aged 20-45
126. Effects of 24 weeks of whole body vibration training on body composition and muscle strength in untrained females
127. Whole body vibration training--improving balance control and muscle endurance
128. The effects of 11 weeks whole body vibration training on jump height, contractile properties and activation of human knee extensors
129. The Influence of Whole-Body Vibration on Creatine Kinase Activity and Jumping Performance in Young Basketball Players
130. Whole Body Vibrations on Functional Capacity, Muscular Strength, and Biochemical Profile in Elders
131. A 5-week whole body vibration training improves peak torque performance but has no effect on stretch reflex in healthy adults: a randomized controlled trial
132. Acute effects of stochastic and sinusoidal whole body vibration on pelvic floor muscle activation
